# Insertion of the Ca^2+^-Independent Phospholipase A_2_ into a Phospholipid Bilayer via Coarse-Grained and Atomistic Molecular Dynamics Simulations

**DOI:** 10.1371/journal.pcbi.1003156

**Published:** 2013-07-25

**Authors:** Denis Bucher, Yuan-Hao Hsu, Varnavas D. Mouchlis, Edward A. Dennis, J. Andrew McCammon

**Affiliations:** 1 Department of Chemistry and Biochemistry, University of California, San Diego, La Jolla, California, United States of America; 2 Center for Theoretical Biological Physics and National Biomedical Computation Resource, University of California, San Diego, La Jolla, California, United States of America; 3 Department of Chemistry, Tunghai University, Taichung, Taiwan; 4 Department of Pharmacology, University of California, San Diego, La Jolla, California, United States of America; 5 Howard Hughes Medical Institute, University of California, San Diego, La Jolla, California, United States of America; Tel Aviv University, Israel

## Abstract

Group VI Ca^2+^-independent phospholipase A_2_ (iPLA_2_) is a water-soluble enzyme that is active when associated with phospholipid membranes. Despite its clear pharmaceutical relevance, no X-ray or NMR structural information is currently available for the iPLA_2_ or its membrane complex. In this paper, we combine homology modeling with coarse-grained (CG) and all-atom (AA) molecular dynamics (MD) simulations to build structural models of iPLA_2_ in association with a phospholipid bilayer. CG-MD simulations of the membrane insertion process were employed to provide a starting point for an atomistic description. Six AA-MD simulations were then conducted for 60 ns, starting from different initial CG structures, to refine the membrane complex. The resulting structures are shown to be consistent with each other and with deuterium exchange mass spectrometry (DXMS) experiments, suggesting that our approach is suitable for the modeling of iPLA_2_ at the membrane surface. The models show that an anchoring region (residues 710–724) forms an amphipathic helix that is stabilized by the membrane. In future studies, the proposed iPLA_2_ models should provide a structural basis for understanding the mechanisms of lipid extraction and drug-inhibition. In addition, the dual-resolution approach discussed here should provide the means for the future exploration of the impact of lipid diversity and sequence mutations on the activity of iPLA_2_ and related enzymes.

## Author Summary

The Ca^2+^-independent phospholipase A_2_ (iPLA_2_) enzyme is a potential target for the development of medicinal agents against heart and neurological diseases, multiple sclerosis, arthritis, and cancer. However, no structural information is currently available for the iPLA_2_. The binding of the enzyme to human membranes is driven by favorable electrostatic and non-polar interactions, but the detailed influence of these factors is not well understood. In this paper, we have combined coarse-grained and all-atom simulations of a homology model of the iPLA_2_. The coarse-grained description allows highly efficient simulations of the protein insertion into a lipid bilayer, while the all-atom simulations are used to refine the structures of the protein–membrane complexes. Finally, the resulting structures are validated experimentally with deuterium exchange experiments. In future works, this approach could be used to build models of other PLA_2_s. The iPLA_2_ models presented here open the door to the computational design of new inhibitors with improved potency and selectivity.

## Introduction

Many membrane proteins remain unexplored at the molecular-level despite their clear pharmaceutical relevance [1], [2]. It is therefore crucial to develop computational methods for the structure prediction of membrane proteins. Homology modeling is a common technique to build an initial model when an appropriate template can be identified. Subsequently, all-atom (AA) molecular dynamics (MD) simulations have been used in the refinement of homology models with some success [3], [4]. However, for protein-membrane systems the construction of structural models is complicated by the need to equilibrate all the possible orientations of the protein in the membrane. Because the current time-scale accessed by AA-MD (hundreds of nanoseconds) is typically too short to simulate the complete insertion process directly, an effective approach to study membrane proteins is to start with a low-resolution model and subsequently go to higher resolution. Coarse-grained (CG) models for proteins [5] such as the MARTINI force field [6], [7] have been used to extend the time-scale of MD simulations by ∼3–4 orders of magnitude, allowing the direct simulation of membrane insertion processes. The force field performs roughly a 4 to 1 mapping between atoms and particles, which has been shown to be sufficiently accurate to study membrane insertion processes [8], [9], including for surface enzymes [10], [11]. However, like other resolution exchange methods [12], [13], this approach remains relatively new and untested and structural models should be validated experimentally whenever possible.

Phospholipase A_2_ (PLA_2_) [1] is one of the largest protein superfamilies identified to date, with 16 groups and many subgroups resulting in more than 35 forms, and represents a promising target for computer-aided drug design (CADD) [14]. All PLA_2_s stabilize at the membrane surface where they can catalyze the hydrolysis of phospholipids to yield fatty acids, involved in signaling, inflammation and in membrane maintenance [15]. The four predominant well-studied types of PLA_2_s found in human tissues are the cytosolic (also known as cPLA_2_), the secreted (sPLA_2_), the calcium-independent (iPLA_2_), and the lipoprotein-associated (Lp-PLA_2_) enzymes. The structures of PLA_2_s–bilayer complexes have been previously approached with deuterium exchange mass spectrometry (DXMS) [16]. These experiments provide information about the solvent accessible surface of the proteins by measuring the rate and number of backbone amide N-H groups that can exchange hydrogen with deuterium when in D_2_O. In this technique, the protein is first enzymatically digested into fragments of several residues in length and mass spectrometry is used to weight the fragments. The experiment is then repeated after the protein is inserted into a membrane to quantify the difference in the number of hydrogen atoms exchanged. These studies have helped define the location of the binding interface with the phospholipid membrane, and have shown that sPLA_2_, cPLA_2_, iPLA_2_ and Lp-PLA_2_ not only have different structures, but also very different membrane association mechanisms. For example, for sPLA_2_, a positively charged protein surface facilitates interactions with the anionic headgroups of the lipid surface [17]. For cPLA_2_, an additional domain (C2) directs the binding [18]. In the case of Lp-PLA_2_, two membrane association helices assist the membrane binding [19]. Finally, for iPLA_2_, an anchor region is directly inserted into the phospholipid membrane [20]. These studies also indicated that the catalytic residues stabilize at a location that is remote from the *sn*-2 position of phospholipids localized in bilayer membranes. This implies that the phospholipids must be extracted from the membrane to be enzymatically hydrolyzed [20].

**Figure 1:**
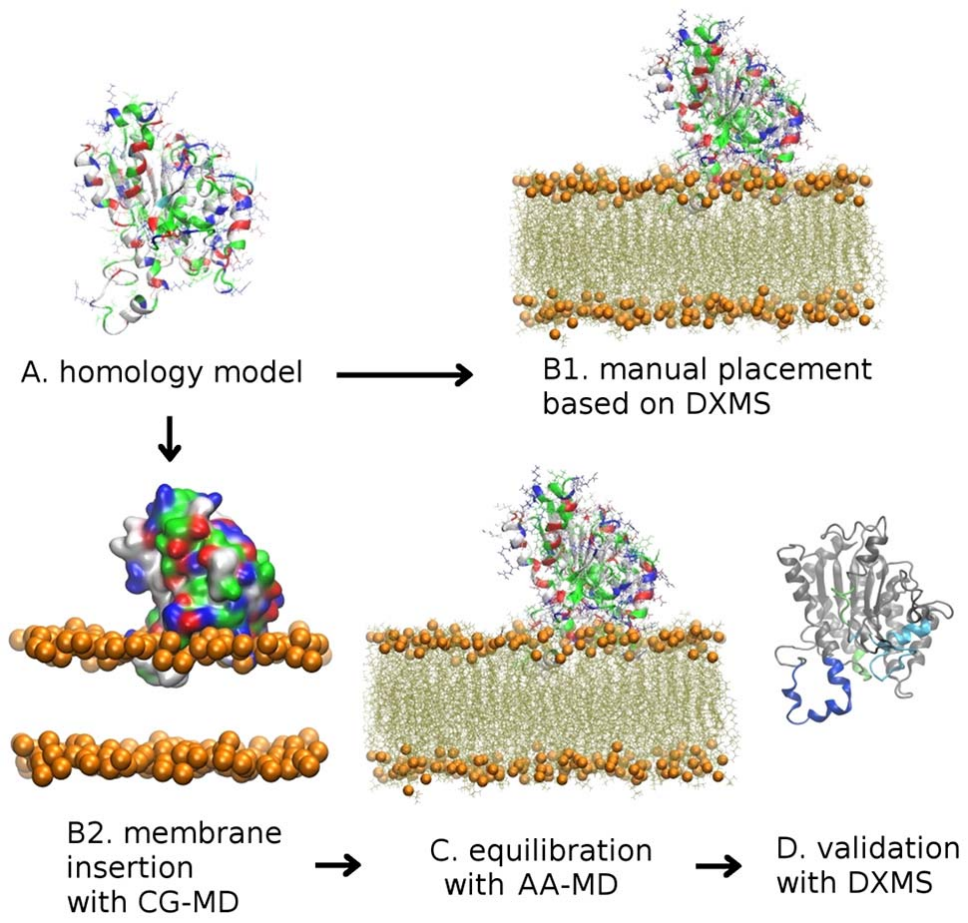
Multi-resolution approach for building 3D models of the iPLA_2_ catalytic domain at the membrane surface. The positioning of homology models at the membrane surface can be guided by DXMS experiments [20], (A→B1); however, this approach is subject to several approximations (see main text). Therefore, in this work, we follow a multi-scale simulation approach where a homology model is first transformed into a CG representation, and used to simulate the membrane insertion (A→B2). AA representations are then reverse-mapped from CG structures of the membrane complex, and equilibrated with extensive all-atom simulations, (C). The reproducibility of the resulting structures for the iPLA_2_–membrane complex is demonstrated, as well as the excellent agreement with DXMS experiments, (D). The protein residues are colored according to their polarity (green = polar; gray = hydrophobic; blue = positively charged; red = negatively charged). Orange spheres correspond to the position of phospholipid phosphate groups.

In this paper, we focus on the modeling of the iPLA_2_ enzyme at the membrane surface. The iPLA_2_ is of particular interest for structure-based drug design, as it is believed to be implicated in a large number of diseases, including Alzheimer disease [21], hypertensive heart failure [22], neurological disorders [23], multiple sclerosis [24], and cancer [25]. There is currently no X-ray or NMR information about the iPLA_2_ structure, or its membrane-associated complex. The chemical properties of the active site of iPLA_2_ are very similar to other PLA_2_s (in particular to cPLA_2_), which can cause inhibitors to display a lack of selectivity [26], leading to unwanted side-effects and toxicity. Thus, a current challenge for inhibitor design targeting iPLA_2_ lies in optimizing the potency and selectivity of promising new compounds, which can be aided by modeling based on DXMS and molecular dynamics [27], [28]. To assist the development of new therapeutic approaches, it is also crucial to understand the detailed interactions of PLA_2_ enzymes with phospholipid bilayers. Previously [20], a structural model for the iPLA_2_–membrane complex was built by homology modeling in combination with DXMS to guide the position of the enzyme model at the membrane surface (Figure 1). However, several approximations limit the accuracy of this approach for drug design applications. First, the DXMS data is subject to interpretation. For instance, the H/D exchange signal is typically averaged over many possible orientations and conformations of the protein, and it is measured only for the amide N-H bond with a resolution of several residues. Second, the use of a rigid protein model does not allow the protein to relax upon binding to the phospholipid bilayer. To overcome these limitations, in this paper we conduct CG-MD simulations to provide equilibrated models of the iPLA_2_–membrane complex. The structures were further refined with AA-MD simulations, and can be used to better understand the mechanisms of the iPLA_2_ membrane insertion and activation.

## Materials and Methods

### CG-MD simulations

Different subtypes and splice variants of iPLA_2_ exist in humans [1]; therefore, we have chosen to focus on the catalytic domain which is conserved throughout. Before conversion into a CG representation, an AA structure was first built by homology with the crystal structure of patatin. Patatin has ∼40% sequence similarity with iPLA_2_ and was solved at a resolution of 2.2 Å (Protein Data Bank code 1OXW) [29]. Prime [30] was used to build the 332 residue homology model. The ionizable side chains of the enzyme were chosen in their default charge states for pH 7 and histidine residues were kept uncharged. Because there is currently no NMR or X-ray information about the iPLA_2_ structure, the stability of the homology model was previously tested with MD simulations [28]. The ranking and scoring of docked compounds, as well as deuterium exchange experiments, in the presence, and in the absence of an inhibitor, also suggested that the active-site residues are well described in our model [28]. The most populated structural cluster in the AA simulations was used as the starting structure for generating the CG structural model. The same AA structure was also used for fitting atomic coordinates into equilibrated CG coordinates of the membrane complex, as described in the next subsection. All CG-MD simulations were conducted with GROMACS 4.5.4 [31]. The MARTINI 2.1 force field was used for the protein [7], together with non-polarizable CG water particles [6]. To maintain the protein secondary and tertiary structure an elastic network was applied composed of harmonic restraints (with a force constant of 10 kJ mol^−1^ Å^−2^) between all backbone particles within 7 Å of each other [32]. Palmitoyl oleoyl-phosphatidylcholine (POPC) molecules were used for the lipid membrane simulations, because the iPLA_2_ is known to be active on this type of membrane [33]. The final simulation system was neutral and contained the protein, 390 randomly positioned POPC molecules, and 11871 CG water particles. The chosen CG water model does not bear charges, and it is blind to electrostatic fields and polarization effects. To compensate for the neglect of explicit polarization, screening of electrostatic interactions is done implicitly, assuming a uniform relative dielectric constant of 15 that is smoothly shifted to zero between 0 and 12 Å [6]. The initial box dimensions were 140×140×140 Å^3^. Prior to the production runs, energy minimization was carried out for 5000 steps using a steepest-descent algorithm. The integration timestep in CG-MD simulations was 25 fs. Temperature was kept constant at 323 K using the velocity rescaling thermostat of Bussi *et al* [34]. A Berendsen barostat [35] was used to apply anisotropic pressure coupling, using a coupling constant of 10.0 ps, a compressibility value of 3×10^−5^ bar^−1^, and a reference pressure of 1 bar. Van der Waals interactions were also smoothly shifted to zero between 9 and 12 Å [6]. For simplicity, the time-scale of CG simulations is reported here without a scaling factor; it is however possible to use a scaling factor of ∼4 to account for the speed-up in the diffusive dynamics of the CG water model with respect to real water [7]. During the CG-MD simulations, the bilayer was found to self-assemble in the presence of the protein within ∼50 ns, leading to a local energy minimum. A total simulation time of ∼30 µs was accumulated to obtain ample statistics about the enzyme–membrane association process.

### AA-MD simulations

The AA models were built in VMD [36] by aligning our best equilibrated AA structure onto the CG structures for the membrane complex, using a least squares fitting of alpha carbons on CG particles. Following this, an equilibrated POPC membrane patch of area 103×103 Å^2^ was aligned on the CG membrane phosphate groups. Lipids within 0.6 Å of the protein were removed, and the system was solvated with TIP3P water. 42 Na^+^ and Cl^−^ ions were added to create a solution with a physiological ion concentration of ∼0.1 mol/L. The AA simulations were conducted in NAMD 2.9 [37] with the CHARMM36 force field [38], [39], using a time step of 2 fs in combination with the SHAKE algorithm [40]. Long-range electrostatic interactions were calculated using the particle mesh Ewald method, and van der Waals interactions utilized a cutoff of 10 Å. The temperature was regulated with a Langevin thermostat [41], using a damping coefficient of 5 ps^−1^. Energy-minimization was carried out for 10,000 steps, followed by an equilibration simulation in the NPT ensemble of 10 ns. Positional restraints (force constant 10 kJ mol^−1^ Å^−2^) were applied to the protein during equilibration, while the system was slowly heated-up, from 0 to 310 K, by 1 K every 4 ps. Water was prevented from entering the empty space between the protein and the membrane using a repulsive potential implemented in NAMD as an external Tcl script. At the end of the equilibration phase, the protein structure was released. Six AA-MD simulations were initiated from CG structures generated by extensive CG-MD sampling and separated by at least 1 µs. The six simulations were conducted in the NPT ensemble with isotropic pressure scaling, and lasted for 60 ns. The coordinates of an equilibrated AA system are included as supporting information (Text S1). To analyze the structure of the iPLA_2_–membrane complex, both the insertion depth and the insertion angle were monitored. We define the insertion angle as the angle between the long helix (residues 724 to 750) and its projection on the membrane surface. The depth of penetration is measured as the distances between the center-of-mass (c.o.m.) of the alpha carbons of anchor residues, and the c.o.m. of the bilayer along the bilayer normal (z axis). Finally, two additional AA-MD simulations in solution were conducted for 100 ns, in order to compare the properties of the iPLA_2_ catalytic domain in solution and in the membrane.

## Results

Our results are discussed in the following order: First, CG-MD simulations are reported for the protein insertion process. Second, we discuss the refinement of AA models generated from the CG structures for the membrane complex. Third, we describe residues in contact with the membrane and compare our results with H/D exchange experiments. Finally, we make some concluding comments about the mechanism of lipid extraction.

### CG-MD simulations of the protein insertion process

**Figure 2:**
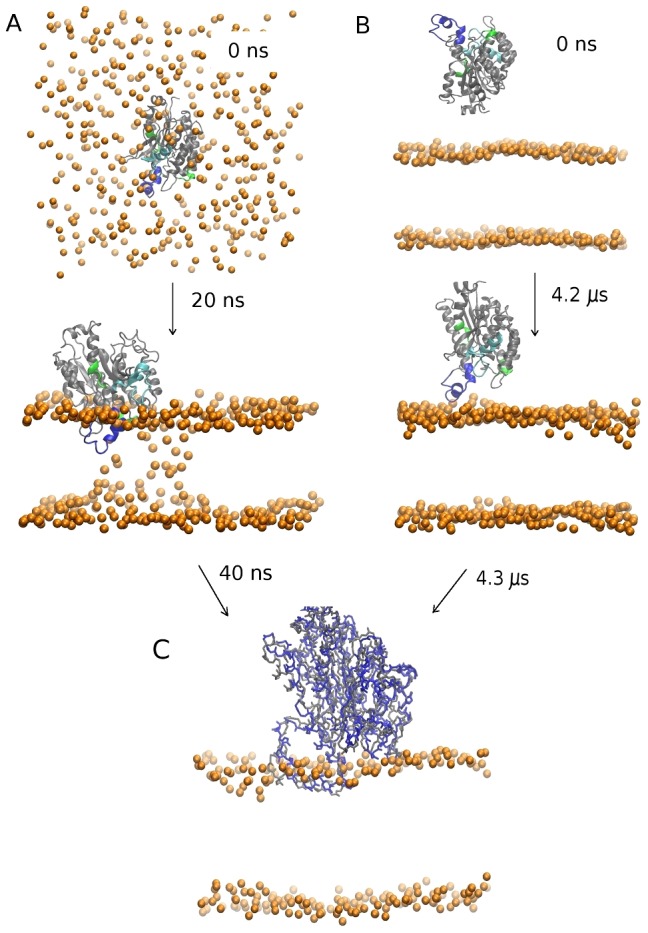
Snapshots from the CG-MD simulations showing the stabilization of the iPLA_2_ catalytic domain at the membrane surface. In (A), the membrane self-assembly was simulated starting from a random distribution of the phospholipids around the protein; and in (B), the membrane was formed at the beginning of the simulations with the protein in solution. The protein is colored in blue for residues that are inside the membrane according to DXMS results (see next subsections), and in green and light blue for residues that display only a weak decrement in H/D exchange consistent with a position above the membrane surface. Phospholipid phosphate particles are shown in orange. (C) The resulting structures for the iPLA_2_–membrane complex obtained with methods A and B are shown superimposed (backbone atoms only).

Two different approaches were employed to study the insertion of iPLA_2_ into a phospholipid bilayer with CG-MD (Figure 2). In the first approach (Figure 2A), ten CG-MD simulations of ∼500 ns were conducted starting from a random configuration of the lipids around the protein. The membrane was found to self-assemble within ∼50 ns into a fully formed phospholipid bilayer. In seven out of these ten simulations, the bilayer formed around the protein and the enzyme–membrane complex equilibrated with the enzyme adopting an interfacial location. In the other three simulations, the bilayer formed and the enzyme remained in the aqueous environment for the entire simulation. In the second approach (Figure 2B), the membrane was already formed and the protein was in solution, and three long CG-MD simulations were conducted. The time before the onset of anchoring was much longer in this case: ∼2.5 µs, 3.2 µs, and 4.1 µs. Inspection of the trajectories reveals that multiple collisions between the enzyme and the membrane occurred before the formation of the enzyme–membrane complex. More specifically, insertion occurred only when the enzyme collided with its anchoring region (residues 710 to 724) pointing toward the membrane, and therefore, ∼90% of the collisions between the protein and the membrane were unproductive. After adopting an interfacial location, iPLA_2_ remained at the interface for the remainder of each simulation, consistent with a stable configuration. In Figure 2C, an alignment of the resulting iPLA_2_–membrane structures with both approaches is shown. A limitation of the first approach is that the membrane can form too rapidly around the protein, and kinetically trap a less stable conformation that corresponds only to a local minimum. This occurred in two of the seven simulations, leading to a conformation with a lower insertion angle of ∼10 deg. The second CG-MD approach did not appear to suffer from this limitation, but it required hundred times longer simulations to observe successful insertion events.

### AA-MD refinement of the enzyme–membrane models

**Figure 3:**
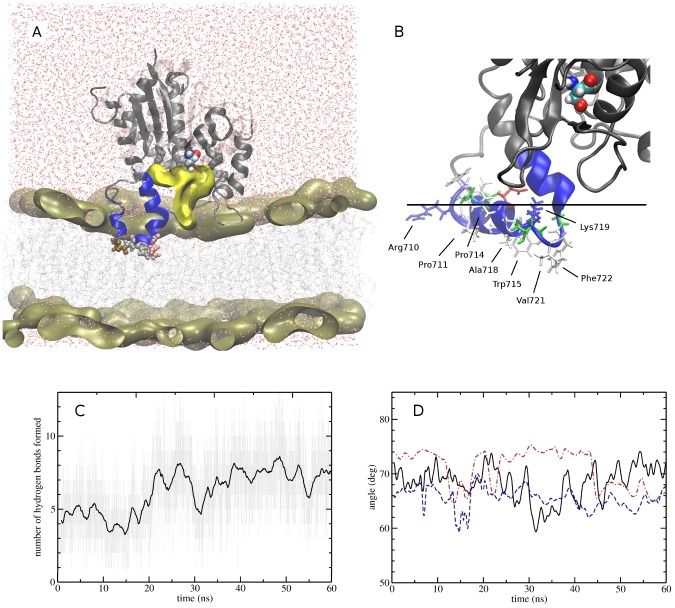
Atomistic MD simulation of the catalytic domain of iPLA_2_ in a lipid bilayer. (A) Cutaway view of the catalytic domain inserted into the lipid bilayers from a representative snapshot of the simulations. The protein is represented as gray ribbons. The lipid phosphate headgroups are shown as a pale brown surface. A blue section of the protein indicates the anchor region (residues 708–730), which shows significantly reduced H/D exchange when the enzyme is in the membrane. The active site Ser519 is shown in CPK representation. A yellow volume indicates a cavity at the entrance of the active-site, which may be employed for the lipid extraction. The inserted side chains of three anchoring residues, Pro714, Trp715, and Leu717, are shown in CPK representations in brown, gray, and pink. (B) Close-up caption of the cluster of hydrophobic residues (Pro711, Pro714, Trp715, Leu717, Val721, Phe722) that are interacting with the lipid membrane. A horizontal line was drawn to indicate the approximate position of phosphate groups. The protein side chains are colored according to their charges (green = polar; gray = hydrophobic; blue = positively charged; red = negatively charged). (C) Equilibration of hydrogen bond interactions between the protein and the lipid headgroups during a 60 ns AA-MD simulation. (D) Time series of the insertion angle in three 60 ns AA-MD simulations.

An important methodological question concerns the ability of multi-scale simulations to generate a unique atomistic structure for the iPLA_2_–membrane complex that corresponds to the global energy minimum. To bring back an all-atoms level of details, six AA-MD simulations were seeded from distinct CG structures of the catalytic domain at the membrane surface. AA-MD simulations were conducted for 60 ns to allow the relaxation of the protein structure that was previously restricted by the use of an elastic network. Detailed interactions such as hydrogen bonding are not represented in the chosen CG model, but were probed with AA-MD. We found that in all six AA-MD simulations, the equilibration of side-chains lead to additional hydrogen bonds with the phospholipids headgroups that equilibrated within ∼20 ns (Figure 3C). Importantly, no significant drift occurred in AA trajectories initiated from the CG structures, and for all six simulations the protein structures could be aligned with a root-mean-square deviation (RMSD) for backbone atoms below 4 Å. In addition, the residues in contact with the lipid headgroups/tails, and the insertion angle (67±8 deg), were in agreement between all simulations within the naturally occurring fluctuations (Figure 3C,D).

The stability of the AA models was further examined by MD relaxation from high-energy structures. Four new models were generated in two tense orientations on the membrane: two models were positioned perpendicularly 3 Å, and 6 Å, deeper into the membrane than the equilibrium model; the other two were tilted by +15 degree (deg), and −15 deg angles. After equilibration of the membrane for 10 ns, the protein was allowed to relax and the changes in the orientation and depth of the protein were monitored for over 40 ns. These simulations were conducted at 340 K to accelerate the protein relaxation. In all four simulations, the average displacement with respect to the initial structure was >6 Å and indicated that the protein has not yet reached a stable position after 40 ns. The simulations also showed a significant displacement of the enzyme out of the lipid bilayer when the insertion was too deep. Similarly, the tilted enzyme models rotate toward the original binding model as expected. Thus, in both relaxation experiments, the enzyme transits from a tense mode to a relaxed mode and slowly converges toward our best guess for the protein position, suggesting that it corresponds to a favorable conformation at the membrane surface. Therefore, we conclude that our multi-scale simulation approach is robust for determining the lowest energy structure for the iPLA_2_–membrane complex.

Figure 3A shows a representative snapshot of an equilibrated AA-MD simulation, and highlights the membrane surface, the anchoring region, and the contours of a protein cavity at the membrane surface. In all six equilibrated structures, the same surface of the protein was found to bind to the membrane. In particular, three regions are in direct contact with the membrane (regions 552–555, 643–646, and 710–724). The main contact region (710–724, shown in Figure 3B) folds into an amphipathic alpha helical structure that is inserted into the membrane. One side of the helix consists of a cluster of hydrophobic residues (Pro711, Pro714, Trp715, Leu717, Val721, and Phe722) that have their side-chains pointing toward the lipid tails and act as hydrophobic anchors. The other side of the helix consists of basic (Arg710, and Lys719) and polar residues (Ser712, Asn713, Glu716, Thr720, and Gly723) that mainly interact with the lipid headgroups. In addition, a second region (643–646) contains Try643 and Arg645 that can also interact with the membrane surface. Tyrosine and arginine residues are known to bind near the lipid tail/headgroups interface, as they can create both favorable hydrogen bonds and hydrophobic interactions. Finally, a third contact region (552–555) helps to stabilize the angle between the protein and the membrane. It is formed by Arg553 that can interact with the lipid headgroups, Pro554 that can interact with the lipid tails, as well as Ser552 and Tyr555 that can form hydrogen bonds with the headgroups. In the CG-MD simulations, the insertion angle was about ∼45 deg when Arg553 was unable to form a hydrogen bond with the lipid headgroups, but it equilibrated to >65 deg when this interaction was formed.

**Figure 4:**
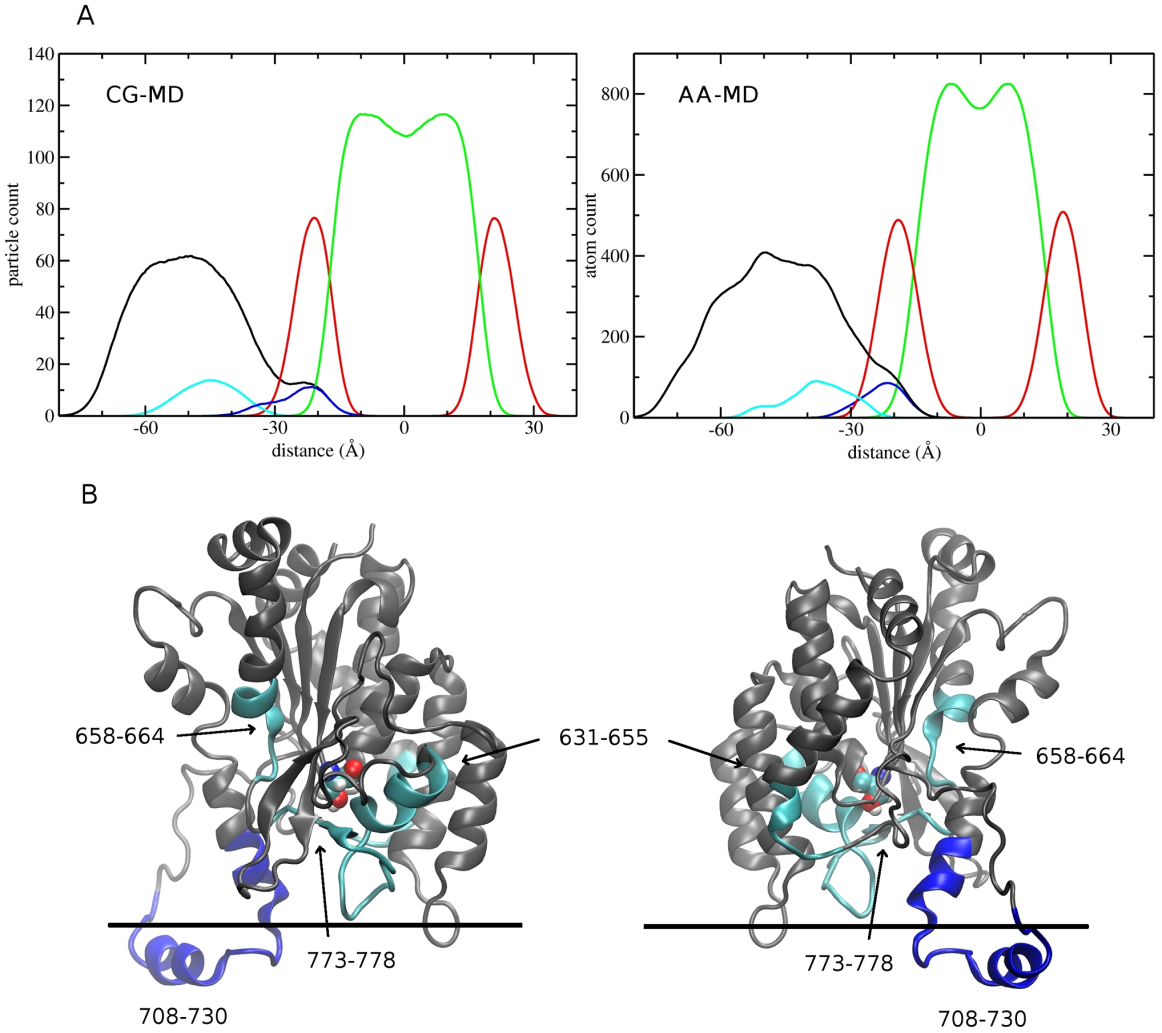
(A) Distribution of residues with respect to the components of the bilayer system (lipid headgroups and tails; red and green, respectively). The protein (black) was magnified ×3 times for better visualization. Also shown is the distribution of hydrophobic anchors (blue) and surface residues (ice-blue) that only showed a small decrement in H/D exchange when in contact with the membrane. (B) DXMS results are color-mapped on the protein model: gray residues did not show a decrement in H/D exchange when the protein was in the membrane, blue residues showed a >70% decrement after 5 min of incubation time, while ice-blue corresponds to a smaller <40% effect after 5 min. The approximate position of the phosphate peak is shown as a horizontal line. The active site Ser591 is drawn in a CPK representation.

The depth of penetration of iPLA_2_ into the lipid bilayer was assessed by measuring the distances between the center-of-mass (c.o.m.) of the alpha carbons relative to the c.o.m. of the bilayer, along the bilayer normal (z axis). In both CG-MD and AA-MD simulations, a similar distribution of residues was observed with respect to the components of the bilayer system (Figure 4A). Among the different surface residues, the positions of basic (Arg710, and Lys719) and polar residues (Ser712, Asn713, Glu716, Thr720, and Gly723) were found to coincide roughly with the phosphate peak, at ∼18.9 (±1.6) Å from the bilayers center (Table 1). The cluster of hydrophobic residues (Pro711, Pro714, Trp715, Leu717, Val721, and Phe722) was found to be inserted ∼3 Å deeper into the membrane in a region occupied by the lipid tails. The perturbation induced by the anchor residues on the vertical packing of the lipids was found to be small. In all six simulations, the location of the catalytic site residue Ser519 was at least 10 Å away from the lipid headgroups, and at an average distance of ∼36 Å from the membrane center. This strongly suggests that the phospholipid substrate molecule must be extracted from the membrane at the beginning of the catalytic reaction.

**Table 1:** Location of surface side-chains relative to the bilayer center.

Residue type	Residue	AA-MD
hydrophobic	Pro711	16.8±1.9
	Pro714	16.4±2.0
	Trp715	15.0±1.7
	Leu717	19.0±2.2
	Val721	15.2±1.6
	Phe722	13.7±1.6
		**16.0±1.8**
basic	Arg710	19.0±2.2
	Lys719	18.3±1.6
		**18.6±1.9**
polar	Ser712	19.4±1.8
	Asn713	20.6±2.0
	Glu716	20.7±1.7
	Thr720	17.2±2.0
	Gly723	18.9±1.6
		**19.3±1.8**

Average ± standard deviation (Å) of the distance between the center of mass of the residue and the center of mass of the bilayer. Averages were taken over the last 20 ns of six AA-MD simulations.

### Comparison between equilibrated structures and DXMS experiments

DXMS experiments were carried out on the Group VIA-2 iPLA_2_ enzyme, which is composed of seven consecutive N-terminal ankyrin repeats, a linker region, and a C-terminal phospholipase catalytic domain. Deuterium exchange on iPLA_2_ was carried out in the presence of the phospholipid substrate, palmitoyl-arachidonyl-phosphatidylcholine (PAPC), and a methyl arachidonyl fluorophosphonate (MAFP) inhibitor to prevent the digestion of the membrane. It identified four regions with significant changes in deuterium exchange upon membrane binding, all located in the catalytic domain (Figure 4B). The region 708–730 showed the largest deuteration levels (>90%) in solution, showing that it is solvent-exposed in the absence of phospholipid vesicles. However, in the presence of phospholipid vesicles, the same region did not become highly deuterated, suggesting that it is involved in the membrane anchoring and therefore no longer solvent accessible. When the incubation time in D_2_O was 10 s, mass spectrometry shows a difference of 13.2 in the average number of deuterium exchanges for this region. Similarly, in the case of a 5 min incubation time, a ∼70% decrease in deuteration levels was measured in the presence of the membrane. The computational models are consistent with this result, as they show that hydrophobic residues Val708, Phe709, Trp715, Leu717, Val721, Phe722, and Leu727, are no longer solvent-accessible in the protein-membrane complex. Interestingly, the negatively charged region 773–778 and the regions 631–655 and 658–664 also showed a decrease of deuteration that is however less pronounced than for the anchor residues (<40% decrease in deuteration levels after 5 min of incubation). In the computational models, these residues belong to a hydrophobic cavity at the membrane surface that leads to the active-site serine. A likely explanation for the weak observed DMXS effect is that a single phospholipid can transiently occupy the cavity, and prevent the solvent from accessing these residues. We are currently exploring this hypothesis in more details and will publish these results separately.

### AA-MD simulations of iPLA_2_ in solution

**Figure 5:**
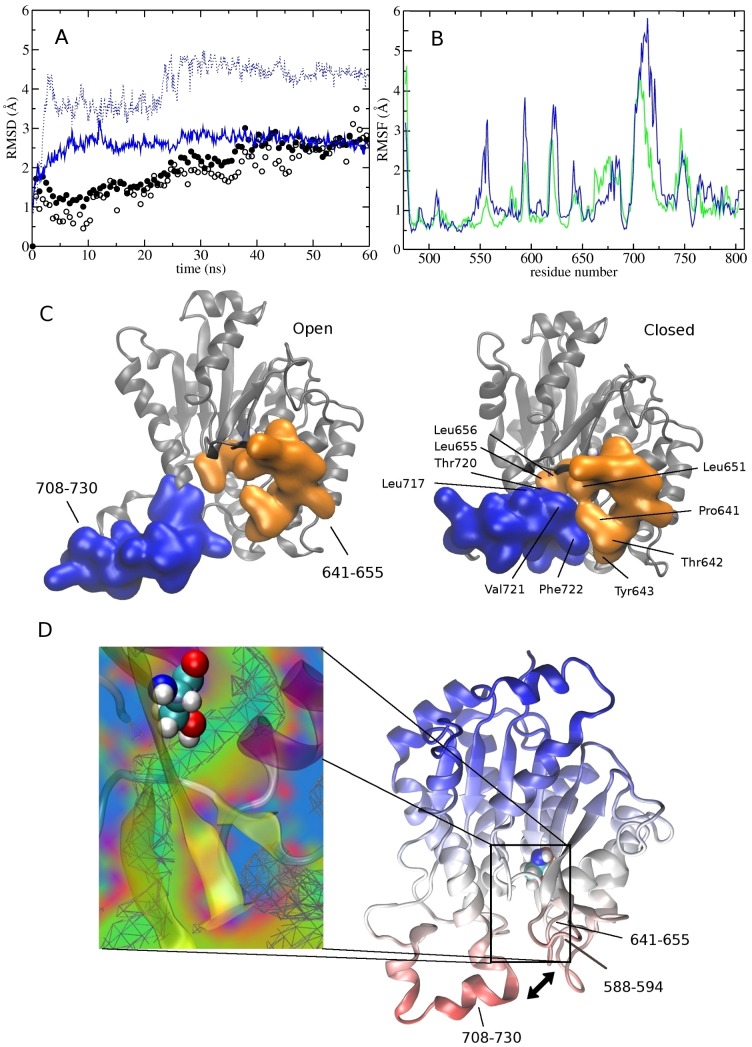
MD simulation of the iPLA_2_ catalytic domain in solution and in the membrane. (A) Root-mean-square-deviations (RMSD) of backbone atoms for the protein (in the membrane, and in solution, full, and empty circles, respectively), and for anchoring residues 708–730 (in the membrane, and in solution, solid blue line, and dotted line, respectively). (B) Root-mean-square-fluctuations (RMSF) of different residues when the protein is in the membrane (green) and in solution (blue). (C) In the absence of the membrane, the exposed hydrophobic residues of the anchor region are no longer stable in solution and can collapse, leasing to a closed conformation of the entrance cavity. (D) Calculated free energy pathway for a hydrophobic (methane) probe entering the active-site cavity when the protein is in the open conformation. Green-blue regions correspond to a favorable (negative) free energy.

In order to detect properties of the enzyme that change upon binding to the membrane, we compared two simulations of iPLA_2_, in solution, and in a membrane. In both these simulations, the RMSD for the protein backbone atoms was found to stabilize below 3 Å, which is indicative of a stable protein model (Figure 5A). Moreover, both simulations showed that the anchor region of iPLA_2_ (710–724) is very dynamic in nature (Figure 5B). However, in solution the amphipathic helix was observed to unfold, indicating that it is stabilized by the interaction with the membrane [42]. In addition, residues of the anchor region were found to move in solution by as much as ∼7 Å, and in some structures to block the entrance of the active-site cavity (Figure 5C). In particular, the bulky Tyr643 residue was found to act as a gatekeeper that can prevent the entrance of incoming ligands. To show this, the free energy profile was calculated for a methane probe entering the active-site with the implicit ligand sampling (ILS) method [43]. The ILS method allows the rapid post-processing an entire MD trajectory to collect qualitative information about the interaction free energy of a small probe. The calculation showed that a favorable free energy pathway exists connecting the membrane to the catalytic Ser519, which is accessible only in the membrane-activated (open) state of the protein (Figure 5D). This uncovers a new mechanism by which the flexible anchor region of iPLA_2_ may provide a form of active-site regulation similar to the lid structure in cPLA_2_.

## Discussion

The present study on iPLA_2_ complements two previous CG simulation studies [10], [44] that also demonstrated the utility of a multi-resolution simulation approach for predicting the surface location of PLA_2_ enzymes. These two previously published CG studies focused on the smaller sPLA_2_ (∼14–19 kDa), which has a known structure, and bears no sequence similarity with the much larger iPLA_2_ (∼84–90 kDa). In one study [10], it was found that the sPLA_2_ protein equilibrated further away from the membrane than suggested by experiments. However, the authors concluded that these differences were most likely due to difficulties in interpreting tryptophan fluorescence experiments, as no drift was observed in their AA-MD simulations originated from the CG models. In support for this explanation, no drift was observed here in the AA-MD simulations originated from the CG models, and the agreement between computer simulations and DXMS experiments was excellent for the prediction of membrane-bound residues.

Our results show that the catalytic domain of iPLA_2_ adopts a well-defined orientation at the membrane surface that is aimed to facilitate the vertical extraction of phospholipids from the membrane. Both the insertion depth and the orientation of iPLA_2_ on the lipid surface are therefore finely tuned to facilitate rapid turnover by coupling hydrolysis and product release with the binding of the next substrate [19], [20]. In addition, the enzyme was observed to diffuse laterally at the membrane surface without any disruption of the membrane complex, with the lipids dynamically repacking around the enzyme. This lateral motion is in agreement with the so-called scooting mechanism, which has been proposed to be important to facilitate the detection of lipid protrusion at the membrane surface [45]. The scooting mechanism contrasts with the hopping mechanism, in which the enzyme dissociates and re-associates with the membrane to explore the lipid surface. The hopping mechanism is believed to be favored by sPLA_2_ in the presence of zwitterionic membranes [44].

The Group VIA-2 splice variant of the iPLA_2_ contains in addition to the catalytic domain, seven ankyrin repeats, and a linker region (∼700 residues). The ankyrin repeats are believed to directly or indirectly assist membrane association because the catalytic domain by itself does not have activity [46]. Our efforts to simulate the full GVIA-2 iPLA_2_ structure did not uncover an alternative mechanism for the membrane insertion in the presence of ankyrin repeats. In particular, two CG-MD simulations of the full GVIA-2 iPLA_2_ structure were conducted for 500 ns starting from a random configuration of the lipids. After insertion into the membrane, no significant differences were observed in the catalytic domain region, suggesting that the catalytic domain *alone* is able to successfully complete the insertion process. However, it cannot be excluded that the additional structural elements increase the probability of membrane insertion, either by providing a second interaction point with the membrane, or by stabilizing the correct orientation of the catalytic domain. Moreover, the rotation of the catalytic domain around an axis perpendicular to the membrane plane was hindered in the full protein structure, which may be indicative of a more stable protein–membrane complex. Group VIA iPLA_2_ has also been shown to be active as an oligomer through radiation inactivation studies [47]; thus, the ankyrin repeats may be crucial for stabilizing the complex by taking part in the assembly of an oligomeric structure.

For a successful hydrolysis of phospholipids, one substrate molecule must be extracted from the lipid aggregate into the active-site of iPLA_2_. Because natural fluctuations in the fluid membrane mostly cause lateral motions in the lipid molecules, an exquisite mechanism must exist to allow the lipid molecule to escape the lipid surface. The structure of the iPLA_2_–membrane complex shows the existence of a hydrophobic cavity near the membrane surface that is likely to assist the lipid extraction by competing with hydrophobic interactions between the substrate and the lipid aggregates. The deuterium exchange experiments also support a scenario in which the cavity is often transiently occupied by a lipid substrate extracted from the membrane. However, the lipid extraction could not be directly observed in our simulations, presumably because the lipid-binding cavity remained closed in the CG simulations due to the use of an elastic network model to stabilize the protein structure. In the AA simulations the time-scale (nanoseconds) was too short to observe the opening of the cavity and the extraction of a lipid from the membrane. Ongoing work in our labs will address this problem by conducting longer time-scale (microsecond) AA simulations, as well as steered MD simulations [48], and provide a more complete picture of the lipid extraction.

The high degree of conformational flexibility of iPLA_2_ during simulations leads us to believe that the flexible loops that form the entrance of the cavity regulate the active-site accessibility. For instance, the hydrophobic residues in the anchor region (710 to 724) fold into an amphipathic helix in the membrane, but adopt an extended conformation when they are in solution, leading to the partial closure of the active-site cavity. We [27] have recently hypothesized that each type of PLA_2_ contains a distinct “membrane interaction site(s)” that should be considered as a typical allosteric site. When the PLA_2_ is associated with a ligand (in this case, the membrane), the enzyme exists in a different conformational state than in solution (R to T transition) in accord with the basic ideas of allostery [49] as recently reviewed by Changeux [50]. Although further work will be needed to confidently map the conformational landscape of iPLA_2_, the results reported herein are consistent with the novel notion of considering the membrane as a ligand, which causes a conformational change in certain water-soluble proteins (such as various PLA_2_s) when they productively associate with a membrane [27]. In addition, the present study identified an amphipathic helix as the anchor region of iPLA_2_. Amphipathic helices have been recently proposed as interesting motifs that help recognize hydrophobic defects in the membrane, such as those created when bending the bilayer [42], [51], [52]. It is possible that the helix in iPLA_2_ confers the enzyme with the ability to detect membrane defects, which is believed to be a key property of PLA_2_s [45].

In future studies, a deeper understanding should be gained of the roles of different protein conformations accessed during the catalytic cycle of iPLA_2_. This will be crucial for inhibitor design, as interrupting a single catalytic step could be sufficient to inhibit the entire reaction. MD simulations could be utilized to explore the role of the enzyme flexibility during the different phases of the catalytic process, including the extraction, binding, and hydrolysis of the substrate and the release of the products in the bilayer. These models will open the door to virtual screening techniques aimed at finding new inhibitors of iPLA_2_ with improved potency and selectivity. Finally, we feel that the multi-scale approach discussed here should prove helpful in designing initial computational models of various PLA_2_ enzymes at the membrane surface, and will lead to further studies on the impact of lipid diversity and sequence mutations on the activity of iPLA_2_ and related enzymes.

## Supporting Information

Text S1 Atomic coordinates of the protein-membrane system after AA-MD refinement, in the protein database (PDB) format.
(DOC)
