# Supplementary material for: Insertion of the Ca2+-Independent Phospholipase A2 into a Phospholipid Bilayer via Coarse-Grained and Atomistic Molecular Dynamics Simulations
Source: PLoS Comput Biol. 2013 Jul 25;9(7):e1003156. doi: 10.1371/journal.pcbi.1003156 (PMC3723492; doi:10.1371/journal.pcbi.1003156)
Supplement: Text S1 — Atomic coordinates of the protein-membrane system after AA-MD refinement, in the protein database (PDB) format. (DOC) [file pcbi.1003156.s001.doc]

CRYST1 100.000 100.000 120.000 90.00 90.00 90.00 P 1 1

ATOM 1 N POPCO 1 -42.284 -47.357 21.137 1.00 0.00 O1 N

ATOM 2 C12 POPCO 1 -41.557 -46.575 22.255 1.00 0.00 O1 C

ATOM 3 H12A POPCO 1 -40.792 -45.931 21.659 1.00 0.00 O1 H

ATOM 4 H12B POPCO 1 -41.035 -47.263 22.932 1.00 0.00 O1 H

ATOM 5 C13 POPCO 1 -42.408 -46.598 19.912 1.00 0.00 O1 C

ATOM 6 H13A POPCO 1 -41.494 -46.092 19.694 1.00 0.00 O1 H

ATOM 7 H13B POPCO 1 -43.065 -45.758 19.981 1.00 0.00 O1 H

ATOM 8 H13C POPCO 1 -42.691 -47.233 19.057 1.00 0.00 O1 H

ATOM 9 C14 POPCO 1 -41.649 -48.638 20.820 1.00 0.00 O1 C

ATOM 10 H14A POPCO 1 -41.564 -49.193 21.732 1.00 0.00 O1 H

ATOM 11 H14B POPCO 1 -40.613 -48.414 20.520 1.00 0.00 O1 H

ATOM 12 H14C POPCO 1 -42.133 -49.374 20.135 1.00 0.00 O1 H

ATOM 13 C15 POPCO 1 -43.693 -47.770 21.555 1.00 0.00 O1 C

ATOM 14 H15A POPCO 1 -43.750 -48.293 22.527 1.00 0.00 O1 H

ATOM 15 H15B POPCO 1 -44.210 -46.887 21.787 1.00 0.00 O1 H

ATOM 16 H15C POPCO 1 -44.197 -48.411 20.826 1.00 0.00 O1 H

ATOM 17 C11 POPCO 1 -42.451 -45.494 23.052 1.00 0.00 O1 C

ATOM 18 H11A POPCO 1 -43.132 -46.184 23.622 1.00 0.00 O1 H

ATOM 19 H11B POPCO 1 -41.771 -44.843 23.652 1.00 0.00 O1 H

ATOM 20 P POPCO 1 -44.526 -44.144 22.620 1.00 0.00 O1 P

ATOM 21 O13 POPCO 1 -44.664 -43.570 23.957 1.00 0.00 O1 O

ATOM 22 O14 POPCO 1 -45.407 -45.273 22.300 1.00 0.00 O1 O

ATOM 23 O12 POPCO 1 -43.051 -44.576 22.217 1.00 0.00 O1 O

ATOM 24 O11 POPCO 1 -44.762 -43.044 21.479 1.00 0.00 O1 O

ATOM 25 C1 POPCO 1 -44.521 -43.256 20.122 1.00 0.00 O1 C

ATOM 26 HA POPCO 1 -43.466 -42.990 19.875 1.00 0.00 O1 H

ATOM 27 HB POPCO 1 -44.714 -44.274 19.798 1.00 0.00 O1 H

ATOM 28 C2 POPCO 1 -45.387 -42.332 19.183 1.00 0.00 O1 C

ATOM 29 HS POPCO 1 -46.408 -42.468 19.476 1.00 0.00 O1 H

ATOM 30 O21 POPCO 1 -45.209 -42.642 17.729 1.00 0.00 O1 O

ATOM 31 C21 POPCO 1 -46.039 -42.052 16.874 1.00 0.00 O1 C

ATOM 32 O22 POPCO 1 -47.067 -41.443 17.194 1.00 0.00 O1 O

ATOM 33 C22 POPCO 1 -45.379 -42.027 15.482 1.00 0.00 O1 C

ATOM 34 H2R POPCO 1 -44.847 -41.122 15.290 1.00 0.00 O1 H

ATOM 35 H2S POPCO 1 -44.759 -42.878 15.277 1.00 0.00 O1 H

ATOM 36 C3 POPCO 1 -45.089 -40.797 19.399 1.00 0.00 O1 C

ATOM 37 HX POPCO 1 -45.621 -40.224 18.630 1.00 0.00 O1 H

ATOM 38 HY POPCO 1 -45.490 -40.569 20.387 1.00 0.00 O1 H

ATOM 39 O31 POPCO 1 -43.681 -40.601 19.381 1.00 0.00 O1 O

ATOM 40 C31 POPCO 1 -43.286 -39.407 19.645 1.00 0.00 O1 C

ATOM 41 O32 POPCO 1 -44.025 -38.459 19.820 1.00 0.00 O1 O

ATOM 42 C32 POPCO 1 -41.729 -39.364 19.638 1.00 0.00 O1 C

ATOM 43 H2X POPCO 1 -41.318 -38.430 20.096 1.00 0.00 O1 H

ATOM 44 H2Y POPCO 1 -41.381 -40.182 20.266 1.00 0.00 O1 H

ATOM 45 C23 POPCO 1 -46.514 -42.129 14.390 1.00 0.00 O1 C

ATOM 46 H3R POPCO 1 -46.960 -43.106 14.411 1.00 0.00 O1 H

ATOM 47 H3S POPCO 1 -47.334 -41.405 14.472 1.00 0.00 O1 H

ATOM 48 C24 POPCO 1 -45.877 -42.240 12.972 1.00 0.00 O1 C

ATOM 49 H4R POPCO 1 -45.281 -41.343 12.912 1.00 0.00 O1 H

ATOM 50 H4S POPCO 1 -45.130 -43.048 12.955 1.00 0.00 O1 H

ATOM 51 C25 POPCO 1 -46.708 -42.384 11.715 1.00 0.00 O1 C

ATOM 52 H5R POPCO 1 -47.333 -43.293 11.815 1.00 0.00 O1 H

ATOM 53 H5S POPCO 1 -47.462 -41.517 11.654 1.00 0.00 O1 H

ATOM 54 C26 POPCO 1 -45.946 -42.454 10.396 1.00 0.00 O1 C

ATOM 55 H6R POPCO 1 -45.454 -41.481 10.257 1.00 0.00 O1 H

ATOM 56 H6S POPCO 1 -45.175 -43.301 10.462 1.00 0.00 O1 H

ATOM 57 C27 POPCO 1 -46.790 -42.674 9.130 1.00 0.00 O1 C

ATOM 58 H7R POPCO 1 -47.137 -43.749 9.110 1.00 0.00 O1 H

ATOM 59 H7S POPCO 1 -47.768 -42.039 9.147 1.00 0.00 O1 H

ATOM 60 C28 POPCO 1 -46.096 -42.172 7.844 1.00 0.00 O1 C

ATOM 61 H8R POPCO 1 -46.108 -41.017 8.021 1.00 0.00 O1 H

ATOM 62 H8S POPCO 1 -45.025 -42.547 7.683 1.00 0.00 O1 H

ATOM 63 C29 POPCO 1 -46.954 -42.554 6.598 1.00 0.00 O1 C

ATOM 64 H91 POPCO 1 -47.922 -42.012 6.456 1.00 0.00 O1 H

ATOM 65 C210 POPCO 1 -46.610 -43.367 5.518 1.00 0.00 O1 C

ATOM 66 H101 POPCO 1 -47.415 -43.433 4.819 1.00 0.00 O1 H

ATOM 67 C211 POPCO 1 -45.342 -44.158 5.249 1.00 0.00 O1 C

ATOM 68 H11R POPCO 1 -44.489 -43.930 6.001 1.00 0.00 O1 H

ATOM 69 H11S POPCO 1 -45.568 -45.151 5.489 1.00 0.00 O1 H

ATOM 70 C212 POPCO 1 -44.736 -43.912 3.921 1.00 0.00 O1 C

ATOM 71 H12R POPCO 1 -44.414 -42.860 3.826 1.00 0.00 O1 H

ATOM 72 H12S POPCO 1 -43.878 -44.554 3.751 1.00 0.00 O1 H

ATOM 73 C213 POPCO 1 -45.592 -44.150 2.697 1.00 0.00 O1 C

ATOM 74 H13R POPCO 1 -45.875 -45.215 2.653 1.00 0.00 O1 H

ATOM 75 H13S POPCO 1 -46.521 -43.499 2.670 1.00 0.00 O1 H

ATOM 76 C214 POPCO 1 -44.832 -43.736 1.451 1.00 0.00 O1 C

ATOM 77 H14R POPCO 1 -44.690 -42.686 1.535 1.00 0.00 O1 H

ATOM 78 H14S POPCO 1 -43.907 -44.269 1.445 1.00 0.00 O1 H

ATOM 79 C215 POPCO 1 -45.502 -44.095 0.122 1.00 0.00 O1 C

ATOM 80 H15R POPCO 1 -45.644 -45.199 0.024 1.00 0.00 O1 H

ATOM 81 H15S POPCO 1 -46.457 -43.588 0.283 1.00 0.00 O1 H

ATOM 82 C216 POPCO 1 -44.767 -43.555 -1.155 1.00 0.00 O1 C

ATOM 83 H16R POPCO 1 -45.028 -42.497 -1.314 1.00 0.00 O1 H

ATOM 84 H16S POPCO 1 -43.671 -43.644 -1.063 1.00 0.00 O1 H

ATOM 85 C217 POPCO 1 -45.157 -44.266 -2.461 1.00 0.00 O1 C

ATOM 86 H17R POPCO 1 -45.252 -45.413 -2.303 1.00 0.00 O1 H

ATOM 87 H17S POPCO 1 -46.174 -43.824 -2.785 1.00 0.00 O1 H

ATOM 88 C218 POPCO 1 -44.146 -44.109 -3.558 1.00 0.00 O1 C

ATOM 89 H18R POPCO 1 -44.461 -44.806 -4.367 1.00 0.00 O1 H

ATOM 90 H18S POPCO 1 -44.124 -43.087 -3.891 1.00 0.00 O1 H

ATOM 91 H18T POPCO 1 -43.205 -44.474 -3.126 1.00 0.00 O1 H

ATOM 92 C33 POPCO 1 -41.105 -39.563 18.222 1.00 0.00 O1 C

ATOM 93 H3X POPCO 1 -41.328 -38.615 17.673 1.00 0.00 O1 H

ATOM 94 H3Y POPCO 1 -39.980 -39.505 18.286 1.00 0.00 O1 H

ATOM 95 C34 POPCO 1 -41.529 -40.779 17.375 1.00 0.00 O1 C

ATOM 96 H4X POPCO 1 -41.389 -41.737 17.952 1.00 0.00 O1 H

ATOM 97 H4Y POPCO 1 -42.632 -40.683 17.070 1.00 0.00 O1 H

ATOM 98 C35 POPCO 1 -40.817 -40.740 16.007 1.00 0.00 O1 C

ATOM 99 H5X POPCO 1 -40.800 -39.746 15.570 1.00 0.00 O1 H

ATOM 100 H5Y POPCO 1 -39.724 -40.964 16.128 1.00 0.00 O1 H

ATOM 101 C36 POPCO 1 -41.405 -41.681 14.940 1.00 0.00 O1 C

ATOM 102 H6X POPCO 1 -41.221 -42.747 15.312 1.00 0.00 O1 H

ATOM 103 H6Y POPCO 1 -42.534 -41.557 14.860 1.00 0.00 O1 H

ATOM 104 C37 POPCO 1 -40.915 -41.563 13.546 1.00 0.00 O1 C

ATOM 105 H7X POPCO 1 -41.065 -40.493 13.219 1.00 0.00 O1 H

ATOM 106 H7Y POPCO 1 -39.847 -41.722 13.414 1.00 0.00 O1 H

ATOM 107 C38 POPCO 1 -41.588 -42.313 12.401 1.00 0.00 O1 C

ATOM 108 H8X POPCO 1 -41.565 -43.355 12.753 1.00 0.00 O1 H

ATOM 109 H8Y POPCO 1 -42.627 -41.916 12.529 1.00 0.00 O1 H

ATOM 110 C39 POPCO 1 -40.882 -42.082 10.979 1.00 0.00 O1 C

ATOM 111 H9X POPCO 1 -40.918 -40.965 10.722 1.00 0.00 O1 H

ATOM 112 H9Y POPCO 1 -39.835 -42.541 11.076 1.00 0.00 O1 H

ATOM 113 C310 POPCO 1 -41.531 -42.669 9.738 1.00 0.00 O1 C

ATOM 114 H10X POPCO 1 -41.665 -43.756 9.888 1.00 0.00 O1 H

ATOM 115 H10Y POPCO 1 -42.381 -42.130 9.555 1.00 0.00 O1 H

ATOM 116 C311 POPCO 1 -40.616 -42.422 8.535 1.00 0.00 O1 C

ATOM 117 H11X POPCO 1 -40.255 -41.325 8.483 1.00 0.00 O1 H

ATOM 118 H11Y POPCO 1 -39.755 -43.067 8.618 1.00 0.00 O1 H

ATOM 119 C312 POPCO 1 -41.415 -42.575 7.127 1.00 0.00 O1 C

ATOM 120 H12X POPCO 1 -41.897 -43.562 7.139 1.00 0.00 O1 H

ATOM 121 H12Y POPCO 1 -42.265 -41.858 7.153 1.00 0.00 O1 H

ATOM 122 C313 POPCO 1 -40.434 -42.339 5.928 1.00 0.00 O1 C

ATOM 123 H13X POPCO 1 -40.309 -41.228 5.976 1.00 0.00 O1 H

ATOM 124 H13Y POPCO 1 -39.468 -42.817 6.254 1.00 0.00 O1 H

ATOM 125 C314 POPCO 1 -40.964 -42.832 4.591 1.00 0.00 O1 C

ATOM 126 H14X POPCO 1 -41.023 -43.989 4.532 1.00 0.00 O1 H

ATOM 127 H14Y POPCO 1 -42.011 -42.345 4.528 1.00 0.00 O1 H

ATOM 128 C315 POPCO 1 -40.113 -42.388 3.479 1.00 0.00 O1 C

ATOM 129 H15X POPCO 1 -40.149 -41.259 3.406 1.00 0.00 O1 H

ATOM 130 H15Y POPCO 1 -39.107 -42.686 3.702 1.00 0.00 O1 H

ATOM 131 C316 POPCO 1 -40.650 -42.850 2.121 1.00 0.00 O1 C

ATOM 132 H16X POPCO 1 -41.411 -42.165 1.778 1.00 0.00 O1 H

ATOM 133 H16Y POPCO 1 -39.859 -42.645 1.328 1.00 0.00 O1 H

ATOM 134 H16Z POPCO 1 -40.899 -43.957 2.212 1.00 0.00 O1 H

ATOM 135 N POPCO 2 -36.230 -34.031 19.926 1.00 0.00 O1 N

ATOM 136 C12 POPCO 2 -35.752 -33.865 18.474 1.00 0.00 O1 C

ATOM 137 H12A POPCO 2 -34.780 -34.315 18.442 1.00 0.00 O1 H

ATOM 138 H12B POPCO 2 -35.698 -32.783 18.228 1.00 0.00 O1 H

ATOM 139 C13 POPCO 2 -36.110 -35.396 20.464 1.00 0.00 O1 C

ATOM 140 H13A POPCO 2 -35.060 -35.648 20.535 1.00 0.00 O1 H

ATOM 141 H13B POPCO 2 -36.503 -36.157 19.741 1.00 0.00 O1 H

ATOM 142 H13C POPCO 2 -36.545 -35.501 21.472 1.00 0.00 O1 H

ATOM 143 C14 POPCO 2 -35.252 -33.340 20.838 1.00 0.00 O1 C

ATOM 144 H14A POPCO 2 -35.356 -32.240 20.682 1.00 0.00 O1 H

ATOM 145 H14B POPCO 2 -34.212 -33.705 20.710 1.00 0.00 O1 H

ATOM 146 H14C POPCO 2 -35.401 -33.580 21.818 1.00 0.00 O1 H

ATOM 147 C15 POPCO 2 -37.620 -33.547 20.059 1.00 0.00 O1 C

ATOM 148 H15A POPCO 2 -37.863 -32.512 19.665 1.00 0.00 O1 H

ATOM 149 H15B POPCO 2 -38.288 -34.197 19.557 1.00 0.00 O1 H

ATOM 150 H15C POPCO 2 -37.860 -33.560 21.114 1.00 0.00 O1 H

ATOM 151 C11 POPCO 2 -36.653 -34.474 17.343 1.00 0.00 O1 C

ATOM 152 H11A POPCO 2 -37.622 -33.840 17.359 1.00 0.00 O1 H

ATOM 153 H11B POPCO 2 -36.164 -34.360 16.310 1.00 0.00 O1 H

ATOM 154 P POPCO 2 -38.172 -36.553 17.918 1.00 0.00 O1 P

ATOM 155 O13 POPCO 2 -39.117 -35.472 18.304 1.00 0.00 O1 O

ATOM 156 O14 POPCO 2 -37.853 -37.529 18.932 1.00 0.00 O1 O

ATOM 157 O12 POPCO 2 -36.801 -35.931 17.442 1.00 0.00 O1 O

ATOM 158 O11 POPCO 2 -38.705 -37.180 16.650 1.00 0.00 O1 O

ATOM 159 C1 POPCO 2 -38.335 -36.914 15.310 1.00 0.00 O1 C

ATOM 160 HA POPCO 2 -37.443 -36.314 15.118 1.00 0.00 O1 H

ATOM 161 HB POPCO 2 -38.005 -37.859 14.814 1.00 0.00 O1 H

ATOM 162 C2 POPCO 2 -39.565 -36.437 14.498 1.00 0.00 O1 C

ATOM 163 HS POPCO 2 -40.280 -37.331 14.523 1.00 0.00 O1 H

ATOM 164 O21 POPCO 2 -39.156 -36.125 13.131 1.00 0.00 O1 O

ATOM 165 C21 POPCO 2 -39.505 -37.023 12.146 1.00 0.00 O1 C

ATOM 166 O22 POPCO 2 -40.162 -38.024 12.258 1.00 0.00 O1 O

ATOM 167 C22 POPCO 2 -38.992 -36.451 10.835 1.00 0.00 O1 C

ATOM 168 H2R POPCO 2 -39.322 -35.407 10.898 1.00 0.00 O1 H

ATOM 169 H2S POPCO 2 -37.832 -36.451 10.836 1.00 0.00 O1 H

ATOM 170 C3 POPCO 2 -40.250 -35.275 15.168 1.00 0.00 O1 C

ATOM 171 HX POPCO 2 -41.144 -35.042 14.679 1.00 0.00 O1 H

ATOM 172 HY POPCO 2 -40.642 -35.575 16.237 1.00 0.00 O1 H

ATOM 173 O31 POPCO 2 -39.363 -34.134 15.281 1.00 0.00 O1 O

ATOM 174 C31 POPCO 2 -39.778 -32.941 15.237 1.00 0.00 O1 C

ATOM 175 O32 POPCO 2 -40.950 -32.597 15.293 1.00 0.00 O1 O

ATOM 176 C32 POPCO 2 -38.586 -31.961 15.124 1.00 0.00 O1 C

ATOM 177 H2X POPCO 2 -38.925 -31.186 15.827 1.00 0.00 O1 H

ATOM 178 H2Y POPCO 2 -37.736 -32.391 15.634 1.00 0.00 O1 H

ATOM 179 C23 POPCO 2 -39.583 -37.174 9.628 1.00 0.00 O1 C

ATOM 180 H3R POPCO 2 -39.218 -38.229 9.639 1.00 0.00 O1 H

ATOM 181 H3S POPCO 2 -40.664 -37.137 9.764 1.00 0.00 O1 H

ATOM 182 C24 POPCO 2 -39.119 -36.447 8.379 1.00 0.00 O1 C

ATOM 183 H4R POPCO 2 -39.396 -35.384 8.509 1.00 0.00 O1 H

ATOM 184 H4S POPCO 2 -38.011 -36.490 8.367 1.00 0.00 O1 H

ATOM 185 C25 POPCO 2 -39.732 -36.946 7.085 1.00 0.00 O1 C

ATOM 186 H5R POPCO 2 -39.602 -37.988 6.956 1.00 0.00 O1 H

ATOM 187 H5S POPCO 2 -40.783 -36.645 7.322 1.00 0.00 O1 H

ATOM 188 C26 POPCO 2 -39.358 -36.051 5.811 1.00 0.00 O1 C

ATOM 189 H6R POPCO 2 -39.388 -35.055 6.125 1.00 0.00 O1 H

ATOM 190 H6S POPCO 2 -38.312 -36.174 5.411 1.00 0.00 O1 H

ATOM 191 C27 POPCO 2 -40.393 -36.242 4.675 1.00 0.00 O1 C

ATOM 192 H7R POPCO 2 -40.384 -37.354 4.356 1.00 0.00 O1 H

ATOM 193 H7S POPCO 2 -41.401 -35.934 4.976 1.00 0.00 O1 H

ATOM 194 C28 POPCO 2 -39.979 -35.382 3.446 1.00 0.00 O1 C

ATOM 195 H8R POPCO 2 -40.015 -34.248 3.707 1.00 0.00 O1 H

ATOM 196 H8S POPCO 2 -38.916 -35.576 3.194 1.00 0.00 O1 H

ATOM 197 C29 POPCO 2 -40.846 -35.724 2.256 1.00 0.00 O1 C

ATOM 198 H91 POPCO 2 -41.931 -35.547 2.488 1.00 0.00 O1 H

ATOM 199 C210 POPCO 2 -40.460 -36.209 1.124 1.00 0.00 O1 C

ATOM 200 H101 POPCO 2 -41.295 -36.345 0.469 1.00 0.00 O1 H

ATOM 201 C211 POPCO 2 -39.042 -36.542 0.748 1.00 0.00 O1 C

ATOM 202 H11R POPCO 2 -38.333 -36.110 1.468 1.00 0.00 O1 H

ATOM 203 H11S POPCO 2 -38.926 -37.651 0.708 1.00 0.00 O1 H

ATOM 204 C212 POPCO 2 -38.790 -36.033 -0.711 1.00 0.00 O1 C

ATOM 205 H12R POPCO 2 -38.912 -34.919 -0.776 1.00 0.00 O1 H

ATOM 206 H12S POPCO 2 -37.715 -36.242 -0.895 1.00 0.00 O1 H

ATOM 207 C213 POPCO 2 -39.626 -36.571 -1.875 1.00 0.00 O1 C

ATOM 208 H13R POPCO 2 -39.367 -37.699 -1.873 1.00 0.00 O1 H

ATOM 209 H13S POPCO 2 -40.699 -36.358 -1.770 1.00 0.00 O1 H

ATOM 210 C214 POPCO 2 -39.075 -35.977 -3.202 1.00 0.00 O1 C

ATOM 211 H14R POPCO 2 -39.391 -34.852 -3.239 1.00 0.00 O1 H

ATOM 212 H14S POPCO 2 -37.958 -35.811 -3.130 1.00 0.00 O1 H

ATOM 213 C215 POPCO 2 -39.465 -36.717 -4.491 1.00 0.00 O1 C

ATOM 214 H15R POPCO 2 -39.169 -37.825 -4.476 1.00 0.00 O1 H

ATOM 215 H15S POPCO 2 -40.533 -36.589 -4.739 1.00 0.00 O1 H

ATOM 216 C216 POPCO 2 -38.691 -36.210 -5.770 1.00 0.00 O1 C

ATOM 217 H16R POPCO 2 -38.886 -35.127 -5.948 1.00 0.00 O1 H

ATOM 218 H16S POPCO 2 -37.631 -36.423 -5.608 1.00 0.00 O1 H

ATOM 219 C217 POPCO 2 -39.148 -37.005 -6.989 1.00 0.00 O1 C

ATOM 220 H17R POPCO 2 -39.119 -38.088 -6.695 1.00 0.00 O1 H

ATOM 221 H17S POPCO 2 -40.207 -36.704 -7.089 1.00 0.00 O1 H

ATOM 222 C218 POPCO 2 -38.494 -36.733 -8.366 1.00 0.00 O1 C

ATOM 223 H18R POPCO 2 -38.899 -37.461 -9.113 1.00 0.00 O1 H

ATOM 224 H18S POPCO 2 -38.700 -35.696 -8.739 1.00 0.00 O1 H

ATOM 225 H18T POPCO 2 -37.479 -37.065 -8.403 1.00 0.00 O1 H

ATOM 226 C33 POPCO 2 -38.356 -31.320 13.687 1.00 0.00 O1 C

ATOM 227 H3X POPCO 2 -39.365 -30.946 13.405 1.00 0.00 O1 H

ATOM 228 H3Y POPCO 2 -37.566 -30.619 13.557 1.00 0.00 O1 H

ATOM 229 C34 POPCO 2 -38.092 -32.350 12.566 1.00 0.00 O1 C

ATOM 230 H4X POPCO 2 -37.255 -33.026 12.709 1.00 0.00 O1 H

ATOM 231 H4Y POPCO 2 -38.945 -32.983 12.440 1.00 0.00 O1 H

ATOM 232 C35 POPCO 2 -37.940 -31.633 11.186 1.00 0.00 O1 C

ATOM 233 H5X POPCO 2 -38.752 -30.890 10.948 1.00 0.00 O1 H

ATOM 234 H5Y POPCO 2 -37.030 -31.031 11.153 1.00 0.00 O1 H

ATOM 235 C36 POPCO 2 -37.889 -32.620 9.985 1.00 0.00 O1 C

ATOM 236 H6X POPCO 2 -37.289 -33.475 10.373 1.00 0.00 O1 H

ATOM 237 H6Y POPCO 2 -38.919 -32.990 9.758 1.00 0.00 O1 H

ATOM 238 C37 POPCO 2 -37.312 -32.001 8.690 1.00 0.00 O1 C

ATOM 239 H7X POPCO 2 -37.828 -31.040 8.543 1.00 0.00 O1 H

ATOM 240 H7Y POPCO 2 -36.231 -31.913 8.810 1.00 0.00 O1 H

ATOM 241 C38 POPCO 2 -37.472 -32.866 7.478 1.00 0.00 O1 C

ATOM 242 H8X POPCO 2 -36.914 -33.892 7.554 1.00 0.00 O1 H

ATOM 243 H8Y POPCO 2 -38.514 -33.211 7.318 1.00 0.00 O1 H

ATOM 244 C39 POPCO 2 -36.857 -32.218 6.164 1.00 0.00 O1 C

ATOM 245 H9X POPCO 2 -37.364 -31.249 5.986 1.00 0.00 O1 H

ATOM 246 H9Y POPCO 2 -35.798 -31.955 6.163 1.00 0.00 O1 H

ATOM 247 C310 POPCO 2 -37.238 -33.012 4.869 1.00 0.00 O1 C

ATOM 248 H10X POPCO 2 -36.458 -33.839 4.823 1.00 0.00 O1 H

ATOM 249 H10Y POPCO 2 -38.286 -33.292 4.864 1.00 0.00 O1 H

ATOM 250 C311 POPCO 2 -36.905 -32.175 3.643 1.00 0.00 O1 C

ATOM 251 H11X POPCO 2 -37.554 -31.283 3.508 1.00 0.00 O1 H

ATOM 252 H11Y POPCO 2 -35.740 -31.915 3.624 1.00 0.00 O1 H

ATOM 253 C312 POPCO 2 -37.244 -32.947 2.278 1.00 0.00 O1 C

ATOM 254 H12X POPCO 2 -36.972 -33.974 2.372 1.00 0.00 O1 H

ATOM 255 H12Y POPCO 2 -38.331 -32.926 2.124 1.00 0.00 O1 H

ATOM 256 C313 POPCO 2 -36.559 -32.334 1.061 1.00 0.00 O1 C

ATOM 257 H13X POPCO 2 -36.933 -31.260 1.071 1.00 0.00 O1 H

ATOM 258 H13Y POPCO 2 -35.469 -32.389 1.246 1.00 0.00 O1 H

ATOM 259 C314 POPCO 2 -36.951 -33.035 -0.215 1.00 0.00 O1 C

ATOM 260 H14X POPCO 2 -36.778 -34.128 -0.115 1.00 0.00 O1 H

ATOM 261 H14Y POPCO 2 -37.976 -32.910 -0.638 1.00 0.00 O1 H

ATOM 262 C315 POPCO 2 -36.070 -32.699 -1.451 1.00 0.00 O1 C

ATOM 263 H15X POPCO 2 -36.214 -31.617 -1.639 1.00 0.00 O1 H

ATOM 264 H15Y POPCO 2 -34.970 -32.700 -1.168 1.00 0.00 O1 H

ATOM 265 C316 POPCO 2 -36.324 -33.569 -2.727 1.00 0.00 O1 C

ATOM 266 H16X POPCO 2 -37.300 -33.438 -3.214 1.00 0.00 O1 H

ATOM 267 H16Y POPCO 2 -35.590 -33.250 -3.483 1.00 0.00 O1 H

ATOM 268 H16Z POPCO 2 -36.241 -34.640 -2.410 1.00 0.00 O1 H

ATOM 269 N POPCO 3 -48.668 -25.780 20.897 1.00 0.00 O1 N

ATOM 270 C12 POPCO 3 -48.865 -24.581 20.061 1.00 0.00 O1 C

ATOM 271 H12A POPCO 3 -49.286 -23.721 20.616 1.00 0.00 O1 H

ATOM 272 H12B POPCO 3 -49.722 -24.812 19.422 1.00 0.00 O1 H

ATOM 273 C13 POPCO 3 -47.961 -25.358 22.143 1.00 0.00 O1 C

ATOM 274 H13A POPCO 3 -48.448 -24.495 22.505 1.00 0.00 O1 H

ATOM 275 H13B POPCO 3 -46.971 -25.140 21.834 1.00 0.00 O1 H

ATOM 276 H13C POPCO 3 -47.877 -26.135 22.894 1.00 0.00 O1 H

ATOM 277 C14 POPCO 3 -50.035 -26.335 21.376 1.00 0.00 O1 C

ATOM 278 H14A POPCO 3 -50.552 -26.679 20.512 1.00 0.00 O1 H

ATOM 279 H14B POPCO 3 -50.606 -25.538 21.885 1.00 0.00 O1 H

ATOM 280 H14C POPCO 3 -49.894 -27.073 22.062 1.00 0.00 O1 H

ATOM 281 C15 POPCO 3 -47.835 -26.889 20.287 1.00 0.00 O1 C

ATOM 282 H15A POPCO 3 -48.265 -27.243 19.315 1.00 0.00 O1 H

ATOM 283 H15B POPCO 3 -46.831 -26.479 20.040 1.00 0.00 O1 H

ATOM 284 H15C POPCO 3 -47.479 -27.735 20.928 1.00 0.00 O1 H

ATOM 285 C11 POPCO 3 -47.639 -24.129 19.245 1.00 0.00 O1 C

ATOM 286 H11A POPCO 3 -47.385 -24.737 18.379 1.00 0.00 O1 H

ATOM 287 H11B POPCO 3 -47.942 -23.099 18.860 1.00 0.00 O1 H

ATOM 288 P POPCO 3 -45.023 -24.426 20.063 1.00 0.00 O1 P

ATOM 289 O13 POPCO 3 -45.176 -25.901 19.972 1.00 0.00 O1 O

ATOM 290 O14 POPCO 3 -44.221 -23.850 21.192 1.00 0.00 O1 O

ATOM 291 O12 POPCO 3 -46.521 -23.772 20.032 1.00 0.00 O1 O

ATOM 292 O11 POPCO 3 -44.295 -24.040 18.777 1.00 0.00 O1 O

ATOM 293 C1 POPCO 3 -44.856 -24.404 17.473 1.00 0.00 O1 C

ATOM 294 HA POPCO 3 -45.200 -25.491 17.475 1.00 0.00 O1 H

ATOM 295 HB POPCO 3 -45.643 -23.689 17.123 1.00 0.00 O1 H

ATOM 296 C2 POPCO 3 -43.730 -24.407 16.357 1.00 0.00 O1 C

ATOM 297 HS POPCO 3 -43.248 -23.361 16.335 1.00 0.00 O1 H

ATOM 298 O21 POPCO 3 -44.300 -24.677 15.049 1.00 0.00 O1 O

ATOM 299 C21 POPCO 3 -43.709 -24.104 13.950 1.00 0.00 O1 C

ATOM 300 O22 POPCO 3 -42.732 -23.420 13.979 1.00 0.00 O1 O

ATOM 301 C22 POPCO 3 -44.507 -24.420 12.698 1.00 0.00 O1 C

ATOM 302 H2R POPCO 3 -45.352 -25.080 12.982 1.00 0.00 O1 H

ATOM 303 H2S POPCO 3 -44.887 -23.423 12.348 1.00 0.00 O1 H

ATOM 304 C3 POPCO 3 -42.500 -25.306 16.634 1.00 0.00 O1 C

ATOM 305 HX POPCO 3 -41.744 -25.024 15.835 1.00 0.00 O1 H

ATOM 306 HY POPCO 3 -42.123 -25.036 17.658 1.00 0.00 O1 H

ATOM 307 O31 POPCO 3 -42.863 -26.704 16.591 1.00 0.00 O1 O

ATOM 308 C31 POPCO 3 -42.485 -27.538 17.511 1.00 0.00 O1 C

ATOM 309 O32 POPCO 3 -41.459 -27.327 18.208 1.00 0.00 O1 O

ATOM 310 C32 POPCO 3 -43.227 -28.916 17.433 1.00 0.00 O1 C

ATOM 311 H2X POPCO 3 -42.959 -29.510 18.275 1.00 0.00 O1 H

ATOM 312 H2Y POPCO 3 -44.313 -28.664 17.593 1.00 0.00 O1 H

ATOM 313 C23 POPCO 3 -43.667 -25.127 11.618 1.00 0.00 O1 C

ATOM 314 H3R POPCO 3 -42.846 -24.390 11.271 1.00 0.00 O1 H

ATOM 315 H3S POPCO 3 -43.174 -26.048 11.926 1.00 0.00 O1 H

ATOM 316 C24 POPCO 3 -44.567 -25.536 10.430 1.00 0.00 O1 C

ATOM 317 H4R POPCO 3 -43.927 -26.318 9.942 1.00 0.00 O1 H

ATOM 318 H4S POPCO 3 -45.562 -26.018 10.749 1.00 0.00 O1 H

ATOM 319 C25 POPCO 3 -45.029 -24.384 9.594 1.00 0.00 O1 C

ATOM 320 H5R POPCO 3 -45.847 -23.921 10.035 1.00 0.00 O1 H

ATOM 321 H5S POPCO 3 -44.203 -23.715 9.522 1.00 0.00 O1 H

ATOM 322 C26 POPCO 3 -45.427 -24.912 8.242 1.00 0.00 O1 C

ATOM 323 H6R POPCO 3 -44.563 -25.377 7.821 1.00 0.00 O1 H

ATOM 324 H6S POPCO 3 -46.262 -25.656 8.260 1.00 0.00 O1 H

ATOM 325 C27 POPCO 3 -45.701 -23.794 7.171 1.00 0.00 O1 C

ATOM 326 H7R POPCO 3 -46.702 -23.289 7.429 1.00 0.00 O1 H

ATOM 327 H7S POPCO 3 -44.873 -23.058 7.304 1.00 0.00 O1 H

ATOM 328 C28 POPCO 3 -45.810 -24.460 5.796 1.00 0.00 O1 C

ATOM 329 H8R POPCO 3 -44.916 -25.028 5.542 1.00 0.00 O1 H

ATOM 330 H8S POPCO 3 -46.609 -25.236 5.854 1.00 0.00 O1 H

ATOM 331 C29 POPCO 3 -45.847 -23.436 4.628 1.00 0.00 O1 C

ATOM 332 H91 POPCO 3 -45.250 -22.548 4.796 1.00 0.00 O1 H

ATOM 333 C210 POPCO 3 -46.434 -23.590 3.407 1.00 0.00 O1 C

ATOM 334 H101 POPCO 3 -46.269 -22.794 2.628 1.00 0.00 O1 H

ATOM 335 C211 POPCO 3 -47.244 -24.745 2.922 1.00 0.00 O1 C

ATOM 336 H11R POPCO 3 -47.369 -25.433 3.762 1.00 0.00 O1 H

ATOM 337 H11S POPCO 3 -48.287 -24.317 2.776 1.00 0.00 O1 H

ATOM 338 C212 POPCO 3 -46.889 -25.392 1.568 1.00 0.00 O1 C

ATOM 339 H12R POPCO 3 -45.900 -25.873 1.688 1.00 0.00 O1 H

ATOM 340 H12S POPCO 3 -47.669 -26.232 1.625 1.00 0.00 O1 H

ATOM 341 C213 POPCO 3 -47.160 -24.663 0.280 1.00 0.00 O1 C

ATOM 342 H13R POPCO 3 -48.201 -24.225 0.339 1.00 0.00 O1 H

ATOM 343 H13S POPCO 3 -46.542 -23.716 0.292 1.00 0.00 O1 H

ATOM 344 C214 POPCO 3 -46.851 -25.481 -0.936 1.00 0.00 O1 C

ATOM 345 H14R POPCO 3 -45.798 -25.774 -0.836 1.00 0.00 O1 H

ATOM 346 H14S POPCO 3 -47.441 -26.417 -1.010 1.00 0.00 O1 H

ATOM 347 C215 POPCO 3 -47.043 -24.629 -2.236 1.00 0.00 O1 C

ATOM 348 H15R POPCO 3 -48.043 -24.077 -2.214 1.00 0.00 O1 H

ATOM 349 H15S POPCO 3 -46.314 -23.755 -2.157 1.00 0.00 O1 H

ATOM 350 C216 POPCO 3 -46.839 -25.555 -3.447 1.00 0.00 O1 C

ATOM 351 H16R POPCO 3 -45.877 -26.171 -3.224 1.00 0.00 O1 H

ATOM 352 H16S POPCO 3 -47.666 -26.231 -3.387 1.00 0.00 O1 H

ATOM 353 C217 POPCO 3 -46.715 -24.842 -4.846 1.00 0.00 O1 C

ATOM 354 H17R POPCO 3 -47.707 -24.595 -5.148 1.00 0.00 O1 H

ATOM 355 H17S POPCO 3 -46.153 -23.888 -4.826 1.00 0.00 O1 H

ATOM 356 C218 POPCO 3 -46.126 -25.754 -5.824 1.00 0.00 O1 C

ATOM 357 H18R POPCO 3 -46.119 -25.216 -6.810 1.00 0.00 O1 H

ATOM 358 H18S POPCO 3 -45.084 -26.039 -5.557 1.00 0.00 O1 H

ATOM 359 H18T POPCO 3 -46.721 -26.654 -5.877 1.00 0.00 O1 H

ATOM 360 C33 POPCO 3 -43.066 -29.731 16.124 1.00 0.00 O1 C

ATOM 361 H3X POPCO 3 -41.998 -30.138 16.153 1.00 0.00 O1 H

ATOM 362 H3Y POPCO 3 -43.689 -30.668 16.169 1.00 0.00 O1 H

ATOM 363 C34 POPCO 3 -43.478 -29.046 14.829 1.00 0.00 O1 C

ATOM 364 H4X POPCO 3 -44.445 -28.549 15.116 1.00 0.00 O1 H

ATOM 365 H4Y POPCO 3 -42.747 -28.219 14.502 1.00 0.00 O1 H

ATOM 366 C35 POPCO 3 -43.651 -29.980 13.645 1.00 0.00 O1 C

ATOM 367 H5X POPCO 3 -42.771 -30.666 13.608 1.00 0.00 O1 H

ATOM 368 H5Y POPCO 3 -44.524 -30.624 13.896 1.00 0.00 O1 H

ATOM 369 C36 POPCO 3 -43.924 -29.255 12.314 1.00 0.00 O1 C

ATOM 370 H6X POPCO 3 -44.946 -28.767 12.512 1.00 0.00 O1 H

ATOM 371 H6Y POPCO 3 -43.187 -28.457 12.118 1.00 0.00 O1 H

ATOM 372 C37 POPCO 3 -44.058 -30.202 11.057 1.00 0.00 O1 C

ATOM 373 H7X POPCO 3 -43.097 -30.624 10.645 1.00 0.00 O1 H

ATOM 374 H7Y POPCO 3 -44.663 -31.027 11.481 1.00 0.00 O1 H

ATOM 375 C38 POPCO 3 -44.773 -29.494 9.869 1.00 0.00 O1 C

ATOM 376 H8X POPCO 3 -45.878 -29.541 10.061 1.00 0.00 O1 H

ATOM 377 H8Y POPCO 3 -44.488 -28.429 9.788 1.00 0.00 O1 H

ATOM 378 C39 POPCO 3 -44.794 -30.244 8.503 1.00 0.00 O1 C

ATOM 379 H9X POPCO 3 -43.768 -30.508 8.238 1.00 0.00 O1 H

ATOM 380 H9Y POPCO 3 -45.337 -31.149 8.742 1.00 0.00 O1 H

ATOM 381 C310 POPCO 3 -45.574 -29.481 7.427 1.00 0.00 O1 C

ATOM 382 H10X POPCO 3 -46.540 -29.163 7.902 1.00 0.00 O1 H

ATOM 383 H10Y POPCO 3 -44.939 -28.575 7.100 1.00 0.00 O1 H

ATOM 384 C311 POPCO 3 -45.830 -30.311 6.144 1.00 0.00 O1 C

ATOM 385 H11X POPCO 3 -44.805 -30.646 5.842 1.00 0.00 O1 H

ATOM 386 H11Y POPCO 3 -46.459 -31.132 6.395 1.00 0.00 O1 H

ATOM 387 C312 POPCO 3 -46.322 -29.516 4.972 1.00 0.00 O1 C

ATOM 388 H12X POPCO 3 -47.267 -29.012 5.058 1.00 0.00 O1 H

ATOM 389 H12Y POPCO 3 -45.581 -28.736 4.793 1.00 0.00 O1 H

ATOM 390 C313 POPCO 3 -46.332 -30.379 3.717 1.00 0.00 O1 C

ATOM 391 H13X POPCO 3 -45.487 -31.006 3.632 1.00 0.00 O1 H

ATOM 392 H13Y POPCO 3 -47.225 -30.986 3.675 1.00 0.00 O1 H

ATOM 393 C314 POPCO 3 -46.502 -29.396 2.493 1.00 0.00 O1 C

ATOM 394 H14X POPCO 3 -47.492 -28.867 2.593 1.00 0.00 O1 H

ATOM 395 H14Y POPCO 3 -45.707 -28.693 2.422 1.00 0.00 O1 H

ATOM 396 C315 POPCO 3 -46.437 -30.013 1.092 1.00 0.00 O1 C

ATOM 397 H15X POPCO 3 -45.520 -30.507 1.022 1.00 0.00 O1 H

ATOM 398 H15Y POPCO 3 -47.211 -30.810 1.087 1.00 0.00 O1 H

ATOM 399 C316 POPCO 3 -46.518 -28.931 -0.017 1.00 0.00 O1 C

ATOM 400 H16X POPCO 3 -45.704 -28.116 0.097 1.00 0.00 O1 H

ATOM 401 H16Y POPCO 3 -46.221 -29.441 -0.912 1.00 0.00 O1 H

ATOM 402 H16Z POPCO 3 -47.570 -28.565 -0.040 1.00 0.00 O1 H

ATOM 403 N POPCO 4 -35.532 -19.191 22.551 1.00 0.00 O1 N

ATOM 404 C12 POPCO 4 -36.785 -18.281 22.306 1.00 0.00 O1 C

ATOM 405 H12A POPCO 4 -37.003 -17.879 23.251 1.00 0.00 O1 H

ATOM 406 H12B POPCO 4 -37.643 -19.005 22.074 1.00 0.00 O1 H

ATOM 407 C13 POPCO 4 -34.350 -18.363 22.981 1.00 0.00 O1 C

ATOM 408 H13A POPCO 4 -34.570 -17.707 23.815 1.00 0.00 O1 H

ATOM 409 H13B POPCO 4 -34.130 -17.722 22.150 1.00 0.00 O1 H

ATOM 410 H13C POPCO 4 -33.494 -19.014 23.135 1.00 0.00 O1 H

ATOM 411 C14 POPCO 4 -35.955 -20.137 23.607 1.00 0.00 O1 C

ATOM 412 H14A POPCO 4 -36.713 -20.819 23.269 1.00 0.00 O1 H

ATOM 413 H14B POPCO 4 -36.368 -19.649 24.499 1.00 0.00 O1 H

ATOM 414 H14C POPCO 4 -35.174 -20.856 23.821 1.00 0.00 O1 H

ATOM 415 C15 POPCO 4 -35.184 -19.962 21.335 1.00 0.00 O1 C

ATOM 416 H15A POPCO 4 -36.069 -20.610 21.131 1.00 0.00 O1 H

ATOM 417 H15B POPCO 4 -35.034 -19.373 20.476 1.00 0.00 O1 H

ATOM 418 H15C POPCO 4 -34.344 -20.614 21.410 1.00 0.00 O1 H

ATOM 419 C11 POPCO 4 -36.836 -17.363 21.102 1.00 0.00 O1 C

ATOM 420 H11A POPCO 4 -37.054 -17.955 20.129 1.00 0.00 O1 H

ATOM 421 H11B POPCO 4 -37.911 -16.870 21.055 1.00 0.00 O1 H

ATOM 422 P POPCO 4 -34.751 -16.375 19.952 1.00 0.00 O1 P

ATOM 423 O13 POPCO 4 -33.412 -16.189 20.544 1.00 0.00 O1 O

ATOM 424 O14 POPCO 4 -35.182 -15.339 18.998 1.00 0.00 O1 O

ATOM 425 O12 POPCO 4 -35.822 -16.376 21.066 1.00 0.00 O1 O

ATOM 426 O11 POPCO 4 -34.818 -17.775 19.189 1.00 0.00 O1 O

ATOM 427 C1 POPCO 4 -35.450 -17.696 17.930 1.00 0.00 O1 C

ATOM 428 HA POPCO 4 -36.501 -17.399 18.009 1.00 0.00 O1 H

ATOM 429 HB POPCO 4 -34.930 -16.898 17.304 1.00 0.00 O1 H

ATOM 430 C2 POPCO 4 -35.457 -19.011 17.159 1.00 0.00 O1 C

ATOM 431 HS POPCO 4 -34.429 -19.460 17.262 1.00 0.00 O1 H

ATOM 432 O21 POPCO 4 -35.859 -18.671 15.880 1.00 0.00 O1 O

ATOM 433 C21 POPCO 4 -35.618 -19.660 14.991 1.00 0.00 O1 C

ATOM 434 O22 POPCO 4 -34.940 -20.666 15.204 1.00 0.00 O1 O

ATOM 435 C22 POPCO 4 -36.322 -19.365 13.663 1.00 0.00 O1 C

ATOM 436 H2R POPCO 4 -37.420 -19.331 13.801 1.00 0.00 O1 H

ATOM 437 H2S POPCO 4 -36.022 -18.417 13.317 1.00 0.00 O1 H

ATOM 438 C3 POPCO 4 -36.426 -20.043 17.772 1.00 0.00 O1 C

ATOM 439 HX POPCO 4 -36.490 -20.978 17.235 1.00 0.00 O1 H

ATOM 440 HY POPCO 4 -35.919 -20.386 18.699 1.00 0.00 O1 H

ATOM 441 O31 POPCO 4 -37.722 -19.453 18.019 1.00 0.00 O1 O

ATOM 442 C31 POPCO 4 -38.666 -20.151 18.681 1.00 0.00 O1 C

ATOM 443 O32 POPCO 4 -38.626 -21.271 19.039 1.00 0.00 O1 O

ATOM 444 C32 POPCO 4 -39.844 -19.273 18.941 1.00 0.00 O1 C

ATOM 445 H2X POPCO 4 -40.455 -19.693 19.844 1.00 0.00 O1 H

ATOM 446 H2Y POPCO 4 -39.452 -18.246 19.204 1.00 0.00 O1 H

ATOM 447 C23 POPCO 4 -36.130 -20.472 12.514 1.00 0.00 O1 C

ATOM 448 H3R POPCO 4 -35.036 -20.564 12.352 1.00 0.00 O1 H

ATOM 449 H3S POPCO 4 -36.520 -21.503 12.838 1.00 0.00 O1 H

ATOM 450 C24 POPCO 4 -36.898 -19.959 11.247 1.00 0.00 O1 C

ATOM 451 H4R POPCO 4 -37.970 -19.976 11.379 1.00 0.00 O1 H

ATOM 452 H4S POPCO 4 -36.595 -18.992 10.796 1.00 0.00 O1 H

ATOM 453 C25 POPCO 4 -36.658 -20.910 10.072 1.00 0.00 O1 C

ATOM 454 H5R POPCO 4 -35.608 -20.712 9.794 1.00 0.00 O1 H

ATOM 455 H5S POPCO 4 -36.802 -21.994 10.294 1.00 0.00 O1 H

ATOM 456 C26 POPCO 4 -37.720 -20.611 8.992 1.00 0.00 O1 C

ATOM 457 H6R POPCO 4 -38.634 -20.969 9.453 1.00 0.00 O1 H

ATOM 458 H6S POPCO 4 -37.828 -19.501 8.880 1.00 0.00 O1 H

ATOM 459 C27 POPCO 4 -37.412 -21.199 7.658 1.00 0.00 O1 C

ATOM 460 H7R POPCO 4 -36.441 -20.787 7.303 1.00 0.00 O1 H

ATOM 461 H7S POPCO 4 -37.239 -22.227 7.881 1.00 0.00 O1 H

ATOM 462 C28 POPCO 4 -38.538 -20.748 6.678 1.00 0.00 O1 C

ATOM 463 H8R POPCO 4 -39.541 -21.141 7.034 1.00 0.00 O1 H

ATOM 464 H8S POPCO 4 -38.562 -19.659 6.781 1.00 0.00 O1 H

ATOM 465 C29 POPCO 4 -38.217 -21.290 5.303 1.00 0.00 O1 C

ATOM 466 H91 POPCO 4 -38.198 -22.399 5.234 1.00 0.00 O1 H

ATOM 467 C210 POPCO 4 -37.633 -20.648 4.217 1.00 0.00 O1 C

ATOM 468 H101 POPCO 4 -37.442 -21.193 3.355 1.00 0.00 O1 H

ATOM 469 C211 POPCO 4 -37.413 -19.161 4.064 1.00 0.00 O1 C

ATOM 470 H11R POPCO 4 -37.864 -18.431 4.817 1.00 0.00 O1 H

ATOM 471 H11S POPCO 4 -36.329 -18.983 4.153 1.00 0.00 O1 H

ATOM 472 C212 POPCO 4 -37.890 -18.616 2.712 1.00 0.00 O1 C

ATOM 473 H12R POPCO 4 -39.002 -18.855 2.596 1.00 0.00 O1 H

ATOM 474 H12S POPCO 4 -37.676 -17.484 2.683 1.00 0.00 O1 H

ATOM 475 C213 POPCO 4 -37.121 -19.233 1.459 1.00 0.00 O1 C

ATOM 476 H13R POPCO 4 -36.050 -18.948 1.400 1.00 0.00 O1 H

ATOM 477 H13S POPCO 4 -37.291 -20.352 1.337 1.00 0.00 O1 H

ATOM 478 C214 POPCO 4 -37.823 -18.682 0.173 1.00 0.00 O1 C

ATOM 479 H14R POPCO 4 -38.788 -19.212 0.048 1.00 0.00 O1 H

ATOM 480 H14S POPCO 4 -38.197 -17.721 0.493 1.00 0.00 O1 H

ATOM 481 C215 POPCO 4 -37.040 -18.758 -1.090 1.00 0.00 O1 C

ATOM 482 H15R POPCO 4 -36.147 -18.112 -1.036 1.00 0.00 O1 H

ATOM 483 H15S POPCO 4 -36.620 -19.771 -1.271 1.00 0.00 O1 H

ATOM 484 C216 POPCO 4 -37.999 -18.203 -2.201 1.00 0.00 O1 C

ATOM 485 H16R POPCO 4 -38.864 -18.699 -2.120 1.00 0.00 O1 H

ATOM 486 H16S POPCO 4 -38.250 -17.104 -2.011 1.00 0.00 O1 H

ATOM 487 C217 POPCO 4 -37.445 -18.278 -3.627 1.00 0.00 O1 C

ATOM 488 H17R POPCO 4 -36.362 -17.877 -3.570 1.00 0.00 O1 H

ATOM 489 H17S POPCO 4 -37.323 -19.322 -3.889 1.00 0.00 O1 H

ATOM 490 C218 POPCO 4 -38.211 -17.474 -4.653 1.00 0.00 O1 C

ATOM 491 H18R POPCO 4 -38.515 -18.175 -5.516 1.00 0.00 O1 H

ATOM 492 H18S POPCO 4 -39.140 -16.982 -4.271 1.00 0.00 O1 H

ATOM 493 H18T POPCO 4 -37.574 -16.650 -5.195 1.00 0.00 O1 H

ATOM 494 C33 POPCO 4 -40.775 -19.114 17.746 1.00 0.00 O1 C

ATOM 495 H3X POPCO 4 -41.022 -20.161 17.484 1.00 0.00 O1 H

ATOM 496 H3Y POPCO 4 -41.754 -18.580 17.912 1.00 0.00 O1 H

ATOM 497 C34 POPCO 4 -40.222 -18.418 16.452 1.00 0.00 O1 C

ATOM 498 H4X POPCO 4 -40.231 -17.328 16.729 1.00 0.00 O1 H

ATOM 499 H4Y POPCO 4 -39.177 -18.768 16.266 1.00 0.00 O1 H

ATOM 500 C35 POPCO 4 -41.077 -18.531 15.224 1.00 0.00 O1 C

ATOM 501 H5X POPCO 4 -41.411 -19.618 15.045 1.00 0.00 O1 H

ATOM 502 H5Y POPCO 4 -41.971 -17.788 15.462 1.00 0.00 O1 H

ATOM 503 C36 POPCO 4 -40.403 -17.972 14.053 1.00 0.00 O1 C

ATOM 504 H6X POPCO 4 -40.240 -16.864 14.252 1.00 0.00 O1 H

ATOM 505 H6Y POPCO 4 -39.388 -18.202 13.807 1.00 0.00 O1 H

ATOM 506 C37 POPCO 4 -41.182 -18.056 12.819 1.00 0.00 O1 C

ATOM 507 H7X POPCO 4 -41.461 -19.077 12.497 1.00 0.00 O1 H

ATOM 508 H7Y POPCO 4 -42.048 -17.448 12.846 1.00 0.00 O1 H

ATOM 509 C38 POPCO 4 -40.265 -17.447 11.747 1.00 0.00 O1 C

ATOM 510 H8X POPCO 4 -39.939 -16.432 12.027 1.00 0.00 O1 H

ATOM 511 H8Y POPCO 4 -39.227 -17.828 11.724 1.00 0.00 O1 H

ATOM 512 C39 POPCO 4 -40.955 -17.453 10.341 1.00 0.00 O1 C

ATOM 513 H9X POPCO 4 -41.525 -18.379 10.120 1.00 0.00 O1 H

ATOM 514 H9Y POPCO 4 -41.760 -16.652 10.498 1.00 0.00 O1 H

ATOM 515 C310 POPCO 4 -40.036 -17.065 9.177 1.00 0.00 O1 C

ATOM 516 H10X POPCO 4 -39.322 -16.254 9.414 1.00 0.00 O1 H

ATOM 517 H10Y POPCO 4 -39.434 -18.045 9.178 1.00 0.00 O1 H

ATOM 518 C311 POPCO 4 -40.773 -16.896 7.793 1.00 0.00 O1 C

ATOM 519 H11X POPCO 4 -41.406 -17.765 7.617 1.00 0.00 O1 H

ATOM 520 H11Y POPCO 4 -41.487 -16.120 7.977 1.00 0.00 O1 H

ATOM 521 C312 POPCO 4 -39.744 -16.518 6.690 1.00 0.00 O1 C

ATOM 522 H12X POPCO 4 -38.981 -15.855 7.187 1.00 0.00 O1 H

ATOM 523 H12Y POPCO 4 -39.178 -17.413 6.279 1.00 0.00 O1 H

ATOM 524 C313 POPCO 4 -40.371 -15.705 5.533 1.00 0.00 O1 C

ATOM 525 H13X POPCO 4 -41.271 -16.367 5.347 1.00 0.00 O1 H

ATOM 526 H13Y POPCO 4 -40.564 -14.733 5.897 1.00 0.00 O1 H

ATOM 527 C314 POPCO 4 -39.474 -15.618 4.225 1.00 0.00 O1 C

ATOM 528 H14X POPCO 4 -38.665 -14.879 4.398 1.00 0.00 O1 H

ATOM 529 H14Y POPCO 4 -39.080 -16.603 3.952 1.00 0.00 O1 H

ATOM 530 C315 POPCO 4 -40.370 -15.221 2.996 1.00 0.00 O1 C

ATOM 531 H15X POPCO 4 -41.330 -15.752 3.099 1.00 0.00 O1 H

ATOM 532 H15Y POPCO 4 -40.694 -14.148 3.019 1.00 0.00 O1 H

ATOM 533 C316 POPCO 4 -39.787 -15.573 1.664 1.00 0.00 O1 C

ATOM 534 H16X POPCO 4 -39.553 -16.661 1.544 1.00 0.00 O1 H

ATOM 535 H16Y POPCO 4 -40.534 -15.211 0.948 1.00 0.00 O1 H

ATOM 536 H16Z POPCO 4 -38.782 -15.094 1.533 1.00 0.00 O1 H

ATOM 537 N POPCO 5 -44.679 -8.366 19.564 1.00 0.00 O1 N

ATOM 538 C12 POPCO 5 -45.676 -9.441 19.039 1.00 0.00 O1 C

ATOM 539 H12A POPCO 5 -46.166 -9.015 18.135 1.00 0.00 O1 H

ATOM 540 H12B POPCO 5 -46.452 -9.568 19.735 1.00 0.00 O1 H

ATOM 541 C13 POPCO 5 -43.557 -7.959 18.594 1.00 0.00 O1 C

ATOM 542 H13A POPCO 5 -44.112 -7.406 17.817 1.00 0.00 O1 H

ATOM 543 H13B POPCO 5 -42.974 -8.830 18.357 1.00 0.00 O1 H

ATOM 544 H13C POPCO 5 -42.813 -7.262 19.000 1.00 0.00 O1 H

ATOM 545 C14 POPCO 5 -45.434 -7.147 20.107 1.00 0.00 O1 C

ATOM 546 H14A POPCO 5 -46.251 -7.357 20.815 1.00 0.00 O1 H

ATOM 547 H14B POPCO 5 -45.700 -6.478 19.284 1.00 0.00 O1 H

ATOM 548 H14C POPCO 5 -44.702 -6.711 20.717 1.00 0.00 O1 H

ATOM 549 C15 POPCO 5 -44.033 -8.860 20.804 1.00 0.00 O1 C

ATOM 550 H15A POPCO 5 -44.821 -9.305 21.394 1.00 0.00 O1 H

ATOM 551 H15B POPCO 5 -43.301 -9.666 20.562 1.00 0.00 O1 H

ATOM 552 H15C POPCO 5 -43.527 -7.949 21.146 1.00 0.00 O1 H

ATOM 553 C11 POPCO 5 -45.077 -10.829 18.680 1.00 0.00 O1 C

ATOM 554 H11A POPCO 5 -44.943 -11.315 19.613 1.00 0.00 O1 H

ATOM 555 H11B POPCO 5 -45.844 -11.428 18.196 1.00 0.00 O1 H

ATOM 556 P POPCO 5 -42.656 -11.427 18.172 1.00 0.00 O1 P

ATOM 557 O13 POPCO 5 -42.881 -12.841 18.308 1.00 0.00 O1 O

ATOM 558 O14 POPCO 5 -42.140 -10.723 19.318 1.00 0.00 O1 O

ATOM 559 O12 POPCO 5 -43.968 -10.700 17.756 1.00 0.00 O1 O

ATOM 560 O11 POPCO 5 -41.524 -11.270 17.082 1.00 0.00 O1 O

ATOM 561 C1 POPCO 5 -41.705 -10.833 15.734 1.00 0.00 O1 C

ATOM 562 HA POPCO 5 -42.690 -10.264 15.632 1.00 0.00 O1 H

ATOM 563 HB POPCO 5 -40.842 -10.203 15.413 1.00 0.00 O1 H

ATOM 564 C2 POPCO 5 -41.686 -11.946 14.560 1.00 0.00 O1 C

ATOM 565 HS POPCO 5 -40.620 -12.313 14.576 1.00 0.00 O1 H

ATOM 566 O21 POPCO 5 -41.998 -11.271 13.347 1.00 0.00 O1 O

ATOM 567 C21 POPCO 5 -41.484 -11.764 12.240 1.00 0.00 O1 C

ATOM 568 O22 POPCO 5 -40.663 -12.675 12.158 1.00 0.00 O1 O

ATOM 569 C22 POPCO 5 -42.303 -11.217 10.981 1.00 0.00 O1 C

ATOM 570 H2R POPCO 5 -43.086 -11.936 10.945 1.00 0.00 O1 H

ATOM 571 H2S POPCO 5 -42.648 -10.166 11.191 1.00 0.00 O1 H

ATOM 572 C3 POPCO 5 -42.703 -13.220 14.852 1.00 0.00 O1 C

ATOM 573 HX POPCO 5 -42.374 -14.070 14.147 1.00 0.00 O1 H

ATOM 574 HY POPCO 5 -42.597 -13.655 15.878 1.00 0.00 O1 H

ATOM 575 O31 POPCO 5 -44.082 -12.831 14.685 1.00 0.00 O1 O

ATOM 576 C31 POPCO 5 -44.959 -13.620 15.235 1.00 0.00 O1 C

ATOM 577 O32 POPCO 5 -44.730 -14.735 15.678 1.00 0.00 O1 O

ATOM 578 C32 POPCO 5 -46.343 -12.925 15.140 1.00 0.00 O1 C

ATOM 579 H2X POPCO 5 -47.049 -13.407 15.855 1.00 0.00 O1 H

ATOM 580 H2Y POPCO 5 -46.198 -11.850 15.496 1.00 0.00 O1 H

ATOM 581 C23 POPCO 5 -41.532 -11.263 9.671 1.00 0.00 O1 C

ATOM 582 H3R POPCO 5 -40.833 -10.412 9.561 1.00 0.00 O1 H

ATOM 583 H3S POPCO 5 -40.952 -12.136 9.406 1.00 0.00 O1 H

ATOM 584 C24 POPCO 5 -42.399 -11.265 8.407 1.00 0.00 O1 C

ATOM 585 H4R POPCO 5 -42.791 -12.256 8.208 1.00 0.00 O1 H

ATOM 586 H4S POPCO 5 -43.190 -10.525 8.674 1.00 0.00 O1 H

ATOM 587 C25 POPCO 5 -41.620 -10.877 7.145 1.00 0.00 O1 C

ATOM 588 H5R POPCO 5 -41.093 -9.922 7.378 1.00 0.00 O1 H

ATOM 589 H5S POPCO 5 -40.728 -11.541 6.955 1.00 0.00 O1 H

ATOM 590 C26 POPCO 5 -42.533 -10.817 5.896 1.00 0.00 O1 C

ATOM 591 H6R POPCO 5 -42.854 -11.790 5.615 1.00 0.00 O1 H

ATOM 592 H6S POPCO 5 -43.406 -10.206 6.147 1.00 0.00 O1 H

ATOM 593 C27 POPCO 5 -41.694 -10.254 4.700 1.00 0.00 O1 C

ATOM 594 H7R POPCO 5 -41.292 -9.235 4.818 1.00 0.00 O1 H

ATOM 595 H7S POPCO 5 -40.939 -11.072 4.748 1.00 0.00 O1 H

ATOM 596 C28 POPCO 5 -42.336 -10.409 3.306 1.00 0.00 O1 C

ATOM 597 H8R POPCO 5 -42.497 -11.553 3.107 1.00 0.00 O1 H

ATOM 598 H8S POPCO 5 -43.401 -10.009 3.442 1.00 0.00 O1 H

ATOM 599 C29 POPCO 5 -41.533 -9.753 2.243 1.00 0.00 O1 C

ATOM 600 H91 POPCO 5 -40.375 -9.798 2.319 1.00 0.00 O1 H

ATOM 601 C210 POPCO 5 -42.045 -9.053 1.152 1.00 0.00 O1 C

ATOM 602 H101 POPCO 5 -41.296 -8.583 0.524 1.00 0.00 O1 H

ATOM 603 C211 POPCO 5 -43.461 -8.790 0.683 1.00 0.00 O1 C

ATOM 604 H11R POPCO 5 -44.264 -9.258 1.387 1.00 0.00 O1 H

ATOM 605 H11S POPCO 5 -43.697 -7.729 0.573 1.00 0.00 O1 H

ATOM 606 C212 POPCO 5 -43.788 -9.460 -0.659 1.00 0.00 O1 C

ATOM 607 H12R POPCO 5 -43.857 -10.577 -0.503 1.00 0.00 O1 H

ATOM 608 H12S POPCO 5 -44.812 -9.104 -1.023 1.00 0.00 O1 H

ATOM 609 C213 POPCO 5 -42.775 -9.209 -1.756 1.00 0.00 O1 C

ATOM 610 H13R POPCO 5 -42.654 -8.110 -1.956 1.00 0.00 O1 H

ATOM 611 H13S POPCO 5 -41.752 -9.605 -1.494 1.00 0.00 O1 H

ATOM 612 C214 POPCO 5 -43.140 -10.045 -3.002 1.00 0.00 O1 C

ATOM 613 H14R POPCO 5 -43.295 -11.189 -2.882 1.00 0.00 O1 H

ATOM 614 H14S POPCO 5 -44.051 -9.727 -3.512 1.00 0.00 O1 H

ATOM 615 C215 POPCO 5 -42.041 -9.922 -4.094 1.00 0.00 O1 C

ATOM 616 H15R POPCO 5 -41.831 -8.883 -4.311 1.00 0.00 O1 H

ATOM 617 H15S POPCO 5 -41.139 -10.492 -3.726 1.00 0.00 O1 H

ATOM 618 C216 POPCO 5 -42.323 -10.649 -5.470 1.00 0.00 O1 C

ATOM 619 H16R POPCO 5 -42.790 -11.696 -5.214 1.00 0.00 O1 H

ATOM 620 H16S POPCO 5 -43.172 -10.079 -5.951 1.00 0.00 O1 H

ATOM 621 C217 POPCO 5 -41.101 -10.667 -6.378 1.00 0.00 O1 C

ATOM 622 H17R POPCO 5 -40.584 -9.620 -6.344 1.00 0.00 O1 H

ATOM 623 H17S POPCO 5 -40.429 -11.403 -6.077 1.00 0.00 O1 H

ATOM 624 C218 POPCO 5 -41.359 -10.983 -7.821 1.00 0.00 O1 C

ATOM 625 H18R POPCO 5 -40.449 -10.776 -8.423 1.00 0.00 O1 H

ATOM 626 H18S POPCO 5 -41.626 -12.016 -7.878 1.00 0.00 O1 H

ATOM 627 H18T POPCO 5 -42.188 -10.409 -8.222 1.00 0.00 O1 H

ATOM 628 C33 POPCO 5 -46.964 -12.882 13.769 1.00 0.00 O1 C

ATOM 629 H3X POPCO 5 -46.905 -13.908 13.339 1.00 0.00 O1 H

ATOM 630 H3Y POPCO 5 -48.015 -12.658 13.841 1.00 0.00 O1 H

ATOM 631 C34 POPCO 5 -46.177 -12.041 12.716 1.00 0.00 O1 C

ATOM 632 H4X POPCO 5 -45.897 -11.033 13.074 1.00 0.00 O1 H

ATOM 633 H4Y POPCO 5 -45.274 -12.594 12.432 1.00 0.00 O1 H

ATOM 634 C35 POPCO 5 -47.038 -11.863 11.476 1.00 0.00 O1 C

ATOM 635 H5X POPCO 5 -47.519 -12.820 11.374 1.00 0.00 O1 H

ATOM 636 H5Y POPCO 5 -47.895 -11.267 11.670 1.00 0.00 O1 H

ATOM 637 C36 POPCO 5 -46.463 -11.472 10.060 1.00 0.00 O1 C

ATOM 638 H6X POPCO 5 -46.073 -10.425 10.045 1.00 0.00 O1 H

ATOM 639 H6Y POPCO 5 -45.549 -12.107 9.775 1.00 0.00 O1 H

ATOM 640 C37 POPCO 5 -47.403 -11.553 8.906 1.00 0.00 O1 C

ATOM 641 H7X POPCO 5 -47.909 -12.495 8.868 1.00 0.00 O1 H

ATOM 642 H7Y POPCO 5 -48.207 -10.757 8.885 1.00 0.00 O1 H

ATOM 643 C38 POPCO 5 -46.627 -11.475 7.600 1.00 0.00 O1 C

ATOM 644 H8X POPCO 5 -45.961 -10.625 7.458 1.00 0.00 O1 H

ATOM 645 H8Y POPCO 5 -45.850 -12.242 7.642 1.00 0.00 O1 H

ATOM 646 C39 POPCO 5 -47.447 -11.492 6.292 1.00 0.00 O1 C

ATOM 647 H9X POPCO 5 -47.784 -12.477 6.060 1.00 0.00 O1 H

ATOM 648 H9Y POPCO 5 -48.360 -10.865 6.348 1.00 0.00 O1 H

ATOM 649 C310 POPCO 5 -46.682 -11.066 5.041 1.00 0.00 O1 C

ATOM 650 H10X POPCO 5 -46.352 -9.993 5.125 1.00 0.00 O1 H

ATOM 651 H10Y POPCO 5 -45.828 -11.745 4.961 1.00 0.00 O1 H

ATOM 652 C311 POPCO 5 -47.504 -11.298 3.731 1.00 0.00 O1 C

ATOM 653 H11X POPCO 5 -47.645 -12.436 3.723 1.00 0.00 O1 H

ATOM 654 H11Y POPCO 5 -48.434 -10.825 4.031 1.00 0.00 O1 H

ATOM 655 C312 POPCO 5 -46.990 -10.623 2.461 1.00 0.00 O1 C

ATOM 656 H12X POPCO 5 -47.135 -9.606 2.488 1.00 0.00 O1 H

ATOM 657 H12Y POPCO 5 -45.912 -10.834 2.246 1.00 0.00 O1 H

ATOM 658 C313 POPCO 5 -47.742 -11.222 1.174 1.00 0.00 O1 C

ATOM 659 H13X POPCO 5 -47.650 -12.301 1.057 1.00 0.00 O1 H

ATOM 660 H13Y POPCO 5 -48.779 -11.029 1.478 1.00 0.00 O1 H

ATOM 661 C314 POPCO 5 -47.367 -10.509 -0.098 1.00 0.00 O1 C

ATOM 662 H14X POPCO 5 -47.646 -9.424 -0.112 1.00 0.00 O1 H

ATOM 663 H14Y POPCO 5 -46.217 -10.553 -0.172 1.00 0.00 O1 H

ATOM 664 C315 POPCO 5 -47.890 -11.168 -1.419 1.00 0.00 O1 C

ATOM 665 H15X POPCO 5 -47.763 -12.309 -1.296 1.00 0.00 O1 H

ATOM 666 H15Y POPCO 5 -48.986 -11.063 -1.417 1.00 0.00 O1 H

ATOM 667 C316 POPCO 5 -47.242 -10.566 -2.756 1.00 0.00 O1 C

ATOM 668 H16X POPCO 5 -46.135 -10.759 -2.831 1.00 0.00 O1 H

ATOM 669 H16Y POPCO 5 -47.802 -11.112 -3.597 1.00 0.00 O1 H

ATOM 670 H16Z POPCO 5 -47.451 -9.454 -2.737 1.00 0.00 O1 H

ATOM 671 N POPCO 6 -39.174 -9.195 19.793 1.00 0.00 O1 N

ATOM 672 C12 POPCO 6 -39.991 -8.254 18.895 1.00 0.00 O1 C

ATOM 673 H12A POPCO 6 -40.945 -8.714 18.805 1.00 0.00 O1 H

ATOM 674 H12B POPCO 6 -39.602 -8.229 17.891 1.00 0.00 O1 H

ATOM 675 C13 POPCO 6 -40.079 -9.308 21.099 1.00 0.00 O1 C

ATOM 676 H13A POPCO 6 -40.970 -9.869 20.771 1.00 0.00 O1 H

ATOM 677 H13B POPCO 6 -40.394 -8.265 21.422 1.00 0.00 O1 H

ATOM 678 H13C POPCO 6 -39.643 -9.975 21.833 1.00 0.00 O1 H

ATOM 679 C14 POPCO 6 -38.907 -10.487 19.183 1.00 0.00 O1 C

ATOM 680 H14A POPCO 6 -38.380 -10.477 18.270 1.00 0.00 O1 H

ATOM 681 H14B POPCO 6 -39.818 -11.048 19.029 1.00 0.00 O1 H

ATOM 682 H14C POPCO 6 -38.307 -11.112 19.741 1.00 0.00 O1 H

ATOM 683 C15 POPCO 6 -37.848 -8.527 20.192 1.00 0.00 O1 C

ATOM 684 H15A POPCO 6 -37.125 -8.578 19.404 1.00 0.00 O1 H

ATOM 685 H15B POPCO 6 -38.030 -7.477 20.463 1.00 0.00 O1 H

ATOM 686 H15C POPCO 6 -37.441 -8.982 21.035 1.00 0.00 O1 H

ATOM 687 C11 POPCO 6 -40.239 -6.766 19.247 1.00 0.00 O1 C

ATOM 688 H11A POPCO 6 -39.377 -6.268 19.734 1.00 0.00 O1 H

ATOM 689 H11B POPCO 6 -40.569 -6.394 18.204 1.00 0.00 O1 H

ATOM 690 P POPCO 6 -41.754 -5.577 21.000 1.00 0.00 O1 P

ATOM 691 O13 POPCO 6 -40.653 -5.307 21.995 1.00 0.00 O1 O

ATOM 692 O14 POPCO 6 -43.073 -5.834 21.622 1.00 0.00 O1 O

ATOM 693 O12 POPCO 6 -41.333 -6.817 20.190 1.00 0.00 O1 O

ATOM 694 O11 POPCO 6 -41.776 -4.391 19.996 1.00 0.00 O1 O

ATOM 695 C1 POPCO 6 -42.988 -4.263 19.102 1.00 0.00 O1 C

ATOM 696 HA POPCO 6 -43.696 -5.134 19.240 1.00 0.00 O1 H

ATOM 697 HB POPCO 6 -43.671 -3.407 19.507 1.00 0.00 O1 H

ATOM 698 C2 POPCO 6 -42.636 -3.903 17.591 1.00 0.00 O1 C

ATOM 699 HS POPCO 6 -43.518 -3.391 17.117 1.00 0.00 O1 H

ATOM 700 O21 POPCO 6 -42.383 -5.133 16.895 1.00 0.00 O1 O

ATOM 701 C21 POPCO 6 -43.470 -5.690 16.315 1.00 0.00 O1 C

ATOM 702 O22 POPCO 6 -44.589 -5.624 16.771 1.00 0.00 O1 O

ATOM 703 C22 POPCO 6 -43.075 -6.267 14.913 1.00 0.00 O1 C

ATOM 704 H2R POPCO 6 -42.337 -7.047 14.882 1.00 0.00 O1 H

ATOM 705 H2S POPCO 6 -44.003 -6.755 14.488 1.00 0.00 O1 H

ATOM 706 C3 POPCO 6 -41.500 -2.847 17.372 1.00 0.00 O1 C

ATOM 707 HX POPCO 6 -41.474 -2.455 16.293 1.00 0.00 O1 H

ATOM 708 HY POPCO 6 -41.760 -1.914 17.997 1.00 0.00 O1 H

ATOM 709 O31 POPCO 6 -40.255 -3.491 17.623 1.00 0.00 O1 O

ATOM 710 C31 POPCO 6 -39.334 -2.788 18.310 1.00 0.00 O1 C

ATOM 711 O32 POPCO 6 -39.472 -1.672 18.707 1.00 0.00 O1 O

ATOM 712 C32 POPCO 6 -38.086 -3.710 18.481 1.00 0.00 O1 C

ATOM 713 H2X POPCO 6 -37.752 -3.582 19.461 1.00 0.00 O1 H

ATOM 714 H2Y POPCO 6 -38.380 -4.727 18.263 1.00 0.00 O1 H

ATOM 715 C23 POPCO 6 -42.744 -5.169 13.915 1.00 0.00 O1 C

ATOM 716 H3R POPCO 6 -43.597 -4.456 13.909 1.00 0.00 O1 H

ATOM 717 H3S POPCO 6 -41.925 -4.499 14.283 1.00 0.00 O1 H

ATOM 718 C24 POPCO 6 -42.511 -5.664 12.482 1.00 0.00 O1 C

ATOM 719 H4R POPCO 6 -41.574 -6.353 12.569 1.00 0.00 O1 H

ATOM 720 H4S POPCO 6 -43.344 -6.198 12.121 1.00 0.00 O1 H

ATOM 721 C25 POPCO 6 -42.119 -4.511 11.554 1.00 0.00 O1 C

ATOM 722 H5R POPCO 6 -43.075 -4.095 11.141 1.00 0.00 O1 H

ATOM 723 H5S POPCO 6 -41.558 -3.637 12.033 1.00 0.00 O1 H

ATOM 724 C26 POPCO 6 -41.229 -5.026 10.366 1.00 0.00 O1 C

ATOM 725 H6R POPCO 6 -40.299 -5.433 10.816 1.00 0.00 O1 H

ATOM 726 H6S POPCO 6 -41.869 -5.834 9.912 1.00 0.00 O1 H

ATOM 727 C27 POPCO 6 -40.720 -4.047 9.326 1.00 0.00 O1 C

ATOM 728 H7R POPCO 6 -41.578 -3.605 8.781 1.00 0.00 O1 H

ATOM 729 H7S POPCO 6 -40.304 -3.095 9.828 1.00 0.00 O1 H

ATOM 730 C28 POPCO 6 -39.620 -4.688 8.405 1.00 0.00 O1 C

ATOM 731 H8R POPCO 6 -38.767 -4.773 9.065 1.00 0.00 O1 H

ATOM 732 H8S POPCO 6 -39.875 -5.680 8.153 1.00 0.00 O1 H

ATOM 733 C29 POPCO 6 -39.443 -3.900 7.184 1.00 0.00 O1 C

ATOM 734 H91 POPCO 6 -38.922 -2.894 7.308 1.00 0.00 O1 H

ATOM 735 C210 POPCO 6 -39.704 -4.256 5.937 1.00 0.00 O1 C

ATOM 736 H101 POPCO 6 -39.463 -3.451 5.156 1.00 0.00 O1 H

ATOM 737 C211 POPCO 6 -40.299 -5.536 5.488 1.00 0.00 O1 C

ATOM 738 H11R POPCO 6 -40.396 -6.280 6.297 1.00 0.00 O1 H

ATOM 739 H11S POPCO 6 -41.339 -5.333 5.199 1.00 0.00 O1 H

ATOM 740 C212 POPCO 6 -39.764 -6.260 4.200 1.00 0.00 O1 C

ATOM 741 H12R POPCO 6 -38.724 -6.389 4.440 1.00 0.00 O1 H

ATOM 742 H12S POPCO 6 -40.145 -7.314 4.033 1.00 0.00 O1 H

ATOM 743 C213 POPCO 6 -39.978 -5.550 2.829 1.00 0.00 O1 C

ATOM 744 H13R POPCO 6 -41.014 -5.636 2.482 1.00 0.00 O1 H

ATOM 745 H13S POPCO 6 -39.610 -4.523 3.024 1.00 0.00 O1 H

ATOM 746 C214 POPCO 6 -39.175 -6.210 1.676 1.00 0.00 O1 C

ATOM 747 H14R POPCO 6 -38.057 -6.094 1.867 1.00 0.00 O1 H

ATOM 748 H14S POPCO 6 -39.401 -7.276 1.670 1.00 0.00 O1 H

ATOM 749 C215 POPCO 6 -39.552 -5.712 0.238 1.00 0.00 O1 C

ATOM 750 H15R POPCO 6 -40.624 -5.574 0.055 1.00 0.00 O1 H

ATOM 751 H15S POPCO 6 -39.000 -4.680 0.216 1.00 0.00 O1 H

ATOM 752 C216 POPCO 6 -39.023 -6.509 -0.928 1.00 0.00 O1 C

ATOM 753 H16R POPCO 6 -37.976 -6.228 -0.938 1.00 0.00 O1 H

ATOM 754 H16S POPCO 6 -39.291 -7.575 -0.804 1.00 0.00 O1 H

ATOM 755 C217 POPCO 6 -39.780 -6.136 -2.264 1.00 0.00 O1 C

ATOM 756 H17R POPCO 6 -40.896 -6.064 -2.050 1.00 0.00 O1 H

ATOM 757 H17S POPCO 6 -39.472 -5.160 -2.836 1.00 0.00 O1 H

ATOM 758 C218 POPCO 6 -39.644 -7.229 -3.312 1.00 0.00 O1 C

ATOM 759 H18R POPCO 6 -40.243 -6.936 -4.237 1.00 0.00 O1 H

ATOM 760 H18S POPCO 6 -38.596 -7.403 -3.770 1.00 0.00 O1 H

ATOM 761 H18T POPCO 6 -40.043 -8.234 -2.906 1.00 0.00 O1 H

ATOM 762 C33 POPCO 6 -37.073 -3.353 17.376 1.00 0.00 O1 C

ATOM 763 H3X POPCO 6 -36.755 -2.224 17.439 1.00 0.00 O1 H

ATOM 764 H3Y POPCO 6 -36.238 -3.987 17.647 1.00 0.00 O1 H

ATOM 765 C34 POPCO 6 -37.524 -3.915 16.008 1.00 0.00 O1 C

ATOM 766 H4X POPCO 6 -37.658 -4.953 16.187 1.00 0.00 O1 H

ATOM 767 H4Y POPCO 6 -38.462 -3.453 15.639 1.00 0.00 O1 H

ATOM 768 C35 POPCO 6 -36.494 -3.656 14.879 1.00 0.00 O1 C

ATOM 769 H5X POPCO 6 -35.863 -2.718 14.983 1.00 0.00 O1 H

ATOM 770 H5Y POPCO 6 -35.790 -4.505 14.998 1.00 0.00 O1 H

ATOM 771 C36 POPCO 6 -37.155 -3.653 13.529 1.00 0.00 O1 C

ATOM 772 H6X POPCO 6 -37.637 -4.570 13.409 1.00 0.00 O1 H

ATOM 773 H6Y POPCO 6 -37.929 -2.956 13.600 1.00 0.00 O1 H

ATOM 774 C37 POPCO 6 -36.076 -3.618 12.449 1.00 0.00 O1 C

ATOM 775 H7X POPCO 6 -35.626 -2.643 12.658 1.00 0.00 O1 H

ATOM 776 H7Y POPCO 6 -35.375 -4.394 12.824 1.00 0.00 O1 H

ATOM 777 C38 POPCO 6 -36.555 -3.605 11.058 1.00 0.00 O1 C

ATOM 778 H8X POPCO 6 -37.317 -4.392 10.904 1.00 0.00 O1 H

ATOM 779 H8Y POPCO 6 -37.172 -2.754 10.787 1.00 0.00 O1 H

ATOM 780 C39 POPCO 6 -35.502 -3.794 9.991 1.00 0.00 O1 C

ATOM 781 H9X POPCO 6 -34.915 -2.825 9.832 1.00 0.00 O1 H

ATOM 782 H9Y POPCO 6 -34.830 -4.640 10.186 1.00 0.00 O1 H

ATOM 783 C310 POPCO 6 -36.081 -4.275 8.647 1.00 0.00 O1 C

ATOM 784 H10X POPCO 6 -36.724 -5.146 8.766 1.00 0.00 O1 H

ATOM 785 H10Y POPCO 6 -36.779 -3.491 8.284 1.00 0.00 O1 H

ATOM 786 C311 POPCO 6 -35.042 -4.568 7.546 1.00 0.00 O1 C

ATOM 787 H11X POPCO 6 -34.229 -3.883 7.537 1.00 0.00 O1 H

ATOM 788 H11Y POPCO 6 -34.721 -5.572 7.974 1.00 0.00 O1 H

ATOM 789 C312 POPCO 6 -35.681 -4.793 6.140 1.00 0.00 O1 C

ATOM 790 H12X POPCO 6 -36.517 -5.483 6.259 1.00 0.00 O1 H

ATOM 791 H12Y POPCO 6 -36.050 -3.828 5.740 1.00 0.00 O1 H

ATOM 792 C313 POPCO 6 -34.757 -5.324 4.999 1.00 0.00 O1 C

ATOM 793 H13X POPCO 6 -33.731 -5.002 5.057 1.00 0.00 O1 H

ATOM 794 H13Y POPCO 6 -34.618 -6.422 5.219 1.00 0.00 O1 H

ATOM 795 C314 POPCO 6 -35.331 -4.989 3.576 1.00 0.00 O1 C

ATOM 796 H14X POPCO 6 -36.342 -5.447 3.410 1.00 0.00 O1 H

ATOM 797 H14Y POPCO 6 -35.574 -3.905 3.346 1.00 0.00 O1 H

ATOM 798 C315 POPCO 6 -34.518 -5.671 2.443 1.00 0.00 O1 C

ATOM 799 H15X POPCO 6 -33.594 -5.115 2.308 1.00 0.00 O1 H

ATOM 800 H15Y POPCO 6 -34.356 -6.751 2.780 1.00 0.00 O1 H

ATOM 801 C316 POPCO 6 -35.239 -5.544 1.034 1.00 0.00 O1 C

ATOM 802 H16X POPCO 6 -36.125 -6.208 1.057 1.00 0.00 O1 H

ATOM 803 H16Y POPCO 6 -35.561 -4.528 1.011 1.00 0.00 O1 H

ATOM 804 H16Z POPCO 6 -34.527 -5.895 0.276 1.00 0.00 O1 H

ATOM 805 N POPCO 7 -34.620 -40.208 21.209 1.00 0.00 O1 N

ATOM 806 C12 POPCO 7 -33.782 -39.574 20.095 1.00 0.00 O1 C

ATOM 807 H12A POPCO 7 -33.042 -38.932 20.650 1.00 0.00 O1 H

ATOM 808 H12B POPCO 7 -34.304 -38.817 19.566 1.00 0.00 O1 H

ATOM 809 C13 POPCO 7 -33.745 -40.610 22.327 1.00 0.00 O1 C

ATOM 810 H13A POPCO 7 -33.334 -39.730 22.774 1.00 0.00 O1 H

ATOM 811 H13B POPCO 7 -32.838 -41.260 22.015 1.00 0.00 O1 H

ATOM 812 H13C POPCO 7 -34.241 -41.028 23.120 1.00 0.00 O1 H

ATOM 813 C14 POPCO 7 -35.548 -39.135 21.734 1.00 0.00 O1 C

ATOM 814 H14A POPCO 7 -36.055 -38.705 20.855 1.00 0.00 O1 H

ATOM 815 H14B POPCO 7 -34.960 -38.387 22.203 1.00 0.00 O1 H

ATOM 816 H14C POPCO 7 -36.334 -39.464 22.338 1.00 0.00 O1 H

ATOM 817 C15 POPCO 7 -35.459 -41.304 20.721 1.00 0.00 O1 C

ATOM 818 H15A POPCO 7 -36.054 -41.019 19.856 1.00 0.00 O1 H

ATOM 819 H15B POPCO 7 -34.810 -42.134 20.387 1.00 0.00 O1 H

ATOM 820 H15C POPCO 7 -36.112 -41.587 21.514 1.00 0.00 O1 H

ATOM 821 C11 POPCO 7 -33.013 -40.425 19.104 1.00 0.00 O1 C

ATOM 822 H11A POPCO 7 -33.802 -40.964 18.564 1.00 0.00 O1 H

ATOM 823 H11B POPCO 7 -32.590 -39.626 18.369 1.00 0.00 O1 H

ATOM 824 P POPCO 7 -31.869 -42.672 20.095 1.00 0.00 O1 P

ATOM 825 O13 POPCO 7 -31.980 -42.952 21.558 1.00 0.00 O1 O

ATOM 826 O14 POPCO 7 -30.572 -43.147 19.565 1.00 0.00 O1 O

ATOM 827 O12 POPCO 7 -31.990 -41.142 19.723 1.00 0.00 O1 O

ATOM 828 O11 POPCO 7 -33.073 -43.356 19.296 1.00 0.00 O1 O

ATOM 829 C1 POPCO 7 -32.948 -43.412 17.829 1.00 0.00 O1 C

ATOM 830 HA POPCO 7 -32.506 -42.444 17.414 1.00 0.00 O1 H

ATOM 831 HB POPCO 7 -32.160 -44.123 17.616 1.00 0.00 O1 H

ATOM 832 C2 POPCO 7 -34.168 -43.679 17.008 1.00 0.00 O1 C

ATOM 833 HS POPCO 7 -34.320 -44.808 17.106 1.00 0.00 O1 H

ATOM 834 O21 POPCO 7 -34.042 -43.320 15.613 1.00 0.00 O1 O

ATOM 835 C21 POPCO 7 -34.623 -44.137 14.699 1.00 0.00 O1 C

ATOM 836 O22 POPCO 7 -35.084 -45.254 14.897 1.00 0.00 O1 O

ATOM 837 C22 POPCO 7 -34.615 -43.343 13.460 1.00 0.00 O1 C

ATOM 838 H2R POPCO 7 -35.167 -42.380 13.596 1.00 0.00 O1 H

ATOM 839 H2S POPCO 7 -33.531 -43.063 13.332 1.00 0.00 O1 H

ATOM 840 C3 POPCO 7 -35.571 -43.214 17.593 1.00 0.00 O1 C

ATOM 841 HX POPCO 7 -36.332 -43.745 17.003 1.00 0.00 O1 H

ATOM 842 HY POPCO 7 -35.667 -43.492 18.643 1.00 0.00 O1 H

ATOM 843 O31 POPCO 7 -35.726 -41.848 17.538 1.00 0.00 O1 O

ATOM 844 C31 POPCO 7 -36.494 -41.285 16.559 1.00 0.00 O1 C

ATOM 845 O32 POPCO 7 -36.994 -41.852 15.633 1.00 0.00 O1 O

ATOM 846 C32 POPCO 7 -36.439 -39.804 16.795 1.00 0.00 O1 C

ATOM 847 H2X POPCO 7 -37.291 -39.253 16.222 1.00 0.00 O1 H

ATOM 848 H2Y POPCO 7 -36.518 -39.545 17.861 1.00 0.00 O1 H

ATOM 849 C23 POPCO 7 -35.033 -44.114 12.175 1.00 0.00 O1 C

ATOM 850 H3R POPCO 7 -34.367 -44.991 12.159 1.00 0.00 O1 H

ATOM 851 H3S POPCO 7 -36.096 -44.440 12.308 1.00 0.00 O1 H

ATOM 852 C24 POPCO 7 -34.744 -43.329 10.887 1.00 0.00 O1 C

ATOM 853 H4R POPCO 7 -35.296 -42.269 10.924 1.00 0.00 O1 H

ATOM 854 H4S POPCO 7 -33.674 -42.911 10.812 1.00 0.00 O1 H

ATOM 855 C25 POPCO 7 -34.965 -44.157 9.570 1.00 0.00 O1 C

ATOM 856 H5R POPCO 7 -34.277 -44.988 9.492 1.00 0.00 O1 H

ATOM 857 H5S POPCO 7 -35.982 -44.561 9.510 1.00 0.00 O1 H

ATOM 858 C26 POPCO 7 -34.615 -43.289 8.293 1.00 0.00 O1 C

ATOM 859 H6R POPCO 7 -35.091 -42.288 8.284 1.00 0.00 O1 H

ATOM 860 H6S POPCO 7 -33.488 -43.175 8.248 1.00 0.00 O1 H

ATOM 861 C27 POPCO 7 -35.158 -44.030 7.053 1.00 0.00 O1 C

ATOM 862 H7R POPCO 7 -34.526 -44.989 6.972 1.00 0.00 O1 H

ATOM 863 H7S POPCO 7 -36.237 -44.314 7.179 1.00 0.00 O1 H

ATOM 864 C28 POPCO 7 -34.870 -43.265 5.672 1.00 0.00 O1 C

ATOM 865 H8R POPCO 7 -35.459 -42.318 5.884 1.00 0.00 O1 H

ATOM 866 H8S POPCO 7 -33.769 -43.075 5.588 1.00 0.00 O1 H

ATOM 867 C29 POPCO 7 -35.321 -43.951 4.456 1.00 0.00 O1 C

ATOM 868 H91 POPCO 7 -36.312 -44.302 4.446 1.00 0.00 O1 H

ATOM 869 C210 POPCO 7 -34.572 -44.207 3.407 1.00 0.00 O1 C

ATOM 870 H101 POPCO 7 -35.054 -44.743 2.610 1.00 0.00 O1 H

ATOM 871 C211 POPCO 7 -33.096 -43.880 3.110 1.00 0.00 O1 C

ATOM 872 H11R POPCO 7 -32.718 -43.133 3.794 1.00 0.00 O1 H

ATOM 873 H11S POPCO 7 -32.405 -44.797 3.212 1.00 0.00 O1 H

ATOM 874 C212 POPCO 7 -32.922 -43.395 1.594 1.00 0.00 O1 C

ATOM 875 H12R POPCO 7 -33.562 -42.528 1.504 1.00 0.00 O1 H

ATOM 876 H12S POPCO 7 -31.844 -43.092 1.590 1.00 0.00 O1 H

ATOM 877 C213 POPCO 7 -33.180 -44.419 0.454 1.00 0.00 O1 C

ATOM 878 H13R POPCO 7 -32.469 -45.287 0.613 1.00 0.00 O1 H

ATOM 879 H13S POPCO 7 -34.214 -44.778 0.449 1.00 0.00 O1 H

ATOM 880 C214 POPCO 7 -32.802 -43.937 -0.893 1.00 0.00 O1 C

ATOM 881 H14R POPCO 7 -33.474 -43.100 -1.283 1.00 0.00 O1 H

ATOM 882 H14S POPCO 7 -31.785 -43.435 -0.890 1.00 0.00 O1 H

ATOM 883 C215 POPCO 7 -32.958 -44.917 -2.054 1.00 0.00 O1 C

ATOM 884 H15R POPCO 7 -32.112 -45.520 -2.215 1.00 0.00 O1 H

ATOM 885 H15S POPCO 7 -33.837 -45.532 -2.090 1.00 0.00 O1 H

ATOM 886 C216 POPCO 7 -33.092 -44.213 -3.402 1.00 0.00 O1 C

ATOM 887 H16R POPCO 7 -34.032 -43.579 -3.318 1.00 0.00 O1 H

ATOM 888 H16S POPCO 7 -32.213 -43.514 -3.486 1.00 0.00 O1 H

ATOM 889 C217 POPCO 7 -33.253 -45.263 -4.524 1.00 0.00 O1 C

ATOM 890 H17R POPCO 7 -32.357 -45.859 -4.350 1.00 0.00 O1 H

ATOM 891 H17S POPCO 7 -34.207 -45.812 -4.249 1.00 0.00 O1 H

ATOM 892 C218 POPCO 7 -33.351 -44.603 -5.896 1.00 0.00 O1 C

ATOM 893 H18R POPCO 7 -33.710 -45.410 -6.573 1.00 0.00 O1 H

ATOM 894 H18S POPCO 7 -34.179 -43.903 -5.834 1.00 0.00 O1 H

ATOM 895 H18T POPCO 7 -32.390 -44.129 -6.273 1.00 0.00 O1 H

ATOM 896 C33 POPCO 7 -35.226 -39.211 16.136 1.00 0.00 O1 C

ATOM 897 H3X POPCO 7 -35.196 -38.110 16.297 1.00 0.00 O1 H

ATOM 898 H3Y POPCO 7 -34.353 -39.721 16.579 1.00 0.00 O1 H

ATOM 899 C34 POPCO 7 -35.209 -39.289 14.596 1.00 0.00 O1 C

ATOM 900 H4X POPCO 7 -35.895 -40.101 14.305 1.00 0.00 O1 H

ATOM 901 H4Y POPCO 7 -35.779 -38.395 14.167 1.00 0.00 O1 H

ATOM 902 C35 POPCO 7 -33.882 -39.516 13.997 1.00 0.00 O1 C

ATOM 903 H5X POPCO 7 -33.136 -38.794 14.305 1.00 0.00 O1 H

ATOM 904 H5Y POPCO 7 -33.506 -40.509 14.324 1.00 0.00 O1 H

ATOM 905 C36 POPCO 7 -34.015 -39.417 12.455 1.00 0.00 O1 C

ATOM 906 H6X POPCO 7 -35.032 -39.863 12.169 1.00 0.00 O1 H

ATOM 907 H6Y POPCO 7 -33.951 -38.318 12.236 1.00 0.00 O1 H

ATOM 908 C37 POPCO 7 -32.767 -39.951 11.708 1.00 0.00 O1 C

ATOM 909 H7X POPCO 7 -31.868 -39.402 12.139 1.00 0.00 O1 H

ATOM 910 H7Y POPCO 7 -32.596 -40.988 11.905 1.00 0.00 O1 H

ATOM 911 C38 POPCO 7 -32.881 -39.592 10.220 1.00 0.00 O1 C

ATOM 912 H8X POPCO 7 -33.895 -39.981 9.791 1.00 0.00 O1 H

ATOM 913 H8Y POPCO 7 -32.997 -38.468 10.083 1.00 0.00 O1 H

ATOM 914 C39 POPCO 7 -31.745 -40.031 9.170 1.00 0.00 O1 C

ATOM 915 H9X POPCO 7 -30.905 -39.332 9.182 1.00 0.00 O1 H

ATOM 916 H9Y POPCO 7 -31.301 -40.966 9.506 1.00 0.00 O1 H

ATOM 917 C310 POPCO 7 -32.133 -40.023 7.724 1.00 0.00 O1 C

ATOM 918 H10X POPCO 7 -32.807 -40.871 7.478 1.00 0.00 O1 H

ATOM 919 H10Y POPCO 7 -32.700 -39.117 7.509 1.00 0.00 O1 H

ATOM 920 C311 POPCO 7 -31.019 -40.137 6.566 1.00 0.00 O1 C

ATOM 921 H11X POPCO 7 -30.102 -39.479 6.779 1.00 0.00 O1 H

ATOM 922 H11Y POPCO 7 -30.690 -41.205 6.757 1.00 0.00 O1 H

ATOM 923 C312 POPCO 7 -31.628 -39.846 5.174 1.00 0.00 O1 C

ATOM 924 H12X POPCO 7 -32.730 -40.169 5.155 1.00 0.00 O1 H

ATOM 925 H12Y POPCO 7 -31.565 -38.765 4.876 1.00 0.00 O1 H

ATOM 926 C313 POPCO 7 -30.838 -40.522 4.034 1.00 0.00 O1 C

ATOM 927 H13X POPCO 7 -29.891 -39.920 3.957 1.00 0.00 O1 H

ATOM 928 H13Y POPCO 7 -30.509 -41.511 4.268 1.00 0.00 O1 H

ATOM 929 C314 POPCO 7 -31.479 -40.199 2.652 1.00 0.00 O1 C

ATOM 930 H14X POPCO 7 -32.164 -41.081 2.485 1.00 0.00 O1 H

ATOM 931 H14Y POPCO 7 -32.088 -39.319 2.750 1.00 0.00 O1 H

ATOM 932 C315 POPCO 7 -30.507 -39.961 1.490 1.00 0.00 O1 C

ATOM 933 H15X POPCO 7 -30.063 -38.989 1.683 1.00 0.00 O1 H

ATOM 934 H15Y POPCO 7 -29.696 -40.622 1.469 1.00 0.00 O1 H

ATOM 935 C316 POPCO 7 -31.180 -39.973 0.033 1.00 0.00 O1 C

ATOM 936 H16X POPCO 7 -31.640 -38.984 -0.076 1.00 0.00 O1 H

ATOM 937 H16Y POPCO 7 -30.395 -40.214 -0.772 1.00 0.00 O1 H

ATOM 938 H16Z POPCO 7 -31.996 -40.755 -0.056 1.00 0.00 O1 H

ATOM 939 N POPCO 8 -28.451 -35.731 19.029 1.00 0.00 O1 N

ATOM 940 C12 POPCO 8 -29.004 -37.132 19.342 1.00 0.00 O1 C

ATOM 941 H12A POPCO 8 -28.725 -37.452 20.337 1.00 0.00 O1 H

ATOM 942 H12B POPCO 8 -28.440 -37.739 18.658 1.00 0.00 O1 H

ATOM 943 C13 POPCO 8 -28.836 -34.718 20.029 1.00 0.00 O1 C

ATOM 944 H13A POPCO 8 -28.846 -35.014 21.076 1.00 0.00 O1 H

ATOM 945 H13B POPCO 8 -29.892 -34.437 19.785 1.00 0.00 O1 H

ATOM 946 H13C POPCO 8 -28.276 -33.823 19.897 1.00 0.00 O1 H

ATOM 947 C14 POPCO 8 -26.962 -35.715 19.017 1.00 0.00 O1 C

ATOM 948 H14A POPCO 8 -26.622 -36.577 18.402 1.00 0.00 O1 H

ATOM 949 H14B POPCO 8 -26.554 -35.955 19.992 1.00 0.00 O1 H

ATOM 950 H14C POPCO 8 -26.452 -34.883 18.620 1.00 0.00 O1 H

ATOM 951 C15 POPCO 8 -28.809 -35.305 17.637 1.00 0.00 O1 C

ATOM 952 H15A POPCO 8 -28.695 -36.119 16.849 1.00 0.00 O1 H

ATOM 953 H15B POPCO 8 -29.843 -35.206 17.573 1.00 0.00 O1 H

ATOM 954 H15C POPCO 8 -28.288 -34.473 17.263 1.00 0.00 O1 H

ATOM 955 C11 POPCO 8 -30.519 -37.422 19.203 1.00 0.00 O1 C

ATOM 956 H11A POPCO 8 -30.789 -37.254 18.165 1.00 0.00 O1 H

ATOM 957 H11B POPCO 8 -30.609 -38.468 19.462 1.00 0.00 O1 H

ATOM 958 P POPCO 8 -32.566 -35.930 19.953 1.00 0.00 O1 P

ATOM 959 O13 POPCO 8 -32.960 -35.116 21.133 1.00 0.00 O1 O

ATOM 960 O14 POPCO 8 -33.541 -36.968 19.551 1.00 0.00 O1 O

ATOM 961 O12 POPCO 8 -31.220 -36.642 20.156 1.00 0.00 O1 O

ATOM 962 O11 POPCO 8 -32.350 -34.914 18.788 1.00 0.00 O1 O

ATOM 963 C1 POPCO 8 -32.392 -35.273 17.445 1.00 0.00 O1 C

ATOM 964 HA POPCO 8 -31.387 -35.538 17.071 1.00 0.00 O1 H

ATOM 965 HB POPCO 8 -33.097 -36.172 17.300 1.00 0.00 O1 H

ATOM 966 C2 POPCO 8 -32.927 -34.185 16.437 1.00 0.00 O1 C

ATOM 967 HS POPCO 8 -34.039 -34.037 16.632 1.00 0.00 O1 H

ATOM 968 O21 POPCO 8 -32.784 -34.729 15.109 1.00 0.00 O1 O

ATOM 969 C21 POPCO 8 -33.454 -34.151 14.184 1.00 0.00 O1 C

ATOM 970 O22 POPCO 8 -34.048 -33.090 14.208 1.00 0.00 O1 O

ATOM 971 C22 POPCO 8 -33.529 -35.081 12.950 1.00 0.00 O1 C

ATOM 972 H2R POPCO 8 -32.527 -35.407 12.776 1.00 0.00 O1 H

ATOM 973 H2S POPCO 8 -34.052 -35.999 13.275 1.00 0.00 O1 H

ATOM 974 C3 POPCO 8 -32.238 -32.840 16.578 1.00 0.00 O1 C

ATOM 975 HX POPCO 8 -32.672 -32.093 15.864 1.00 0.00 O1 H

ATOM 976 HY POPCO 8 -32.379 -32.503 17.644 1.00 0.00 O1 H

ATOM 977 O31 POPCO 8 -30.813 -32.979 16.398 1.00 0.00 O1 O

ATOM 978 C31 POPCO 8 -30.146 -31.788 16.533 1.00 0.00 O1 C

ATOM 979 O32 POPCO 8 -30.660 -30.721 16.889 1.00 0.00 O1 O

ATOM 980 C32 POPCO 8 -28.694 -31.862 16.102 1.00 0.00 O1 C

ATOM 981 H2X POPCO 8 -28.053 -30.969 16.270 1.00 0.00 O1 H

ATOM 982 H2Y POPCO 8 -28.177 -32.728 16.596 1.00 0.00 O1 H

ATOM 983 C23 POPCO 8 -34.030 -34.347 11.718 1.00 0.00 O1 C

ATOM 984 H3R POPCO 8 -35.058 -33.908 11.857 1.00 0.00 O1 H

ATOM 985 H3S POPCO 8 -33.307 -33.539 11.414 1.00 0.00 O1 H

ATOM 986 C24 POPCO 8 -34.207 -35.293 10.532 1.00 0.00 O1 C

ATOM 987 H4R POPCO 8 -33.244 -35.791 10.393 1.00 0.00 O1 H

ATOM 988 H4S POPCO 8 -34.785 -36.189 10.687 1.00 0.00 O1 H

ATOM 989 C25 POPCO 8 -34.569 -34.568 9.174 1.00 0.00 O1 C

ATOM 990 H5R POPCO 8 -35.679 -34.757 9.100 1.00 0.00 O1 H

ATOM 991 H5S POPCO 8 -34.385 -33.488 9.213 1.00 0.00 O1 H

ATOM 992 C26 POPCO 8 -33.932 -35.229 7.915 1.00 0.00 O1 C

ATOM 993 H6R POPCO 8 -32.808 -35.191 7.784 1.00 0.00 O1 H

ATOM 994 H6S POPCO 8 -34.203 -36.317 8.024 1.00 0.00 O1 H

ATOM 995 C27 POPCO 8 -34.539 -34.789 6.577 1.00 0.00 O1 C

ATOM 996 H7R POPCO 8 -35.584 -35.087 6.500 1.00 0.00 O1 H

ATOM 997 H7S POPCO 8 -34.501 -33.715 6.595 1.00 0.00 O1 H

ATOM 998 C28 POPCO 8 -33.632 -35.314 5.378 1.00 0.00 O1 C

ATOM 999 H8R POPCO 8 -32.631 -35.111 5.708 1.00 0.00 O1 H

ATOM 1000 H8S POPCO 8 -33.790 -36.369 5.197 1.00 0.00 O1 H

ATOM 1001 C29 POPCO 8 -33.893 -34.474 4.110 1.00 0.00 O1 C

ATOM 1002 H91 POPCO 8 -33.575 -33.431 4.248 1.00 0.00 O1 H

ATOM 1003 C210 POPCO 8 -34.388 -34.835 2.874 1.00 0.00 O1 C

ATOM 1004 H101 POPCO 8 -34.503 -34.124 2.040 1.00 0.00 O1 H

ATOM 1005 C211 POPCO 8 -34.890 -36.198 2.512 1.00 0.00 O1 C

ATOM 1006 H11R POPCO 8 -34.837 -36.833 3.437 1.00 0.00 O1 H

ATOM 1007 H11S POPCO 8 -35.995 -36.074 2.287 1.00 0.00 O1 H

ATOM 1008 C212 POPCO 8 -34.199 -36.706 1.209 1.00 0.00 O1 C

ATOM 1009 H12R POPCO 8 -33.205 -37.215 1.395 1.00 0.00 O1 H

ATOM 1010 H12S POPCO 8 -34.824 -37.436 0.700 1.00 0.00 O1 H

ATOM 1011 C213 POPCO 8 -33.894 -35.673 0.102 1.00 0.00 O1 C

ATOM 1012 H13R POPCO 8 -34.506 -34.731 0.179 1.00 0.00 O1 H

ATOM 1013 H13S POPCO 8 -32.905 -35.300 0.357 1.00 0.00 O1 H

ATOM 1014 C214 POPCO 8 -33.807 -36.330 -1.288 1.00 0.00 O1 C

ATOM 1015 H14R POPCO 8 -33.280 -37.257 -1.098 1.00 0.00 O1 H

ATOM 1016 H14S POPCO 8 -34.865 -36.583 -1.514 1.00 0.00 O1 H

ATOM 1017 C215 POPCO 8 -33.171 -35.412 -2.293 1.00 0.00 O1 C

ATOM 1018 H15R POPCO 8 -33.748 -34.442 -2.309 1.00 0.00 O1 H

ATOM 1019 H15S POPCO 8 -32.187 -35.146 -1.997 1.00 0.00 O1 H

ATOM 1020 C216 POPCO 8 -33.153 -36.028 -3.759 1.00 0.00 O1 C

ATOM 1021 H16R POPCO 8 -32.531 -36.969 -3.897 1.00 0.00 O1 H

ATOM 1022 H16S POPCO 8 -34.237 -36.135 -3.987 1.00 0.00 O1 H

ATOM 1023 C217 POPCO 8 -32.413 -35.142 -4.837 1.00 0.00 O1 C

ATOM 1024 H17R POPCO 8 -33.013 -34.225 -4.935 1.00 0.00 O1 H

ATOM 1025 H17S POPCO 8 -31.428 -34.800 -4.437 1.00 0.00 O1 H

ATOM 1026 C218 POPCO 8 -32.283 -35.818 -6.239 1.00 0.00 O1 C

ATOM 1027 H18R POPCO 8 -31.733 -35.057 -6.887 1.00 0.00 O1 H

ATOM 1028 H18S POPCO 8 -31.683 -36.752 -5.994 1.00 0.00 O1 H

ATOM 1029 H18T POPCO 8 -33.219 -36.085 -6.739 1.00 0.00 O1 H

ATOM 1030 C33 POPCO 8 -28.511 -32.043 14.508 1.00 0.00 O1 C

ATOM 1031 H3X POPCO 8 -28.823 -31.161 13.976 1.00 0.00 O1 H

ATOM 1032 H3Y POPCO 8 -27.360 -32.033 14.437 1.00 0.00 O1 H

ATOM 1033 C34 POPCO 8 -29.124 -33.296 13.881 1.00 0.00 O1 C

ATOM 1034 H4X POPCO 8 -28.776 -34.167 14.447 1.00 0.00 O1 H

ATOM 1035 H4Y POPCO 8 -30.238 -33.206 13.877 1.00 0.00 O1 H

ATOM 1036 C35 POPCO 8 -28.588 -33.444 12.490 1.00 0.00 O1 C

ATOM 1037 H5X POPCO 8 -28.781 -32.549 11.853 1.00 0.00 O1 H

ATOM 1038 H5Y POPCO 8 -27.504 -33.772 12.485 1.00 0.00 O1 H

ATOM 1039 C36 POPCO 8 -29.417 -34.576 11.774 1.00 0.00 O1 C

ATOM 1040 H6X POPCO 8 -29.258 -35.461 12.401 1.00 0.00 O1 H

ATOM 1041 H6Y POPCO 8 -30.498 -34.240 11.625 1.00 0.00 O1 H

ATOM 1042 C37 POPCO 8 -28.832 -34.715 10.307 1.00 0.00 O1 C

ATOM 1043 H7X POPCO 8 -28.771 -33.678 9.876 1.00 0.00 O1 H

ATOM 1044 H7Y POPCO 8 -27.813 -35.057 10.328 1.00 0.00 O1 H

ATOM 1045 C38 POPCO 8 -29.684 -35.577 9.326 1.00 0.00 O1 C

ATOM 1046 H8X POPCO 8 -29.973 -36.497 9.714 1.00 0.00 O1 H

ATOM 1047 H8Y POPCO 8 -30.554 -34.996 9.038 1.00 0.00 O1 H

ATOM 1048 C39 POPCO 8 -28.887 -35.797 8.011 1.00 0.00 O1 C

ATOM 1049 H9X POPCO 8 -28.208 -34.982 7.726 1.00 0.00 O1 H

ATOM 1050 H9Y POPCO 8 -28.258 -36.663 8.026 1.00 0.00 O1 H

ATOM 1051 C310 POPCO 8 -29.758 -35.937 6.811 1.00 0.00 O1 C

ATOM 1052 H10X POPCO 8 -30.621 -36.541 7.027 1.00 0.00 O1 H

ATOM 1053 H10Y POPCO 8 -30.119 -34.897 6.596 1.00 0.00 O1 H

ATOM 1054 C311 POPCO 8 -29.060 -36.563 5.620 1.00 0.00 O1 C

ATOM 1055 H11X POPCO 8 -28.057 -36.059 5.609 1.00 0.00 O1 H

ATOM 1056 H11Y POPCO 8 -28.941 -37.656 5.709 1.00 0.00 O1 H

ATOM 1057 C312 POPCO 8 -29.882 -36.327 4.302 1.00 0.00 O1 C

ATOM 1058 H12X POPCO 8 -30.863 -36.890 4.365 1.00 0.00 O1 H

ATOM 1059 H12Y POPCO 8 -30.163 -35.238 4.266 1.00 0.00 O1 H

ATOM 1060 C313 POPCO 8 -29.076 -36.607 3.014 1.00 0.00 O1 C

ATOM 1061 H13X POPCO 8 -28.165 -36.018 3.093 1.00 0.00 O1 H

ATOM 1062 H13Y POPCO 8 -28.729 -37.654 3.023 1.00 0.00 O1 H

ATOM 1063 C314 POPCO 8 -29.854 -36.240 1.697 1.00 0.00 O1 C

ATOM 1064 H14X POPCO 8 -30.766 -36.857 1.639 1.00 0.00 O1 H

ATOM 1065 H14Y POPCO 8 -30.216 -35.187 1.794 1.00 0.00 O1 H

ATOM 1066 C315 POPCO 8 -29.023 -36.388 0.445 1.00 0.00 O1 C

ATOM 1067 H15X POPCO 8 -28.118 -35.725 0.575 1.00 0.00 O1 H

ATOM 1068 H15Y POPCO 8 -28.595 -37.438 0.404 1.00 0.00 O1 H

ATOM 1069 C316 POPCO 8 -29.879 -36.100 -0.766 1.00 0.00 O1 C

ATOM 1070 H16X POPCO 8 -30.018 -35.059 -0.828 1.00 0.00 O1 H

ATOM 1071 H16Y POPCO 8 -29.508 -36.315 -1.774 1.00 0.00 O1 H

ATOM 1072 H16Z POPCO 8 -30.859 -36.658 -0.601 1.00 0.00 O1 H

ATOM 1073 N POPCO 9 -39.478 -26.062 22.011 1.00 0.00 O1 N

ATOM 1074 C12 POPCO 9 -39.411 -24.588 21.449 1.00 0.00 O1 C

ATOM 1075 H12A POPCO 9 -40.105 -23.915 21.943 1.00 0.00 O1 H

ATOM 1076 H12B POPCO 9 -39.837 -24.735 20.441 1.00 0.00 O1 H

ATOM 1077 C13 POPCO 9 -39.366 -26.056 23.436 1.00 0.00 O1 C

ATOM 1078 H13A POPCO 9 -40.248 -25.747 23.981 1.00 0.00 O1 H

ATOM 1079 H13B POPCO 9 -38.620 -25.311 23.692 1.00 0.00 O1 H

ATOM 1080 H13C POPCO 9 -38.912 -27.006 23.777 1.00 0.00 O1 H

ATOM 1081 C14 POPCO 9 -40.727 -26.630 21.466 1.00 0.00 O1 C

ATOM 1082 H14A POPCO 9 -40.555 -26.950 20.442 1.00 0.00 O1 H

ATOM 1083 H14B POPCO 9 -41.483 -25.829 21.469 1.00 0.00 O1 H

ATOM 1084 H14C POPCO 9 -40.983 -27.494 21.882 1.00 0.00 O1 H

ATOM 1085 C15 POPCO 9 -38.323 -26.845 21.483 1.00 0.00 O1 C

ATOM 1086 H15A POPCO 9 -38.248 -26.784 20.388 1.00 0.00 O1 H

ATOM 1087 H15B POPCO 9 -37.305 -26.482 21.731 1.00 0.00 O1 H

ATOM 1088 H15C POPCO 9 -38.392 -27.865 21.704 1.00 0.00 O1 H

ATOM 1089 C11 POPCO 9 -38.027 -23.770 21.421 1.00 0.00 O1 C

ATOM 1090 H11A POPCO 9 -37.350 -24.362 20.782 1.00 0.00 O1 H

ATOM 1091 H11B POPCO 9 -38.166 -22.720 20.952 1.00 0.00 O1 H

ATOM 1092 P POPCO 9 -36.158 -24.193 23.352 1.00 0.00 O1 P

ATOM 1093 O13 POPCO 9 -36.505 -25.346 24.215 1.00 0.00 O1 O

ATOM 1094 O14 POPCO 9 -35.508 -23.023 23.942 1.00 0.00 O1 O

ATOM 1095 O12 POPCO 9 -37.532 -23.522 22.717 1.00 0.00 O1 O

ATOM 1096 O11 POPCO 9 -35.347 -24.777 22.108 1.00 0.00 O1 O

ATOM 1097 C1 POPCO 9 -34.814 -23.763 21.231 1.00 0.00 O1 C

ATOM 1098 HA POPCO 9 -35.607 -23.121 20.816 1.00 0.00 O1 H

ATOM 1099 HB POPCO 9 -34.018 -23.161 21.728 1.00 0.00 O1 H

ATOM 1100 C2 POPCO 9 -34.190 -24.395 19.978 1.00 0.00 O1 C

ATOM 1101 HS POPCO 9 -33.187 -24.710 20.377 1.00 0.00 O1 H

ATOM 1102 O21 POPCO 9 -34.059 -23.365 18.993 1.00 0.00 O1 O

ATOM 1103 C21 POPCO 9 -33.246 -23.822 18.043 1.00 0.00 O1 C

ATOM 1104 O22 POPCO 9 -32.353 -24.680 18.073 1.00 0.00 O1 O

ATOM 1105 C22 POPCO 9 -33.745 -23.274 16.719 1.00 0.00 O1 C

ATOM 1106 H2R POPCO 9 -34.865 -23.478 16.644 1.00 0.00 O1 H

ATOM 1107 H2S POPCO 9 -33.588 -22.164 16.650 1.00 0.00 O1 H

ATOM 1108 C3 POPCO 9 -34.940 -25.748 19.422 1.00 0.00 O1 C

ATOM 1109 HX POPCO 9 -34.330 -26.307 18.717 1.00 0.00 O1 H

ATOM 1110 HY POPCO 9 -35.077 -26.532 20.204 1.00 0.00 O1 H

ATOM 1111 O31 POPCO 9 -36.236 -25.431 18.894 1.00 0.00 O1 O

ATOM 1112 C31 POPCO 9 -36.855 -26.447 18.422 1.00 0.00 O1 C

ATOM 1113 O32 POPCO 9 -36.594 -27.682 18.617 1.00 0.00 O1 O

ATOM 1114 C32 POPCO 9 -38.043 -25.923 17.684 1.00 0.00 O1 C

ATOM 1115 H2X POPCO 9 -38.844 -26.005 18.430 1.00 0.00 O1 H

ATOM 1116 H2Y POPCO 9 -37.883 -24.819 17.441 1.00 0.00 O1 H

ATOM 1117 C23 POPCO 9 -33.081 -24.044 15.490 1.00 0.00 O1 C

ATOM 1118 H3R POPCO 9 -31.986 -23.854 15.524 1.00 0.00 O1 H

ATOM 1119 H3S POPCO 9 -33.308 -25.136 15.609 1.00 0.00 O1 H

ATOM 1120 C24 POPCO 9 -33.637 -23.679 14.126 1.00 0.00 O1 C

ATOM 1121 H4R POPCO 9 -34.729 -23.959 14.131 1.00 0.00 O1 H

ATOM 1122 H4S POPCO 9 -33.512 -22.640 13.938 1.00 0.00 O1 H

ATOM 1123 C25 POPCO 9 -32.916 -24.344 12.941 1.00 0.00 O1 C

ATOM 1124 H5R POPCO 9 -31.832 -24.039 13.003 1.00 0.00 O1 H

ATOM 1125 H5S POPCO 9 -32.922 -25.463 13.025 1.00 0.00 O1 H

ATOM 1126 C26 POPCO 9 -33.512 -24.091 11.566 1.00 0.00 O1 C

ATOM 1127 H6R POPCO 9 -34.623 -24.182 11.517 1.00 0.00 O1 H

ATOM 1128 H6S POPCO 9 -33.527 -22.924 11.373 1.00 0.00 O1 H

ATOM 1129 C27 POPCO 9 -32.675 -24.661 10.435 1.00 0.00 O1 C

ATOM 1130 H7R POPCO 9 -31.650 -24.164 10.395 1.00 0.00 O1 H

ATOM 1131 H7S POPCO 9 -32.367 -25.708 10.692 1.00 0.00 O1 H

ATOM 1132 C28 POPCO 9 -33.300 -24.325 9.068 1.00 0.00 O1 C

ATOM 1133 H8R POPCO 9 -34.399 -24.669 9.074 1.00 0.00 O1 H

ATOM 1134 H8S POPCO 9 -33.242 -23.238 8.993 1.00 0.00 O1 H

ATOM 1135 C29 POPCO 9 -32.809 -24.887 7.807 1.00 0.00 O1 C

ATOM 1136 H91 POPCO 9 -31.939 -25.506 7.863 1.00 0.00 O1 H

ATOM 1137 C210 POPCO 9 -33.239 -24.648 6.580 1.00 0.00 O1 C

ATOM 1138 H101 POPCO 9 -32.634 -25.082 5.727 1.00 0.00 O1 H

ATOM 1139 C211 POPCO 9 -34.503 -23.883 6.206 1.00 0.00 O1 C

ATOM 1140 H11R POPCO 9 -35.223 -24.007 7.098 1.00 0.00 O1 H

ATOM 1141 H11S POPCO 9 -34.244 -22.819 6.212 1.00 0.00 O1 H

ATOM 1142 C212 POPCO 9 -35.151 -24.344 4.938 1.00 0.00 O1 C

ATOM 1143 H12R POPCO 9 -34.996 -25.415 4.767 1.00 0.00 O1 H

ATOM 1144 H12S POPCO 9 -36.247 -24.216 5.003 1.00 0.00 O1 H

ATOM 1145 C213 POPCO 9 -34.718 -23.630 3.673 1.00 0.00 O1 C

ATOM 1146 H13R POPCO 9 -35.093 -22.561 3.711 1.00 0.00 O1 H

ATOM 1147 H13S POPCO 9 -33.565 -23.522 3.716 1.00 0.00 O1 H

ATOM 1148 C214 POPCO 9 -35.096 -24.299 2.336 1.00 0.00 O1 C

ATOM 1149 H14R POPCO 9 -34.491 -25.157 2.201 1.00 0.00 O1 H

ATOM 1150 H14S POPCO 9 -36.113 -24.624 2.374 1.00 0.00 O1 H

ATOM 1151 C215 POPCO 9 -35.031 -23.426 1.052 1.00 0.00 O1 C

ATOM 1152 H15R POPCO 9 -35.920 -22.678 1.055 1.00 0.00 O1 H

ATOM 1153 H15S POPCO 9 -34.090 -22.832 1.084 1.00 0.00 O1 H

ATOM 1154 C216 POPCO 9 -35.072 -24.207 -0.237 1.00 0.00 O1 C

ATOM 1155 H16R POPCO 9 -34.330 -24.949 -0.196 1.00 0.00 O1 H

ATOM 1156 H16S POPCO 9 -36.088 -24.645 -0.227 1.00 0.00 O1 H

ATOM 1157 C217 POPCO 9 -34.987 -23.330 -1.523 1.00 0.00 O1 C

ATOM 1158 H17R POPCO 9 -35.432 -22.379 -1.291 1.00 0.00 O1 H

ATOM 1159 H17S POPCO 9 -33.875 -23.182 -1.656 1.00 0.00 O1 H

ATOM 1160 C218 POPCO 9 -35.474 -24.018 -2.825 1.00 0.00 O1 C

ATOM 1161 H18R POPCO 9 -35.602 -23.352 -3.696 1.00 0.00 O1 H

ATOM 1162 H18S POPCO 9 -34.770 -24.745 -3.091 1.00 0.00 O1 H

ATOM 1163 H18T POPCO 9 -36.487 -24.473 -2.694 1.00 0.00 O1 H

ATOM 1164 C33 POPCO 9 -38.357 -26.807 16.464 1.00 0.00 O1 C

ATOM 1165 H3X POPCO 9 -38.672 -27.848 16.800 1.00 0.00 O1 H

ATOM 1166 H3Y POPCO 9 -39.342 -26.399 16.140 1.00 0.00 O1 H

ATOM 1167 C34 POPCO 9 -37.343 -26.716 15.357 1.00 0.00 O1 C

ATOM 1168 H4X POPCO 9 -37.301 -25.635 15.040 1.00 0.00 O1 H

ATOM 1169 H4Y POPCO 9 -36.334 -27.046 15.628 1.00 0.00 O1 H

ATOM 1170 C35 POPCO 9 -37.766 -27.580 14.268 1.00 0.00 O1 C

ATOM 1171 H5X POPCO 9 -37.431 -28.637 14.406 1.00 0.00 O1 H

ATOM 1172 H5Y POPCO 9 -38.865 -27.540 14.015 1.00 0.00 O1 H

ATOM 1173 C36 POPCO 9 -37.108 -27.063 12.850 1.00 0.00 O1 C

ATOM 1174 H6X POPCO 9 -37.379 -25.963 12.803 1.00 0.00 O1 H

ATOM 1175 H6Y POPCO 9 -35.944 -27.145 12.956 1.00 0.00 O1 H

ATOM 1176 C37 POPCO 9 -37.651 -27.813 11.635 1.00 0.00 O1 C

ATOM 1177 H7X POPCO 9 -37.300 -28.859 11.781 1.00 0.00 O1 H

ATOM 1178 H7Y POPCO 9 -38.771 -27.645 11.612 1.00 0.00 O1 H

ATOM 1179 C38 POPCO 9 -37.178 -27.228 10.305 1.00 0.00 O1 C

ATOM 1180 H8X POPCO 9 -37.556 -26.162 10.193 1.00 0.00 O1 H

ATOM 1181 H8Y POPCO 9 -36.034 -27.249 10.373 1.00 0.00 O1 H

ATOM 1182 C39 POPCO 9 -37.612 -28.031 9.057 1.00 0.00 O1 C

ATOM 1183 H9X POPCO 9 -37.166 -29.066 9.051 1.00 0.00 O1 H

ATOM 1184 H9Y POPCO 9 -38.727 -28.073 8.982 1.00 0.00 O1 H

ATOM 1185 C310 POPCO 9 -37.130 -27.268 7.783 1.00 0.00 O1 C

ATOM 1186 H10X POPCO 9 -37.644 -26.246 7.719 1.00 0.00 O1 H

ATOM 1187 H10Y POPCO 9 -36.069 -27.269 7.663 1.00 0.00 O1 H

ATOM 1188 C311 POPCO 9 -37.674 -27.889 6.525 1.00 0.00 O1 C

ATOM 1189 H11X POPCO 9 -37.133 -28.823 6.229 1.00 0.00 O1 H

ATOM 1190 H11Y POPCO 9 -38.708 -28.151 6.623 1.00 0.00 O1 H

ATOM 1191 C312 POPCO 9 -37.501 -27.012 5.234 1.00 0.00 O1 C

ATOM 1192 H12X POPCO 9 -38.201 -26.152 5.325 1.00 0.00 O1 H

ATOM 1193 H12Y POPCO 9 -36.484 -26.606 5.047 1.00 0.00 O1 H

ATOM 1194 C313 POPCO 9 -37.875 -27.814 4.014 1.00 0.00 O1 C

ATOM 1195 H13X POPCO 9 -37.473 -28.846 3.914 1.00 0.00 O1 H

ATOM 1196 H13Y POPCO 9 -38.938 -27.973 4.098 1.00 0.00 O1 H

ATOM 1197 C314 POPCO 9 -37.654 -27.111 2.662 1.00 0.00 O1 C

ATOM 1198 H14X POPCO 9 -38.117 -26.101 2.783 1.00 0.00 O1 H

ATOM 1199 H14Y POPCO 9 -36.541 -27.077 2.556 1.00 0.00 O1 H

ATOM 1200 C315 POPCO 9 -38.284 -27.854 1.492 1.00 0.00 O1 C

ATOM 1201 H15X POPCO 9 -37.871 -28.925 1.378 1.00 0.00 O1 H

ATOM 1202 H15Y POPCO 9 -39.287 -28.205 1.789 1.00 0.00 O1 H

ATOM 1203 C316 POPCO 9 -38.316 -27.136 0.141 1.00 0.00 O1 C

ATOM 1204 H16X POPCO 9 -37.281 -26.701 -0.033 1.00 0.00 O1 H

ATOM 1205 H16Y POPCO 9 -38.568 -27.711 -0.719 1.00 0.00 O1 H

ATOM 1206 H16Z POPCO 9 -39.066 -26.348 0.223 1.00 0.00 O1 H

ATOM 1207 N POPCO 10 -27.595 -20.195 25.481 1.00 0.00 O1 N

ATOM 1208 C12 POPCO 10 -27.964 -19.316 24.351 1.00 0.00 O1 C

ATOM 1209 H12A POPCO 10 -27.394 -19.531 23.471 1.00 0.00 O1 H

ATOM 1210 H12B POPCO 10 -27.736 -18.241 24.563 1.00 0.00 O1 H

ATOM 1211 C13 POPCO 10 -26.184 -19.777 25.882 1.00 0.00 O1 C

ATOM 1212 H13A POPCO 10 -25.854 -20.330 26.732 1.00 0.00 O1 H

ATOM 1213 H13B POPCO 10 -26.228 -18.794 26.298 1.00 0.00 O1 H

ATOM 1214 H13C POPCO 10 -25.369 -19.936 25.125 1.00 0.00 O1 H

ATOM 1215 C14 POPCO 10 -28.596 -20.224 26.637 1.00 0.00 O1 C

ATOM 1216 H14A POPCO 10 -29.541 -20.154 26.125 1.00 0.00 O1 H

ATOM 1217 H14B POPCO 10 -28.633 -19.386 27.252 1.00 0.00 O1 H

ATOM 1218 H14C POPCO 10 -28.589 -21.039 27.306 1.00 0.00 O1 H

ATOM 1219 C15 POPCO 10 -27.304 -21.536 24.945 1.00 0.00 O1 C

ATOM 1220 H15A POPCO 10 -28.241 -22.008 24.773 1.00 0.00 O1 H

ATOM 1221 H15B POPCO 10 -26.764 -21.471 23.988 1.00 0.00 O1 H

ATOM 1222 H15C POPCO 10 -26.703 -22.180 25.548 1.00 0.00 O1 H

ATOM 1223 C11 POPCO 10 -29.413 -19.450 23.733 1.00 0.00 O1 C

ATOM 1224 H11A POPCO 10 -30.209 -19.353 24.521 1.00 0.00 O1 H

ATOM 1225 H11B POPCO 10 -29.557 -18.619 22.998 1.00 0.00 O1 H

ATOM 1226 P POPCO 10 -28.966 -20.919 21.703 1.00 0.00 O1 P

ATOM 1227 O13 POPCO 10 -29.236 -22.332 21.303 1.00 0.00 O1 O

ATOM 1228 O14 POPCO 10 -27.548 -20.569 21.848 1.00 0.00 O1 O

ATOM 1229 O12 POPCO 10 -29.633 -20.664 23.096 1.00 0.00 O1 O

ATOM 1230 O11 POPCO 10 -29.580 -19.874 20.696 1.00 0.00 O1 O

ATOM 1231 C1 POPCO 10 -29.146 -19.704 19.334 1.00 0.00 O1 C

ATOM 1232 HA POPCO 10 -29.166 -18.605 19.062 1.00 0.00 O1 H

ATOM 1233 HB POPCO 10 -28.092 -20.078 19.227 1.00 0.00 O1 H

ATOM 1234 C2 POPCO 10 -30.109 -20.385 18.310 1.00 0.00 O1 C

ATOM 1235 HS POPCO 10 -30.050 -21.424 18.629 1.00 0.00 O1 H

ATOM 1236 O21 POPCO 10 -29.687 -20.135 16.962 1.00 0.00 O1 O

ATOM 1237 C21 POPCO 10 -29.903 -21.128 16.008 1.00 0.00 O1 C

ATOM 1238 O22 POPCO 10 -30.337 -22.220 16.330 1.00 0.00 O1 O

ATOM 1239 C22 POPCO 10 -29.564 -20.661 14.597 1.00 0.00 O1 C

ATOM 1240 H2R POPCO 10 -30.083 -19.696 14.424 1.00 0.00 O1 H

ATOM 1241 H2S POPCO 10 -28.434 -20.412 14.562 1.00 0.00 O1 H

ATOM 1242 C3 POPCO 10 -31.610 -20.009 18.431 1.00 0.00 O1 C

ATOM 1243 HX POPCO 10 -32.091 -20.496 17.646 1.00 0.00 O1 H

ATOM 1244 HY POPCO 10 -31.953 -20.596 19.333 1.00 0.00 O1 H

ATOM 1245 O31 POPCO 10 -31.840 -18.613 18.476 1.00 0.00 O1 O

ATOM 1246 C31 POPCO 10 -31.776 -17.930 17.278 1.00 0.00 O1 C

ATOM 1247 O32 POPCO 10 -31.828 -18.482 16.180 1.00 0.00 O1 O

ATOM 1248 C32 POPCO 10 -31.513 -16.455 17.478 1.00 0.00 O1 C

ATOM 1249 H2X POPCO 10 -32.407 -15.879 17.251 1.00 0.00 O1 H

ATOM 1250 H2Y POPCO 10 -31.216 -16.289 18.558 1.00 0.00 O1 H

ATOM 1251 C23 POPCO 10 -29.960 -21.553 13.446 1.00 0.00 O1 C

ATOM 1252 H3R POPCO 10 -29.422 -22.510 13.569 1.00 0.00 O1 H

ATOM 1253 H3S POPCO 10 -31.049 -21.914 13.413 1.00 0.00 O1 H

ATOM 1254 C24 POPCO 10 -29.569 -21.009 12.086 1.00 0.00 O1 C

ATOM 1255 H4R POPCO 10 -30.039 -19.961 11.986 1.00 0.00 O1 H

ATOM 1256 H4S POPCO 10 -28.462 -20.874 11.887 1.00 0.00 O1 H

ATOM 1257 C25 POPCO 10 -30.055 -21.746 10.810 1.00 0.00 O1 C

ATOM 1258 H5R POPCO 10 -29.585 -22.766 10.804 1.00 0.00 O1 H

ATOM 1259 H5S POPCO 10 -31.159 -21.819 10.895 1.00 0.00 O1 H

ATOM 1260 C26 POPCO 10 -29.754 -21.048 9.560 1.00 0.00 O1 C

ATOM 1261 H6R POPCO 10 -29.790 -19.917 9.609 1.00 0.00 O1 H

ATOM 1262 H6S POPCO 10 -28.711 -21.334 9.188 1.00 0.00 O1 H

ATOM 1263 C27 POPCO 10 -30.566 -21.570 8.380 1.00 0.00 O1 C

ATOM 1264 H7R POPCO 10 -30.447 -22.704 8.326 1.00 0.00 O1 H

ATOM 1265 H7S POPCO 10 -31.608 -21.339 8.539 1.00 0.00 O1 H

ATOM 1266 C28 POPCO 10 -30.224 -20.855 7.000 1.00 0.00 O1 C

ATOM 1267 H8R POPCO 10 -30.523 -19.784 7.281 1.00 0.00 O1 H

ATOM 1268 H8S POPCO 10 -29.092 -20.935 6.929 1.00 0.00 O1 H

ATOM 1269 C29 POPCO 10 -31.069 -21.356 5.895 1.00 0.00 O1 C

ATOM 1270 H91 POPCO 10 -32.108 -21.494 6.144 1.00 0.00 O1 H

ATOM 1271 C210 POPCO 10 -30.621 -21.659 4.652 1.00 0.00 O1 C

ATOM 1272 H101 POPCO 10 -31.248 -21.945 3.832 1.00 0.00 O1 H

ATOM 1273 C211 POPCO 10 -29.192 -21.587 4.110 1.00 0.00 O1 C

ATOM 1274 H11R POPCO 10 -28.419 -21.178 4.852 1.00 0.00 O1 H

ATOM 1275 H11S POPCO 10 -28.893 -22.521 3.633 1.00 0.00 O1 H

ATOM 1276 C212 POPCO 10 -29.236 -20.654 2.883 1.00 0.00 O1 C

ATOM 1277 H12R POPCO 10 -29.854 -19.709 3.101 1.00 0.00 O1 H

ATOM 1278 H12S POPCO 10 -28.142 -20.393 2.779 1.00 0.00 O1 H

ATOM 1279 C213 POPCO 10 -29.651 -21.386 1.617 1.00 0.00 O1 C

ATOM 1280 H13R POPCO 10 -29.205 -22.391 1.630 1.00 0.00 O1 H

ATOM 1281 H13S POPCO 10 -30.745 -21.559 1.554 1.00 0.00 O1 H

ATOM 1282 C214 POPCO 10 -29.275 -20.523 0.377 1.00 0.00 O1 C

ATOM 1283 H14R POPCO 10 -29.658 -19.515 0.602 1.00 0.00 O1 H

ATOM 1284 H14S POPCO 10 -28.139 -20.342 0.285 1.00 0.00 O1 H

ATOM 1285 C215 POPCO 10 -29.929 -21.029 -0.996 1.00 0.00 O1 C

ATOM 1286 H15R POPCO 10 -29.672 -22.096 -1.142 1.00 0.00 O1 H

ATOM 1287 H15S POPCO 10 -31.018 -21.013 -0.892 1.00 0.00 O1 H

ATOM 1288 C216 POPCO 10 -29.457 -20.295 -2.378 1.00 0.00 O1 C

ATOM 1289 H16R POPCO 10 -29.813 -19.268 -2.338 1.00 0.00 O1 H

ATOM 1290 H16S POPCO 10 -28.380 -20.333 -2.370 1.00 0.00 O1 H

ATOM 1291 C217 POPCO 10 -30.019 -20.843 -3.633 1.00 0.00 O1 C

ATOM 1292 H17R POPCO 10 -29.712 -21.912 -3.615 1.00 0.00 O1 H

ATOM 1293 H17S POPCO 10 -31.185 -20.986 -3.591 1.00 0.00 O1 H

ATOM 1294 C218 POPCO 10 -29.658 -20.026 -4.921 1.00 0.00 O1 C

ATOM 1295 H18R POPCO 10 -30.139 -20.423 -5.814 1.00 0.00 O1 H

ATOM 1296 H18S POPCO 10 -30.035 -19.016 -4.754 1.00 0.00 O1 H

ATOM 1297 H18T POPCO 10 -28.524 -19.845 -5.097 1.00 0.00 O1 H

ATOM 1298 C33 POPCO 10 -30.510 -16.050 16.428 1.00 0.00 O1 C

ATOM 1299 H3X POPCO 10 -30.802 -16.413 15.399 1.00 0.00 O1 H

ATOM 1300 H3Y POPCO 10 -30.470 -14.964 16.281 1.00 0.00 O1 H

ATOM 1301 C34 POPCO 10 -29.115 -16.687 16.727 1.00 0.00 O1 C

ATOM 1302 H4X POPCO 10 -28.769 -16.085 17.583 1.00 0.00 O1 H

ATOM 1303 H4Y POPCO 10 -29.106 -17.693 17.188 1.00 0.00 O1 H

ATOM 1304 C35 POPCO 10 -28.054 -16.534 15.627 1.00 0.00 O1 C

ATOM 1305 H5X POPCO 10 -28.336 -15.646 15.047 1.00 0.00 O1 H

ATOM 1306 H5Y POPCO 10 -26.974 -16.344 16.037 1.00 0.00 O1 H

ATOM 1307 C36 POPCO 10 -27.873 -17.710 14.663 1.00 0.00 O1 C

ATOM 1308 H6X POPCO 10 -27.227 -18.493 15.097 1.00 0.00 O1 H

ATOM 1309 H6Y POPCO 10 -28.911 -18.130 14.552 1.00 0.00 O1 H

ATOM 1310 C37 POPCO 10 -27.320 -17.280 13.354 1.00 0.00 O1 C

ATOM 1311 H7X POPCO 10 -27.889 -16.376 13.010 1.00 0.00 O1 H

ATOM 1312 H7Y POPCO 10 -26.249 -17.078 13.435 1.00 0.00 O1 H

ATOM 1313 C38 POPCO 10 -27.464 -18.265 12.175 1.00 0.00 O1 C

ATOM 1314 H8X POPCO 10 -26.887 -19.154 12.436 1.00 0.00 O1 H

ATOM 1315 H8Y POPCO 10 -28.514 -18.555 11.980 1.00 0.00 O1 H

ATOM 1316 C39 POPCO 10 -26.990 -17.527 10.917 1.00 0.00 O1 C

ATOM 1317 H9X POPCO 10 -27.438 -16.505 10.974 1.00 0.00 O1 H

ATOM 1318 H9Y POPCO 10 -25.895 -17.328 11.232 1.00 0.00 O1 H

ATOM 1319 C310 POPCO 10 -27.035 -18.230 9.595 1.00 0.00 O1 C

ATOM 1320 H10X POPCO 10 -26.399 -19.078 9.596 1.00 0.00 O1 H

ATOM 1321 H10Y POPCO 10 -28.071 -18.639 9.377 1.00 0.00 O1 H

ATOM 1322 C311 POPCO 10 -26.547 -17.416 8.395 1.00 0.00 O1 C

ATOM 1323 H11X POPCO 10 -26.808 -16.310 8.415 1.00 0.00 O1 H

ATOM 1324 H11Y POPCO 10 -25.426 -17.454 8.420 1.00 0.00 O1 H

ATOM 1325 C312 POPCO 10 -27.014 -18.098 7.112 1.00 0.00 O1 C

ATOM 1326 H12X POPCO 10 -26.553 -19.029 7.090 1.00 0.00 O1 H

ATOM 1327 H12Y POPCO 10 -28.070 -18.207 7.276 1.00 0.00 O1 H

ATOM 1328 C313 POPCO 10 -26.784 -17.429 5.799 1.00 0.00 O1 C

ATOM 1329 H13X POPCO 10 -27.318 -16.406 5.854 1.00 0.00 O1 H

ATOM 1330 H13Y POPCO 10 -25.693 -17.331 5.697 1.00 0.00 O1 H

ATOM 1331 C314 POPCO 10 -27.161 -18.201 4.558 1.00 0.00 O1 C

ATOM 1332 H14X POPCO 10 -26.689 -19.189 4.443 1.00 0.00 O1 H

ATOM 1333 H14Y POPCO 10 -28.263 -18.286 4.561 1.00 0.00 O1 H

ATOM 1334 C315 POPCO 10 -26.861 -17.448 3.261 1.00 0.00 O1 C

ATOM 1335 H15X POPCO 10 -27.375 -16.462 3.425 1.00 0.00 O1 H

ATOM 1336 H15Y POPCO 10 -25.699 -17.249 3.265 1.00 0.00 O1 H

ATOM 1337 C316 POPCO 10 -27.302 -17.919 1.838 1.00 0.00 O1 C

ATOM 1338 H16X POPCO 10 -28.394 -18.024 1.807 1.00 0.00 O1 H

ATOM 1339 H16Y POPCO 10 -26.851 -17.346 1.026 1.00 0.00 O1 H

ATOM 1340 H16Z POPCO 10 -26.864 -18.988 1.752 1.00 0.00 O1 H

ATOM 1341 N POPCO 11 -34.710 -13.292 16.396 1.00 0.00 O1 N

ATOM 1342 C12 POPCO 11 -33.693 -12.954 17.566 1.00 0.00 O1 C

ATOM 1343 H12A POPCO 11 -32.748 -12.786 17.047 1.00 0.00 O1 H

ATOM 1344 H12B POPCO 11 -33.634 -13.873 18.171 1.00 0.00 O1 H

ATOM 1345 C13 POPCO 11 -34.489 -12.258 15.378 1.00 0.00 O1 C

ATOM 1346 H13A POPCO 11 -33.379 -12.163 15.036 1.00 0.00 O1 H

ATOM 1347 H13B POPCO 11 -34.772 -11.290 15.815 1.00 0.00 O1 H

ATOM 1348 H13C POPCO 11 -35.137 -12.351 14.484 1.00 0.00 O1 H

ATOM 1349 C14 POPCO 11 -34.376 -14.671 15.887 1.00 0.00 O1 C

ATOM 1350 H14A POPCO 11 -34.562 -15.311 16.714 1.00 0.00 O1 H

ATOM 1351 H14B POPCO 11 -33.296 -14.846 15.700 1.00 0.00 O1 H

ATOM 1352 H14C POPCO 11 -35.073 -14.921 15.015 1.00 0.00 O1 H

ATOM 1353 C15 POPCO 11 -36.129 -13.225 16.929 1.00 0.00 O1 C

ATOM 1354 H15A POPCO 11 -36.258 -13.914 17.694 1.00 0.00 O1 H

ATOM 1355 H15B POPCO 11 -36.246 -12.226 17.381 1.00 0.00 O1 H

ATOM 1356 H15C POPCO 11 -36.897 -13.338 16.262 1.00 0.00 O1 H

ATOM 1357 C11 POPCO 11 -33.972 -11.694 18.505 1.00 0.00 O1 C

ATOM 1358 H11A POPCO 11 -34.964 -11.938 19.045 1.00 0.00 O1 H

ATOM 1359 H11B POPCO 11 -33.192 -11.606 19.278 1.00 0.00 O1 H

ATOM 1360 P POPCO 11 -35.087 -9.488 17.625 1.00 0.00 O1 P

ATOM 1361 O13 POPCO 11 -35.231 -8.543 18.748 1.00 0.00 O1 O

ATOM 1362 O14 POPCO 11 -36.232 -10.417 17.345 1.00 0.00 O1 O

ATOM 1363 O12 POPCO 11 -33.839 -10.393 17.812 1.00 0.00 O1 O

ATOM 1364 O11 POPCO 11 -34.592 -8.837 16.282 1.00 0.00 O1 O

ATOM 1365 C1 POPCO 11 -35.541 -8.841 15.195 1.00 0.00 O1 C

ATOM 1366 HA POPCO 11 -35.625 -9.909 14.905 1.00 0.00 O1 H

ATOM 1367 HB POPCO 11 -36.493 -8.387 15.471 1.00 0.00 O1 H

ATOM 1368 C2 POPCO 11 -34.877 -8.231 13.964 1.00 0.00 O1 C

ATOM 1369 HS POPCO 11 -34.824 -7.113 14.045 1.00 0.00 O1 H

ATOM 1370 O21 POPCO 11 -35.748 -8.702 12.856 1.00 0.00 O1 O

ATOM 1371 C21 POPCO 11 -35.874 -7.826 11.863 1.00 0.00 O1 C

ATOM 1372 O22 POPCO 11 -35.545 -6.676 11.762 1.00 0.00 O1 O

ATOM 1373 C22 POPCO 11 -36.697 -8.447 10.795 1.00 0.00 O1 C

ATOM 1374 H2R POPCO 11 -36.803 -9.556 11.005 1.00 0.00 O1 H

ATOM 1375 H2S POPCO 11 -37.780 -8.067 10.806 1.00 0.00 O1 H

ATOM 1376 C3 POPCO 11 -33.406 -8.625 13.657 1.00 0.00 O1 C

ATOM 1377 HX POPCO 11 -32.991 -8.108 12.789 1.00 0.00 O1 H

ATOM 1378 HY POPCO 11 -32.805 -8.350 14.489 1.00 0.00 O1 H

ATOM 1379 O31 POPCO 11 -33.350 -10.065 13.427 1.00 0.00 O1 O

ATOM 1380 C31 POPCO 11 -32.175 -10.474 12.817 1.00 0.00 O1 C

ATOM 1381 O32 POPCO 11 -31.362 -9.713 12.283 1.00 0.00 O1 O

ATOM 1382 C32 POPCO 11 -31.962 -11.959 12.831 1.00 0.00 O1 C

ATOM 1383 H2X POPCO 11 -31.368 -12.282 13.748 1.00 0.00 O1 H

ATOM 1384 H2Y POPCO 11 -32.984 -12.404 12.899 1.00 0.00 O1 H

ATOM 1385 C23 POPCO 11 -36.124 -8.129 9.409 1.00 0.00 O1 C

ATOM 1386 H3R POPCO 11 -36.202 -7.082 9.206 1.00 0.00 O1 H

ATOM 1387 H3S POPCO 11 -35.006 -8.414 9.410 1.00 0.00 O1 H

ATOM 1388 C24 POPCO 11 -36.723 -9.034 8.331 1.00 0.00 O1 C

ATOM 1389 H4R POPCO 11 -36.345 -10.000 8.555 1.00 0.00 O1 H

ATOM 1390 H4S POPCO 11 -37.874 -8.978 8.381 1.00 0.00 O1 H

ATOM 1391 C25 POPCO 11 -36.238 -8.592 6.954 1.00 0.00 O1 C

ATOM 1392 H5R POPCO 11 -36.566 -7.539 6.790 1.00 0.00 O1 H

ATOM 1393 H5S POPCO 11 -35.171 -8.539 6.946 1.00 0.00 O1 H

ATOM 1394 C26 POPCO 11 -36.790 -9.386 5.795 1.00 0.00 O1 C

ATOM 1395 H6R POPCO 11 -36.356 -10.387 5.861 1.00 0.00 O1 H

ATOM 1396 H6S POPCO 11 -37.914 -9.338 5.798 1.00 0.00 O1 H

ATOM 1397 C27 POPCO 11 -36.298 -8.803 4.479 1.00 0.00 O1 C

ATOM 1398 H7R POPCO 11 -36.618 -7.732 4.382 1.00 0.00 O1 H

ATOM 1399 H7S POPCO 11 -35.215 -8.805 4.394 1.00 0.00 O1 H

ATOM 1400 C28 POPCO 11 -36.889 -9.538 3.314 1.00 0.00 O1 C

ATOM 1401 H8R POPCO 11 -36.368 -10.510 3.335 1.00 0.00 O1 H

ATOM 1402 H8S POPCO 11 -37.997 -9.759 3.437 1.00 0.00 O1 H

ATOM 1403 C29 POPCO 11 -36.672 -8.810 2.009 1.00 0.00 O1 C

ATOM 1404 H91 POPCO 11 -36.190 -7.795 2.033 1.00 0.00 O1 H

ATOM 1405 C210 POPCO 11 -37.052 -9.213 0.712 1.00 0.00 O1 C

ATOM 1406 H101 POPCO 11 -36.863 -8.574 -0.169 1.00 0.00 O1 H

ATOM 1407 C211 POPCO 11 -37.796 -10.492 0.361 1.00 0.00 O1 C

ATOM 1408 H11R POPCO 11 -37.847 -11.218 1.209 1.00 0.00 O1 H

ATOM 1409 H11S POPCO 11 -38.885 -10.207 0.109 1.00 0.00 O1 H

ATOM 1410 C212 POPCO 11 -37.100 -11.111 -0.942 1.00 0.00 O1 C

ATOM 1411 H12R POPCO 11 -36.038 -11.248 -0.691 1.00 0.00 O1 H

ATOM 1412 H12S POPCO 11 -37.519 -12.123 -1.089 1.00 0.00 O1 H

ATOM 1413 C213 POPCO 11 -37.049 -10.229 -2.138 1.00 0.00 O1 C

ATOM 1414 H13R POPCO 11 -38.140 -9.895 -2.254 1.00 0.00 O1 H

ATOM 1415 H13S POPCO 11 -36.494 -9.284 -1.943 1.00 0.00 O1 H

ATOM 1416 C214 POPCO 11 -36.756 -11.081 -3.428 1.00 0.00 O1 C

ATOM 1417 H14R POPCO 11 -35.663 -11.361 -3.451 1.00 0.00 O1 H

ATOM 1418 H14S POPCO 11 -37.477 -11.929 -3.282 1.00 0.00 O1 H

ATOM 1419 C215 POPCO 11 -37.376 -10.442 -4.732 1.00 0.00 O1 C

ATOM 1420 H15R POPCO 11 -38.484 -10.667 -4.803 1.00 0.00 O1 H

ATOM 1421 H15S POPCO 11 -37.330 -9.320 -4.670 1.00 0.00 O1 H

ATOM 1422 C216 POPCO 11 -36.594 -10.863 -6.038 1.00 0.00 O1 C

ATOM 1423 H16R POPCO 11 -35.625 -10.408 -5.886 1.00 0.00 O1 H

ATOM 1424 H16S POPCO 11 -36.461 -11.948 -6.268 1.00 0.00 O1 H

ATOM 1425 C217 POPCO 11 -37.086 -10.256 -7.336 1.00 0.00 O1 C

ATOM 1426 H17R POPCO 11 -38.147 -10.428 -7.456 1.00 0.00 O1 H

ATOM 1427 H17S POPCO 11 -36.797 -9.186 -7.290 1.00 0.00 O1 H

ATOM 1428 C218 POPCO 11 -36.374 -10.788 -8.596 1.00 0.00 O1 C

ATOM 1429 H18R POPCO 11 -36.815 -10.497 -9.600 1.00 0.00 O1 H

ATOM 1430 H18S POPCO 11 -35.319 -10.427 -8.576 1.00 0.00 O1 H

ATOM 1431 H18T POPCO 11 -36.449 -11.877 -8.598 1.00 0.00 O1 H

ATOM 1432 C33 POPCO 11 -31.278 -12.509 11.485 1.00 0.00 O1 C

ATOM 1433 H3X POPCO 11 -30.249 -12.132 11.255 1.00 0.00 O1 H

ATOM 1434 H3Y POPCO 11 -31.021 -13.542 11.733 1.00 0.00 O1 H

ATOM 1435 C34 POPCO 11 -32.250 -12.674 10.249 1.00 0.00 O1 C

ATOM 1436 H4X POPCO 11 -31.855 -13.422 9.513 1.00 0.00 O1 H

ATOM 1437 H4Y POPCO 11 -33.214 -13.166 10.551 1.00 0.00 O1 H

ATOM 1438 C35 POPCO 11 -32.633 -11.422 9.468 1.00 0.00 O1 C

ATOM 1439 H5X POPCO 11 -32.966 -10.665 10.268 1.00 0.00 O1 H

ATOM 1440 H5Y POPCO 11 -31.829 -11.041 8.821 1.00 0.00 O1 H

ATOM 1441 C36 POPCO 11 -33.908 -11.612 8.523 1.00 0.00 O1 C

ATOM 1442 H6X POPCO 11 -34.781 -11.741 9.238 1.00 0.00 O1 H

ATOM 1443 H6Y POPCO 11 -34.016 -10.591 7.937 1.00 0.00 O1 H

ATOM 1444 C37 POPCO 11 -33.884 -12.834 7.591 1.00 0.00 O1 C

ATOM 1445 H7X POPCO 11 -32.880 -12.889 7.136 1.00 0.00 O1 H

ATOM 1446 H7Y POPCO 11 -34.119 -13.773 8.180 1.00 0.00 O1 H

ATOM 1447 C38 POPCO 11 -34.875 -12.782 6.457 1.00 0.00 O1 C

ATOM 1448 H8X POPCO 11 -35.832 -12.471 6.772 1.00 0.00 O1 H

ATOM 1449 H8Y POPCO 11 -34.568 -11.921 5.846 1.00 0.00 O1 H

ATOM 1450 C39 POPCO 11 -34.956 -13.942 5.572 1.00 0.00 O1 C

ATOM 1451 H9X POPCO 11 -33.942 -14.377 5.474 1.00 0.00 O1 H

ATOM 1452 H9Y POPCO 11 -35.448 -14.859 6.103 1.00 0.00 O1 H

ATOM 1453 C310 POPCO 11 -35.472 -13.608 4.184 1.00 0.00 O1 C

ATOM 1454 H10X POPCO 11 -36.447 -13.289 4.354 1.00 0.00 O1 H

ATOM 1455 H10Y POPCO 11 -34.936 -12.760 3.792 1.00 0.00 O1 H

ATOM 1456 C311 POPCO 11 -35.467 -14.718 3.128 1.00 0.00 O1 C

ATOM 1457 H11X POPCO 11 -34.368 -15.047 3.205 1.00 0.00 O1 H

ATOM 1458 H11Y POPCO 11 -36.199 -15.540 3.319 1.00 0.00 O1 H

ATOM 1459 C312 POPCO 11 -35.683 -14.211 1.703 1.00 0.00 O1 C

ATOM 1460 H12X POPCO 11 -36.706 -13.784 1.704 1.00 0.00 O1 H

ATOM 1461 H12Y POPCO 11 -35.075 -13.350 1.462 1.00 0.00 O1 H

ATOM 1462 C313 POPCO 11 -35.669 -15.363 0.716 1.00 0.00 O1 C

ATOM 1463 H13X POPCO 11 -34.627 -15.795 0.765 1.00 0.00 O1 H

ATOM 1464 H13Y POPCO 11 -36.314 -16.223 0.984 1.00 0.00 O1 H

ATOM 1465 C314 POPCO 11 -35.638 -14.886 -0.769 1.00 0.00 O1 C

ATOM 1466 H14X POPCO 11 -36.601 -15.098 -1.337 1.00 0.00 O1 H

ATOM 1467 H14Y POPCO 11 -35.528 -13.731 -0.727 1.00 0.00 O1 H

ATOM 1468 C315 POPCO 11 -34.518 -15.406 -1.647 1.00 0.00 O1 C

ATOM 1469 H15X POPCO 11 -33.493 -15.016 -1.296 1.00 0.00 O1 H

ATOM 1470 H15Y POPCO 11 -34.443 -16.495 -1.454 1.00 0.00 O1 H

ATOM 1471 C316 POPCO 11 -34.602 -15.134 -3.171 1.00 0.00 O1 C

ATOM 1472 H16X POPCO 11 -34.982 -14.141 -3.366 1.00 0.00 O1 H

ATOM 1473 H16Y POPCO 11 -33.521 -15.351 -3.577 1.00 0.00 O1 H

ATOM 1474 H16Z POPCO 11 -35.401 -15.848 -3.536 1.00 0.00 O1 H

ATOM 1475 N POPCO 12 -32.062 -7.264 19.188 1.00 0.00 O1 N

ATOM 1476 C12 POPCO 12 -32.937 -6.813 17.991 1.00 0.00 O1 C

ATOM 1477 H12A POPCO 12 -33.972 -7.161 18.173 1.00 0.00 O1 H

ATOM 1478 H12B POPCO 12 -32.474 -7.248 17.155 1.00 0.00 O1 H

ATOM 1479 C13 POPCO 12 -32.988 -7.184 20.404 1.00 0.00 O1 C

ATOM 1480 H13A POPCO 12 -33.927 -7.714 20.176 1.00 0.00 O1 H

ATOM 1481 H13B POPCO 12 -33.365 -6.118 20.487 1.00 0.00 O1 H

ATOM 1482 H13C POPCO 12 -32.449 -7.426 21.313 1.00 0.00 O1 H

ATOM 1483 C14 POPCO 12 -31.757 -8.699 19.013 1.00 0.00 O1 C

ATOM 1484 H14A POPCO 12 -31.276 -8.897 18.032 1.00 0.00 O1 H

ATOM 1485 H14B POPCO 12 -32.633 -9.314 19.058 1.00 0.00 O1 H

ATOM 1486 H14C POPCO 12 -31.099 -9.076 19.789 1.00 0.00 O1 H

ATOM 1487 C15 POPCO 12 -30.865 -6.424 19.362 1.00 0.00 O1 C

ATOM 1488 H15A POPCO 12 -30.199 -6.459 18.514 1.00 0.00 O1 H

ATOM 1489 H15B POPCO 12 -31.198 -5.348 19.394 1.00 0.00 O1 H

ATOM 1490 H15C POPCO 12 -30.379 -6.596 20.288 1.00 0.00 O1 H

ATOM 1491 C11 POPCO 12 -33.049 -5.259 17.668 1.00 0.00 O1 C

ATOM 1492 H11A POPCO 12 -32.037 -4.844 17.441 1.00 0.00 O1 H

ATOM 1493 H11B POPCO 12 -33.543 -5.040 16.630 1.00 0.00 O1 H

ATOM 1494 P POPCO 12 -32.952 -3.162 19.219 1.00 0.00 O1 P

ATOM 1495 O13 POPCO 12 -32.026 -3.726 20.169 1.00 0.00 O1 O

ATOM 1496 O14 POPCO 12 -33.924 -2.152 19.745 1.00 0.00 O1 O

ATOM 1497 O12 POPCO 12 -33.671 -4.476 18.719 1.00 0.00 O1 O

ATOM 1498 O11 POPCO 12 -32.261 -2.438 17.945 1.00 0.00 O1 O

ATOM 1499 C1 POPCO 12 -32.892 -2.036 16.757 1.00 0.00 O1 C

ATOM 1500 HA POPCO 12 -33.511 -2.891 16.446 1.00 0.00 O1 H

ATOM 1501 HB POPCO 12 -33.532 -1.177 16.965 1.00 0.00 O1 H

ATOM 1502 C2 POPCO 12 -31.820 -1.641 15.572 1.00 0.00 O1 C

ATOM 1503 HS POPCO 12 -31.672 -0.594 15.931 1.00 0.00 O1 H

ATOM 1504 O21 POPCO 12 -32.546 -1.705 14.323 1.00 0.00 O1 O

ATOM 1505 C21 POPCO 12 -31.942 -1.123 13.252 1.00 0.00 O1 C

ATOM 1506 O22 POPCO 12 -30.962 -0.435 13.373 1.00 0.00 O1 O

ATOM 1507 C22 POPCO 12 -32.708 -1.240 11.880 1.00 0.00 O1 C

ATOM 1508 H2R POPCO 12 -33.070 -2.237 11.708 1.00 0.00 O1 H

ATOM 1509 H2S POPCO 12 -33.575 -0.539 11.837 1.00 0.00 O1 H

ATOM 1510 C3 POPCO 12 -30.505 -2.388 15.576 1.00 0.00 O1 C

ATOM 1511 HX POPCO 12 -29.802 -2.022 14.820 1.00 0.00 O1 H

ATOM 1512 HY POPCO 12 -30.016 -2.228 16.530 1.00 0.00 O1 H

ATOM 1513 O31 POPCO 12 -30.711 -3.814 15.401 1.00 0.00 O1 O

ATOM 1514 C31 POPCO 12 -29.630 -4.526 15.610 1.00 0.00 O1 C

ATOM 1515 O32 POPCO 12 -28.490 -4.032 15.790 1.00 0.00 O1 O

ATOM 1516 C32 POPCO 12 -29.880 -6.067 15.519 1.00 0.00 O1 C

ATOM 1517 H2X POPCO 12 -29.276 -6.659 16.285 1.00 0.00 O1 H

ATOM 1518 H2Y POPCO 12 -31.019 -6.208 15.700 1.00 0.00 O1 H

ATOM 1519 C23 POPCO 12 -31.796 -0.905 10.632 1.00 0.00 O1 C

ATOM 1520 H3R POPCO 12 -31.502 0.201 10.673 1.00 0.00 O1 H

ATOM 1521 H3S POPCO 12 -30.881 -1.564 10.800 1.00 0.00 O1 H

ATOM 1522 C24 POPCO 12 -32.547 -1.303 9.347 1.00 0.00 O1 C

ATOM 1523 H4R POPCO 12 -32.973 -2.266 9.446 1.00 0.00 O1 H

ATOM 1524 H4S POPCO 12 -33.468 -0.656 9.313 1.00 0.00 O1 H

ATOM 1525 C25 POPCO 12 -31.697 -0.947 8.090 1.00 0.00 O1 C

ATOM 1526 H5R POPCO 12 -31.686 0.212 7.916 1.00 0.00 O1 H

ATOM 1527 H5S POPCO 12 -30.698 -1.327 8.296 1.00 0.00 O1 H

ATOM 1528 C26 POPCO 12 -32.134 -1.734 6.854 1.00 0.00 O1 C

ATOM 1529 H6R POPCO 12 -32.196 -2.835 7.180 1.00 0.00 O1 H

ATOM 1530 H6S POPCO 12 -33.158 -1.321 6.627 1.00 0.00 O1 H

ATOM 1531 C27 POPCO 12 -31.289 -1.508 5.619 1.00 0.00 O1 C

ATOM 1532 H7R POPCO 12 -31.447 -0.418 5.351 1.00 0.00 O1 H

ATOM 1533 H7S POPCO 12 -30.245 -1.668 5.795 1.00 0.00 O1 H

ATOM 1534 C28 POPCO 12 -31.814 -2.374 4.428 1.00 0.00 O1 C

ATOM 1535 H8R POPCO 12 -31.984 -3.404 4.606 1.00 0.00 O1 H

ATOM 1536 H8S POPCO 12 -32.868 -1.985 4.259 1.00 0.00 O1 H

ATOM 1537 C29 POPCO 12 -30.898 -2.084 3.239 1.00 0.00 O1 C

ATOM 1538 H91 POPCO 12 -29.819 -2.173 3.424 1.00 0.00 O1 H

ATOM 1539 C210 POPCO 12 -31.274 -2.003 1.963 1.00 0.00 O1 C

ATOM 1540 H101 POPCO 12 -30.609 -1.784 1.096 1.00 0.00 O1 H

ATOM 1541 C211 POPCO 12 -32.692 -2.131 1.474 1.00 0.00 O1 C

ATOM 1542 H11R POPCO 12 -33.245 -2.699 2.273 1.00 0.00 O1 H

ATOM 1543 H11S POPCO 12 -33.090 -1.108 1.304 1.00 0.00 O1 H

ATOM 1544 C212 POPCO 12 -32.891 -2.706 0.043 1.00 0.00 O1 C

ATOM 1545 H12R POPCO 12 -32.276 -3.634 -0.056 1.00 0.00 O1 H

ATOM 1546 H12S POPCO 12 -33.914 -3.077 -0.115 1.00 0.00 O1 H

ATOM 1547 C213 POPCO 12 -32.437 -1.756 -1.101 1.00 0.00 O1 C

ATOM 1548 H13R POPCO 12 -33.068 -0.829 -1.086 1.00 0.00 O1 H

ATOM 1549 H13S POPCO 12 -31.422 -1.521 -0.781 1.00 0.00 O1 H

ATOM 1550 C214 POPCO 12 -32.354 -2.496 -2.468 1.00 0.00 O1 C

ATOM 1551 H14R POPCO 12 -31.618 -3.315 -2.535 1.00 0.00 O1 H

ATOM 1552 H14S POPCO 12 -33.316 -3.075 -2.534 1.00 0.00 O1 H

ATOM 1553 C215 POPCO 12 -32.216 -1.517 -3.661 1.00 0.00 O1 C

ATOM 1554 H15R POPCO 12 -32.983 -0.671 -3.564 1.00 0.00 O1 H

ATOM 1555 H15S POPCO 12 -31.157 -1.166 -3.621 1.00 0.00 O1 H

ATOM 1556 C216 POPCO 12 -32.315 -2.138 -5.055 1.00 0.00 O1 C

ATOM 1557 H16R POPCO 12 -31.303 -2.579 -5.213 1.00 0.00 O1 H

ATOM 1558 H16S POPCO 12 -32.987 -3.083 -5.135 1.00 0.00 O1 H

ATOM 1559 C217 POPCO 12 -32.695 -1.092 -6.093 1.00 0.00 O1 C

ATOM 1560 H17R POPCO 12 -33.721 -0.775 -6.105 1.00 0.00 O1 H

ATOM 1561 H17S POPCO 12 -32.070 -0.165 -5.963 1.00 0.00 O1 H

ATOM 1562 C218 POPCO 12 -32.378 -1.534 -7.488 1.00 0.00 O1 C

ATOM 1563 H18R POPCO 12 -32.643 -0.795 -8.294 1.00 0.00 O1 H

ATOM 1564 H18S POPCO 12 -31.248 -1.612 -7.529 1.00 0.00 O1 H

ATOM 1565 H18T POPCO 12 -32.859 -2.500 -7.800 1.00 0.00 O1 H

ATOM 1566 C33 POPCO 12 -29.619 -6.827 14.175 1.00 0.00 O1 C

ATOM 1567 H3X POPCO 12 -28.549 -7.098 14.027 1.00 0.00 O1 H

ATOM 1568 H3Y POPCO 12 -30.258 -7.691 14.250 1.00 0.00 O1 H

ATOM 1569 C34 POPCO 12 -30.043 -5.921 13.010 1.00 0.00 O1 C

ATOM 1570 H4X POPCO 12 -30.990 -5.464 13.296 1.00 0.00 O1 H

ATOM 1571 H4Y POPCO 12 -29.336 -5.047 12.856 1.00 0.00 O1 H

ATOM 1572 C35 POPCO 12 -30.066 -6.698 11.635 1.00 0.00 O1 C

ATOM 1573 H5X POPCO 12 -29.108 -7.174 11.451 1.00 0.00 O1 H

ATOM 1574 H5Y POPCO 12 -30.777 -7.556 11.789 1.00 0.00 O1 H

ATOM 1575 C36 POPCO 12 -30.366 -5.858 10.420 1.00 0.00 O1 C

ATOM 1576 H6X POPCO 12 -31.291 -5.271 10.569 1.00 0.00 O1 H

ATOM 1577 H6Y POPCO 12 -29.598 -5.114 10.389 1.00 0.00 O1 H

ATOM 1578 C37 POPCO 12 -30.483 -6.716 9.218 1.00 0.00 O1 C

ATOM 1579 H7X POPCO 12 -29.684 -7.465 9.094 1.00 0.00 O1 H

ATOM 1580 H7Y POPCO 12 -31.389 -7.364 9.343 1.00 0.00 O1 H

ATOM 1581 C38 POPCO 12 -30.703 -6.026 7.816 1.00 0.00 O1 C

ATOM 1582 H8X POPCO 12 -31.644 -5.509 7.933 1.00 0.00 O1 H

ATOM 1583 H8Y POPCO 12 -29.867 -5.313 7.744 1.00 0.00 O1 H

ATOM 1584 C39 POPCO 12 -30.922 -6.980 6.640 1.00 0.00 O1 C

ATOM 1585 H9X POPCO 12 -30.638 -8.055 6.807 1.00 0.00 O1 H

ATOM 1586 H9Y POPCO 12 -32.022 -6.895 6.428 1.00 0.00 O1 H

ATOM 1587 C310 POPCO 12 -30.205 -6.524 5.372 1.00 0.00 O1 C

ATOM 1588 H10X POPCO 12 -30.705 -5.513 5.166 1.00 0.00 O1 H

ATOM 1589 H10Y POPCO 12 -29.074 -6.456 5.438 1.00 0.00 O1 H

ATOM 1590 C311 POPCO 12 -30.566 -7.516 4.250 1.00 0.00 O1 C

ATOM 1591 H11X POPCO 12 -30.032 -8.476 4.568 1.00 0.00 O1 H

ATOM 1592 H11Y POPCO 12 -31.648 -7.719 4.391 1.00 0.00 O1 H

ATOM 1593 C312 POPCO 12 -30.166 -7.023 2.865 1.00 0.00 O1 C

ATOM 1594 H12X POPCO 12 -30.317 -5.936 2.937 1.00 0.00 O1 H

ATOM 1595 H12Y POPCO 12 -29.047 -7.118 2.754 1.00 0.00 O1 H

ATOM 1596 C313 POPCO 12 -30.845 -7.595 1.625 1.00 0.00 O1 C

ATOM 1597 H13X POPCO 12 -30.501 -8.648 1.529 1.00 0.00 O1 H

ATOM 1598 H13Y POPCO 12 -31.893 -7.635 1.889 1.00 0.00 O1 H

ATOM 1599 C314 POPCO 12 -30.759 -6.692 0.378 1.00 0.00 O1 C

ATOM 1600 H14X POPCO 12 -31.050 -5.667 0.624 1.00 0.00 O1 H

ATOM 1601 H14Y POPCO 12 -29.662 -6.835 0.158 1.00 0.00 O1 H

ATOM 1602 C315 POPCO 12 -31.714 -7.072 -0.699 1.00 0.00 O1 C

ATOM 1603 H15X POPCO 12 -32.307 -7.923 -0.240 1.00 0.00 O1 H

ATOM 1604 H15Y POPCO 12 -32.447 -6.324 -0.807 1.00 0.00 O1 H

ATOM 1605 C316 POPCO 12 -31.107 -7.552 -2.015 1.00 0.00 O1 C

ATOM 1606 H16X POPCO 12 -30.182 -8.184 -1.925 1.00 0.00 O1 H

ATOM 1607 H16Y POPCO 12 -31.897 -8.184 -2.486 1.00 0.00 O1 H

ATOM 1608 H16Z POPCO 12 -30.988 -6.624 -2.610 1.00 0.00 O1 H

ATOM 1609 N POPCO 13 -28.616 -42.832 16.748 1.00 0.00 O1 N

ATOM 1610 C12 POPCO 13 -29.750 -41.910 17.124 1.00 0.00 O1 C

ATOM 1611 H12A POPCO 13 -29.794 -41.921 18.178 1.00 0.00 O1 H

ATOM 1612 H12B POPCO 13 -30.672 -42.367 16.889 1.00 0.00 O1 H

ATOM 1613 C13 POPCO 13 -27.469 -42.386 17.670 1.00 0.00 O1 C

ATOM 1614 H13A POPCO 13 -27.742 -42.327 18.766 1.00 0.00 O1 H

ATOM 1615 H13B POPCO 13 -27.286 -41.274 17.412 1.00 0.00 O1 H

ATOM 1616 H13C POPCO 13 -26.634 -43.044 17.524 1.00 0.00 O1 H

ATOM 1617 C14 POPCO 13 -28.945 -44.202 17.178 1.00 0.00 O1 C

ATOM 1618 H14A POPCO 13 -29.758 -44.531 16.601 1.00 0.00 O1 H

ATOM 1619 H14B POPCO 13 -29.198 -44.210 18.229 1.00 0.00 O1 H

ATOM 1620 H14C POPCO 13 -28.184 -44.933 16.933 1.00 0.00 O1 H

ATOM 1621 C15 POPCO 13 -28.252 -42.814 15.356 1.00 0.00 O1 C

ATOM 1622 H15A POPCO 13 -29.118 -43.101 14.819 1.00 0.00 O1 H

ATOM 1623 H15B POPCO 13 -27.889 -41.887 14.972 1.00 0.00 O1 H

ATOM 1624 H15C POPCO 13 -27.426 -43.503 15.247 1.00 0.00 O1 H

ATOM 1625 C11 POPCO 13 -29.845 -40.402 16.639 1.00 0.00 O1 C

ATOM 1626 H11A POPCO 13 -30.046 -40.330 15.562 1.00 0.00 O1 H

ATOM 1627 H11B POPCO 13 -30.762 -39.900 17.110 1.00 0.00 O1 H

ATOM 1628 P POPCO 13 -27.795 -38.580 16.287 1.00 0.00 O1 P

ATOM 1629 O13 POPCO 13 -26.709 -38.085 17.239 1.00 0.00 O1 O

ATOM 1630 O14 POPCO 13 -28.588 -37.500 15.789 1.00 0.00 O1 O

ATOM 1631 O12 POPCO 13 -28.708 -39.643 17.089 1.00 0.00 O1 O

ATOM 1632 O11 POPCO 13 -27.158 -39.495 15.174 1.00 0.00 O1 O

ATOM 1633 C1 POPCO 13 -27.793 -39.660 13.903 1.00 0.00 O1 C

ATOM 1634 HA POPCO 13 -28.389 -40.562 13.826 1.00 0.00 O1 H

ATOM 1635 HB POPCO 13 -28.434 -38.780 13.555 1.00 0.00 O1 H

ATOM 1636 C2 POPCO 13 -26.693 -39.842 12.848 1.00 0.00 O1 C

ATOM 1637 HS POPCO 13 -26.041 -38.959 12.979 1.00 0.00 O1 H

ATOM 1638 O21 POPCO 13 -27.367 -39.855 11.539 1.00 0.00 O1 O

ATOM 1639 C21 POPCO 13 -26.623 -39.347 10.540 1.00 0.00 O1 C

ATOM 1640 O22 POPCO 13 -25.566 -38.714 10.649 1.00 0.00 O1 O

ATOM 1641 C22 POPCO 13 -27.310 -39.820 9.224 1.00 0.00 O1 C

ATOM 1642 H2R POPCO 13 -27.648 -40.820 9.334 1.00 0.00 O1 H

ATOM 1643 H2S POPCO 13 -28.215 -39.180 9.085 1.00 0.00 O1 H

ATOM 1644 C3 POPCO 13 -25.885 -41.122 13.051 1.00 0.00 O1 C

ATOM 1645 HX POPCO 13 -25.007 -41.088 12.370 1.00 0.00 O1 H

ATOM 1646 HY POPCO 13 -25.507 -41.033 14.073 1.00 0.00 O1 H

ATOM 1647 O31 POPCO 13 -26.644 -42.311 12.753 1.00 0.00 O1 O

ATOM 1648 C31 POPCO 13 -25.800 -43.410 12.555 1.00 0.00 O1 C

ATOM 1649 O32 POPCO 13 -24.626 -43.445 12.915 1.00 0.00 O1 O

ATOM 1650 C32 POPCO 13 -26.593 -44.590 12.045 1.00 0.00 O1 C

ATOM 1651 H2X POPCO 13 -26.754 -45.259 12.903 1.00 0.00 O1 H

ATOM 1652 H2Y POPCO 13 -27.558 -44.162 11.596 1.00 0.00 O1 H

ATOM 1653 C23 POPCO 13 -26.382 -39.528 7.993 1.00 0.00 O1 C

ATOM 1654 H3R POPCO 13 -26.232 -38.377 7.872 1.00 0.00 O1 H

ATOM 1655 H3S POPCO 13 -25.286 -39.857 8.088 1.00 0.00 O1 H

ATOM 1656 C24 POPCO 13 -26.927 -40.047 6.653 1.00 0.00 O1 C

ATOM 1657 H4R POPCO 13 -27.077 -41.168 6.596 1.00 0.00 O1 H

ATOM 1658 H4S POPCO 13 -27.995 -39.688 6.534 1.00 0.00 O1 H

ATOM 1659 C25 POPCO 13 -26.064 -39.596 5.406 1.00 0.00 O1 C

ATOM 1660 H5R POPCO 13 -25.835 -38.497 5.351 1.00 0.00 O1 H

ATOM 1661 H5S POPCO 13 -25.011 -40.040 5.381 1.00 0.00 O1 H

ATOM 1662 C26 POPCO 13 -26.591 -40.130 4.053 1.00 0.00 O1 C

ATOM 1663 H6R POPCO 13 -26.516 -41.259 3.941 1.00 0.00 O1 H

ATOM 1664 H6S POPCO 13 -27.599 -39.785 3.903 1.00 0.00 O1 H

ATOM 1665 C27 POPCO 13 -25.850 -39.565 2.853 1.00 0.00 O1 C

ATOM 1666 H7R POPCO 13 -26.079 -38.506 2.752 1.00 0.00 O1 H

ATOM 1667 H7S POPCO 13 -24.800 -39.695 2.900 1.00 0.00 O1 H

ATOM 1668 C28 POPCO 13 -26.213 -40.249 1.439 1.00 0.00 O1 C

ATOM 1669 H8R POPCO 13 -26.057 -41.271 1.619 1.00 0.00 O1 H

ATOM 1670 H8S POPCO 13 -27.342 -40.086 1.262 1.00 0.00 O1 H

ATOM 1671 C29 POPCO 13 -25.359 -39.884 0.250 1.00 0.00 O1 C

ATOM 1672 H91 POPCO 13 -24.314 -40.245 0.258 1.00 0.00 O1 H

ATOM 1673 C210 POPCO 13 -25.791 -39.326 -0.910 1.00 0.00 O1 C

ATOM 1674 H101 POPCO 13 -25.006 -39.214 -1.699 1.00 0.00 O1 H

ATOM 1675 C211 POPCO 13 -27.194 -38.847 -1.320 1.00 0.00 O1 C

ATOM 1676 H11R POPCO 13 -27.991 -39.139 -0.578 1.00 0.00 O1 H

ATOM 1677 H11S POPCO 13 -27.180 -37.751 -1.336 1.00 0.00 O1 H

ATOM 1678 C212 POPCO 13 -27.735 -39.218 -2.689 1.00 0.00 O1 C

ATOM 1679 H12R POPCO 13 -27.357 -40.237 -2.971 1.00 0.00 O1 H

ATOM 1680 H12S POPCO 13 -28.834 -39.077 -2.632 1.00 0.00 O1 H

ATOM 1681 C213 POPCO 13 -27.178 -38.375 -3.832 1.00 0.00 O1 C

ATOM 1682 H13R POPCO 13 -27.529 -37.381 -3.619 1.00 0.00 O1 H

ATOM 1683 H13S POPCO 13 -26.036 -38.347 -3.898 1.00 0.00 O1 H

ATOM 1684 C214 POPCO 13 -27.585 -38.735 -5.277 1.00 0.00 O1 C

ATOM 1685 H14R POPCO 13 -27.051 -39.699 -5.610 1.00 0.00 O1 H

ATOM 1686 H14S POPCO 13 -28.699 -38.975 -5.190 1.00 0.00 O1 H

ATOM 1687 C215 POPCO 13 -27.354 -37.759 -6.485 1.00 0.00 O1 C

ATOM 1688 H15R POPCO 13 -27.947 -36.814 -6.270 1.00 0.00 O1 H

ATOM 1689 H15S POPCO 13 -26.260 -37.571 -6.652 1.00 0.00 O1 H

ATOM 1690 C216 POPCO 13 -27.967 -38.267 -7.800 1.00 0.00 O1 C

ATOM 1691 H16R POPCO 13 -27.695 -39.362 -8.016 1.00 0.00 O1 H

ATOM 1692 H16S POPCO 13 -29.019 -38.263 -7.621 1.00 0.00 O1 H

ATOM 1693 C217 POPCO 13 -27.508 -37.429 -8.991 1.00 0.00 O1 C

ATOM 1694 H17R POPCO 13 -27.903 -36.403 -8.913 1.00 0.00 O1 H

ATOM 1695 H17S POPCO 13 -26.392 -37.376 -8.877 1.00 0.00 O1 H

ATOM 1696 C218 POPCO 13 -27.845 -38.107 -10.343 1.00 0.00 O1 C

ATOM 1697 H18R POPCO 13 -27.374 -37.596 -11.220 1.00 0.00 O1 H

ATOM 1698 H18S POPCO 13 -27.471 -39.110 -10.284 1.00 0.00 O1 H

ATOM 1699 H18T POPCO 13 -28.920 -38.198 -10.392 1.00 0.00 O1 H

ATOM 1700 C33 POPCO 13 -25.921 -45.532 10.950 1.00 0.00 O1 C

ATOM 1701 H3X POPCO 13 -24.834 -45.643 11.147 1.00 0.00 O1 H

ATOM 1702 H3Y POPCO 13 -26.450 -46.457 10.909 1.00 0.00 O1 H

ATOM 1703 C34 POPCO 13 -26.026 -44.946 9.519 1.00 0.00 O1 C

ATOM 1704 H4X POPCO 13 -27.107 -44.733 9.141 1.00 0.00 O1 H

ATOM 1705 H4Y POPCO 13 -25.490 -43.890 9.514 1.00 0.00 O1 H

ATOM 1706 C35 POPCO 13 -25.189 -45.915 8.556 1.00 0.00 O1 C

ATOM 1707 H5X POPCO 13 -24.142 -46.020 8.937 1.00 0.00 O1 H

ATOM 1708 H5Y POPCO 13 -25.692 -46.892 8.406 1.00 0.00 O1 H

ATOM 1709 C36 POPCO 13 -24.888 -45.451 7.140 1.00 0.00 O1 C

ATOM 1710 H6X POPCO 13 -24.134 -44.727 7.194 1.00 0.00 O1 H

ATOM 1711 H6Y POPCO 13 -24.373 -46.258 6.592 1.00 0.00 O1 H

ATOM 1712 C37 POPCO 13 -26.182 -45.201 6.397 1.00 0.00 O1 C

ATOM 1713 H7X POPCO 13 -26.831 -46.118 6.347 1.00 0.00 O1 H

ATOM 1714 H7Y POPCO 13 -26.863 -44.500 7.005 1.00 0.00 O1 H

ATOM 1715 C38 POPCO 13 -25.953 -44.537 5.025 1.00 0.00 O1 C

ATOM 1716 H8X POPCO 13 -25.399 -43.526 5.050 1.00 0.00 O1 H

ATOM 1717 H8Y POPCO 13 -25.325 -45.219 4.411 1.00 0.00 O1 H

ATOM 1718 C39 POPCO 13 -27.300 -44.443 4.258 1.00 0.00 O1 C

ATOM 1719 H9X POPCO 13 -27.826 -45.460 4.215 1.00 0.00 O1 H

ATOM 1720 H9Y POPCO 13 -27.955 -43.755 4.851 1.00 0.00 O1 H

ATOM 1721 C310 POPCO 13 -27.077 -43.880 2.831 1.00 0.00 O1 C

ATOM 1722 H10X POPCO 13 -26.817 -42.813 2.852 1.00 0.00 O1 H

ATOM 1723 H10Y POPCO 13 -26.234 -44.416 2.267 1.00 0.00 O1 H

ATOM 1724 C311 POPCO 13 -28.304 -43.756 1.936 1.00 0.00 O1 C

ATOM 1725 H11X POPCO 13 -28.747 -44.748 1.775 1.00 0.00 O1 H

ATOM 1726 H11Y POPCO 13 -28.979 -43.087 2.482 1.00 0.00 O1 H

ATOM 1727 C312 POPCO 13 -27.837 -43.147 0.571 1.00 0.00 O1 C

ATOM 1728 H12X POPCO 13 -27.779 -42.011 0.796 1.00 0.00 O1 H

ATOM 1729 H12Y POPCO 13 -26.819 -43.509 0.352 1.00 0.00 O1 H

ATOM 1730 C313 POPCO 13 -28.907 -43.267 -0.578 1.00 0.00 O1 C

ATOM 1731 H13X POPCO 13 -28.980 -44.370 -0.672 1.00 0.00 O1 H

ATOM 1732 H13Y POPCO 13 -29.870 -42.892 -0.233 1.00 0.00 O1 H

ATOM 1733 C314 POPCO 13 -28.546 -42.786 -2.021 1.00 0.00 O1 C

ATOM 1734 H14X POPCO 13 -28.311 -41.769 -1.983 1.00 0.00 O1 H

ATOM 1735 H14Y POPCO 13 -27.602 -43.297 -2.288 1.00 0.00 O1 H

ATOM 1736 C315 POPCO 13 -29.560 -42.959 -3.059 1.00 0.00 O1 C

ATOM 1737 H15X POPCO 13 -29.761 -44.074 -2.841 1.00 0.00 O1 H

ATOM 1738 H15Y POPCO 13 -30.525 -42.411 -2.881 1.00 0.00 O1 H

ATOM 1739 C316 POPCO 13 -29.165 -42.753 -4.496 1.00 0.00 O1 C

ATOM 1740 H16X POPCO 13 -28.456 -43.528 -4.814 1.00 0.00 O1 H

ATOM 1741 H16Y POPCO 13 -30.094 -42.756 -5.153 1.00 0.00 O1 H

ATOM 1742 H16Z POPCO 13 -28.578 -41.786 -4.469 1.00 0.00 O1 H

ATOM 1743 N POPCO 14 -20.407 -32.203 19.514 1.00 0.00 O1 N

ATOM 1744 C12 POPCO 14 -21.507 -33.231 19.586 1.00 0.00 O1 C

ATOM 1745 H12A POPCO 14 -21.826 -33.246 20.638 1.00 0.00 O1 H

ATOM 1746 H12B POPCO 14 -21.054 -34.208 19.357 1.00 0.00 O1 H

ATOM 1747 C13 POPCO 14 -20.781 -30.948 20.236 1.00 0.00 O1 C

ATOM 1748 H13A POPCO 14 -21.078 -31.202 21.213 1.00 0.00 O1 H

ATOM 1749 H13B POPCO 14 -21.605 -30.430 19.732 1.00 0.00 O1 H

ATOM 1750 H13C POPCO 14 -19.964 -30.243 20.269 1.00 0.00 O1 H

ATOM 1751 C14 POPCO 14 -19.202 -32.705 20.076 1.00 0.00 O1 C

ATOM 1752 H14A POPCO 14 -18.804 -33.595 19.586 1.00 0.00 O1 H

ATOM 1753 H14B POPCO 14 -19.275 -32.957 21.127 1.00 0.00 O1 H

ATOM 1754 H14C POPCO 14 -18.361 -31.934 20.013 1.00 0.00 O1 H

ATOM 1755 C15 POPCO 14 -20.120 -31.909 18.132 1.00 0.00 O1 C

ATOM 1756 H15A POPCO 14 -19.926 -32.801 17.612 1.00 0.00 O1 H

ATOM 1757 H15B POPCO 14 -21.033 -31.569 17.593 1.00 0.00 O1 H

ATOM 1758 H15C POPCO 14 -19.280 -31.217 17.926 1.00 0.00 O1 H

ATOM 1759 C11 POPCO 14 -22.783 -33.171 18.678 1.00 0.00 O1 C

ATOM 1760 H11A POPCO 14 -22.518 -33.327 17.614 1.00 0.00 O1 H

ATOM 1761 H11B POPCO 14 -23.407 -34.065 18.880 1.00 0.00 O1 H

ATOM 1762 P POPCO 14 -24.079 -31.055 17.772 1.00 0.00 O1 P

ATOM 1763 O13 POPCO 14 -24.269 -29.755 18.432 1.00 0.00 O1 O

ATOM 1764 O14 POPCO 14 -25.314 -31.779 17.244 1.00 0.00 O1 O

ATOM 1765 O12 POPCO 14 -23.487 -31.998 18.918 1.00 0.00 O1 O

ATOM 1766 O11 POPCO 14 -22.940 -31.058 16.667 1.00 0.00 O1 O

ATOM 1767 C1 POPCO 14 -23.388 -31.262 15.318 1.00 0.00 O1 C

ATOM 1768 HA POPCO 14 -23.907 -32.256 15.293 1.00 0.00 O1 H

ATOM 1769 HB POPCO 14 -23.986 -30.430 15.092 1.00 0.00 O1 H

ATOM 1770 C2 POPCO 14 -22.170 -31.353 14.379 1.00 0.00 O1 C

ATOM 1771 HS POPCO 14 -21.707 -30.363 14.385 1.00 0.00 O1 H

ATOM 1772 O21 POPCO 14 -22.708 -31.632 13.057 1.00 0.00 O1 O

ATOM 1773 C21 POPCO 14 -21.911 -31.261 12.092 1.00 0.00 O1 C

ATOM 1774 O22 POPCO 14 -20.979 -30.443 12.247 1.00 0.00 O1 O

ATOM 1775 C22 POPCO 14 -22.062 -32.058 10.820 1.00 0.00 O1 C

ATOM 1776 H2R POPCO 14 -21.891 -33.192 10.984 1.00 0.00 O1 H

ATOM 1777 H2S POPCO 14 -23.085 -31.867 10.417 1.00 0.00 O1 H

ATOM 1778 C3 POPCO 14 -21.131 -32.464 14.806 1.00 0.00 O1 C

ATOM 1779 HX POPCO 14 -20.243 -32.286 14.196 1.00 0.00 O1 H

ATOM 1780 HY POPCO 14 -20.845 -32.353 15.871 1.00 0.00 O1 H

ATOM 1781 O31 POPCO 14 -21.642 -33.783 14.612 1.00 0.00 O1 O

ATOM 1782 C31 POPCO 14 -21.084 -34.796 15.409 1.00 0.00 O1 C

ATOM 1783 O32 POPCO 14 -20.182 -34.693 16.178 1.00 0.00 O1 O

ATOM 1784 C32 POPCO 14 -21.976 -36.026 15.160 1.00 0.00 O1 C

ATOM 1785 H2X POPCO 14 -21.898 -36.715 15.980 1.00 0.00 O1 H

ATOM 1786 H2Y POPCO 14 -23.064 -35.760 15.148 1.00 0.00 O1 H

ATOM 1787 C23 POPCO 14 -20.962 -31.613 9.766 1.00 0.00 O1 C

ATOM 1788 H3R POPCO 14 -20.951 -30.509 9.574 1.00 0.00 O1 H

ATOM 1789 H3S POPCO 14 -19.970 -31.930 10.128 1.00 0.00 O1 H

ATOM 1790 C24 POPCO 14 -21.261 -32.305 8.405 1.00 0.00 O1 C

ATOM 1791 H4R POPCO 14 -20.956 -33.359 8.614 1.00 0.00 O1 H

ATOM 1792 H4S POPCO 14 -22.377 -32.174 8.277 1.00 0.00 O1 H

ATOM 1793 C25 POPCO 14 -20.602 -31.681 7.125 1.00 0.00 O1 C

ATOM 1794 H5R POPCO 14 -20.885 -30.657 6.859 1.00 0.00 O1 H

ATOM 1795 H5S POPCO 14 -19.530 -31.731 7.089 1.00 0.00 O1 H

ATOM 1796 C26 POPCO 14 -20.943 -32.439 5.818 1.00 0.00 O1 C

ATOM 1797 H6R POPCO 14 -20.663 -33.459 5.892 1.00 0.00 O1 H

ATOM 1798 H6S POPCO 14 -22.086 -32.655 5.780 1.00 0.00 O1 H

ATOM 1799 C27 POPCO 14 -20.215 -31.840 4.581 1.00 0.00 O1 C

ATOM 1800 H7R POPCO 14 -20.884 -30.990 4.137 1.00 0.00 O1 H

ATOM 1801 H7S POPCO 14 -19.276 -31.393 4.895 1.00 0.00 O1 H

ATOM 1802 C28 POPCO 14 -19.952 -32.948 3.535 1.00 0.00 O1 C

ATOM 1803 H8R POPCO 14 -19.320 -33.714 4.038 1.00 0.00 O1 H

ATOM 1804 H8S POPCO 14 -20.907 -33.447 3.364 1.00 0.00 O1 H

ATOM 1805 C29 POPCO 14 -19.245 -32.283 2.346 1.00 0.00 O1 C

ATOM 1806 H91 POPCO 14 -18.229 -31.977 2.396 1.00 0.00 O1 H

ATOM 1807 C210 POPCO 14 -19.974 -31.887 1.301 1.00 0.00 O1 C

ATOM 1808 H101 POPCO 14 -19.425 -31.363 0.494 1.00 0.00 O1 H

ATOM 1809 C211 POPCO 14 -21.457 -32.002 1.031 1.00 0.00 O1 C

ATOM 1810 H11R POPCO 14 -22.101 -32.702 1.723 1.00 0.00 O1 H

ATOM 1811 H11S POPCO 14 -21.938 -31.026 1.076 1.00 0.00 O1 H

ATOM 1812 C212 POPCO 14 -21.813 -32.577 -0.377 1.00 0.00 O1 C

ATOM 1813 H12R POPCO 14 -21.687 -33.670 -0.392 1.00 0.00 O1 H

ATOM 1814 H12S POPCO 14 -22.945 -32.350 -0.471 1.00 0.00 O1 H

ATOM 1815 C213 POPCO 14 -21.278 -31.947 -1.658 1.00 0.00 O1 C

ATOM 1816 H13R POPCO 14 -21.335 -30.827 -1.662 1.00 0.00 O1 H

ATOM 1817 H13S POPCO 14 -20.226 -32.262 -1.698 1.00 0.00 O1 H

ATOM 1818 C214 POPCO 14 -21.932 -32.601 -2.871 1.00 0.00 O1 C

ATOM 1819 H14R POPCO 14 -21.947 -33.639 -2.668 1.00 0.00 O1 H

ATOM 1820 H14S POPCO 14 -22.950 -32.208 -2.927 1.00 0.00 O1 H

ATOM 1821 C215 POPCO 14 -21.347 -32.249 -4.253 1.00 0.00 O1 C

ATOM 1822 H15R POPCO 14 -21.386 -31.067 -4.226 1.00 0.00 O1 H

ATOM 1823 H15S POPCO 14 -20.310 -32.640 -4.327 1.00 0.00 O1 H

ATOM 1824 C216 POPCO 14 -22.198 -32.817 -5.412 1.00 0.00 O1 C

ATOM 1825 H16R POPCO 14 -22.519 -33.892 -5.299 1.00 0.00 O1 H

ATOM 1826 H16S POPCO 14 -23.092 -32.217 -5.455 1.00 0.00 O1 H

ATOM 1827 C217 POPCO 14 -21.386 -32.666 -6.755 1.00 0.00 O1 C

ATOM 1828 H17R POPCO 14 -20.630 -31.841 -6.480 1.00 0.00 O1 H

ATOM 1829 H17S POPCO 14 -20.800 -33.601 -6.862 1.00 0.00 O1 H

ATOM 1830 C218 POPCO 14 -22.205 -32.434 -8.029 1.00 0.00 O1 C

ATOM 1831 H18R POPCO 14 -21.510 -32.159 -8.885 1.00 0.00 O1 H

ATOM 1832 H18S POPCO 14 -22.783 -33.363 -8.255 1.00 0.00 O1 H

ATOM 1833 H18T POPCO 14 -22.977 -31.618 -7.880 1.00 0.00 O1 H

ATOM 1834 C33 POPCO 14 -21.640 -36.730 13.862 1.00 0.00 O1 C

ATOM 1835 H3X POPCO 14 -20.649 -37.201 13.836 1.00 0.00 O1 H

ATOM 1836 H3Y POPCO 14 -22.330 -37.624 13.801 1.00 0.00 O1 H

ATOM 1837 C34 POPCO 14 -21.848 -35.967 12.529 1.00 0.00 O1 C

ATOM 1838 H4X POPCO 14 -22.602 -35.221 12.768 1.00 0.00 O1 H

ATOM 1839 H4Y POPCO 14 -20.868 -35.360 12.409 1.00 0.00 O1 H

ATOM 1840 C35 POPCO 14 -22.113 -36.718 11.245 1.00 0.00 O1 C

ATOM 1841 H5X POPCO 14 -21.403 -37.495 10.961 1.00 0.00 O1 H

ATOM 1842 H5Y POPCO 14 -23.108 -37.226 11.391 1.00 0.00 O1 H

ATOM 1843 C36 POPCO 14 -22.325 -35.809 9.979 1.00 0.00 O1 C

ATOM 1844 H6X POPCO 14 -22.867 -34.979 10.425 1.00 0.00 O1 H

ATOM 1845 H6Y POPCO 14 -21.282 -35.468 9.634 1.00 0.00 O1 H

ATOM 1846 C37 POPCO 14 -23.093 -36.512 8.812 1.00 0.00 O1 C

ATOM 1847 H7X POPCO 14 -22.444 -37.279 8.332 1.00 0.00 O1 H

ATOM 1848 H7Y POPCO 14 -24.032 -36.989 9.263 1.00 0.00 O1 H

ATOM 1849 C38 POPCO 14 -23.536 -35.509 7.741 1.00 0.00 O1 C

ATOM 1850 H8X POPCO 14 -24.234 -34.818 8.190 1.00 0.00 O1 H

ATOM 1851 H8Y POPCO 14 -22.772 -34.915 7.229 1.00 0.00 O1 H

ATOM 1852 C39 POPCO 14 -24.410 -36.199 6.569 1.00 0.00 O1 C

ATOM 1853 H9X POPCO 14 -23.870 -37.033 6.215 1.00 0.00 O1 H

ATOM 1854 H9Y POPCO 14 -25.343 -36.584 6.941 1.00 0.00 O1 H

ATOM 1855 C310 POPCO 14 -24.647 -35.183 5.350 1.00 0.00 O1 C

ATOM 1856 H10X POPCO 14 -25.260 -34.244 5.591 1.00 0.00 O1 H

ATOM 1857 H10Y POPCO 14 -23.568 -34.912 5.130 1.00 0.00 O1 H

ATOM 1858 C311 POPCO 14 -25.042 -35.953 4.118 1.00 0.00 O1 C

ATOM 1859 H11X POPCO 14 -24.352 -36.775 4.004 1.00 0.00 O1 H

ATOM 1860 H11Y POPCO 14 -26.053 -36.345 3.980 1.00 0.00 O1 H

ATOM 1861 C312 POPCO 14 -24.760 -35.050 2.911 1.00 0.00 O1 C

ATOM 1862 H12X POPCO 14 -25.337 -34.193 3.176 1.00 0.00 O1 H

ATOM 1863 H12Y POPCO 14 -23.768 -34.689 2.915 1.00 0.00 O1 H

ATOM 1864 C313 POPCO 14 -25.149 -35.748 1.630 1.00 0.00 O1 C

ATOM 1865 H13X POPCO 14 -24.774 -36.725 1.709 1.00 0.00 O1 H

ATOM 1866 H13Y POPCO 14 -26.264 -35.771 1.615 1.00 0.00 O1 H

ATOM 1867 C314 POPCO 14 -24.489 -35.187 0.317 1.00 0.00 O1 C

ATOM 1868 H14X POPCO 14 -24.743 -34.155 0.094 1.00 0.00 O1 H

ATOM 1869 H14Y POPCO 14 -23.443 -35.263 0.566 1.00 0.00 O1 H

ATOM 1870 C315 POPCO 14 -25.076 -35.864 -0.925 1.00 0.00 O1 C

ATOM 1871 H15X POPCO 14 -24.929 -37.000 -0.828 1.00 0.00 O1 H

ATOM 1872 H15Y POPCO 14 -26.192 -35.697 -0.912 1.00 0.00 O1 H

ATOM 1873 C316 POPCO 14 -24.516 -35.391 -2.295 1.00 0.00 O1 C

ATOM 1874 H16X POPCO 14 -23.365 -35.574 -2.310 1.00 0.00 O1 H

ATOM 1875 H16Y POPCO 14 -25.068 -35.914 -3.073 1.00 0.00 O1 H

ATOM 1876 H16Z POPCO 14 -24.726 -34.261 -2.487 1.00 0.00 O1 H

ATOM 1877 N POPCO 15 -28.040 -24.722 19.054 1.00 0.00 O1 N

ATOM 1878 C12 POPCO 15 -28.124 -23.326 18.410 1.00 0.00 O1 C

ATOM 1879 H12A POPCO 15 -28.429 -22.517 19.084 1.00 0.00 O1 H

ATOM 1880 H12B POPCO 15 -28.938 -23.319 17.652 1.00 0.00 O1 H

ATOM 1881 C13 POPCO 15 -27.086 -24.610 20.262 1.00 0.00 O1 C

ATOM 1882 H13A POPCO 15 -26.293 -23.994 19.988 1.00 0.00 O1 H

ATOM 1883 H13B POPCO 15 -27.540 -23.991 21.041 1.00 0.00 O1 H

ATOM 1884 H13C POPCO 15 -26.667 -25.575 20.695 1.00 0.00 O1 H

ATOM 1885 C14 POPCO 15 -29.400 -24.985 19.627 1.00 0.00 O1 C

ATOM 1886 H14A POPCO 15 -30.144 -24.972 18.813 1.00 0.00 O1 H

ATOM 1887 H14B POPCO 15 -29.575 -23.988 20.208 1.00 0.00 O1 H

ATOM 1888 H14C POPCO 15 -29.384 -25.776 20.302 1.00 0.00 O1 H

ATOM 1889 C15 POPCO 15 -27.702 -25.782 18.051 1.00 0.00 O1 C

ATOM 1890 H15A POPCO 15 -28.259 -25.681 17.166 1.00 0.00 O1 H

ATOM 1891 H15B POPCO 15 -26.679 -25.651 17.705 1.00 0.00 O1 H

ATOM 1892 H15C POPCO 15 -27.938 -26.767 18.428 1.00 0.00 O1 H

ATOM 1893 C11 POPCO 15 -26.940 -22.773 17.631 1.00 0.00 O1 C

ATOM 1894 H11A POPCO 15 -26.684 -23.523 16.966 1.00 0.00 O1 H

ATOM 1895 H11B POPCO 15 -27.234 -21.833 17.087 1.00 0.00 O1 H

ATOM 1896 P POPCO 15 -24.395 -22.453 18.196 1.00 0.00 O1 P

ATOM 1897 O13 POPCO 15 -23.660 -22.552 19.482 1.00 0.00 O1 O

ATOM 1898 O14 POPCO 15 -24.133 -21.255 17.372 1.00 0.00 O1 O

ATOM 1899 O12 POPCO 15 -25.922 -22.551 18.481 1.00 0.00 O1 O

ATOM 1900 O11 POPCO 15 -24.215 -23.722 17.340 1.00 0.00 O1 O

ATOM 1901 C1 POPCO 15 -24.137 -23.704 15.929 1.00 0.00 O1 C

ATOM 1902 HA POPCO 15 -25.067 -23.825 15.336 1.00 0.00 O1 H

ATOM 1903 HB POPCO 15 -23.786 -22.725 15.554 1.00 0.00 O1 H

ATOM 1904 C2 POPCO 15 -23.312 -24.934 15.425 1.00 0.00 O1 C

ATOM 1905 HS POPCO 15 -22.252 -24.542 15.655 1.00 0.00 O1 H

ATOM 1906 O21 POPCO 15 -23.545 -25.223 14.035 1.00 0.00 O1 O

ATOM 1907 C21 POPCO 15 -22.655 -24.728 13.210 1.00 0.00 O1 C

ATOM 1908 O22 POPCO 15 -21.729 -23.972 13.379 1.00 0.00 O1 O

ATOM 1909 C22 POPCO 15 -23.016 -25.363 11.901 1.00 0.00 O1 C

ATOM 1910 H2R POPCO 15 -22.096 -25.734 11.458 1.00 0.00 O1 H

ATOM 1911 H2S POPCO 15 -23.656 -26.198 12.123 1.00 0.00 O1 H

ATOM 1912 C3 POPCO 15 -23.673 -26.259 16.184 1.00 0.00 O1 C

ATOM 1913 HX POPCO 15 -22.879 -26.959 15.864 1.00 0.00 O1 H

ATOM 1914 HY POPCO 15 -23.714 -26.198 17.264 1.00 0.00 O1 H

ATOM 1915 O31 POPCO 15 -24.901 -26.924 15.895 1.00 0.00 O1 O

ATOM 1916 C31 POPCO 15 -25.062 -27.840 14.971 1.00 0.00 O1 C

ATOM 1917 O32 POPCO 15 -24.189 -28.199 14.182 1.00 0.00 O1 O

ATOM 1918 C32 POPCO 15 -26.465 -28.456 15.147 1.00 0.00 O1 C

ATOM 1919 H2X POPCO 15 -26.405 -29.403 15.561 1.00 0.00 O1 H

ATOM 1920 H2Y POPCO 15 -27.046 -27.835 15.864 1.00 0.00 O1 H

ATOM 1921 C23 POPCO 15 -23.538 -24.330 10.782 1.00 0.00 O1 C

ATOM 1922 H3R POPCO 15 -24.539 -24.004 11.085 1.00 0.00 O1 H

ATOM 1923 H3S POPCO 15 -22.874 -23.448 10.764 1.00 0.00 O1 H

ATOM 1924 C24 POPCO 15 -23.662 -25.051 9.413 1.00 0.00 O1 C

ATOM 1925 H4R POPCO 15 -22.593 -25.469 9.292 1.00 0.00 O1 H

ATOM 1926 H4S POPCO 15 -24.431 -25.874 9.429 1.00 0.00 O1 H

ATOM 1927 C25 POPCO 15 -24.055 -24.012 8.304 1.00 0.00 O1 C

ATOM 1928 H5R POPCO 15 -24.937 -23.383 8.677 1.00 0.00 O1 H

ATOM 1929 H5S POPCO 15 -23.213 -23.305 8.105 1.00 0.00 O1 H

ATOM 1930 C26 POPCO 15 -24.502 -24.680 7.038 1.00 0.00 O1 C

ATOM 1931 H6R POPCO 15 -24.091 -25.740 6.964 1.00 0.00 O1 H

ATOM 1932 H6S POPCO 15 -25.574 -24.565 6.999 1.00 0.00 O1 H

ATOM 1933 C27 POPCO 15 -24.041 -23.930 5.791 1.00 0.00 O1 C

ATOM 1934 H7R POPCO 15 -24.419 -22.932 5.768 1.00 0.00 O1 H

ATOM 1935 H7S POPCO 15 -22.917 -23.793 5.929 1.00 0.00 O1 H

ATOM 1936 C28 POPCO 15 -24.345 -24.806 4.537 1.00 0.00 O1 C

ATOM 1937 H8R POPCO 15 -23.773 -25.717 4.712 1.00 0.00 O1 H

ATOM 1938 H8S POPCO 15 -25.420 -25.135 4.454 1.00 0.00 O1 H

ATOM 1939 C29 POPCO 15 -23.942 -24.084 3.290 1.00 0.00 O1 C

ATOM 1940 H91 POPCO 15 -22.911 -23.896 3.203 1.00 0.00 O1 H

ATOM 1941 C210 POPCO 15 -24.746 -23.415 2.377 1.00 0.00 O1 C

ATOM 1942 H101 POPCO 15 -24.170 -22.848 1.587 1.00 0.00 O1 H

ATOM 1943 C211 POPCO 15 -26.226 -23.319 2.321 1.00 0.00 O1 C

ATOM 1944 H11R POPCO 15 -26.650 -23.796 3.215 1.00 0.00 O1 H

ATOM 1945 H11S POPCO 15 -26.660 -22.326 2.520 1.00 0.00 O1 H

ATOM 1946 C212 POPCO 15 -26.765 -23.894 1.027 1.00 0.00 O1 C

ATOM 1947 H12R POPCO 15 -26.356 -24.890 0.981 1.00 0.00 O1 H

ATOM 1948 H12S POPCO 15 -27.819 -23.997 1.221 1.00 0.00 O1 H

ATOM 1949 C213 POPCO 15 -26.449 -23.226 -0.280 1.00 0.00 O1 C

ATOM 1950 H13R POPCO 15 -26.913 -22.182 -0.328 1.00 0.00 O1 H

ATOM 1951 H13S POPCO 15 -25.289 -23.269 -0.417 1.00 0.00 O1 H

ATOM 1952 C214 POPCO 15 -26.914 -23.999 -1.490 1.00 0.00 O1 C

ATOM 1953 H14R POPCO 15 -26.480 -25.061 -1.581 1.00 0.00 O1 H

ATOM 1954 H14S POPCO 15 -28.030 -24.095 -1.234 1.00 0.00 O1 H

ATOM 1955 C215 POPCO 15 -26.564 -23.381 -2.765 1.00 0.00 O1 C

ATOM 1956 H15R POPCO 15 -27.127 -22.399 -2.907 1.00 0.00 O1 H

ATOM 1957 H15S POPCO 15 -25.465 -23.225 -2.743 1.00 0.00 O1 H

ATOM 1958 C216 POPCO 15 -26.965 -24.309 -3.983 1.00 0.00 O1 C

ATOM 1959 H16R POPCO 15 -26.695 -25.380 -3.842 1.00 0.00 O1 H

ATOM 1960 H16S POPCO 15 -28.092 -24.218 -4.045 1.00 0.00 O1 H

ATOM 1961 C217 POPCO 15 -26.384 -23.750 -5.318 1.00 0.00 O1 C

ATOM 1962 H17R POPCO 15 -26.783 -22.755 -5.450 1.00 0.00 O1 H

ATOM 1963 H17S POPCO 15 -25.242 -23.673 -5.372 1.00 0.00 O1 H

ATOM 1964 C218 POPCO 15 -26.890 -24.453 -6.554 1.00 0.00 O1 C

ATOM 1965 H18R POPCO 15 -26.466 -25.462 -6.614 1.00 0.00 O1 H

ATOM 1966 H18S POPCO 15 -27.985 -24.685 -6.478 1.00 0.00 O1 H

ATOM 1967 H18T POPCO 15 -26.600 -23.952 -7.478 1.00 0.00 O1 H

ATOM 1968 C33 POPCO 15 -27.088 -28.248 13.695 1.00 0.00 O1 C

ATOM 1969 H3X POPCO 15 -26.391 -28.592 12.871 1.00 0.00 O1 H

ATOM 1970 H3Y POPCO 15 -27.966 -28.973 13.421 1.00 0.00 O1 H

ATOM 1971 C34 POPCO 15 -27.448 -26.744 13.470 1.00 0.00 O1 C

ATOM 1972 H4X POPCO 15 -27.965 -26.355 14.376 1.00 0.00 O1 H

ATOM 1973 H4Y POPCO 15 -26.535 -26.125 13.360 1.00 0.00 O1 H

ATOM 1974 C35 POPCO 15 -28.308 -26.411 12.282 1.00 0.00 O1 C

ATOM 1975 H5X POPCO 15 -29.175 -26.980 12.359 1.00 0.00 O1 H

ATOM 1976 H5Y POPCO 15 -28.497 -25.291 12.339 1.00 0.00 O1 H

ATOM 1977 C36 POPCO 15 -27.557 -26.624 10.913 1.00 0.00 O1 C

ATOM 1978 H6X POPCO 15 -26.627 -26.056 10.832 1.00 0.00 O1 H

ATOM 1979 H6Y POPCO 15 -27.185 -27.640 10.949 1.00 0.00 O1 H

ATOM 1980 C37 POPCO 15 -28.487 -26.499 9.758 1.00 0.00 O1 C

ATOM 1981 H7X POPCO 15 -29.291 -27.307 9.748 1.00 0.00 O1 H

ATOM 1982 H7Y POPCO 15 -28.989 -25.522 9.814 1.00 0.00 O1 H

ATOM 1983 C38 POPCO 15 -27.670 -26.519 8.379 1.00 0.00 O1 C

ATOM 1984 H8X POPCO 15 -27.455 -25.532 8.075 1.00 0.00 O1 H

ATOM 1985 H8Y POPCO 15 -26.703 -27.126 8.603 1.00 0.00 O1 H

ATOM 1986 C39 POPCO 15 -28.387 -27.337 7.251 1.00 0.00 O1 C

ATOM 1987 H9X POPCO 15 -28.173 -28.454 7.310 1.00 0.00 O1 H

ATOM 1988 H9Y POPCO 15 -29.436 -27.124 7.373 1.00 0.00 O1 H

ATOM 1989 C310 POPCO 15 -27.982 -26.935 5.896 1.00 0.00 O1 C

ATOM 1990 H10X POPCO 15 -28.289 -25.838 5.767 1.00 0.00 O1 H

ATOM 1991 H10Y POPCO 15 -26.876 -26.851 5.830 1.00 0.00 O1 H

ATOM 1992 C311 POPCO 15 -28.350 -27.870 4.811 1.00 0.00 O1 C

ATOM 1993 H11X POPCO 15 -27.991 -28.961 4.895 1.00 0.00 O1 H

ATOM 1994 H11Y POPCO 15 -29.513 -27.980 4.847 1.00 0.00 O1 H

ATOM 1995 C312 POPCO 15 -27.909 -27.321 3.402 1.00 0.00 O1 C

ATOM 1996 H12X POPCO 15 -28.238 -26.277 3.430 1.00 0.00 O1 H

ATOM 1997 H12Y POPCO 15 -26.766 -27.375 3.290 1.00 0.00 O1 H

ATOM 1998 C313 POPCO 15 -28.513 -27.995 2.251 1.00 0.00 O1 C

ATOM 1999 H13X POPCO 15 -28.539 -29.097 2.484 1.00 0.00 O1 H

ATOM 2000 H13Y POPCO 15 -29.636 -27.750 2.069 1.00 0.00 O1 H

ATOM 2001 C314 POPCO 15 -27.846 -27.574 0.913 1.00 0.00 O1 C

ATOM 2002 H14X POPCO 15 -27.774 -26.451 0.931 1.00 0.00 O1 H

ATOM 2003 H14Y POPCO 15 -26.762 -27.973 0.660 1.00 0.00 O1 H

ATOM 2004 C315 POPCO 15 -28.695 -27.959 -0.370 1.00 0.00 O1 C

ATOM 2005 H15X POPCO 15 -28.974 -28.999 -0.142 1.00 0.00 O1 H

ATOM 2006 H15Y POPCO 15 -29.614 -27.369 -0.597 1.00 0.00 O1 H

ATOM 2007 C316 POPCO 15 -27.874 -27.929 -1.660 1.00 0.00 O1 C

ATOM 2008 H16X POPCO 15 -26.837 -28.371 -1.545 1.00 0.00 O1 H

ATOM 2009 H16Y POPCO 15 -28.350 -28.588 -2.369 1.00 0.00 O1 H

ATOM 2010 H16Z POPCO 15 -27.817 -26.914 -2.060 1.00 0.00 O1 H

ATOM 2011 N POPCO 16 -22.213 -19.448 20.516 1.00 0.00 O1 N

ATOM 2012 C12 POPCO 16 -23.326 -19.357 19.533 1.00 0.00 O1 C

ATOM 2013 H12A POPCO 16 -24.306 -19.509 19.882 1.00 0.00 O1 H

ATOM 2014 H12B POPCO 16 -23.232 -20.118 18.741 1.00 0.00 O1 H

ATOM 2015 C13 POPCO 16 -22.493 -18.692 21.745 1.00 0.00 O1 C

ATOM 2016 H13A POPCO 16 -23.524 -18.701 21.878 1.00 0.00 O1 H

ATOM 2017 H13B POPCO 16 -22.319 -17.589 21.556 1.00 0.00 O1 H

ATOM 2018 H13C POPCO 16 -21.918 -19.040 22.550 1.00 0.00 O1 H

ATOM 2019 C14 POPCO 16 -21.913 -20.891 20.864 1.00 0.00 O1 C

ATOM 2020 H14A POPCO 16 -21.676 -21.482 20.007 1.00 0.00 O1 H

ATOM 2021 H14B POPCO 16 -22.785 -21.364 21.335 1.00 0.00 O1 H

ATOM 2022 H14C POPCO 16 -21.203 -20.873 21.606 1.00 0.00 O1 H

ATOM 2023 C15 POPCO 16 -20.971 -18.905 19.945 1.00 0.00 O1 C

ATOM 2024 H15A POPCO 16 -20.867 -19.129 18.877 1.00 0.00 O1 H

ATOM 2025 H15B POPCO 16 -20.984 -17.767 19.979 1.00 0.00 O1 H

ATOM 2026 H15C POPCO 16 -20.042 -19.298 20.286 1.00 0.00 O1 H

ATOM 2027 C11 POPCO 16 -23.512 -17.957 18.740 1.00 0.00 O1 C

ATOM 2028 H11A POPCO 16 -22.747 -17.798 17.982 1.00 0.00 O1 H

ATOM 2029 H11B POPCO 16 -24.448 -18.200 18.189 1.00 0.00 O1 H

ATOM 2030 P POPCO 16 -23.003 -15.536 19.532 1.00 0.00 O1 P

ATOM 2031 O13 POPCO 16 -22.346 -15.409 20.821 1.00 0.00 O1 O

ATOM 2032 O14 POPCO 16 -24.046 -14.550 19.248 1.00 0.00 O1 O

ATOM 2033 O12 POPCO 16 -23.737 -16.911 19.568 1.00 0.00 O1 O

ATOM 2034 O11 POPCO 16 -21.931 -15.597 18.350 1.00 0.00 O1 O

ATOM 2035 C1 POPCO 16 -22.201 -15.336 17.020 1.00 0.00 O1 C

ATOM 2036 HA POPCO 16 -22.899 -16.054 16.637 1.00 0.00 O1 H

ATOM 2037 HB POPCO 16 -22.663 -14.355 16.914 1.00 0.00 O1 H

ATOM 2038 C2 POPCO 16 -20.965 -15.394 16.103 1.00 0.00 O1 C

ATOM 2039 HS POPCO 16 -20.390 -14.642 16.575 1.00 0.00 O1 H

ATOM 2040 O21 POPCO 16 -21.283 -15.081 14.788 1.00 0.00 O1 O

ATOM 2041 C21 POPCO 16 -20.187 -14.662 14.055 1.00 0.00 O1 C

ATOM 2042 O22 POPCO 16 -19.174 -14.212 14.521 1.00 0.00 O1 O

ATOM 2043 C22 POPCO 16 -20.707 -14.607 12.568 1.00 0.00 O1 C

ATOM 2044 H2R POPCO 16 -21.274 -15.518 12.383 1.00 0.00 O1 H

ATOM 2045 H2S POPCO 16 -21.445 -13.742 12.642 1.00 0.00 O1 H

ATOM 2046 C3 POPCO 16 -20.109 -16.740 16.179 1.00 0.00 O1 C

ATOM 2047 HX POPCO 16 -19.174 -16.560 15.645 1.00 0.00 O1 H

ATOM 2048 HY POPCO 16 -19.979 -16.841 17.289 1.00 0.00 O1 H

ATOM 2049 O31 POPCO 16 -20.755 -17.872 15.617 1.00 0.00 O1 O

ATOM 2050 C31 POPCO 16 -20.787 -18.995 16.253 1.00 0.00 O1 C

ATOM 2051 O32 POPCO 16 -20.109 -19.367 17.231 1.00 0.00 O1 O

ATOM 2052 C32 POPCO 16 -21.777 -19.946 15.683 1.00 0.00 O1 C

ATOM 2053 H2X POPCO 16 -21.967 -20.657 16.466 1.00 0.00 O1 H

ATOM 2054 H2Y POPCO 16 -22.736 -19.422 15.433 1.00 0.00 O1 H

ATOM 2055 C23 POPCO 16 -19.625 -14.380 11.456 1.00 0.00 O1 C

ATOM 2056 H3R POPCO 16 -19.261 -13.334 11.491 1.00 0.00 O1 H

ATOM 2057 H3S POPCO 16 -18.795 -15.134 11.568 1.00 0.00 O1 H

ATOM 2058 C24 POPCO 16 -20.271 -14.679 10.104 1.00 0.00 O1 C

ATOM 2059 H4R POPCO 16 -20.740 -15.655 10.256 1.00 0.00 O1 H

ATOM 2060 H4S POPCO 16 -21.083 -14.000 9.783 1.00 0.00 O1 H

ATOM 2061 C25 POPCO 16 -19.275 -14.714 8.858 1.00 0.00 O1 C

ATOM 2062 H5R POPCO 16 -18.849 -13.714 8.749 1.00 0.00 O1 H

ATOM 2063 H5S POPCO 16 -18.463 -15.410 9.177 1.00 0.00 O1 H

ATOM 2064 C26 POPCO 16 -20.116 -15.085 7.709 1.00 0.00 O1 C

ATOM 2065 H6R POPCO 16 -20.611 -16.040 7.847 1.00 0.00 O1 H

ATOM 2066 H6S POPCO 16 -20.962 -14.417 7.623 1.00 0.00 O1 H

ATOM 2067 C27 POPCO 16 -19.338 -15.163 6.429 1.00 0.00 O1 C

ATOM 2068 H7R POPCO 16 -18.835 -14.244 6.112 1.00 0.00 O1 H

ATOM 2069 H7S POPCO 16 -18.531 -15.917 6.659 1.00 0.00 O1 H

ATOM 2070 C28 POPCO 16 -20.200 -15.738 5.245 1.00 0.00 O1 C

ATOM 2071 H8R POPCO 16 -20.866 -16.594 5.572 1.00 0.00 O1 H

ATOM 2072 H8S POPCO 16 -20.930 -14.947 4.858 1.00 0.00 O1 H

ATOM 2073 C29 POPCO 16 -19.249 -16.218 4.174 1.00 0.00 O1 C

ATOM 2074 H91 POPCO 16 -18.261 -16.608 4.440 1.00 0.00 O1 H

ATOM 2075 C210 POPCO 16 -19.618 -16.084 2.882 1.00 0.00 O1 C

ATOM 2076 H101 POPCO 16 -18.934 -16.391 2.144 1.00 0.00 O1 H

ATOM 2077 C211 POPCO 16 -20.976 -15.507 2.426 1.00 0.00 O1 C

ATOM 2078 H11R POPCO 16 -21.740 -15.569 3.191 1.00 0.00 O1 H

ATOM 2079 H11S POPCO 16 -20.948 -14.433 2.378 1.00 0.00 O1 H

ATOM 2080 C212 POPCO 16 -21.455 -16.071 1.096 1.00 0.00 O1 C

ATOM 2081 H12R POPCO 16 -21.426 -17.079 1.025 1.00 0.00 O1 H

ATOM 2082 H12S POPCO 16 -22.512 -15.706 0.995 1.00 0.00 O1 H

ATOM 2083 C213 POPCO 16 -20.658 -15.494 -0.066 1.00 0.00 O1 C

ATOM 2084 H13R POPCO 16 -20.760 -14.381 -0.091 1.00 0.00 O1 H

ATOM 2085 H13S POPCO 16 -19.593 -15.609 0.020 1.00 0.00 O1 H

ATOM 2086 C214 POPCO 16 -21.222 -16.114 -1.318 1.00 0.00 O1 C

ATOM 2087 H14R POPCO 16 -21.102 -17.212 -1.173 1.00 0.00 O1 H

ATOM 2088 H14S POPCO 16 -22.290 -15.797 -1.375 1.00 0.00 O1 H

ATOM 2089 C215 POPCO 16 -20.559 -15.804 -2.663 1.00 0.00 O1 C

ATOM 2090 H15R POPCO 16 -20.575 -14.652 -2.852 1.00 0.00 O1 H

ATOM 2091 H15S POPCO 16 -19.512 -16.172 -2.485 1.00 0.00 O1 H

ATOM 2092 C216 POPCO 16 -21.060 -16.604 -3.877 1.00 0.00 O1 C

ATOM 2093 H16R POPCO 16 -20.605 -17.611 -3.869 1.00 0.00 O1 H

ATOM 2094 H16S POPCO 16 -22.161 -16.571 -3.915 1.00 0.00 O1 H

ATOM 2095 C217 POPCO 16 -20.442 -16.139 -5.231 1.00 0.00 O1 C

ATOM 2096 H17R POPCO 16 -20.747 -15.091 -5.435 1.00 0.00 O1 H

ATOM 2097 H17S POPCO 16 -19.367 -16.245 -5.140 1.00 0.00 O1 H

ATOM 2098 C218 POPCO 16 -21.029 -16.976 -6.352 1.00 0.00 O1 C

ATOM 2099 H18R POPCO 16 -20.560 -16.676 -7.316 1.00 0.00 O1 H

ATOM 2100 H18S POPCO 16 -20.797 -18.001 -6.111 1.00 0.00 O1 H

ATOM 2101 H18T POPCO 16 -22.105 -16.837 -6.338 1.00 0.00 O1 H

ATOM 2102 C33 POPCO 16 -21.411 -20.764 14.469 1.00 0.00 O1 C

ATOM 2103 H3X POPCO 16 -20.654 -21.581 14.683 1.00 0.00 O1 H

ATOM 2104 H3Y POPCO 16 -22.278 -21.374 14.103 1.00 0.00 O1 H

ATOM 2105 C34 POPCO 16 -20.999 -20.008 13.246 1.00 0.00 O1 C

ATOM 2106 H4X POPCO 16 -21.047 -18.886 13.310 1.00 0.00 O1 H

ATOM 2107 H4Y POPCO 16 -19.903 -20.238 13.020 1.00 0.00 O1 H

ATOM 2108 C35 POPCO 16 -21.732 -20.395 11.926 1.00 0.00 O1 C

ATOM 2109 H5X POPCO 16 -21.696 -21.477 11.875 1.00 0.00 O1 H

ATOM 2110 H5Y POPCO 16 -22.785 -20.175 12.128 1.00 0.00 O1 H

ATOM 2111 C36 POPCO 16 -21.253 -19.676 10.609 1.00 0.00 O1 C

ATOM 2112 H6X POPCO 16 -21.187 -18.589 10.796 1.00 0.00 O1 H

ATOM 2113 H6Y POPCO 16 -20.177 -20.041 10.404 1.00 0.00 O1 H

ATOM 2114 C37 POPCO 16 -22.075 -20.102 9.391 1.00 0.00 O1 C

ATOM 2115 H7X POPCO 16 -21.787 -21.119 9.219 1.00 0.00 O1 H

ATOM 2116 H7Y POPCO 16 -23.155 -20.047 9.616 1.00 0.00 O1 H

ATOM 2117 C38 POPCO 16 -21.709 -19.309 8.162 1.00 0.00 O1 C

ATOM 2118 H8X POPCO 16 -21.881 -18.228 8.370 1.00 0.00 O1 H

ATOM 2119 H8Y POPCO 16 -20.648 -19.603 7.921 1.00 0.00 O1 H

ATOM 2120 C39 POPCO 16 -22.685 -19.784 7.009 1.00 0.00 O1 C

ATOM 2121 H9X POPCO 16 -22.588 -20.789 6.704 1.00 0.00 O1 H

ATOM 2122 H9Y POPCO 16 -23.669 -19.676 7.458 1.00 0.00 O1 H

ATOM 2123 C310 POPCO 16 -22.512 -18.886 5.814 1.00 0.00 O1 C

ATOM 2124 H10X POPCO 16 -22.901 -17.881 6.044 1.00 0.00 O1 H

ATOM 2125 H10Y POPCO 16 -21.427 -18.799 5.535 1.00 0.00 O1 H

ATOM 2126 C311 POPCO 16 -23.301 -19.496 4.638 1.00 0.00 O1 C

ATOM 2127 H11X POPCO 16 -23.031 -20.606 4.496 1.00 0.00 O1 H

ATOM 2128 H11Y POPCO 16 -24.378 -19.439 4.886 1.00 0.00 O1 H

ATOM 2129 C312 POPCO 16 -23.166 -18.750 3.270 1.00 0.00 O1 C

ATOM 2130 H12X POPCO 16 -23.670 -17.726 3.279 1.00 0.00 O1 H

ATOM 2131 H12Y POPCO 16 -22.063 -18.420 3.120 1.00 0.00 O1 H

ATOM 2132 C313 POPCO 16 -23.850 -19.709 2.187 1.00 0.00 O1 C

ATOM 2133 H13X POPCO 16 -23.275 -20.599 1.927 1.00 0.00 O1 H

ATOM 2134 H13Y POPCO 16 -24.826 -19.986 2.624 1.00 0.00 O1 H

ATOM 2135 C314 POPCO 16 -24.157 -18.991 0.877 1.00 0.00 O1 C

ATOM 2136 H14X POPCO 16 -24.817 -18.088 1.087 1.00 0.00 O1 H

ATOM 2137 H14Y POPCO 16 -23.313 -18.495 0.474 1.00 0.00 O1 H

ATOM 2138 C315 POPCO 16 -24.898 -19.954 -0.168 1.00 0.00 O1 C

ATOM 2139 H15X POPCO 16 -24.174 -20.777 -0.510 1.00 0.00 O1 H

ATOM 2140 H15Y POPCO 16 -25.771 -20.503 0.315 1.00 0.00 O1 H

ATOM 2141 C316 POPCO 16 -25.580 -19.117 -1.233 1.00 0.00 O1 C

ATOM 2142 H16X POPCO 16 -24.988 -18.473 -1.920 1.00 0.00 O1 H

ATOM 2143 H16Y POPCO 16 -26.186 -19.792 -1.864 1.00 0.00 O1 H

ATOM 2144 H16Z POPCO 16 -26.282 -18.405 -0.714 1.00 0.00 O1 H

ATOM 2145 N POPCO 17 -27.436 -14.441 19.840 1.00 0.00 O1 N

ATOM 2146 C12 POPCO 17 -26.823 -14.432 18.475 1.00 0.00 O1 C

ATOM 2147 H12A POPCO 17 -27.151 -15.301 17.958 1.00 0.00 O1 H

ATOM 2148 H12B POPCO 17 -25.740 -14.383 18.505 1.00 0.00 O1 H

ATOM 2149 C13 POPCO 17 -28.874 -14.792 19.817 1.00 0.00 O1 C

ATOM 2150 H13A POPCO 17 -28.933 -15.742 19.341 1.00 0.00 O1 H

ATOM 2151 H13B POPCO 17 -29.440 -14.038 19.203 1.00 0.00 O1 H

ATOM 2152 H13C POPCO 17 -29.318 -14.800 20.793 1.00 0.00 O1 H

ATOM 2153 C14 POPCO 17 -26.618 -15.393 20.632 1.00 0.00 O1 C

ATOM 2154 H14A POPCO 17 -25.641 -15.393 20.239 1.00 0.00 O1 H

ATOM 2155 H14B POPCO 17 -27.000 -16.388 20.486 1.00 0.00 O1 H

ATOM 2156 H14C POPCO 17 -26.761 -15.178 21.727 1.00 0.00 O1 H

ATOM 2157 C15 POPCO 17 -27.377 -13.134 20.452 1.00 0.00 O1 C

ATOM 2158 H15A POPCO 17 -26.375 -12.626 20.298 1.00 0.00 O1 H

ATOM 2159 H15B POPCO 17 -28.080 -12.450 19.977 1.00 0.00 O1 H

ATOM 2160 H15C POPCO 17 -27.589 -13.084 21.473 1.00 0.00 O1 H

ATOM 2161 C11 POPCO 17 -27.191 -13.215 17.610 1.00 0.00 O1 C

ATOM 2162 H11A POPCO 17 -26.842 -12.262 18.006 1.00 0.00 O1 H

ATOM 2163 H11B POPCO 17 -26.584 -13.299 16.714 1.00 0.00 O1 H

ATOM 2164 P POPCO 17 -29.521 -11.969 17.465 1.00 0.00 O1 P

ATOM 2165 O13 POPCO 17 -29.523 -11.523 18.867 1.00 0.00 O1 O

ATOM 2166 O14 POPCO 17 -30.830 -12.337 16.803 1.00 0.00 O1 O

ATOM 2167 O12 POPCO 17 -28.582 -13.231 17.274 1.00 0.00 O1 O

ATOM 2168 O11 POPCO 17 -28.779 -10.939 16.543 1.00 0.00 O1 O

ATOM 2169 C1 POPCO 17 -28.942 -11.111 15.089 1.00 0.00 O1 C

ATOM 2170 HA POPCO 17 -28.784 -12.183 14.760 1.00 0.00 O1 H

ATOM 2171 HB POPCO 17 -29.901 -10.646 14.856 1.00 0.00 O1 H

ATOM 2172 C2 POPCO 17 -27.836 -10.391 14.288 1.00 0.00 O1 C

ATOM 2173 HS POPCO 17 -27.789 -9.301 14.635 1.00 0.00 O1 H

ATOM 2174 O21 POPCO 17 -28.276 -10.572 12.949 1.00 0.00 O1 O

ATOM 2175 C21 POPCO 17 -27.676 -9.760 12.058 1.00 0.00 O1 C

ATOM 2176 O22 POPCO 17 -26.812 -8.966 12.210 1.00 0.00 O1 O

ATOM 2177 C22 POPCO 17 -28.251 -10.135 10.696 1.00 0.00 O1 C

ATOM 2178 H2R POPCO 17 -28.456 -11.280 10.759 1.00 0.00 O1 H

ATOM 2179 H2S POPCO 17 -29.184 -9.571 10.634 1.00 0.00 O1 H

ATOM 2180 C3 POPCO 17 -26.404 -10.958 14.585 1.00 0.00 O1 C

ATOM 2181 HX POPCO 17 -25.627 -10.324 14.061 1.00 0.00 O1 H

ATOM 2182 HY POPCO 17 -26.196 -11.044 15.680 1.00 0.00 O1 H

ATOM 2183 O31 POPCO 17 -26.325 -12.290 14.145 1.00 0.00 O1 O

ATOM 2184 C31 POPCO 17 -25.159 -12.902 14.236 1.00 0.00 O1 C

ATOM 2185 O32 POPCO 17 -24.113 -12.425 14.554 1.00 0.00 O1 O

ATOM 2186 C32 POPCO 17 -25.187 -14.392 13.957 1.00 0.00 O1 C

ATOM 2187 H2X POPCO 17 -24.629 -14.746 14.790 1.00 0.00 O1 H

ATOM 2188 H2Y POPCO 17 -26.291 -14.731 13.933 1.00 0.00 O1 H

ATOM 2189 C23 POPCO 17 -27.351 -10.134 9.489 1.00 0.00 O1 C

ATOM 2190 H3R POPCO 17 -26.986 -9.100 9.337 1.00 0.00 O1 H

ATOM 2191 H3S POPCO 17 -26.347 -10.761 9.769 1.00 0.00 O1 H

ATOM 2192 C24 POPCO 17 -27.917 -10.844 8.212 1.00 0.00 O1 C

ATOM 2193 H4R POPCO 17 -27.807 -11.896 8.277 1.00 0.00 O1 H

ATOM 2194 H4S POPCO 17 -28.946 -10.595 8.166 1.00 0.00 O1 H

ATOM 2195 C25 POPCO 17 -27.379 -10.309 6.917 1.00 0.00 O1 C

ATOM 2196 H5R POPCO 17 -27.545 -9.222 6.910 1.00 0.00 O1 H

ATOM 2197 H5S POPCO 17 -26.257 -10.443 6.906 1.00 0.00 O1 H

ATOM 2198 C26 POPCO 17 -28.129 -10.896 5.692 1.00 0.00 O1 C

ATOM 2199 H6R POPCO 17 -28.004 -11.964 5.608 1.00 0.00 O1 H

ATOM 2200 H6S POPCO 17 -29.237 -10.711 5.777 1.00 0.00 O1 H

ATOM 2201 C27 POPCO 17 -27.520 -10.248 4.479 1.00 0.00 O1 C

ATOM 2202 H7R POPCO 17 -27.455 -9.110 4.585 1.00 0.00 O1 H

ATOM 2203 H7S POPCO 17 -26.461 -10.640 4.462 1.00 0.00 O1 H

ATOM 2204 C28 POPCO 17 -28.180 -10.608 3.109 1.00 0.00 O1 C

ATOM 2205 H8R POPCO 17 -28.152 -11.674 2.895 1.00 0.00 O1 H

ATOM 2206 H8S POPCO 17 -29.274 -10.448 3.023 1.00 0.00 O1 H

ATOM 2207 C29 POPCO 17 -27.563 -10.017 1.878 1.00 0.00 O1 C

ATOM 2208 H91 POPCO 17 -27.029 -9.130 2.047 1.00 0.00 O1 H

ATOM 2209 C210 POPCO 17 -27.848 -10.313 0.608 1.00 0.00 O1 C

ATOM 2210 H101 POPCO 17 -27.246 -9.699 -0.139 1.00 0.00 O1 H

ATOM 2211 C211 POPCO 17 -28.721 -11.361 0.091 1.00 0.00 O1 C

ATOM 2212 H11R POPCO 17 -28.838 -12.216 0.837 1.00 0.00 O1 H

ATOM 2213 H11S POPCO 17 -29.743 -10.971 0.032 1.00 0.00 O1 H

ATOM 2214 C212 POPCO 17 -28.226 -11.978 -1.224 1.00 0.00 O1 C

ATOM 2215 H12R POPCO 17 -27.135 -12.338 -1.027 1.00 0.00 O1 H

ATOM 2216 H12S POPCO 17 -28.907 -12.815 -1.567 1.00 0.00 O1 H

ATOM 2217 C213 POPCO 17 -28.242 -11.094 -2.432 1.00 0.00 O1 C

ATOM 2218 H13R POPCO 17 -29.230 -10.644 -2.425 1.00 0.00 O1 H

ATOM 2219 H13S POPCO 17 -27.565 -10.223 -2.260 1.00 0.00 O1 H

ATOM 2220 C214 POPCO 17 -27.921 -11.853 -3.637 1.00 0.00 O1 C

ATOM 2221 H14R POPCO 17 -26.896 -12.359 -3.661 1.00 0.00 O1 H

ATOM 2222 H14S POPCO 17 -28.607 -12.754 -3.735 1.00 0.00 O1 H

ATOM 2223 C215 POPCO 17 -28.026 -11.097 -4.981 1.00 0.00 O1 C

ATOM 2224 H15R POPCO 17 -29.071 -10.852 -5.106 1.00 0.00 O1 H

ATOM 2225 H15S POPCO 17 -27.455 -10.144 -4.887 1.00 0.00 O1 H

ATOM 2226 C216 POPCO 17 -27.512 -11.942 -6.158 1.00 0.00 O1 C

ATOM 2227 H16R POPCO 17 -26.381 -11.987 -6.276 1.00 0.00 O1 H

ATOM 2228 H16S POPCO 17 -28.033 -13.005 -6.190 1.00 0.00 O1 H

ATOM 2229 C217 POPCO 17 -27.986 -11.259 -7.455 1.00 0.00 O1 C

ATOM 2230 H17R POPCO 17 -29.124 -11.184 -7.323 1.00 0.00 O1 H

ATOM 2231 H17S POPCO 17 -27.668 -10.217 -7.497 1.00 0.00 O1 H

ATOM 2232 C218 POPCO 17 -27.562 -12.106 -8.694 1.00 0.00 O1 C

ATOM 2233 H18R POPCO 17 -27.775 -11.563 -9.624 1.00 0.00 O1 H

ATOM 2234 H18S POPCO 17 -26.488 -12.435 -8.606 1.00 0.00 O1 H

ATOM 2235 H18T POPCO 17 -28.188 -13.009 -8.781 1.00 0.00 O1 H

ATOM 2236 C33 POPCO 17 -24.577 -14.660 12.550 1.00 0.00 O1 C

ATOM 2237 H3X POPCO 17 -23.500 -14.618 12.653 1.00 0.00 O1 H

ATOM 2238 H3Y POPCO 17 -24.739 -15.740 12.237 1.00 0.00 O1 H

ATOM 2239 C34 POPCO 17 -25.143 -13.774 11.398 1.00 0.00 O1 C

ATOM 2240 H4X POPCO 17 -26.268 -13.946 11.447 1.00 0.00 O1 H

ATOM 2241 H4Y POPCO 17 -24.965 -12.689 11.526 1.00 0.00 O1 H

ATOM 2242 C35 POPCO 17 -24.635 -14.203 10.008 1.00 0.00 O1 C

ATOM 2243 H5X POPCO 17 -23.549 -14.020 10.037 1.00 0.00 O1 H

ATOM 2244 H5Y POPCO 17 -24.646 -15.319 9.857 1.00 0.00 O1 H

ATOM 2245 C36 POPCO 17 -25.273 -13.531 8.798 1.00 0.00 O1 C

ATOM 2246 H6X POPCO 17 -26.388 -13.639 8.882 1.00 0.00 O1 H

ATOM 2247 H6Y POPCO 17 -25.128 -12.501 8.904 1.00 0.00 O1 H

ATOM 2248 C37 POPCO 17 -24.715 -14.097 7.474 1.00 0.00 O1 C

ATOM 2249 H7X POPCO 17 -23.687 -13.768 7.247 1.00 0.00 O1 H

ATOM 2250 H7Y POPCO 17 -24.831 -15.238 7.526 1.00 0.00 O1 H

ATOM 2251 C38 POPCO 17 -25.344 -13.630 6.208 1.00 0.00 O1 C

ATOM 2252 H8X POPCO 17 -26.376 -13.944 6.226 1.00 0.00 O1 H

ATOM 2253 H8Y POPCO 17 -25.310 -12.506 6.202 1.00 0.00 O1 H

ATOM 2254 C39 POPCO 17 -24.760 -14.157 4.888 1.00 0.00 O1 C

ATOM 2255 H9X POPCO 17 -23.852 -13.560 4.603 1.00 0.00 O1 H

ATOM 2256 H9Y POPCO 17 -24.528 -15.178 4.971 1.00 0.00 O1 H

ATOM 2257 C310 POPCO 17 -25.689 -14.142 3.602 1.00 0.00 O1 C

ATOM 2258 H10X POPCO 17 -26.550 -14.919 3.737 1.00 0.00 O1 H

ATOM 2259 H10Y POPCO 17 -26.079 -13.129 3.558 1.00 0.00 O1 H

ATOM 2260 C311 POPCO 17 -24.979 -14.569 2.319 1.00 0.00 O1 C

ATOM 2261 H11X POPCO 17 -24.098 -13.914 2.086 1.00 0.00 O1 H

ATOM 2262 H11Y POPCO 17 -24.650 -15.665 2.413 1.00 0.00 O1 H

ATOM 2263 C312 POPCO 17 -25.876 -14.481 1.105 1.00 0.00 O1 C

ATOM 2264 H12X POPCO 17 -26.761 -15.146 1.304 1.00 0.00 O1 H

ATOM 2265 H12Y POPCO 17 -26.311 -13.453 0.900 1.00 0.00 O1 H

ATOM 2266 C313 POPCO 17 -25.118 -14.868 -0.194 1.00 0.00 O1 C

ATOM 2267 H13X POPCO 17 -24.394 -14.131 -0.571 1.00 0.00 O1 H

ATOM 2268 H13Y POPCO 17 -24.478 -15.795 -0.092 1.00 0.00 O1 H

ATOM 2269 C314 POPCO 17 -26.147 -15.210 -1.284 1.00 0.00 O1 C

ATOM 2270 H14X POPCO 17 -26.737 -16.012 -0.903 1.00 0.00 O1 H

ATOM 2271 H14Y POPCO 17 -26.792 -14.373 -1.489 1.00 0.00 O1 H

ATOM 2272 C315 POPCO 17 -25.485 -15.726 -2.569 1.00 0.00 O1 C

ATOM 2273 H15X POPCO 17 -24.662 -15.125 -2.748 1.00 0.00 O1 H

ATOM 2274 H15Y POPCO 17 -25.067 -16.700 -2.423 1.00 0.00 O1 H

ATOM 2275 C316 POPCO 17 -26.427 -15.803 -3.806 1.00 0.00 O1 C

ATOM 2276 H16X POPCO 17 -26.628 -14.777 -4.175 1.00 0.00 O1 H

ATOM 2277 H16Y POPCO 17 -25.956 -16.394 -4.608 1.00 0.00 O1 H

ATOM 2278 H16Z POPCO 17 -27.349 -16.363 -3.627 1.00 0.00 O1 H

ATOM 2279 N POPCO 18 -20.012 -0.301 22.912 1.00 0.00 O1 N

ATOM 2280 C12 POPCO 18 -20.010 -1.036 21.640 1.00 0.00 O1 C

ATOM 2281 H12A POPCO 18 -18.959 -0.967 21.436 1.00 0.00 O1 H

ATOM 2282 H12B POPCO 18 -20.510 -0.534 20.816 1.00 0.00 O1 H

ATOM 2283 C13 POPCO 18 -18.795 -0.964 23.643 1.00 0.00 O1 C

ATOM 2284 H13A POPCO 18 -17.935 -1.164 22.983 1.00 0.00 O1 H

ATOM 2285 H13B POPCO 18 -19.007 -1.892 24.088 1.00 0.00 O1 H

ATOM 2286 H13C POPCO 18 -18.346 -0.288 24.402 1.00 0.00 O1 H

ATOM 2287 C14 POPCO 18 -19.731 1.155 22.667 1.00 0.00 O1 C

ATOM 2288 H14A POPCO 18 -20.368 1.651 22.012 1.00 0.00 O1 H

ATOM 2289 H14B POPCO 18 -18.736 1.161 22.205 1.00 0.00 O1 H

ATOM 2290 H14C POPCO 18 -19.718 1.653 23.612 1.00 0.00 O1 H

ATOM 2291 C15 POPCO 18 -21.317 -0.448 23.715 1.00 0.00 O1 C

ATOM 2292 H15A POPCO 18 -22.085 0.066 23.207 1.00 0.00 O1 H

ATOM 2293 H15B POPCO 18 -21.676 -1.465 23.768 1.00 0.00 O1 H

ATOM 2294 H15C POPCO 18 -21.304 0.051 24.683 1.00 0.00 O1 H

ATOM 2295 C11 POPCO 18 -20.296 -2.615 21.619 1.00 0.00 O1 C

ATOM 2296 H11A POPCO 18 -20.490 -3.116 22.584 1.00 0.00 O1 H

ATOM 2297 H11B POPCO 18 -21.133 -2.847 21.014 1.00 0.00 O1 H

ATOM 2298 P POPCO 18 -17.726 -3.074 21.211 1.00 0.00 O1 P

ATOM 2299 O13 POPCO 18 -17.360 -3.834 22.381 1.00 0.00 O1 O

ATOM 2300 O14 POPCO 18 -17.276 -1.667 21.222 1.00 0.00 O1 O

ATOM 2301 O12 POPCO 18 -19.225 -3.264 20.895 1.00 0.00 O1 O

ATOM 2302 O11 POPCO 18 -16.998 -3.723 19.944 1.00 0.00 O1 O

ATOM 2303 C1 POPCO 18 -17.232 -3.319 18.615 1.00 0.00 O1 C

ATOM 2304 HA POPCO 18 -18.032 -2.583 18.526 1.00 0.00 O1 H

ATOM 2305 HB POPCO 18 -16.352 -2.869 18.146 1.00 0.00 O1 H

ATOM 2306 C2 POPCO 18 -17.636 -4.519 17.686 1.00 0.00 O1 C

ATOM 2307 HS POPCO 18 -16.821 -5.220 17.585 1.00 0.00 O1 H

ATOM 2308 O21 POPCO 18 -17.748 -3.960 16.345 1.00 0.00 O1 O

ATOM 2309 C21 POPCO 18 -17.993 -4.726 15.290 1.00 0.00 O1 C

ATOM 2310 O22 POPCO 18 -18.012 -5.938 15.271 1.00 0.00 O1 O

ATOM 2311 C22 POPCO 18 -17.931 -3.911 14.012 1.00 0.00 O1 C

ATOM 2312 H2R POPCO 18 -18.693 -3.084 14.074 1.00 0.00 O1 H

ATOM 2313 H2S POPCO 18 -16.902 -3.493 13.971 1.00 0.00 O1 H

ATOM 2314 C3 POPCO 18 -18.868 -5.245 18.317 1.00 0.00 O1 C

ATOM 2315 HX POPCO 18 -18.785 -6.251 17.900 1.00 0.00 O1 H

ATOM 2316 HY POPCO 18 -18.710 -5.392 19.484 1.00 0.00 O1 H

ATOM 2317 O31 POPCO 18 -20.136 -4.597 18.095 1.00 0.00 O1 O

ATOM 2318 C31 POPCO 18 -21.272 -4.880 18.844 1.00 0.00 O1 C

ATOM 2319 O32 POPCO 18 -21.373 -5.648 19.749 1.00 0.00 O1 O

ATOM 2320 C32 POPCO 18 -22.394 -3.929 18.402 1.00 0.00 O1 C

ATOM 2321 H2X POPCO 18 -23.115 -3.980 19.260 1.00 0.00 O1 H

ATOM 2322 H2Y POPCO 18 -22.032 -2.873 18.295 1.00 0.00 O1 H

ATOM 2323 C23 POPCO 18 -18.113 -4.872 12.784 1.00 0.00 O1 C

ATOM 2324 H3R POPCO 18 -17.378 -5.753 12.690 1.00 0.00 O1 H

ATOM 2325 H3S POPCO 18 -19.084 -5.434 12.877 1.00 0.00 O1 H

ATOM 2326 C24 POPCO 18 -18.242 -4.097 11.453 1.00 0.00 O1 C

ATOM 2327 H4R POPCO 18 -19.149 -3.388 11.497 1.00 0.00 O1 H

ATOM 2328 H4S POPCO 18 -17.316 -3.413 11.287 1.00 0.00 O1 H

ATOM 2329 C25 POPCO 18 -18.497 -5.052 10.318 1.00 0.00 O1 C

ATOM 2330 H5R POPCO 18 -17.790 -5.892 10.239 1.00 0.00 O1 H

ATOM 2331 H5S POPCO 18 -19.568 -5.490 10.390 1.00 0.00 O1 H

ATOM 2332 C26 POPCO 18 -18.542 -4.216 9.041 1.00 0.00 O1 C

ATOM 2333 H6R POPCO 18 -19.156 -3.340 9.161 1.00 0.00 O1 H

ATOM 2334 H6S POPCO 18 -17.523 -3.808 8.898 1.00 0.00 O1 H

ATOM 2335 C27 POPCO 18 -18.956 -4.847 7.719 1.00 0.00 O1 C

ATOM 2336 H7R POPCO 18 -18.396 -5.792 7.632 1.00 0.00 O1 H

ATOM 2337 H7S POPCO 18 -20.046 -4.946 7.778 1.00 0.00 O1 H

ATOM 2338 C28 POPCO 18 -18.549 -4.000 6.513 1.00 0.00 O1 C

ATOM 2339 H8R POPCO 18 -19.149 -3.075 6.478 1.00 0.00 O1 H

ATOM 2340 H8S POPCO 18 -17.544 -3.685 6.615 1.00 0.00 O1 H

ATOM 2341 C29 POPCO 18 -18.646 -4.806 5.223 1.00 0.00 O1 C

ATOM 2342 H91 POPCO 18 -18.610 -5.908 5.490 1.00 0.00 O1 H

ATOM 2343 C210 POPCO 18 -18.620 -4.371 3.981 1.00 0.00 O1 C

ATOM 2344 H101 POPCO 18 -18.707 -5.164 3.178 1.00 0.00 O1 H

ATOM 2345 C211 POPCO 18 -18.377 -2.863 3.625 1.00 0.00 O1 C

ATOM 2346 H11R POPCO 18 -18.673 -2.223 4.456 1.00 0.00 O1 H

ATOM 2347 H11S POPCO 18 -17.245 -2.760 3.562 1.00 0.00 O1 H

ATOM 2348 C212 POPCO 18 -19.084 -2.306 2.443 1.00 0.00 O1 C

ATOM 2349 H12R POPCO 18 -20.223 -2.363 2.555 1.00 0.00 O1 H

ATOM 2350 H12S POPCO 18 -18.752 -1.294 2.403 1.00 0.00 O1 H

ATOM 2351 C213 POPCO 18 -18.713 -3.041 1.084 1.00 0.00 O1 C

ATOM 2352 H13R POPCO 18 -17.607 -3.288 1.222 1.00 0.00 O1 H

ATOM 2353 H13S POPCO 18 -19.212 -4.035 1.010 1.00 0.00 O1 H

ATOM 2354 C214 POPCO 18 -19.104 -2.374 -0.250 1.00 0.00 O1 C

ATOM 2355 H14R POPCO 18 -20.223 -2.397 -0.345 1.00 0.00 O1 H

ATOM 2356 H14S POPCO 18 -18.797 -1.278 -0.171 1.00 0.00 O1 H

ATOM 2357 C215 POPCO 18 -18.435 -2.982 -1.495 1.00 0.00 O1 C

ATOM 2358 H15R POPCO 18 -17.350 -3.063 -1.389 1.00 0.00 O1 H

ATOM 2359 H15S POPCO 18 -18.749 -4.057 -1.458 1.00 0.00 O1 H

ATOM 2360 C216 POPCO 18 -18.997 -2.289 -2.680 1.00 0.00 O1 C

ATOM 2361 H16R POPCO 18 -20.071 -2.239 -2.653 1.00 0.00 O1 H

ATOM 2362 H16S POPCO 18 -18.652 -1.215 -2.684 1.00 0.00 O1 H

ATOM 2363 C217 POPCO 18 -18.620 -2.999 -4.027 1.00 0.00 O1 C

ATOM 2364 H17R POPCO 18 -17.613 -2.936 -4.225 1.00 0.00 O1 H

ATOM 2365 H17S POPCO 18 -18.959 -4.063 -4.106 1.00 0.00 O1 H

ATOM 2366 C218 POPCO 18 -19.319 -2.275 -5.256 1.00 0.00 O1 C

ATOM 2367 H18R POPCO 18 -18.734 -2.401 -6.271 1.00 0.00 O1 H

ATOM 2368 H18S POPCO 18 -20.384 -2.586 -5.285 1.00 0.00 O1 H

ATOM 2369 H18T POPCO 18 -19.466 -1.207 -5.152 1.00 0.00 O1 H

ATOM 2370 C33 POPCO 18 -23.034 -4.501 17.093 1.00 0.00 O1 C

ATOM 2371 H3X POPCO 18 -23.212 -5.588 17.114 1.00 0.00 O1 H

ATOM 2372 H3Y POPCO 18 -23.952 -3.864 16.963 1.00 0.00 O1 H

ATOM 2373 C34 POPCO 18 -22.189 -4.230 15.888 1.00 0.00 O1 C

ATOM 2374 H4X POPCO 18 -21.866 -3.086 15.950 1.00 0.00 O1 H

ATOM 2375 H4Y POPCO 18 -21.322 -4.932 15.795 1.00 0.00 O1 H

ATOM 2376 C35 POPCO 18 -22.993 -4.484 14.611 1.00 0.00 O1 C

ATOM 2377 H5X POPCO 18 -23.278 -5.556 14.566 1.00 0.00 O1 H

ATOM 2378 H5Y POPCO 18 -23.937 -3.841 14.574 1.00 0.00 O1 H

ATOM 2379 C36 POPCO 18 -22.104 -4.172 13.309 1.00 0.00 O1 C

ATOM 2380 H6X POPCO 18 -21.522 -3.225 13.461 1.00 0.00 O1 H

ATOM 2381 H6Y POPCO 18 -21.440 -5.030 13.206 1.00 0.00 O1 H

ATOM 2382 C37 POPCO 18 -23.006 -3.914 12.069 1.00 0.00 O1 C

ATOM 2383 H7X POPCO 18 -23.529 -4.903 11.801 1.00 0.00 O1 H

ATOM 2384 H7Y POPCO 18 -23.768 -3.182 12.362 1.00 0.00 O1 H

ATOM 2385 C38 POPCO 18 -22.161 -3.416 10.882 1.00 0.00 O1 C

ATOM 2386 H8X POPCO 18 -21.577 -2.501 11.156 1.00 0.00 O1 H

ATOM 2387 H8Y POPCO 18 -21.557 -4.211 10.343 1.00 0.00 O1 H

ATOM 2388 C39 POPCO 18 -23.049 -2.898 9.782 1.00 0.00 O1 C

ATOM 2389 H9X POPCO 18 -23.831 -3.686 9.643 1.00 0.00 O1 H

ATOM 2390 H9Y POPCO 18 -23.736 -1.999 10.024 1.00 0.00 O1 H

ATOM 2391 C310 POPCO 18 -22.155 -2.480 8.605 1.00 0.00 O1 C

ATOM 2392 H10X POPCO 18 -21.432 -1.747 8.967 1.00 0.00 O1 H

ATOM 2393 H10Y POPCO 18 -21.596 -3.328 8.320 1.00 0.00 O1 H

ATOM 2394 C311 POPCO 18 -22.967 -2.171 7.356 1.00 0.00 O1 C

ATOM 2395 H11X POPCO 18 -23.471 -3.128 7.011 1.00 0.00 O1 H

ATOM 2396 H11Y POPCO 18 -23.840 -1.504 7.675 1.00 0.00 O1 H

ATOM 2397 C312 POPCO 18 -22.114 -1.576 6.235 1.00 0.00 O1 C

ATOM 2398 H12X POPCO 18 -21.667 -0.665 6.695 1.00 0.00 O1 H

ATOM 2399 H12Y POPCO 18 -21.309 -2.279 5.878 1.00 0.00 O1 H

ATOM 2400 C313 POPCO 18 -22.950 -1.109 4.993 1.00 0.00 O1 C

ATOM 2401 H13X POPCO 18 -23.325 -2.041 4.542 1.00 0.00 O1 H

ATOM 2402 H13Y POPCO 18 -23.764 -0.558 5.354 1.00 0.00 O1 H

ATOM 2403 C314 POPCO 18 -22.000 -0.276 4.023 1.00 0.00 O1 C

ATOM 2404 H14X POPCO 18 -21.627 0.711 4.374 1.00 0.00 O1 H

ATOM 2405 H14Y POPCO 18 -21.154 -0.965 3.889 1.00 0.00 O1 H

ATOM 2406 C315 POPCO 18 -22.683 -0.128 2.574 1.00 0.00 O1 C

ATOM 2407 H15X POPCO 18 -23.029 -1.183 2.445 1.00 0.00 O1 H

ATOM 2408 H15Y POPCO 18 -23.610 0.575 2.746 1.00 0.00 O1 H

ATOM 2409 C316 POPCO 18 -21.739 0.384 1.527 1.00 0.00 O1 C

ATOM 2410 H16X POPCO 18 -21.028 -0.455 1.213 1.00 0.00 O1 H

ATOM 2411 H16Y POPCO 18 -22.270 0.656 0.600 1.00 0.00 O1 H

ATOM 2412 H16Z POPCO 18 -21.104 1.204 1.947 1.00 0.00 O1 H

ATOM 2413 N POPCO 19 -13.777 -45.239 17.570 1.00 0.00 O1 N

ATOM 2414 C12 POPCO 19 -13.166 -46.316 16.717 1.00 0.00 O1 C

ATOM 2415 H12A POPCO 19 -12.760 -47.046 17.440 1.00 0.00 O1 H

ATOM 2416 H12B POPCO 19 -12.286 -45.961 16.219 1.00 0.00 O1 H

ATOM 2417 C13 POPCO 19 -14.648 -45.764 18.602 1.00 0.00 O1 C

ATOM 2418 H13A POPCO 19 -14.125 -46.494 19.158 1.00 0.00 O1 H

ATOM 2419 H13B POPCO 19 -15.356 -46.504 18.044 1.00 0.00 O1 H

ATOM 2420 H13C POPCO 19 -15.097 -45.088 19.257 1.00 0.00 O1 H

ATOM 2421 C14 POPCO 19 -12.703 -44.430 18.270 1.00 0.00 O1 C

ATOM 2422 H14A POPCO 19 -12.195 -43.860 17.557 1.00 0.00 O1 H

ATOM 2423 H14B POPCO 19 -12.081 -45.120 18.778 1.00 0.00 O1 H

ATOM 2424 H14C POPCO 19 -13.324 -43.766 18.981 1.00 0.00 O1 H

ATOM 2425 C15 POPCO 19 -14.650 -44.397 16.681 1.00 0.00 O1 C

ATOM 2426 H15A POPCO 19 -14.074 -44.048 15.821 1.00 0.00 O1 H

ATOM 2427 H15B POPCO 19 -15.469 -44.938 16.286 1.00 0.00 O1 H

ATOM 2428 H15C POPCO 19 -15.131 -43.588 17.146 1.00 0.00 O1 H

ATOM 2429 C11 POPCO 19 -14.032 -47.018 15.610 1.00 0.00 O1 C

ATOM 2430 H11A POPCO 19 -14.351 -46.223 14.840 1.00 0.00 O1 H

ATOM 2431 H11B POPCO 19 -13.423 -47.793 15.084 1.00 0.00 O1 H

ATOM 2432 P POPCO 19 -16.684 -47.430 15.883 1.00 0.00 O1 P

ATOM 2433 O13 POPCO 19 -17.258 -47.001 17.158 1.00 0.00 O1 O

ATOM 2434 O14 POPCO 19 -17.093 -48.624 15.219 1.00 0.00 O1 O

ATOM 2435 O12 POPCO 19 -15.173 -47.643 16.117 1.00 0.00 O1 O

ATOM 2436 O11 POPCO 19 -16.723 -46.252 14.846 1.00 0.00 O1 O

ATOM 2437 C1 POPCO 19 -16.522 -46.322 13.425 1.00 0.00 O1 C

ATOM 2438 HA POPCO 19 -15.423 -46.370 13.137 1.00 0.00 O1 H

ATOM 2439 HB POPCO 19 -17.000 -47.288 13.018 1.00 0.00 O1 H

ATOM 2440 C2 POPCO 19 -17.194 -45.202 12.623 1.00 0.00 O1 C

ATOM 2441 HS POPCO 19 -18.296 -45.388 12.662 1.00 0.00 O1 H

ATOM 2442 O21 POPCO 19 -16.583 -45.279 11.301 1.00 0.00 O1 O

ATOM 2443 C21 POPCO 19 -17.116 -45.922 10.371 1.00 0.00 O1 C

ATOM 2444 O22 POPCO 19 -18.133 -46.649 10.419 1.00 0.00 O1 O

ATOM 2445 C22 POPCO 19 -16.277 -45.621 9.060 1.00 0.00 O1 C

ATOM 2446 H2R POPCO 19 -16.277 -44.514 8.918 1.00 0.00 O1 H

ATOM 2447 H2S POPCO 19 -15.179 -45.823 9.249 1.00 0.00 O1 H

ATOM 2448 C3 POPCO 19 -16.906 -43.752 13.172 1.00 0.00 O1 C

ATOM 2449 HX POPCO 19 -17.509 -43.035 12.505 1.00 0.00 O1 H

ATOM 2450 HY POPCO 19 -17.229 -43.731 14.214 1.00 0.00 O1 H

ATOM 2451 O31 POPCO 19 -15.572 -43.433 13.089 1.00 0.00 O1 O

ATOM 2452 C31 POPCO 19 -15.321 -42.308 13.699 1.00 0.00 O1 C

ATOM 2453 O32 POPCO 19 -16.130 -41.524 14.161 1.00 0.00 O1 O

ATOM 2454 C32 POPCO 19 -13.860 -41.987 13.751 1.00 0.00 O1 C

ATOM 2455 H2X POPCO 19 -13.601 -41.552 14.738 1.00 0.00 O1 H

ATOM 2456 H2Y POPCO 19 -13.251 -42.912 13.834 1.00 0.00 O1 H

ATOM 2457 C23 POPCO 19 -16.678 -46.355 7.744 1.00 0.00 O1 C

ATOM 2458 H3R POPCO 19 -16.423 -47.401 7.924 1.00 0.00 O1 H

ATOM 2459 H3S POPCO 19 -17.791 -46.101 7.538 1.00 0.00 O1 H

ATOM 2460 C24 POPCO 19 -15.995 -45.856 6.486 1.00 0.00 O1 C

ATOM 2461 H4R POPCO 19 -15.836 -44.781 6.450 1.00 0.00 O1 H

ATOM 2462 H4S POPCO 19 -15.000 -46.328 6.441 1.00 0.00 O1 H

ATOM 2463 C25 POPCO 19 -16.848 -46.155 5.196 1.00 0.00 O1 C

ATOM 2464 H5R POPCO 19 -16.686 -47.197 4.945 1.00 0.00 O1 H

ATOM 2465 H5S POPCO 19 -17.959 -46.022 5.392 1.00 0.00 O1 H

ATOM 2466 C26 POPCO 19 -16.530 -45.194 4.050 1.00 0.00 O1 C

ATOM 2467 H6R POPCO 19 -16.900 -44.179 4.419 1.00 0.00 O1 H

ATOM 2468 H6S POPCO 19 -15.438 -45.144 3.930 1.00 0.00 O1 H

ATOM 2469 C27 POPCO 19 -17.381 -45.545 2.789 1.00 0.00 O1 C

ATOM 2470 H7R POPCO 19 -17.372 -46.635 2.664 1.00 0.00 O1 H

ATOM 2471 H7S POPCO 19 -18.406 -45.342 3.082 1.00 0.00 O1 H

ATOM 2472 C28 POPCO 19 -16.948 -44.628 1.534 1.00 0.00 O1 C

ATOM 2473 H8R POPCO 19 -17.243 -43.546 1.917 1.00 0.00 O1 H

ATOM 2474 H8S POPCO 19 -15.846 -44.946 1.507 1.00 0.00 O1 H

ATOM 2475 C29 POPCO 19 -17.537 -44.839 0.124 1.00 0.00 O1 C

ATOM 2476 H91 POPCO 19 -18.554 -45.227 0.045 1.00 0.00 O1 H

ATOM 2477 C210 POPCO 19 -16.852 -44.591 -0.974 1.00 0.00 O1 C

ATOM 2478 H101 POPCO 19 -17.376 -44.844 -1.917 1.00 0.00 O1 H

ATOM 2479 C211 POPCO 19 -15.450 -44.086 -1.079 1.00 0.00 O1 C

ATOM 2480 H11R POPCO 19 -15.211 -43.452 -0.183 1.00 0.00 O1 H

ATOM 2481 H11S POPCO 19 -14.869 -45.038 -1.007 1.00 0.00 O1 H

ATOM 2482 C212 POPCO 19 -15.128 -43.370 -2.386 1.00 0.00 O1 C

ATOM 2483 H12R POPCO 19 -15.773 -42.544 -2.570 1.00 0.00 O1 H

ATOM 2484 H12S POPCO 19 -14.064 -43.009 -2.214 1.00 0.00 O1 H

ATOM 2485 C213 POPCO 19 -14.865 -44.227 -3.638 1.00 0.00 O1 C

ATOM 2486 H13R POPCO 19 -14.151 -45.107 -3.332 1.00 0.00 O1 H

ATOM 2487 H13S POPCO 19 -15.798 -44.671 -4.015 1.00 0.00 O1 H

ATOM 2488 C214 POPCO 19 -14.259 -43.363 -4.797 1.00 0.00 O1 C

ATOM 2489 H14R POPCO 19 -14.934 -42.496 -5.044 1.00 0.00 O1 H

ATOM 2490 H14S POPCO 19 -13.209 -42.949 -4.601 1.00 0.00 O1 H

ATOM 2491 C215 POPCO 19 -14.163 -44.178 -6.112 1.00 0.00 O1 C

ATOM 2492 H15R POPCO 19 -13.383 -44.991 -6.008 1.00 0.00 O1 H

ATOM 2493 H15S POPCO 19 -15.116 -44.720 -6.226 1.00 0.00 O1 H

ATOM 2494 C216 POPCO 19 -13.668 -43.312 -7.327 1.00 0.00 O1 C

ATOM 2495 H16R POPCO 19 -14.118 -42.344 -7.302 1.00 0.00 O1 H

ATOM 2496 H16S POPCO 19 -12.623 -43.053 -7.146 1.00 0.00 O1 H

ATOM 2497 C217 POPCO 19 -13.922 -43.934 -8.700 1.00 0.00 O1 C

ATOM 2498 H17R POPCO 19 -13.474 -44.974 -8.764 1.00 0.00 O1 H

ATOM 2499 H17S POPCO 19 -14.998 -43.986 -8.763 1.00 0.00 O1 H

ATOM 2500 C218 POPCO 19 -13.394 -43.143 -9.935 1.00 0.00 O1 C

ATOM 2501 H18R POPCO 19 -13.830 -43.661 -10.849 1.00 0.00 O1 H

ATOM 2502 H18S POPCO 19 -13.665 -42.041 -9.934 1.00 0.00 O1 H

ATOM 2503 H18T POPCO 19 -12.324 -43.134 -10.061 1.00 0.00 O1 H

ATOM 2504 C33 POPCO 19 -13.383 -41.042 12.596 1.00 0.00 O1 C

ATOM 2505 H3X POPCO 19 -13.880 -40.015 12.665 1.00 0.00 O1 H

ATOM 2506 H3Y POPCO 19 -12.252 -40.912 12.625 1.00 0.00 O1 H

ATOM 2507 C34 POPCO 19 -13.690 -41.722 11.223 1.00 0.00 O1 C

ATOM 2508 H4X POPCO 19 -13.232 -42.663 11.292 1.00 0.00 O1 H

ATOM 2509 H4Y POPCO 19 -14.778 -41.935 11.166 1.00 0.00 O1 H

ATOM 2510 C35 POPCO 19 -13.203 -41.015 9.964 1.00 0.00 O1 C

ATOM 2511 H5X POPCO 19 -13.309 -39.944 10.139 1.00 0.00 O1 H

ATOM 2512 H5Y POPCO 19 -12.118 -41.111 9.843 1.00 0.00 O1 H

ATOM 2513 C36 POPCO 19 -14.002 -41.369 8.648 1.00 0.00 O1 C

ATOM 2514 H6X POPCO 19 -13.692 -42.385 8.322 1.00 0.00 O1 H

ATOM 2515 H6Y POPCO 19 -15.133 -41.269 8.878 1.00 0.00 O1 H

ATOM 2516 C37 POPCO 19 -13.699 -40.453 7.414 1.00 0.00 O1 C

ATOM 2517 H7X POPCO 19 -14.101 -39.464 7.534 1.00 0.00 O1 H

ATOM 2518 H7Y POPCO 19 -12.587 -40.367 7.397 1.00 0.00 O1 H

ATOM 2519 C38 POPCO 19 -14.083 -41.103 6.048 1.00 0.00 O1 C

ATOM 2520 H8X POPCO 19 -13.772 -42.213 5.917 1.00 0.00 O1 H

ATOM 2521 H8Y POPCO 19 -15.210 -41.154 6.040 1.00 0.00 O1 H

ATOM 2522 C39 POPCO 19 -13.586 -40.288 4.886 1.00 0.00 O1 C

ATOM 2523 H9X POPCO 19 -14.102 -39.289 4.903 1.00 0.00 O1 H

ATOM 2524 H9Y POPCO 19 -12.477 -40.175 4.923 1.00 0.00 O1 H

ATOM 2525 C310 POPCO 19 -14.038 -40.931 3.512 1.00 0.00 O1 C

ATOM 2526 H10X POPCO 19 -13.807 -42.028 3.567 1.00 0.00 O1 H

ATOM 2527 H10Y POPCO 19 -15.157 -40.752 3.454 1.00 0.00 O1 H

ATOM 2528 C311 POPCO 19 -13.382 -40.236 2.299 1.00 0.00 O1 C

ATOM 2529 H11X POPCO 19 -13.811 -39.263 2.300 1.00 0.00 O1 H

ATOM 2530 H11Y POPCO 19 -12.279 -40.180 2.500 1.00 0.00 O1 H

ATOM 2531 C312 POPCO 19 -13.546 -40.947 0.955 1.00 0.00 O1 C

ATOM 2532 H12X POPCO 19 -13.017 -41.941 1.019 1.00 0.00 O1 H

ATOM 2533 H12Y POPCO 19 -14.638 -41.222 0.625 1.00 0.00 O1 H

ATOM 2534 C313 POPCO 19 -13.025 -40.037 -0.124 1.00 0.00 O1 C

ATOM 2535 H13X POPCO 19 -13.730 -39.179 -0.159 1.00 0.00 O1 H

ATOM 2536 H13Y POPCO 19 -12.039 -39.655 0.118 1.00 0.00 O1 H

ATOM 2537 C314 POPCO 19 -13.085 -40.524 -1.557 1.00 0.00 O1 C

ATOM 2538 H14X POPCO 19 -12.583 -41.459 -1.716 1.00 0.00 O1 H

ATOM 2539 H14Y POPCO 19 -14.163 -40.776 -1.687 1.00 0.00 O1 H

ATOM 2540 C315 POPCO 19 -12.664 -39.397 -2.462 1.00 0.00 O1 C

ATOM 2541 H15X POPCO 19 -13.048 -38.399 -2.107 1.00 0.00 O1 H

ATOM 2542 H15Y POPCO 19 -11.481 -39.316 -2.366 1.00 0.00 O1 H

ATOM 2543 C316 POPCO 19 -12.953 -39.634 -3.919 1.00 0.00 O1 C

ATOM 2544 H16X POPCO 19 -14.034 -39.756 -3.995 1.00 0.00 O1 H

ATOM 2545 H16Y POPCO 19 -12.761 -38.743 -4.485 1.00 0.00 O1 H

ATOM 2546 H16Z POPCO 19 -12.495 -40.531 -4.376 1.00 0.00 O1 H

ATOM 2547 N POPCO 20 -9.481 -35.453 21.939 1.00 0.00 O1 N

ATOM 2548 C12 POPCO 20 -8.896 -34.957 20.581 1.00 0.00 O1 C

ATOM 2549 H12A POPCO 20 -7.851 -35.216 20.553 1.00 0.00 O1 H

ATOM 2550 H12B POPCO 20 -8.917 -33.862 20.526 1.00 0.00 O1 H

ATOM 2551 C13 POPCO 20 -9.540 -37.017 21.998 1.00 0.00 O1 C

ATOM 2552 H13A POPCO 20 -8.495 -37.373 22.014 1.00 0.00 O1 H

ATOM 2553 H13B POPCO 20 -10.026 -37.492 21.232 1.00 0.00 O1 H

ATOM 2554 H13C POPCO 20 -9.823 -37.331 23.028 1.00 0.00 O1 H

ATOM 2555 C14 POPCO 20 -8.588 -34.888 23.003 1.00 0.00 O1 C

ATOM 2556 H14A POPCO 20 -8.300 -33.841 22.778 1.00 0.00 O1 H

ATOM 2557 H14B POPCO 20 -7.554 -35.348 23.042 1.00 0.00 O1 H

ATOM 2558 H14C POPCO 20 -8.950 -34.913 23.989 1.00 0.00 O1 H

ATOM 2559 C15 POPCO 20 -10.895 -34.958 22.117 1.00 0.00 O1 C

ATOM 2560 H15A POPCO 20 -10.945 -33.870 21.893 1.00 0.00 O1 H

ATOM 2561 H15B POPCO 20 -11.684 -35.550 21.689 1.00 0.00 O1 H

ATOM 2562 H15C POPCO 20 -11.155 -35.060 23.176 1.00 0.00 O1 H

ATOM 2563 C11 POPCO 20 -9.448 -35.544 19.259 1.00 0.00 O1 C

ATOM 2564 H11A POPCO 20 -10.578 -35.708 19.364 1.00 0.00 O1 H

ATOM 2565 H11B POPCO 20 -9.215 -34.742 18.499 1.00 0.00 O1 H

ATOM 2566 P POPCO 20 -9.298 -38.095 18.404 1.00 0.00 O1 P

ATOM 2567 O13 POPCO 20 -9.817 -38.897 19.577 1.00 0.00 O1 O

ATOM 2568 O14 POPCO 20 -8.213 -38.726 17.606 1.00 0.00 O1 O

ATOM 2569 O12 POPCO 20 -8.782 -36.714 18.950 1.00 0.00 O1 O

ATOM 2570 O11 POPCO 20 -10.560 -37.739 17.554 1.00 0.00 O1 O

ATOM 2571 C1 POPCO 20 -10.359 -37.005 16.349 1.00 0.00 O1 C

ATOM 2572 HA POPCO 20 -9.683 -36.157 16.477 1.00 0.00 O1 H

ATOM 2573 HB POPCO 20 -9.917 -37.698 15.591 1.00 0.00 O1 H

ATOM 2574 C2 POPCO 20 -11.774 -36.553 15.858 1.00 0.00 O1 C

ATOM 2575 HS POPCO 20 -12.401 -37.470 15.830 1.00 0.00 O1 H

ATOM 2576 O21 POPCO 20 -11.752 -35.967 14.485 1.00 0.00 O1 O

ATOM 2577 C21 POPCO 20 -12.216 -36.723 13.471 1.00 0.00 O1 C

ATOM 2578 O22 POPCO 20 -12.806 -37.768 13.593 1.00 0.00 O1 O

ATOM 2579 C22 POPCO 20 -11.925 -36.038 12.122 1.00 0.00 O1 C

ATOM 2580 H2R POPCO 20 -12.551 -35.112 12.016 1.00 0.00 O1 H

ATOM 2581 H2S POPCO 20 -10.915 -35.709 12.087 1.00 0.00 O1 H

ATOM 2582 C3 POPCO 20 -12.334 -35.583 16.885 1.00 0.00 O1 C

ATOM 2583 HX POPCO 20 -13.336 -35.364 16.521 1.00 0.00 O1 H

ATOM 2584 HY POPCO 20 -12.376 -35.938 17.934 1.00 0.00 O1 H

ATOM 2585 O31 POPCO 20 -11.479 -34.402 16.913 1.00 0.00 O1 O

ATOM 2586 C31 POPCO 20 -11.900 -33.262 16.323 1.00 0.00 O1 C

ATOM 2587 O32 POPCO 20 -13.046 -33.043 15.943 1.00 0.00 O1 O

ATOM 2588 C32 POPCO 20 -10.777 -32.155 16.188 1.00 0.00 O1 C

ATOM 2589 H2X POPCO 20 -11.303 -31.266 15.989 1.00 0.00 O1 H

ATOM 2590 H2Y POPCO 20 -10.374 -32.005 17.198 1.00 0.00 O1 H

ATOM 2591 C23 POPCO 20 -12.294 -36.855 10.920 1.00 0.00 O1 C

ATOM 2592 H3R POPCO 20 -11.888 -37.917 11.012 1.00 0.00 O1 H

ATOM 2593 H3S POPCO 20 -13.400 -36.927 10.836 1.00 0.00 O1 H

ATOM 2594 C24 POPCO 20 -11.777 -36.192 9.650 1.00 0.00 O1 C

ATOM 2595 H4R POPCO 20 -12.198 -35.175 9.599 1.00 0.00 O1 H

ATOM 2596 H4S POPCO 20 -10.676 -36.092 9.615 1.00 0.00 O1 H

ATOM 2597 C25 POPCO 20 -12.377 -36.942 8.460 1.00 0.00 O1 C

ATOM 2598 H5R POPCO 20 -12.269 -38.048 8.535 1.00 0.00 O1 H

ATOM 2599 H5S POPCO 20 -13.483 -36.766 8.495 1.00 0.00 O1 H

ATOM 2600 C26 POPCO 20 -11.762 -36.571 7.189 1.00 0.00 O1 C

ATOM 2601 H6R POPCO 20 -11.670 -35.510 6.974 1.00 0.00 O1 H

ATOM 2602 H6S POPCO 20 -10.668 -37.005 7.060 1.00 0.00 O1 H

ATOM 2603 C27 POPCO 20 -12.374 -37.154 5.884 1.00 0.00 O1 C

ATOM 2604 H7R POPCO 20 -12.244 -38.269 5.984 1.00 0.00 O1 H

ATOM 2605 H7S POPCO 20 -13.490 -36.964 5.759 1.00 0.00 O1 H

ATOM 2606 C28 POPCO 20 -11.700 -36.609 4.635 1.00 0.00 O1 C

ATOM 2607 H8R POPCO 20 -11.887 -35.482 4.668 1.00 0.00 O1 H

ATOM 2608 H8S POPCO 20 -10.628 -36.696 4.622 1.00 0.00 O1 H

ATOM 2609 C29 POPCO 20 -12.341 -37.121 3.317 1.00 0.00 O1 C

ATOM 2610 H91 POPCO 20 -13.297 -37.570 3.386 1.00 0.00 O1 H

ATOM 2611 C210 POPCO 20 -11.809 -37.009 2.095 1.00 0.00 O1 C

ATOM 2612 H101 POPCO 20 -12.271 -37.356 1.166 1.00 0.00 O1 H

ATOM 2613 C211 POPCO 20 -10.473 -36.341 1.835 1.00 0.00 O1 C

ATOM 2614 H11R POPCO 20 -10.137 -35.705 2.725 1.00 0.00 O1 H

ATOM 2615 H11S POPCO 20 -9.790 -37.194 1.706 1.00 0.00 O1 H

ATOM 2616 C212 POPCO 20 -10.410 -35.562 0.475 1.00 0.00 O1 C

ATOM 2617 H12R POPCO 20 -11.204 -34.802 0.426 1.00 0.00 O1 H

ATOM 2618 H12S POPCO 20 -9.461 -34.954 0.391 1.00 0.00 O1 H

ATOM 2619 C213 POPCO 20 -10.397 -36.500 -0.719 1.00 0.00 O1 C

ATOM 2620 H13R POPCO 20 -9.669 -37.387 -0.470 1.00 0.00 O1 H

ATOM 2621 H13S POPCO 20 -11.436 -36.835 -0.866 1.00 0.00 O1 H

ATOM 2622 C214 POPCO 20 -10.054 -35.844 -2.107 1.00 0.00 O1 C

ATOM 2623 H14R POPCO 20 -10.599 -34.829 -2.126 1.00 0.00 O1 H

ATOM 2624 H14S POPCO 20 -9.022 -35.500 -2.041 1.00 0.00 O1 H

ATOM 2625 C215 POPCO 20 -10.238 -36.699 -3.333 1.00 0.00 O1 C

ATOM 2626 H15R POPCO 20 -9.611 -37.590 -3.189 1.00 0.00 O1 H

ATOM 2627 H15S POPCO 20 -11.318 -37.000 -3.334 1.00 0.00 O1 H

ATOM 2628 C216 POPCO 20 -9.898 -36.118 -4.745 1.00 0.00 O1 C

ATOM 2629 H16R POPCO 20 -10.527 -35.231 -4.984 1.00 0.00 O1 H

ATOM 2630 H16S POPCO 20 -8.781 -35.925 -4.777 1.00 0.00 O1 H

ATOM 2631 C217 POPCO 20 -10.224 -37.171 -5.819 1.00 0.00 O1 C

ATOM 2632 H17R POPCO 20 -9.575 -38.107 -5.659 1.00 0.00 O1 H

ATOM 2633 H17S POPCO 20 -11.211 -37.586 -5.711 1.00 0.00 O1 H

ATOM 2634 C218 POPCO 20 -9.929 -36.630 -7.207 1.00 0.00 O1 C

ATOM 2635 H18R POPCO 20 -10.272 -37.412 -7.989 1.00 0.00 O1 H

ATOM 2636 H18S POPCO 20 -10.502 -35.673 -7.314 1.00 0.00 O1 H

ATOM 2637 H18T POPCO 20 -8.859 -36.556 -7.307 1.00 0.00 O1 H

ATOM 2638 C33 POPCO 20 -9.767 -32.431 15.045 1.00 0.00 O1 C

ATOM 2639 H3X POPCO 20 -8.907 -31.685 15.006 1.00 0.00 O1 H

ATOM 2640 H3Y POPCO 20 -9.309 -33.422 15.111 1.00 0.00 O1 H

ATOM 2641 C34 POPCO 20 -10.388 -32.419 13.648 1.00 0.00 O1 C

ATOM 2642 H4X POPCO 20 -11.281 -33.159 13.580 1.00 0.00 O1 H

ATOM 2643 H4Y POPCO 20 -10.906 -31.470 13.497 1.00 0.00 O1 H

ATOM 2644 C35 POPCO 20 -9.447 -32.705 12.478 1.00 0.00 O1 C

ATOM 2645 H5X POPCO 20 -8.485 -32.042 12.504 1.00 0.00 O1 H

ATOM 2646 H5Y POPCO 20 -9.125 -33.766 12.533 1.00 0.00 O1 H

ATOM 2647 C36 POPCO 20 -10.110 -32.426 11.067 1.00 0.00 O1 C

ATOM 2648 H6X POPCO 20 -10.971 -33.179 11.035 1.00 0.00 O1 H

ATOM 2649 H6Y POPCO 20 -10.628 -31.399 11.044 1.00 0.00 O1 H

ATOM 2650 C37 POPCO 20 -9.211 -32.997 9.902 1.00 0.00 O1 C

ATOM 2651 H7X POPCO 20 -8.232 -32.477 9.745 1.00 0.00 O1 H

ATOM 2652 H7Y POPCO 20 -8.896 -34.060 10.135 1.00 0.00 O1 H

ATOM 2653 C38 POPCO 20 -9.903 -32.858 8.512 1.00 0.00 O1 C

ATOM 2654 H8X POPCO 20 -10.830 -33.456 8.553 1.00 0.00 O1 H

ATOM 2655 H8Y POPCO 20 -10.208 -31.815 8.247 1.00 0.00 O1 H

ATOM 2656 C39 POPCO 20 -9.021 -33.300 7.332 1.00 0.00 O1 C

ATOM 2657 H9X POPCO 20 -8.200 -32.563 7.346 1.00 0.00 O1 H

ATOM 2658 H9Y POPCO 20 -8.560 -34.305 7.697 1.00 0.00 O1 H

ATOM 2659 C310 POPCO 20 -9.649 -33.378 5.966 1.00 0.00 O1 C

ATOM 2660 H10X POPCO 20 -9.179 -34.142 5.322 1.00 0.00 O1 H

ATOM 2661 H10Y POPCO 20 -10.676 -33.625 5.975 1.00 0.00 O1 H

ATOM 2662 C311 POPCO 20 -9.443 -32.167 5.112 1.00 0.00 O1 C

ATOM 2663 H11X POPCO 20 -9.769 -31.242 5.664 1.00 0.00 O1 H

ATOM 2664 H11Y POPCO 20 -8.354 -32.092 5.043 1.00 0.00 O1 H

ATOM 2665 C312 POPCO 20 -10.125 -32.413 3.734 1.00 0.00 O1 C

ATOM 2666 H12X POPCO 20 -9.673 -33.422 3.365 1.00 0.00 O1 H

ATOM 2667 H12Y POPCO 20 -11.168 -32.620 3.907 1.00 0.00 O1 H

ATOM 2668 C313 POPCO 20 -9.810 -31.279 2.806 1.00 0.00 O1 C

ATOM 2669 H13X POPCO 20 -10.323 -30.277 3.040 1.00 0.00 O1 H

ATOM 2670 H13Y POPCO 20 -8.687 -31.091 2.840 1.00 0.00 O1 H

ATOM 2671 C314 POPCO 20 -10.247 -31.566 1.417 1.00 0.00 O1 C

ATOM 2672 H14X POPCO 20 -9.875 -32.628 1.204 1.00 0.00 O1 H

ATOM 2673 H14Y POPCO 20 -11.346 -31.471 1.353 1.00 0.00 O1 H

ATOM 2674 C315 POPCO 20 -9.636 -30.593 0.411 1.00 0.00 O1 C

ATOM 2675 H15X POPCO 20 -9.653 -29.558 0.648 1.00 0.00 O1 H

ATOM 2676 H15Y POPCO 20 -8.595 -30.866 0.429 1.00 0.00 O1 H

ATOM 2677 C316 POPCO 20 -10.148 -30.768 -1.022 1.00 0.00 O1 C

ATOM 2678 H16X POPCO 20 -11.064 -30.231 -1.161 1.00 0.00 O1 H

ATOM 2679 H16Y POPCO 20 -9.463 -30.171 -1.678 1.00 0.00 O1 H

ATOM 2680 H16Z POPCO 20 -10.171 -31.794 -1.518 1.00 0.00 O1 H

ATOM 2681 N POPCO 21 -14.812 -26.980 22.105 1.00 0.00 O1 N

ATOM 2682 C12 POPCO 21 -13.783 -27.949 21.600 1.00 0.00 O1 C

ATOM 2683 H12A POPCO 21 -13.232 -28.368 22.470 1.00 0.00 O1 H

ATOM 2684 H12B POPCO 21 -12.966 -27.398 21.058 1.00 0.00 O1 H

ATOM 2685 C13 POPCO 21 -15.388 -27.383 23.403 1.00 0.00 O1 C

ATOM 2686 H13A POPCO 21 -14.535 -27.456 24.154 1.00 0.00 O1 H

ATOM 2687 H13B POPCO 21 -15.813 -28.378 23.365 1.00 0.00 O1 H

ATOM 2688 H13C POPCO 21 -16.215 -26.758 23.734 1.00 0.00 O1 H

ATOM 2689 C14 POPCO 21 -13.979 -25.659 22.262 1.00 0.00 O1 C

ATOM 2690 H14A POPCO 21 -13.647 -25.237 21.309 1.00 0.00 O1 H

ATOM 2691 H14B POPCO 21 -13.067 -25.804 22.895 1.00 0.00 O1 H

ATOM 2692 H14C POPCO 21 -14.576 -24.877 22.712 1.00 0.00 O1 H

ATOM 2693 C15 POPCO 21 -15.987 -26.738 21.286 1.00 0.00 O1 C

ATOM 2694 H15A POPCO 21 -15.747 -26.569 20.270 1.00 0.00 O1 H

ATOM 2695 H15B POPCO 21 -16.631 -27.640 21.303 1.00 0.00 O1 H

ATOM 2696 H15C POPCO 21 -16.549 -25.837 21.613 1.00 0.00 O1 H

ATOM 2697 C11 POPCO 21 -14.325 -29.168 20.783 1.00 0.00 O1 C

ATOM 2698 H11A POPCO 21 -14.750 -28.906 19.821 1.00 0.00 O1 H

ATOM 2699 H11B POPCO 21 -13.576 -29.898 20.519 1.00 0.00 O1 H

ATOM 2700 P POPCO 21 -16.708 -30.192 21.347 1.00 0.00 O1 P

ATOM 2701 O13 POPCO 21 -17.582 -29.615 22.392 1.00 0.00 O1 O

ATOM 2702 O14 POPCO 21 -16.824 -31.642 21.142 1.00 0.00 O1 O

ATOM 2703 O12 POPCO 21 -15.232 -29.825 21.626 1.00 0.00 O1 O

ATOM 2704 O11 POPCO 21 -17.213 -29.531 19.922 1.00 0.00 O1 O

ATOM 2705 C1 POPCO 21 -16.802 -30.115 18.664 1.00 0.00 O1 C

ATOM 2706 HA POPCO 21 -15.634 -30.078 18.572 1.00 0.00 O1 H

ATOM 2707 HB POPCO 21 -17.224 -31.165 18.487 1.00 0.00 O1 H

ATOM 2708 C2 POPCO 21 -17.474 -29.237 17.534 1.00 0.00 O1 C

ATOM 2709 HS POPCO 21 -18.593 -29.304 17.674 1.00 0.00 O1 H

ATOM 2710 O21 POPCO 21 -17.133 -29.767 16.173 1.00 0.00 O1 O

ATOM 2711 C21 POPCO 21 -18.008 -29.560 15.224 1.00 0.00 O1 C

ATOM 2712 O22 POPCO 21 -19.067 -28.984 15.401 1.00 0.00 O1 O

ATOM 2713 C22 POPCO 21 -17.564 -30.178 13.874 1.00 0.00 O1 C

ATOM 2714 H2R POPCO 21 -16.475 -30.453 13.875 1.00 0.00 O1 H

ATOM 2715 H2S POPCO 21 -18.212 -31.076 13.773 1.00 0.00 O1 H

ATOM 2716 C3 POPCO 21 -17.088 -27.763 17.625 1.00 0.00 O1 C

ATOM 2717 HX POPCO 21 -17.536 -27.160 16.828 1.00 0.00 O1 H

ATOM 2718 HY POPCO 21 -17.560 -27.434 18.604 1.00 0.00 O1 H

ATOM 2719 O31 POPCO 21 -15.678 -27.618 17.558 1.00 0.00 O1 O

ATOM 2720 C31 POPCO 21 -15.240 -26.440 17.947 1.00 0.00 O1 C

ATOM 2721 O32 POPCO 21 -15.935 -25.589 18.505 1.00 0.00 O1 O

ATOM 2722 C32 POPCO 21 -13.720 -26.279 17.805 1.00 0.00 O1 C

ATOM 2723 H2X POPCO 21 -13.307 -25.752 18.715 1.00 0.00 O1 H

ATOM 2724 H2Y POPCO 21 -13.262 -27.205 17.627 1.00 0.00 O1 H

ATOM 2725 C23 POPCO 21 -17.922 -29.222 12.724 1.00 0.00 O1 C

ATOM 2726 H3R POPCO 21 -18.983 -29.307 12.362 1.00 0.00 O1 H

ATOM 2727 H3S POPCO 21 -17.736 -28.219 13.124 1.00 0.00 O1 H

ATOM 2728 C24 POPCO 21 -17.056 -29.506 11.526 1.00 0.00 O1 C

ATOM 2729 H4R POPCO 21 -16.031 -28.993 11.719 1.00 0.00 O1 H

ATOM 2730 H4S POPCO 21 -16.746 -30.590 11.513 1.00 0.00 O1 H

ATOM 2731 C25 POPCO 21 -17.632 -29.190 10.145 1.00 0.00 O1 C

ATOM 2732 H5R POPCO 21 -18.495 -29.874 9.988 1.00 0.00 O1 H

ATOM 2733 H5S POPCO 21 -17.945 -28.075 10.180 1.00 0.00 O1 H

ATOM 2734 C26 POPCO 21 -16.724 -29.432 8.990 1.00 0.00 O1 C

ATOM 2735 H6R POPCO 21 -15.846 -28.793 9.343 1.00 0.00 O1 H

ATOM 2736 H6S POPCO 21 -16.319 -30.420 8.953 1.00 0.00 O1 H

ATOM 2737 C27 POPCO 21 -17.338 -28.828 7.701 1.00 0.00 O1 C

ATOM 2738 H7R POPCO 21 -18.344 -29.185 7.427 1.00 0.00 O1 H

ATOM 2739 H7S POPCO 21 -17.334 -27.738 7.997 1.00 0.00 O1 H

ATOM 2740 C28 POPCO 21 -16.370 -28.991 6.437 1.00 0.00 O1 C

ATOM 2741 H8R POPCO 21 -15.307 -28.778 6.660 1.00 0.00 O1 H

ATOM 2742 H8S POPCO 21 -16.352 -30.186 6.295 1.00 0.00 O1 H

ATOM 2743 C29 POPCO 21 -16.956 -28.109 5.302 1.00 0.00 O1 C

ATOM 2744 H91 POPCO 21 -17.308 -27.197 5.693 1.00 0.00 O1 H

ATOM 2745 C210 POPCO 21 -16.916 -28.433 4.016 1.00 0.00 O1 C

ATOM 2746 H101 POPCO 21 -17.454 -27.713 3.378 1.00 0.00 O1 H

ATOM 2747 C211 POPCO 21 -16.375 -29.729 3.488 1.00 0.00 O1 C

ATOM 2748 H11R POPCO 21 -16.013 -30.383 4.279 1.00 0.00 O1 H

ATOM 2749 H11S POPCO 21 -17.254 -30.321 3.140 1.00 0.00 O1 H

ATOM 2750 C212 POPCO 21 -15.416 -29.569 2.378 1.00 0.00 O1 C

ATOM 2751 H12R POPCO 21 -14.507 -28.956 2.704 1.00 0.00 O1 H

ATOM 2752 H12S POPCO 21 -14.867 -30.558 2.171 1.00 0.00 O1 H

ATOM 2753 C213 POPCO 21 -16.069 -29.044 1.075 1.00 0.00 O1 C

ATOM 2754 H13R POPCO 21 -17.053 -29.629 0.967 1.00 0.00 O1 H

ATOM 2755 H13S POPCO 21 -16.157 -27.928 1.200 1.00 0.00 O1 H

ATOM 2756 C214 POPCO 21 -15.165 -29.299 -0.150 1.00 0.00 O1 C

ATOM 2757 H14R POPCO 21 -14.293 -28.740 0.104 1.00 0.00 O1 H

ATOM 2758 H14S POPCO 21 -14.824 -30.354 -0.162 1.00 0.00 O1 H

ATOM 2759 C215 POPCO 21 -15.826 -28.919 -1.511 1.00 0.00 O1 C

ATOM 2760 H15R POPCO 21 -16.682 -29.635 -1.453 1.00 0.00 O1 H

ATOM 2761 H15S POPCO 21 -16.298 -27.901 -1.436 1.00 0.00 O1 H

ATOM 2762 C216 POPCO 21 -15.029 -28.960 -2.843 1.00 0.00 O1 C

ATOM 2763 H16R POPCO 21 -14.129 -28.321 -2.783 1.00 0.00 O1 H

ATOM 2764 H16S POPCO 21 -14.792 -29.980 -3.079 1.00 0.00 O1 H

ATOM 2765 C217 POPCO 21 -15.934 -28.481 -3.999 1.00 0.00 O1 C

ATOM 2766 H17R POPCO 21 -16.905 -28.977 -3.907 1.00 0.00 O1 H

ATOM 2767 H17S POPCO 21 -16.147 -27.349 -3.798 1.00 0.00 O1 H

ATOM 2768 C218 POPCO 21 -15.351 -28.601 -5.443 1.00 0.00 O1 C

ATOM 2769 H18R POPCO 21 -15.936 -27.925 -6.170 1.00 0.00 O1 H

ATOM 2770 H18S POPCO 21 -14.326 -28.122 -5.459 1.00 0.00 O1 H

ATOM 2771 H18T POPCO 21 -15.237 -29.641 -5.774 1.00 0.00 O1 H

ATOM 2772 C33 POPCO 21 -13.254 -25.436 16.603 1.00 0.00 O1 C

ATOM 2773 H3X POPCO 21 -13.698 -24.442 16.539 1.00 0.00 O1 H

ATOM 2774 H3Y POPCO 21 -12.187 -25.377 16.786 1.00 0.00 O1 H

ATOM 2775 C34 POPCO 21 -13.338 -26.063 15.203 1.00 0.00 O1 C

ATOM 2776 H4X POPCO 21 -12.688 -25.518 14.512 1.00 0.00 O1 H

ATOM 2777 H4Y POPCO 21 -13.045 -27.157 15.223 1.00 0.00 O1 H

ATOM 2778 C35 POPCO 21 -14.754 -25.896 14.560 1.00 0.00 O1 C

ATOM 2779 H5X POPCO 21 -15.538 -26.544 15.123 1.00 0.00 O1 H

ATOM 2780 H5Y POPCO 21 -15.136 -24.879 14.612 1.00 0.00 O1 H

ATOM 2781 C36 POPCO 21 -14.837 -26.557 13.168 1.00 0.00 O1 C

ATOM 2782 H6X POPCO 21 -14.466 -27.619 13.298 1.00 0.00 O1 H

ATOM 2783 H6Y POPCO 21 -15.876 -26.612 12.760 1.00 0.00 O1 H

ATOM 2784 C37 POPCO 21 -13.980 -25.878 12.133 1.00 0.00 O1 C

ATOM 2785 H7X POPCO 21 -14.091 -24.798 12.271 1.00 0.00 O1 H

ATOM 2786 H7Y POPCO 21 -12.887 -26.122 12.461 1.00 0.00 O1 H

ATOM 2787 C38 POPCO 21 -14.316 -26.438 10.753 1.00 0.00 O1 C

ATOM 2788 H8X POPCO 21 -14.098 -27.465 10.633 1.00 0.00 O1 H

ATOM 2789 H8Y POPCO 21 -15.394 -26.267 10.549 1.00 0.00 O1 H

ATOM 2790 C39 POPCO 21 -13.645 -25.683 9.599 1.00 0.00 O1 C

ATOM 2791 H9X POPCO 21 -13.990 -24.628 9.604 1.00 0.00 O1 H

ATOM 2792 H9Y POPCO 21 -12.565 -25.586 9.756 1.00 0.00 O1 H

ATOM 2793 C310 POPCO 21 -13.846 -26.231 8.180 1.00 0.00 O1 C

ATOM 2794 H10X POPCO 21 -13.669 -27.326 8.306 1.00 0.00 O1 H

ATOM 2795 H10Y POPCO 21 -14.929 -26.091 7.946 1.00 0.00 O1 H

ATOM 2796 C311 POPCO 21 -12.966 -25.717 7.038 1.00 0.00 O1 C

ATOM 2797 H11X POPCO 21 -13.131 -24.661 6.806 1.00 0.00 O1 H

ATOM 2798 H11Y POPCO 21 -11.846 -25.796 7.223 1.00 0.00 O1 H

ATOM 2799 C312 POPCO 21 -13.182 -26.623 5.842 1.00 0.00 O1 C

ATOM 2800 H12X POPCO 21 -12.969 -27.679 5.967 1.00 0.00 O1 H

ATOM 2801 H12Y POPCO 21 -14.290 -26.632 5.604 1.00 0.00 O1 H

ATOM 2802 C313 POPCO 21 -12.454 -26.112 4.629 1.00 0.00 O1 C

ATOM 2803 H13X POPCO 21 -12.886 -25.161 4.347 1.00 0.00 O1 H

ATOM 2804 H13Y POPCO 21 -11.388 -25.885 4.945 1.00 0.00 O1 H

ATOM 2805 C314 POPCO 21 -12.583 -27.123 3.502 1.00 0.00 O1 C

ATOM 2806 H14X POPCO 21 -12.176 -28.160 3.785 1.00 0.00 O1 H

ATOM 2807 H14Y POPCO 21 -13.701 -27.297 3.411 1.00 0.00 O1 H

ATOM 2808 C315 POPCO 21 -11.920 -26.769 2.155 1.00 0.00 O1 C

ATOM 2809 H15X POPCO 21 -10.913 -26.389 2.595 1.00 0.00 O1 H

ATOM 2810 H15Y POPCO 21 -11.707 -27.617 1.483 1.00 0.00 O1 H

ATOM 2811 C316 POPCO 21 -12.724 -25.756 1.284 1.00 0.00 O1 C

ATOM 2812 H16X POPCO 21 -13.056 -24.819 1.856 1.00 0.00 O1 H

ATOM 2813 H16Y POPCO 21 -12.152 -25.526 0.294 1.00 0.00 O1 H

ATOM 2814 H16Z POPCO 21 -13.675 -26.330 1.158 1.00 0.00 O1 H

ATOM 2815 N POPCO 22 -9.386 -17.917 18.514 1.00 0.00 O1 N

ATOM 2816 C12 POPCO 22 -9.963 -19.237 19.064 1.00 0.00 O1 C

ATOM 2817 H12A POPCO 22 -9.351 -20.050 18.603 1.00 0.00 O1 H

ATOM 2818 H12B POPCO 22 -10.941 -19.420 18.637 1.00 0.00 O1 H

ATOM 2819 C13 POPCO 22 -8.044 -17.580 18.983 1.00 0.00 O1 C

ATOM 2820 H13A POPCO 22 -7.446 -18.531 18.890 1.00 0.00 O1 H

ATOM 2821 H13B POPCO 22 -8.077 -17.410 20.067 1.00 0.00 O1 H

ATOM 2822 H13C POPCO 22 -7.505 -16.840 18.508 1.00 0.00 O1 H

ATOM 2823 C14 POPCO 22 -9.519 -18.015 17.047 1.00 0.00 O1 C

ATOM 2824 H14A POPCO 22 -10.474 -17.845 16.590 1.00 0.00 O1 H

ATOM 2825 H14B POPCO 22 -9.048 -18.953 16.686 1.00 0.00 O1 H

ATOM 2826 H14C POPCO 22 -8.916 -17.219 16.543 1.00 0.00 O1 H

ATOM 2827 C15 POPCO 22 -10.232 -16.754 18.908 1.00 0.00 O1 C

ATOM 2828 H15A POPCO 22 -11.254 -16.990 18.813 1.00 0.00 O1 H

ATOM 2829 H15B POPCO 22 -10.110 -16.620 19.992 1.00 0.00 O1 H

ATOM 2830 H15C POPCO 22 -10.144 -15.915 18.356 1.00 0.00 O1 H

ATOM 2831 C11 POPCO 22 -10.208 -19.467 20.530 1.00 0.00 O1 C

ATOM 2832 H11A POPCO 22 -9.196 -19.466 20.959 1.00 0.00 O1 H

ATOM 2833 H11B POPCO 22 -10.691 -18.571 20.922 1.00 0.00 O1 H

ATOM 2834 P POPCO 22 -10.781 -21.973 19.928 1.00 0.00 O1 P

ATOM 2835 O13 POPCO 22 -11.338 -23.093 20.738 1.00 0.00 O1 O

ATOM 2836 O14 POPCO 22 -9.394 -22.051 19.468 1.00 0.00 O1 O

ATOM 2837 O12 POPCO 22 -10.916 -20.656 20.843 1.00 0.00 O1 O

ATOM 2838 O11 POPCO 22 -11.664 -21.605 18.666 1.00 0.00 O1 O

ATOM 2839 C1 POPCO 22 -11.219 -21.579 17.369 1.00 0.00 O1 C

ATOM 2840 HA POPCO 22 -10.621 -20.637 17.117 1.00 0.00 O1 H

ATOM 2841 HB POPCO 22 -10.649 -22.525 17.209 1.00 0.00 O1 H

ATOM 2842 C2 POPCO 22 -12.413 -21.546 16.394 1.00 0.00 O1 C

ATOM 2843 HS POPCO 22 -13.107 -22.478 16.503 1.00 0.00 O1 H

ATOM 2844 O21 POPCO 22 -11.942 -21.250 15.070 1.00 0.00 O1 O

ATOM 2845 C21 POPCO 22 -12.384 -22.073 14.120 1.00 0.00 O1 C

ATOM 2846 O22 POPCO 22 -12.808 -23.235 14.275 1.00 0.00 O1 O

ATOM 2847 C22 POPCO 22 -12.388 -21.367 12.854 1.00 0.00 O1 C

ATOM 2848 H2R POPCO 22 -13.200 -20.525 12.824 1.00 0.00 O1 H

ATOM 2849 H2S POPCO 22 -11.353 -20.892 12.792 1.00 0.00 O1 H

ATOM 2850 C3 POPCO 22 -13.386 -20.401 16.819 1.00 0.00 O1 C

ATOM 2851 HX POPCO 22 -14.232 -20.451 16.065 1.00 0.00 O1 H

ATOM 2852 HY POPCO 22 -13.900 -20.459 17.818 1.00 0.00 O1 H

ATOM 2853 O31 POPCO 22 -12.886 -19.064 16.579 1.00 0.00 O1 O

ATOM 2854 C31 POPCO 22 -13.652 -18.130 17.033 1.00 0.00 O1 C

ATOM 2855 O32 POPCO 22 -14.527 -18.242 17.826 1.00 0.00 O1 O

ATOM 2856 C32 POPCO 22 -13.267 -16.800 16.421 1.00 0.00 O1 C

ATOM 2857 H2X POPCO 22 -14.070 -16.129 16.650 1.00 0.00 O1 H

ATOM 2858 H2Y POPCO 22 -12.425 -16.280 16.834 1.00 0.00 O1 H

ATOM 2859 C23 POPCO 22 -12.538 -22.226 11.631 1.00 0.00 O1 C

ATOM 2860 H3R POPCO 22 -11.924 -23.161 11.718 1.00 0.00 O1 H

ATOM 2861 H3S POPCO 22 -13.603 -22.508 11.550 1.00 0.00 O1 H

ATOM 2862 C24 POPCO 22 -12.075 -21.538 10.341 1.00 0.00 O1 C

ATOM 2863 H4R POPCO 22 -12.660 -20.575 10.270 1.00 0.00 O1 H

ATOM 2864 H4S POPCO 22 -11.054 -21.134 10.488 1.00 0.00 O1 H

ATOM 2865 C25 POPCO 22 -12.284 -22.306 9.077 1.00 0.00 O1 C

ATOM 2866 H5R POPCO 22 -11.764 -23.337 9.154 1.00 0.00 O1 H

ATOM 2867 H5S POPCO 22 -13.411 -22.531 8.910 1.00 0.00 O1 H

ATOM 2868 C26 POPCO 22 -11.830 -21.495 7.767 1.00 0.00 O1 C

ATOM 2869 H6R POPCO 22 -12.175 -20.500 7.920 1.00 0.00 O1 H

ATOM 2870 H6S POPCO 22 -10.669 -21.468 7.754 1.00 0.00 O1 H

ATOM 2871 C27 POPCO 22 -12.494 -21.969 6.513 1.00 0.00 O1 C

ATOM 2872 H7R POPCO 22 -12.082 -22.953 6.251 1.00 0.00 O1 H

ATOM 2873 H7S POPCO 22 -13.569 -22.113 6.593 1.00 0.00 O1 H

ATOM 2874 C28 POPCO 22 -12.375 -21.003 5.358 1.00 0.00 O1 C

ATOM 2875 H8R POPCO 22 -12.879 -20.037 5.683 1.00 0.00 O1 H

ATOM 2876 H8S POPCO 22 -11.305 -20.764 5.222 1.00 0.00 O1 H

ATOM 2877 C29 POPCO 22 -12.920 -21.440 4.046 1.00 0.00 O1 C

ATOM 2878 H91 POPCO 22 -14.019 -21.526 4.036 1.00 0.00 O1 H

ATOM 2879 C210 POPCO 22 -12.318 -21.977 2.895 1.00 0.00 O1 C

ATOM 2880 H101 POPCO 22 -13.070 -22.309 2.089 1.00 0.00 O1 H

ATOM 2881 C211 POPCO 22 -10.807 -22.157 2.544 1.00 0.00 O1 C

ATOM 2882 H11R POPCO 22 -10.240 -21.734 3.369 1.00 0.00 O1 H

ATOM 2883 H11S POPCO 22 -10.545 -23.225 2.729 1.00 0.00 O1 H

ATOM 2884 C212 POPCO 22 -10.288 -21.644 1.209 1.00 0.00 O1 C

ATOM 2885 H12R POPCO 22 -10.652 -20.517 1.086 1.00 0.00 O1 H

ATOM 2886 H12S POPCO 22 -9.219 -21.681 1.111 1.00 0.00 O1 H

ATOM 2887 C213 POPCO 22 -10.922 -22.414 0.062 1.00 0.00 O1 C

ATOM 2888 H13R POPCO 22 -10.845 -23.553 0.270 1.00 0.00 O1 H

ATOM 2889 H13S POPCO 22 -12.025 -22.060 0.051 1.00 0.00 O1 H

ATOM 2890 C214 POPCO 22 -10.054 -22.104 -1.208 1.00 0.00 O1 C

ATOM 2891 H14R POPCO 22 -9.396 -21.161 -1.025 1.00 0.00 O1 H

ATOM 2892 H14S POPCO 22 -9.378 -22.977 -1.505 1.00 0.00 O1 H

ATOM 2893 C215 POPCO 22 -10.884 -21.790 -2.464 1.00 0.00 O1 C

ATOM 2894 H15R POPCO 22 -11.522 -22.695 -2.522 1.00 0.00 O1 H

ATOM 2895 H15S POPCO 22 -11.449 -20.829 -2.369 1.00 0.00 O1 H

ATOM 2896 C216 POPCO 22 -9.943 -21.758 -3.647 1.00 0.00 O1 C

ATOM 2897 H16R POPCO 22 -9.221 -20.975 -3.586 1.00 0.00 O1 H

ATOM 2898 H16S POPCO 22 -9.328 -22.666 -3.669 1.00 0.00 O1 H

ATOM 2899 C217 POPCO 22 -10.732 -21.460 -4.932 1.00 0.00 O1 C

ATOM 2900 H17R POPCO 22 -11.191 -22.339 -5.408 1.00 0.00 O1 H

ATOM 2901 H17S POPCO 22 -11.431 -20.718 -4.710 1.00 0.00 O1 H

ATOM 2902 C218 POPCO 22 -9.820 -20.894 -5.966 1.00 0.00 O1 C

ATOM 2903 H18R POPCO 22 -10.226 -21.040 -6.985 1.00 0.00 O1 H

ATOM 2904 H18S POPCO 22 -9.688 -19.866 -5.797 1.00 0.00 O1 H

ATOM 2905 H18T POPCO 22 -8.850 -21.492 -6.021 1.00 0.00 O1 H

ATOM 2906 C33 POPCO 22 -13.172 -16.881 14.824 1.00 0.00 O1 C

ATOM 2907 H3X POPCO 22 -14.053 -17.414 14.320 1.00 0.00 O1 H

ATOM 2908 H3Y POPCO 22 -13.349 -15.863 14.492 1.00 0.00 O1 H

ATOM 2909 C34 POPCO 22 -11.934 -17.402 14.141 1.00 0.00 O1 C

ATOM 2910 H4X POPCO 22 -11.045 -16.797 14.451 1.00 0.00 O1 H

ATOM 2911 H4Y POPCO 22 -11.685 -18.402 14.427 1.00 0.00 O1 H

ATOM 2912 C35 POPCO 22 -12.160 -17.431 12.612 1.00 0.00 O1 C

ATOM 2913 H5X POPCO 22 -13.093 -18.083 12.410 1.00 0.00 O1 H

ATOM 2914 H5Y POPCO 22 -12.459 -16.367 12.341 1.00 0.00 O1 H

ATOM 2915 C36 POPCO 22 -10.937 -17.886 11.908 1.00 0.00 O1 C

ATOM 2916 H6X POPCO 22 -10.053 -17.721 12.547 1.00 0.00 O1 H

ATOM 2917 H6Y POPCO 22 -11.021 -18.942 11.916 1.00 0.00 O1 H

ATOM 2918 C37 POPCO 22 -10.723 -17.318 10.536 1.00 0.00 O1 C

ATOM 2919 H7X POPCO 22 -11.657 -17.044 10.018 1.00 0.00 O1 H

ATOM 2920 H7Y POPCO 22 -10.091 -16.371 10.667 1.00 0.00 O1 H

ATOM 2921 C38 POPCO 22 -10.006 -18.232 9.521 1.00 0.00 O1 C

ATOM 2922 H8X POPCO 22 -9.224 -18.803 10.032 1.00 0.00 O1 H

ATOM 2923 H8Y POPCO 22 -10.630 -19.034 9.327 1.00 0.00 O1 H

ATOM 2924 C39 POPCO 22 -9.505 -17.647 8.166 1.00 0.00 O1 C

ATOM 2925 H9X POPCO 22 -10.262 -16.953 7.839 1.00 0.00 O1 H

ATOM 2926 H9Y POPCO 22 -8.583 -17.073 8.299 1.00 0.00 O1 H

ATOM 2927 C310 POPCO 22 -9.182 -18.757 7.147 1.00 0.00 O1 C

ATOM 2928 H10X POPCO 22 -8.615 -19.604 7.543 1.00 0.00 O1 H

ATOM 2929 H10Y POPCO 22 -10.133 -19.231 6.838 1.00 0.00 O1 H

ATOM 2930 C311 POPCO 22 -8.516 -18.122 5.889 1.00 0.00 O1 C

ATOM 2931 H11X POPCO 22 -9.291 -17.426 5.424 1.00 0.00 O1 H

ATOM 2932 H11Y POPCO 22 -7.638 -17.562 6.268 1.00 0.00 O1 H

ATOM 2933 C312 POPCO 22 -8.012 -19.259 4.983 1.00 0.00 O1 C

ATOM 2934 H12X POPCO 22 -7.322 -19.956 5.568 1.00 0.00 O1 H

ATOM 2935 H12Y POPCO 22 -8.820 -19.859 4.597 1.00 0.00 O1 H

ATOM 2936 C313 POPCO 22 -7.339 -18.698 3.655 1.00 0.00 O1 C

ATOM 2937 H13X POPCO 22 -8.142 -18.024 3.297 1.00 0.00 O1 H

ATOM 2938 H13Y POPCO 22 -6.318 -18.258 3.812 1.00 0.00 O1 H

ATOM 2939 C314 POPCO 22 -7.158 -19.574 2.438 1.00 0.00 O1 C

ATOM 2940 H14X POPCO 22 -6.394 -20.358 2.643 1.00 0.00 O1 H

ATOM 2941 H14Y POPCO 22 -8.131 -20.005 2.352 1.00 0.00 O1 H

ATOM 2942 C315 POPCO 22 -6.761 -18.720 1.205 1.00 0.00 O1 C

ATOM 2943 H15X POPCO 22 -7.550 -18.062 0.887 1.00 0.00 O1 H

ATOM 2944 H15Y POPCO 22 -5.850 -18.119 1.494 1.00 0.00 O1 H

ATOM 2945 C316 POPCO 22 -6.259 -19.500 -0.004 1.00 0.00 O1 C

ATOM 2946 H16X POPCO 22 -5.281 -19.952 0.240 1.00 0.00 O1 H

ATOM 2947 H16Y POPCO 22 -6.860 -20.410 -0.143 1.00 0.00 O1 H

ATOM 2948 H16Z POPCO 22 -6.262 -18.890 -0.910 1.00 0.00 O1 H

ATOM 2949 N POPCO 23 -16.401 -12.589 17.994 1.00 0.00 O1 N

ATOM 2950 C12 POPCO 23 -15.781 -11.252 17.733 1.00 0.00 O1 C

ATOM 2951 H12A POPCO 23 -15.519 -10.837 18.582 1.00 0.00 O1 H

ATOM 2952 H12B POPCO 23 -16.647 -10.693 17.300 1.00 0.00 O1 H

ATOM 2953 C13 POPCO 23 -15.561 -13.377 18.887 1.00 0.00 O1 C

ATOM 2954 H13A POPCO 23 -15.153 -12.864 19.688 1.00 0.00 O1 H

ATOM 2955 H13B POPCO 23 -14.682 -13.754 18.392 1.00 0.00 O1 H

ATOM 2956 H13C POPCO 23 -16.067 -14.266 19.247 1.00 0.00 O1 H

ATOM 2957 C14 POPCO 23 -17.700 -12.452 18.721 1.00 0.00 O1 C

ATOM 2958 H14A POPCO 23 -18.484 -12.009 18.100 1.00 0.00 O1 H

ATOM 2959 H14B POPCO 23 -17.580 -11.706 19.516 1.00 0.00 O1 H

ATOM 2960 H14C POPCO 23 -18.077 -13.433 18.948 1.00 0.00 O1 H

ATOM 2961 C15 POPCO 23 -16.612 -13.374 16.728 1.00 0.00 O1 C

ATOM 2962 H15A POPCO 23 -17.300 -12.852 16.118 1.00 0.00 O1 H

ATOM 2963 H15B POPCO 23 -15.664 -13.573 16.266 1.00 0.00 O1 H

ATOM 2964 H15C POPCO 23 -17.134 -14.261 16.888 1.00 0.00 O1 H

ATOM 2965 C11 POPCO 23 -14.608 -11.176 16.831 1.00 0.00 O1 C

ATOM 2966 H11A POPCO 23 -14.830 -11.367 15.702 1.00 0.00 O1 H

ATOM 2967 H11B POPCO 23 -14.198 -10.154 16.790 1.00 0.00 O1 H

ATOM 2968 P POPCO 23 -12.608 -13.028 16.826 1.00 0.00 O1 P

ATOM 2969 O13 POPCO 23 -12.626 -14.040 17.842 1.00 0.00 O1 O

ATOM 2970 O14 POPCO 23 -11.365 -12.590 16.273 1.00 0.00 O1 O

ATOM 2971 O12 POPCO 23 -13.474 -11.844 17.324 1.00 0.00 O1 O

ATOM 2972 O11 POPCO 23 -13.486 -13.491 15.600 1.00 0.00 O1 O

ATOM 2973 C1 POPCO 23 -13.005 -13.188 14.269 1.00 0.00 O1 C

ATOM 2974 HA POPCO 23 -12.660 -12.077 14.228 1.00 0.00 O1 H

ATOM 2975 HB POPCO 23 -12.203 -13.885 13.985 1.00 0.00 O1 H

ATOM 2976 C2 POPCO 23 -14.092 -13.374 13.185 1.00 0.00 O1 C

ATOM 2977 HS POPCO 23 -14.410 -14.442 13.332 1.00 0.00 O1 H

ATOM 2978 O21 POPCO 23 -13.586 -13.076 11.893 1.00 0.00 O1 O

ATOM 2979 C21 POPCO 23 -14.149 -13.794 10.798 1.00 0.00 O1 C

ATOM 2980 O22 POPCO 23 -14.853 -14.785 10.928 1.00 0.00 O1 O

ATOM 2981 C22 POPCO 23 -13.712 -13.187 9.466 1.00 0.00 O1 C

ATOM 2982 H2R POPCO 23 -13.582 -12.050 9.592 1.00 0.00 O1 H

ATOM 2983 H2S POPCO 23 -12.727 -13.603 9.173 1.00 0.00 O1 H

ATOM 2984 C3 POPCO 23 -15.573 -12.652 13.275 1.00 0.00 O1 C

ATOM 2985 HX POPCO 23 -16.297 -13.225 12.801 1.00 0.00 O1 H

ATOM 2986 HY POPCO 23 -15.625 -12.804 14.354 1.00 0.00 O1 H

ATOM 2987 O31 POPCO 23 -15.558 -11.255 12.886 1.00 0.00 O1 O

ATOM 2988 C31 POPCO 23 -16.367 -10.469 13.537 1.00 0.00 O1 C

ATOM 2989 O32 POPCO 23 -17.031 -10.791 14.468 1.00 0.00 O1 O

ATOM 2990 C32 POPCO 23 -16.171 -8.993 13.115 1.00 0.00 O1 C

ATOM 2991 H2X POPCO 23 -16.674 -8.326 13.868 1.00 0.00 O1 H

ATOM 2992 H2Y POPCO 23 -15.137 -8.771 13.104 1.00 0.00 O1 H

ATOM 2993 C23 POPCO 23 -14.706 -13.520 8.332 1.00 0.00 O1 C

ATOM 2994 H3R POPCO 23 -14.702 -14.602 8.165 1.00 0.00 O1 H

ATOM 2995 H3S POPCO 23 -15.688 -13.178 8.662 1.00 0.00 O1 H

ATOM 2996 C24 POPCO 23 -14.238 -12.844 7.028 1.00 0.00 O1 C

ATOM 2997 H4R POPCO 23 -14.337 -11.762 7.119 1.00 0.00 O1 H

ATOM 2998 H4S POPCO 23 -13.147 -13.026 6.958 1.00 0.00 O1 H

ATOM 2999 C25 POPCO 23 -14.986 -13.438 5.823 1.00 0.00 O1 C

ATOM 3000 H5R POPCO 23 -14.956 -14.566 5.969 1.00 0.00 O1 H

ATOM 3001 H5S POPCO 23 -15.990 -13.100 6.026 1.00 0.00 O1 H

ATOM 3002 C26 POPCO 23 -14.485 -13.126 4.470 1.00 0.00 O1 C

ATOM 3003 H6R POPCO 23 -14.689 -12.060 4.204 1.00 0.00 O1 H

ATOM 3004 H6S POPCO 23 -13.391 -13.188 4.375 1.00 0.00 O1 H

ATOM 3005 C27 POPCO 23 -15.109 -13.870 3.196 1.00 0.00 O1 C

ATOM 3006 H7R POPCO 23 -14.817 -14.908 3.169 1.00 0.00 O1 H

ATOM 3007 H7S POPCO 23 -16.247 -13.719 3.480 1.00 0.00 O1 H

ATOM 3008 C28 POPCO 23 -15.045 -12.969 1.893 1.00 0.00 O1 C

ATOM 3009 H8R POPCO 23 -15.739 -12.126 2.013 1.00 0.00 O1 H

ATOM 3010 H8S POPCO 23 -13.997 -12.547 1.894 1.00 0.00 O1 H

ATOM 3011 C29 POPCO 23 -15.383 -13.782 0.696 1.00 0.00 O1 C

ATOM 3012 H91 POPCO 23 -16.176 -14.572 0.798 1.00 0.00 O1 H

ATOM 3013 C210 POPCO 23 -14.889 -13.601 -0.498 1.00 0.00 O1 C

ATOM 3014 H101 POPCO 23 -15.184 -14.232 -1.335 1.00 0.00 O1 H

ATOM 3015 C211 POPCO 23 -13.811 -12.611 -0.946 1.00 0.00 O1 C

ATOM 3016 H11R POPCO 23 -13.260 -12.054 -0.110 1.00 0.00 O1 H

ATOM 3017 H11S POPCO 23 -13.006 -13.326 -1.363 1.00 0.00 O1 H

ATOM 3018 C212 POPCO 23 -14.243 -11.590 -2.010 1.00 0.00 O1 C

ATOM 3019 H12R POPCO 23 -15.250 -11.250 -1.758 1.00 0.00 O1 H

ATOM 3020 H12S POPCO 23 -13.576 -10.731 -1.985 1.00 0.00 O1 H

ATOM 3021 C213 POPCO 23 -14.370 -12.133 -3.481 1.00 0.00 O1 C

ATOM 3022 H13R POPCO 23 -13.378 -12.701 -3.684 1.00 0.00 O1 H

ATOM 3023 H13S POPCO 23 -15.216 -12.910 -3.543 1.00 0.00 O1 H

ATOM 3024 C214 POPCO 23 -14.620 -11.073 -4.506 1.00 0.00 O1 C

ATOM 3025 H14R POPCO 23 -15.639 -10.695 -4.481 1.00 0.00 O1 H

ATOM 3026 H14S POPCO 23 -13.979 -10.239 -4.239 1.00 0.00 O1 H

ATOM 3027 C215 POPCO 23 -14.435 -11.621 -5.890 1.00 0.00 O1 C

ATOM 3028 H15R POPCO 23 -13.430 -12.112 -6.101 1.00 0.00 O1 H

ATOM 3029 H15S POPCO 23 -15.226 -12.497 -5.935 1.00 0.00 O1 H

ATOM 3030 C216 POPCO 23 -14.800 -10.595 -6.938 1.00 0.00 O1 C

ATOM 3031 H16R POPCO 23 -15.694 -9.954 -6.559 1.00 0.00 O1 H

ATOM 3032 H16S POPCO 23 -13.987 -9.786 -7.030 1.00 0.00 O1 H

ATOM 3033 C217 POPCO 23 -15.182 -11.193 -8.288 1.00 0.00 O1 C

ATOM 3034 H17R POPCO 23 -14.277 -11.673 -8.712 1.00 0.00 O1 H

ATOM 3035 H17S POPCO 23 -16.022 -11.896 -8.126 1.00 0.00 O1 H

ATOM 3036 C218 POPCO 23 -15.621 -10.009 -9.204 1.00 0.00 O1 C

ATOM 3037 H18R POPCO 23 -15.941 -10.416 -10.122 1.00 0.00 O1 H

ATOM 3038 H18S POPCO 23 -16.445 -9.371 -8.737 1.00 0.00 O1 H

ATOM 3039 H18T POPCO 23 -14.826 -9.196 -9.290 1.00 0.00 O1 H

ATOM 3040 C33 POPCO 23 -16.871 -8.633 11.836 1.00 0.00 O1 C

ATOM 3041 H3X POPCO 23 -17.933 -8.813 11.903 1.00 0.00 O1 H

ATOM 3042 H3Y POPCO 23 -16.787 -7.560 11.703 1.00 0.00 O1 H

ATOM 3043 C34 POPCO 23 -16.057 -9.205 10.674 1.00 0.00 O1 C

ATOM 3044 H4X POPCO 23 -14.990 -8.992 10.779 1.00 0.00 O1 H

ATOM 3045 H4Y POPCO 23 -16.191 -10.328 10.482 1.00 0.00 O1 H

ATOM 3046 C35 POPCO 23 -16.497 -8.538 9.350 1.00 0.00 O1 C

ATOM 3047 H5X POPCO 23 -17.596 -8.445 9.128 1.00 0.00 O1 H

ATOM 3048 H5Y POPCO 23 -16.083 -7.471 9.368 1.00 0.00 O1 H

ATOM 3049 C36 POPCO 23 -15.727 -9.302 8.174 1.00 0.00 O1 C

ATOM 3050 H6X POPCO 23 -14.648 -9.317 8.330 1.00 0.00 O1 H

ATOM 3051 H6Y POPCO 23 -16.289 -10.310 8.184 1.00 0.00 O1 H

ATOM 3052 C37 POPCO 23 -15.967 -8.570 6.874 1.00 0.00 O1 C

ATOM 3053 H7X POPCO 23 -17.057 -8.462 6.762 1.00 0.00 O1 H

ATOM 3054 H7Y POPCO 23 -15.479 -7.536 6.781 1.00 0.00 O1 H

ATOM 3055 C38 POPCO 23 -15.586 -9.289 5.583 1.00 0.00 O1 C

ATOM 3056 H8X POPCO 23 -14.493 -9.481 5.641 1.00 0.00 O1 H

ATOM 3057 H8Y POPCO 23 -16.253 -10.199 5.554 1.00 0.00 O1 H

ATOM 3058 C39 POPCO 23 -15.812 -8.580 4.345 1.00 0.00 O1 C

ATOM 3059 H9X POPCO 23 -16.910 -8.270 4.345 1.00 0.00 O1 H

ATOM 3060 H9Y POPCO 23 -15.098 -7.746 4.279 1.00 0.00 O1 H

ATOM 3061 C310 POPCO 23 -15.719 -9.394 2.990 1.00 0.00 O1 C

ATOM 3062 H10X POPCO 23 -14.694 -9.856 2.973 1.00 0.00 O1 H

ATOM 3063 H10Y POPCO 23 -16.608 -10.121 3.133 1.00 0.00 O1 H

ATOM 3064 C311 POPCO 23 -15.757 -8.461 1.832 1.00 0.00 O1 C

ATOM 3065 H11X POPCO 23 -16.730 -7.846 2.023 1.00 0.00 O1 H

ATOM 3066 H11Y POPCO 23 -14.832 -7.866 1.810 1.00 0.00 O1 H

ATOM 3067 C312 POPCO 23 -15.919 -9.131 0.441 1.00 0.00 O1 C

ATOM 3068 H12X POPCO 23 -15.082 -9.804 0.305 1.00 0.00 O1 H

ATOM 3069 H12Y POPCO 23 -16.837 -9.765 0.517 1.00 0.00 O1 H

ATOM 3070 C313 POPCO 23 -16.078 -7.986 -0.641 1.00 0.00 O1 C

ATOM 3071 H13X POPCO 23 -17.050 -7.500 -0.496 1.00 0.00 O1 H

ATOM 3072 H13Y POPCO 23 -15.331 -7.144 -0.463 1.00 0.00 O1 H

ATOM 3073 C314 POPCO 23 -15.922 -8.380 -2.079 1.00 0.00 O1 C

ATOM 3074 H14X POPCO 23 -14.900 -8.682 -2.292 1.00 0.00 O1 H

ATOM 3075 H14Y POPCO 23 -16.578 -9.240 -2.396 1.00 0.00 O1 H

ATOM 3076 C315 POPCO 23 -16.217 -7.305 -3.096 1.00 0.00 O1 C

ATOM 3077 H15X POPCO 23 -17.283 -6.964 -3.089 1.00 0.00 O1 H

ATOM 3078 H15Y POPCO 23 -15.620 -6.428 -2.775 1.00 0.00 O1 H

ATOM 3079 C316 POPCO 23 -15.820 -7.679 -4.545 1.00 0.00 O1 C

ATOM 3080 H16X POPCO 23 -16.671 -8.285 -4.958 1.00 0.00 O1 H

ATOM 3081 H16Y POPCO 23 -15.669 -6.751 -5.190 1.00 0.00 O1 H

ATOM 3082 H16Z POPCO 23 -14.843 -8.213 -4.620 1.00 0.00 O1 H

ATOM 3083 N POPCO 24 -13.655 -3.033 21.769 1.00 0.00 O1 N

ATOM 3084 C12 POPCO 24 -14.432 -2.465 20.578 1.00 0.00 O1 C

ATOM 3085 H12A POPCO 24 -15.068 -1.689 20.994 1.00 0.00 O1 H

ATOM 3086 H12B POPCO 24 -15.087 -3.279 20.273 1.00 0.00 O1 H

ATOM 3087 C13 POPCO 24 -13.209 -1.900 22.694 1.00 0.00 O1 C

ATOM 3088 H13A POPCO 24 -14.092 -1.261 22.873 1.00 0.00 O1 H

ATOM 3089 H13B POPCO 24 -12.453 -1.351 22.261 1.00 0.00 O1 H

ATOM 3090 H13C POPCO 24 -12.843 -2.182 23.631 1.00 0.00 O1 H

ATOM 3091 C14 POPCO 24 -14.466 -3.873 22.544 1.00 0.00 O1 C

ATOM 3092 H14A POPCO 24 -14.896 -4.607 21.830 1.00 0.00 O1 H

ATOM 3093 H14B POPCO 24 -15.298 -3.452 23.198 1.00 0.00 O1 H

ATOM 3094 H14C POPCO 24 -13.783 -4.450 23.158 1.00 0.00 O1 H

ATOM 3095 C15 POPCO 24 -12.468 -3.760 21.179 1.00 0.00 O1 C

ATOM 3096 H15A POPCO 24 -12.794 -4.533 20.523 1.00 0.00 O1 H

ATOM 3097 H15B POPCO 24 -11.878 -3.026 20.599 1.00 0.00 O1 H

ATOM 3098 H15C POPCO 24 -11.831 -4.016 21.916 1.00 0.00 O1 H

ATOM 3099 C11 POPCO 24 -13.747 -1.854 19.375 1.00 0.00 O1 C

ATOM 3100 H11A POPCO 24 -13.125 -2.679 18.921 1.00 0.00 O1 H

ATOM 3101 H11B POPCO 24 -14.487 -1.540 18.636 1.00 0.00 O1 H

ATOM 3102 P POPCO 24 -11.488 -0.443 19.879 1.00 0.00 O1 P

ATOM 3103 O13 POPCO 24 -11.140 -0.590 21.248 1.00 0.00 O1 O

ATOM 3104 O14 POPCO 24 -11.156 0.864 19.221 1.00 0.00 O1 O

ATOM 3105 O12 POPCO 24 -13.096 -0.674 19.806 1.00 0.00 O1 O

ATOM 3106 O11 POPCO 24 -10.812 -1.655 19.008 1.00 0.00 O1 O

ATOM 3107 C1 POPCO 24 -10.901 -1.582 17.586 1.00 0.00 O1 C

ATOM 3108 HA POPCO 24 -11.919 -1.765 17.302 1.00 0.00 O1 H

ATOM 3109 HB POPCO 24 -10.582 -0.608 17.247 1.00 0.00 O1 H

ATOM 3110 C2 POPCO 24 -10.208 -2.783 16.861 1.00 0.00 O1 C

ATOM 3111 HS POPCO 24 -9.130 -2.584 16.840 1.00 0.00 O1 H

ATOM 3112 O21 POPCO 24 -10.708 -2.822 15.468 1.00 0.00 O1 O

ATOM 3113 C21 POPCO 24 -9.844 -2.622 14.461 1.00 0.00 O1 C

ATOM 3114 O22 POPCO 24 -8.642 -2.629 14.597 1.00 0.00 O1 O

ATOM 3115 C22 POPCO 24 -10.706 -2.424 13.131 1.00 0.00 O1 C

ATOM 3116 H2R POPCO 24 -11.114 -3.396 12.869 1.00 0.00 O1 H

ATOM 3117 H2S POPCO 24 -11.571 -1.780 13.342 1.00 0.00 O1 H

ATOM 3118 C3 POPCO 24 -10.508 -4.112 17.629 1.00 0.00 O1 C

ATOM 3119 HX POPCO 24 -9.844 -4.887 17.181 1.00 0.00 O1 H

ATOM 3120 HY POPCO 24 -10.407 -4.066 18.721 1.00 0.00 O1 H

ATOM 3121 O31 POPCO 24 -11.807 -4.588 17.293 1.00 0.00 O1 O

ATOM 3122 C31 POPCO 24 -12.443 -5.459 18.050 1.00 0.00 O1 C

ATOM 3123 O32 POPCO 24 -12.097 -5.956 19.105 1.00 0.00 O1 O

ATOM 3124 C32 POPCO 24 -13.678 -5.956 17.315 1.00 0.00 O1 C

ATOM 3125 H2X POPCO 24 -14.029 -6.863 17.940 1.00 0.00 O1 H

ATOM 3126 H2Y POPCO 24 -14.412 -5.126 17.323 1.00 0.00 O1 H

ATOM 3127 C23 POPCO 24 -9.856 -1.904 11.955 1.00 0.00 O1 C

ATOM 3128 H3R POPCO 24 -9.615 -0.852 12.036 1.00 0.00 O1 H

ATOM 3129 H3S POPCO 24 -8.943 -2.517 11.801 1.00 0.00 O1 H

ATOM 3130 C24 POPCO 24 -10.652 -1.902 10.632 1.00 0.00 O1 C

ATOM 3131 H4R POPCO 24 -10.715 -2.947 10.362 1.00 0.00 O1 H

ATOM 3132 H4S POPCO 24 -11.607 -1.404 10.916 1.00 0.00 O1 H

ATOM 3133 C25 POPCO 24 -10.105 -1.064 9.415 1.00 0.00 O1 C

ATOM 3134 H5R POPCO 24 -9.843 -0.067 9.761 1.00 0.00 O1 H

ATOM 3135 H5S POPCO 24 -9.167 -1.613 9.176 1.00 0.00 O1 H

ATOM 3136 C26 POPCO 24 -11.061 -0.981 8.166 1.00 0.00 O1 C

ATOM 3137 H6R POPCO 24 -11.167 -2.057 7.837 1.00 0.00 O1 H

ATOM 3138 H6S POPCO 24 -12.038 -0.669 8.483 1.00 0.00 O1 H

ATOM 3139 C27 POPCO 24 -10.488 -0.143 6.901 1.00 0.00 O1 C

ATOM 3140 H7R POPCO 24 -10.382 0.913 7.130 1.00 0.00 O1 H

ATOM 3141 H7S POPCO 24 -9.466 -0.635 6.664 1.00 0.00 O1 H

ATOM 3142 C28 POPCO 24 -11.485 -0.374 5.658 1.00 0.00 O1 C

ATOM 3143 H8R POPCO 24 -11.616 -1.458 5.672 1.00 0.00 O1 H

ATOM 3144 H8S POPCO 24 -12.453 0.150 6.050 1.00 0.00 O1 H

ATOM 3145 C29 POPCO 24 -11.098 0.232 4.340 1.00 0.00 O1 C

ATOM 3146 H91 POPCO 24 -9.997 0.324 4.213 1.00 0.00 O1 H

ATOM 3147 C210 POPCO 24 -11.967 0.672 3.380 1.00 0.00 O1 C

ATOM 3148 H101 POPCO 24 -11.639 1.084 2.434 1.00 0.00 O1 H

ATOM 3149 C211 POPCO 24 -13.427 0.590 3.401 1.00 0.00 O1 C

ATOM 3150 H11R POPCO 24 -13.747 0.280 4.479 1.00 0.00 O1 H

ATOM 3151 H11S POPCO 24 -13.797 1.626 3.152 1.00 0.00 O1 H

ATOM 3152 C212 POPCO 24 -13.967 -0.429 2.419 1.00 0.00 O1 C

ATOM 3153 H12R POPCO 24 -13.427 -1.374 2.609 1.00 0.00 O1 H

ATOM 3154 H12S POPCO 24 -14.992 -0.608 2.616 1.00 0.00 O1 H

ATOM 3155 C213 POPCO 24 -13.839 -0.007 0.971 1.00 0.00 O1 C

ATOM 3156 H13R POPCO 24 -14.408 0.931 0.967 1.00 0.00 O1 H

ATOM 3157 H13S POPCO 24 -12.784 0.218 0.916 1.00 0.00 O1 H

ATOM 3158 C214 POPCO 24 -14.314 -0.886 -0.107 1.00 0.00 O1 C

ATOM 3159 H14R POPCO 24 -13.661 -1.814 0.044 1.00 0.00 O1 H

ATOM 3160 H14S POPCO 24 -15.371 -1.180 0.049 1.00 0.00 O1 H

ATOM 3161 C215 POPCO 24 -14.100 -0.396 -1.519 1.00 0.00 O1 C

ATOM 3162 H15R POPCO 24 -14.773 0.553 -1.781 1.00 0.00 O1 H

ATOM 3163 H15S POPCO 24 -13.029 -0.164 -1.722 1.00 0.00 O1 H

ATOM 3164 C216 POPCO 24 -14.325 -1.524 -2.628 1.00 0.00 O1 C

ATOM 3165 H16R POPCO 24 -13.714 -2.472 -2.344 1.00 0.00 O1 H

ATOM 3166 H16S POPCO 24 -15.406 -1.843 -2.535 1.00 0.00 O1 H

ATOM 3167 C217 POPCO 24 -14.153 -1.077 -4.102 1.00 0.00 O1 C

ATOM 3168 H17R POPCO 24 -14.892 -0.284 -4.443 1.00 0.00 O1 H

ATOM 3169 H17S POPCO 24 -13.225 -0.503 -4.090 1.00 0.00 O1 H

ATOM 3170 C218 POPCO 24 -14.159 -2.135 -5.167 1.00 0.00 O1 C

ATOM 3171 H18R POPCO 24 -14.113 -1.657 -6.201 1.00 0.00 O1 H

ATOM 3172 H18S POPCO 24 -13.259 -2.790 -5.076 1.00 0.00 O1 H

ATOM 3173 H18T POPCO 24 -15.072 -2.778 -5.085 1.00 0.00 O1 H

ATOM 3174 C33 POPCO 24 -13.508 -6.289 15.792 1.00 0.00 O1 C

ATOM 3175 H3X POPCO 24 -12.606 -6.919 15.596 1.00 0.00 O1 H

ATOM 3176 H3Y POPCO 24 -14.357 -6.926 15.466 1.00 0.00 O1 H

ATOM 3177 C34 POPCO 24 -13.736 -5.071 14.801 1.00 0.00 O1 C

ATOM 3178 H4X POPCO 24 -14.741 -4.742 14.954 1.00 0.00 O1 H

ATOM 3179 H4Y POPCO 24 -12.947 -4.265 14.918 1.00 0.00 O1 H

ATOM 3180 C35 POPCO 24 -13.631 -5.476 13.315 1.00 0.00 O1 C

ATOM 3181 H5X POPCO 24 -12.600 -5.807 13.172 1.00 0.00 O1 H

ATOM 3182 H5Y POPCO 24 -14.250 -6.398 13.207 1.00 0.00 O1 H

ATOM 3183 C36 POPCO 24 -14.113 -4.439 12.349 1.00 0.00 O1 C

ATOM 3184 H6X POPCO 24 -14.897 -3.749 12.785 1.00 0.00 O1 H

ATOM 3185 H6Y POPCO 24 -13.161 -3.852 12.162 1.00 0.00 O1 H

ATOM 3186 C37 POPCO 24 -14.529 -5.068 10.978 1.00 0.00 O1 C

ATOM 3187 H7X POPCO 24 -13.956 -5.966 10.735 1.00 0.00 O1 H

ATOM 3188 H7Y POPCO 24 -15.621 -5.393 11.107 1.00 0.00 O1 H

ATOM 3189 C38 POPCO 24 -14.401 -4.019 9.857 1.00 0.00 O1 C

ATOM 3190 H8X POPCO 24 -15.083 -3.141 10.069 1.00 0.00 O1 H

ATOM 3191 H8Y POPCO 24 -13.386 -3.548 9.889 1.00 0.00 O1 H

ATOM 3192 C39 POPCO 24 -14.595 -4.744 8.531 1.00 0.00 O1 C

ATOM 3193 H9X POPCO 24 -13.843 -5.534 8.361 1.00 0.00 O1 H

ATOM 3194 H9Y POPCO 24 -15.637 -5.058 8.330 1.00 0.00 O1 H

ATOM 3195 C310 POPCO 24 -14.226 -3.804 7.386 1.00 0.00 O1 C

ATOM 3196 H10X POPCO 24 -15.082 -3.125 7.203 1.00 0.00 O1 H

ATOM 3197 H10Y POPCO 24 -13.243 -3.333 7.599 1.00 0.00 O1 H

ATOM 3198 C311 POPCO 24 -14.134 -4.614 6.085 1.00 0.00 O1 C

ATOM 3199 H11X POPCO 24 -13.185 -5.215 5.989 1.00 0.00 O1 H

ATOM 3200 H11Y POPCO 24 -14.990 -5.306 6.078 1.00 0.00 O1 H

ATOM 3201 C312 POPCO 24 -14.220 -3.814 4.734 1.00 0.00 O1 C

ATOM 3202 H12X POPCO 24 -14.923 -3.002 4.787 1.00 0.00 O1 H

ATOM 3203 H12Y POPCO 24 -13.253 -3.386 4.534 1.00 0.00 O1 H

ATOM 3204 C313 POPCO 24 -14.680 -4.696 3.648 1.00 0.00 O1 C

ATOM 3205 H13X POPCO 24 -13.976 -5.566 3.459 1.00 0.00 O1 H

ATOM 3206 H13Y POPCO 24 -15.660 -5.070 3.797 1.00 0.00 O1 H

ATOM 3207 C314 POPCO 24 -14.610 -3.988 2.231 1.00 0.00 O1 C

ATOM 3208 H14X POPCO 24 -15.210 -3.059 2.128 1.00 0.00 O1 H

ATOM 3209 H14Y POPCO 24 -13.551 -3.903 2.008 1.00 0.00 O1 H

ATOM 3210 C315 POPCO 24 -15.150 -4.859 1.096 1.00 0.00 O1 C

ATOM 3211 H15X POPCO 24 -14.539 -5.728 1.119 1.00 0.00 O1 H

ATOM 3212 H15Y POPCO 24 -16.206 -5.053 1.442 1.00 0.00 O1 H

ATOM 3213 C316 POPCO 24 -14.985 -4.430 -0.379 1.00 0.00 O1 C

ATOM 3214 H16X POPCO 24 -13.872 -4.390 -0.532 1.00 0.00 O1 H

ATOM 3215 H16Y POPCO 24 -15.568 -5.071 -1.044 1.00 0.00 O1 H

ATOM 3216 H16Z POPCO 24 -15.373 -3.440 -0.517 1.00 0.00 O1 H

ATOM 3217 N POPCO 25 -6.884 -41.015 20.375 1.00 0.00 O1 N

ATOM 3218 C12 POPCO 25 -6.195 -40.292 19.226 1.00 0.00 O1 C

ATOM 3219 H12A POPCO 25 -5.384 -39.849 19.681 1.00 0.00 O1 H

ATOM 3220 H12B POPCO 25 -6.984 -39.598 18.882 1.00 0.00 O1 H

ATOM 3221 C13 POPCO 25 -5.994 -41.928 21.165 1.00 0.00 O1 C

ATOM 3222 H13A POPCO 25 -5.298 -41.355 21.838 1.00 0.00 O1 H

ATOM 3223 H13B POPCO 25 -5.334 -42.446 20.488 1.00 0.00 O1 H

ATOM 3224 H13C POPCO 25 -6.529 -42.766 21.564 1.00 0.00 O1 H

ATOM 3225 C14 POPCO 25 -7.520 -40.017 21.271 1.00 0.00 O1 C

ATOM 3226 H14A POPCO 25 -8.231 -39.382 20.718 1.00 0.00 O1 H

ATOM 3227 H14B POPCO 25 -8.076 -40.309 22.137 1.00 0.00 O1 H

ATOM 3228 H14C POPCO 25 -6.709 -39.330 21.686 1.00 0.00 O1 H

ATOM 3229 C15 POPCO 25 -7.975 -41.837 19.709 1.00 0.00 O1 C

ATOM 3230 H15A POPCO 25 -8.656 -41.111 19.294 1.00 0.00 O1 H

ATOM 3231 H15B POPCO 25 -7.536 -42.528 19.044 1.00 0.00 O1 H

ATOM 3232 H15C POPCO 25 -8.466 -42.435 20.485 1.00 0.00 O1 H

ATOM 3233 C11 POPCO 25 -5.583 -41.063 18.017 1.00 0.00 O1 C

ATOM 3234 H11A POPCO 25 -6.426 -41.560 17.447 1.00 0.00 O1 H

ATOM 3235 H11B POPCO 25 -5.211 -40.357 17.287 1.00 0.00 O1 H

ATOM 3236 P POPCO 25 -4.563 -43.486 18.100 1.00 0.00 O1 P

ATOM 3237 O13 POPCO 25 -5.564 -44.096 19.048 1.00 0.00 O1 O

ATOM 3238 O14 POPCO 25 -3.152 -44.027 18.265 1.00 0.00 O1 O

ATOM 3239 O12 POPCO 25 -4.532 -41.948 18.360 1.00 0.00 O1 O

ATOM 3240 O11 POPCO 25 -5.162 -43.734 16.679 1.00 0.00 O1 O

ATOM 3241 C1 POPCO 25 -4.699 -43.191 15.538 1.00 0.00 O1 C

ATOM 3242 HA POPCO 25 -4.512 -42.081 15.682 1.00 0.00 O1 H

ATOM 3243 HB POPCO 25 -3.732 -43.690 15.241 1.00 0.00 O1 H

ATOM 3244 C2 POPCO 25 -5.672 -43.425 14.345 1.00 0.00 O1 C

ATOM 3245 HS POPCO 25 -5.606 -44.605 14.254 1.00 0.00 O1 H

ATOM 3246 O21 POPCO 25 -5.191 -42.791 13.122 1.00 0.00 O1 O

ATOM 3247 C21 POPCO 25 -5.469 -43.429 12.033 1.00 0.00 O1 C

ATOM 3248 O22 POPCO 25 -6.101 -44.504 11.934 1.00 0.00 O1 O

ATOM 3249 C22 POPCO 25 -4.909 -42.540 10.903 1.00 0.00 O1 C

ATOM 3250 H2R POPCO 25 -5.449 -41.549 10.751 1.00 0.00 O1 H

ATOM 3251 H2S POPCO 25 -3.893 -42.432 10.969 1.00 0.00 O1 H

ATOM 3252 C3 POPCO 25 -7.200 -43.126 14.605 1.00 0.00 O1 C

ATOM 3253 HX POPCO 25 -7.807 -43.685 13.847 1.00 0.00 O1 H

ATOM 3254 HY POPCO 25 -7.416 -43.498 15.651 1.00 0.00 O1 H

ATOM 3255 O31 POPCO 25 -7.331 -41.691 14.643 1.00 0.00 O1 O

ATOM 3256 C31 POPCO 25 -8.503 -41.230 14.370 1.00 0.00 O1 C

ATOM 3257 O32 POPCO 25 -9.492 -41.959 14.252 1.00 0.00 O1 O

ATOM 3258 C32 POPCO 25 -8.501 -39.744 14.242 1.00 0.00 O1 C

ATOM 3259 H2X POPCO 25 -9.186 -39.410 15.088 1.00 0.00 O1 H

ATOM 3260 H2Y POPCO 25 -7.514 -39.367 14.578 1.00 0.00 O1 H

ATOM 3261 C23 POPCO 25 -5.218 -43.336 9.586 1.00 0.00 O1 C

ATOM 3262 H3R POPCO 25 -4.723 -44.400 9.640 1.00 0.00 O1 H

ATOM 3263 H3S POPCO 25 -6.313 -43.451 9.439 1.00 0.00 O1 H

ATOM 3264 C24 POPCO 25 -4.736 -42.550 8.374 1.00 0.00 O1 C

ATOM 3265 H4R POPCO 25 -5.192 -41.586 8.401 1.00 0.00 O1 H

ATOM 3266 H4S POPCO 25 -3.610 -42.316 8.522 1.00 0.00 O1 H

ATOM 3267 C25 POPCO 25 -5.112 -43.329 7.179 1.00 0.00 O1 C

ATOM 3268 H5R POPCO 25 -4.441 -44.221 7.036 1.00 0.00 O1 H

ATOM 3269 H5S POPCO 25 -6.183 -43.721 7.165 1.00 0.00 O1 H

ATOM 3270 C26 POPCO 25 -4.966 -42.498 5.904 1.00 0.00 O1 C

ATOM 3271 H6R POPCO 25 -5.784 -41.692 5.738 1.00 0.00 O1 H

ATOM 3272 H6S POPCO 25 -3.996 -42.107 5.799 1.00 0.00 O1 H

ATOM 3273 C27 POPCO 25 -5.110 -43.552 4.765 1.00 0.00 O1 C

ATOM 3274 H7R POPCO 25 -4.266 -44.286 4.877 1.00 0.00 O1 H

ATOM 3275 H7S POPCO 25 -6.120 -44.096 4.860 1.00 0.00 O1 H

ATOM 3276 C28 POPCO 25 -5.010 -42.925 3.350 1.00 0.00 O1 C

ATOM 3277 H8R POPCO 25 -5.756 -42.069 3.333 1.00 0.00 O1 H

ATOM 3278 H8S POPCO 25 -4.083 -42.381 3.155 1.00 0.00 O1 H

ATOM 3279 C29 POPCO 25 -5.412 -43.858 2.183 1.00 0.00 O1 C

ATOM 3280 H91 POPCO 25 -6.337 -44.466 2.343 1.00 0.00 O1 H

ATOM 3281 C210 POPCO 25 -4.729 -43.985 1.006 1.00 0.00 O1 C

ATOM 3282 H101 POPCO 25 -5.118 -44.780 0.213 1.00 0.00 O1 H

ATOM 3283 C211 POPCO 25 -3.473 -43.307 0.472 1.00 0.00 O1 C

ATOM 3284 H11R POPCO 25 -2.915 -42.705 1.318 1.00 0.00 O1 H

ATOM 3285 H11S POPCO 25 -2.767 -44.052 0.119 1.00 0.00 O1 H

ATOM 3286 C212 POPCO 25 -3.704 -42.291 -0.668 1.00 0.00 O1 C

ATOM 3287 H12R POPCO 25 -4.521 -41.604 -0.389 1.00 0.00 O1 H

ATOM 3288 H12S POPCO 25 -2.778 -41.619 -0.643 1.00 0.00 O1 H

ATOM 3289 C213 POPCO 25 -3.921 -43.008 -1.941 1.00 0.00 O1 C

ATOM 3290 H13R POPCO 25 -2.995 -43.477 -2.216 1.00 0.00 O1 H

ATOM 3291 H13S POPCO 25 -4.784 -43.713 -1.818 1.00 0.00 O1 H

ATOM 3292 C214 POPCO 25 -4.163 -42.158 -3.162 1.00 0.00 O1 C

ATOM 3293 H14R POPCO 25 -5.110 -41.562 -3.016 1.00 0.00 O1 H

ATOM 3294 H14S POPCO 25 -3.335 -41.514 -3.205 1.00 0.00 O1 H

ATOM 3295 C215 POPCO 25 -4.348 -42.932 -4.519 1.00 0.00 O1 C

ATOM 3296 H15R POPCO 25 -3.563 -43.710 -4.787 1.00 0.00 O1 H

ATOM 3297 H15S POPCO 25 -5.365 -43.445 -4.308 1.00 0.00 O1 H

ATOM 3298 C216 POPCO 25 -4.562 -41.938 -5.663 1.00 0.00 O1 C

ATOM 3299 H16R POPCO 25 -5.524 -41.409 -5.489 1.00 0.00 O1 H

ATOM 3300 H16S POPCO 25 -3.695 -41.165 -5.704 1.00 0.00 O1 H

ATOM 3301 C217 POPCO 25 -4.898 -42.676 -6.963 1.00 0.00 O1 C

ATOM 3302 H17R POPCO 25 -4.084 -43.322 -7.319 1.00 0.00 O1 H

ATOM 3303 H17S POPCO 25 -5.777 -43.360 -6.780 1.00 0.00 O1 H

ATOM 3304 C218 POPCO 25 -5.270 -41.754 -8.159 1.00 0.00 O1 C

ATOM 3305 H18R POPCO 25 -6.128 -42.201 -8.772 1.00 0.00 O1 H

ATOM 3306 H18S POPCO 25 -5.710 -40.838 -7.739 1.00 0.00 O1 H

ATOM 3307 H18T POPCO 25 -4.440 -41.587 -8.897 1.00 0.00 O1 H

ATOM 3308 C33 POPCO 25 -8.969 -39.153 12.859 1.00 0.00 O1 C

ATOM 3309 H3X POPCO 25 -10.011 -39.653 12.668 1.00 0.00 O1 H

ATOM 3310 H3Y POPCO 25 -9.089 -38.042 12.977 1.00 0.00 O1 H

ATOM 3311 C34 POPCO 25 -7.968 -39.598 11.720 1.00 0.00 O1 C

ATOM 3312 H4X POPCO 25 -6.973 -39.280 12.035 1.00 0.00 O1 H

ATOM 3313 H4Y POPCO 25 -7.963 -40.676 11.752 1.00 0.00 O1 H

ATOM 3314 C35 POPCO 25 -8.318 -38.942 10.361 1.00 0.00 O1 C

ATOM 3315 H5X POPCO 25 -9.352 -39.273 10.182 1.00 0.00 O1 H

ATOM 3316 H5Y POPCO 25 -8.311 -37.856 10.606 1.00 0.00 O1 H

ATOM 3317 C36 POPCO 25 -7.517 -39.228 9.149 1.00 0.00 O1 C

ATOM 3318 H6X POPCO 25 -6.434 -39.248 9.387 1.00 0.00 O1 H

ATOM 3319 H6Y POPCO 25 -7.736 -40.221 8.691 1.00 0.00 O1 H

ATOM 3320 C37 POPCO 25 -7.828 -38.143 8.032 1.00 0.00 O1 C

ATOM 3321 H7X POPCO 25 -8.967 -38.156 7.809 1.00 0.00 O1 H

ATOM 3322 H7Y POPCO 25 -7.616 -37.100 8.384 1.00 0.00 O1 H

ATOM 3323 C38 POPCO 25 -7.122 -38.489 6.689 1.00 0.00 O1 C

ATOM 3324 H8X POPCO 25 -6.086 -38.746 6.985 1.00 0.00 O1 H

ATOM 3325 H8Y POPCO 25 -7.546 -39.417 6.450 1.00 0.00 O1 H

ATOM 3326 C39 POPCO 25 -7.337 -37.559 5.517 1.00 0.00 O1 C

ATOM 3327 H9X POPCO 25 -8.449 -37.289 5.618 1.00 0.00 O1 H

ATOM 3328 H9Y POPCO 25 -6.672 -36.639 5.573 1.00 0.00 O1 H

ATOM 3329 C310 POPCO 25 -7.145 -38.351 4.166 1.00 0.00 O1 C

ATOM 3330 H10X POPCO 25 -6.267 -39.065 4.300 1.00 0.00 O1 H

ATOM 3331 H10Y POPCO 25 -8.001 -39.083 4.026 1.00 0.00 O1 H

ATOM 3332 C311 POPCO 25 -7.054 -37.569 2.898 1.00 0.00 O1 C

ATOM 3333 H11X POPCO 25 -7.876 -36.792 2.802 1.00 0.00 O1 H

ATOM 3334 H11Y POPCO 25 -6.109 -37.032 2.880 1.00 0.00 O1 H

ATOM 3335 C312 POPCO 25 -7.013 -38.426 1.566 1.00 0.00 O1 C

ATOM 3336 H12X POPCO 25 -6.443 -39.405 1.743 1.00 0.00 O1 H

ATOM 3337 H12Y POPCO 25 -8.053 -38.751 1.338 1.00 0.00 O1 H

ATOM 3338 C313 POPCO 25 -6.542 -37.637 0.321 1.00 0.00 O1 C

ATOM 3339 H13X POPCO 25 -7.348 -36.918 0.154 1.00 0.00 O1 H

ATOM 3340 H13Y POPCO 25 -5.596 -37.038 0.614 1.00 0.00 O1 H

ATOM 3341 C314 POPCO 25 -6.095 -38.480 -0.837 1.00 0.00 O1 C

ATOM 3342 H14X POPCO 25 -5.023 -38.760 -0.765 1.00 0.00 O1 H

ATOM 3343 H14Y POPCO 25 -6.664 -39.429 -0.862 1.00 0.00 O1 H

ATOM 3344 C315 POPCO 25 -6.279 -37.697 -2.137 1.00 0.00 O1 C

ATOM 3345 H15X POPCO 25 -7.319 -37.338 -2.236 1.00 0.00 O1 H

ATOM 3346 H15Y POPCO 25 -5.607 -36.766 -2.062 1.00 0.00 O1 H

ATOM 3347 C316 POPCO 25 -5.855 -38.455 -3.365 1.00 0.00 O1 C

ATOM 3348 H16X POPCO 25 -6.330 -39.444 -3.500 1.00 0.00 O1 H

ATOM 3349 H16Y POPCO 25 -5.788 -37.846 -4.283 1.00 0.00 O1 H

ATOM 3350 H16Z POPCO 25 -4.814 -38.705 -3.212 1.00 0.00 O1 H

ATOM 3351 N POPCO 26 -1.772 -38.407 22.866 1.00 0.00 O1 N

ATOM 3352 C12 POPCO 26 -2.587 -39.337 21.917 1.00 0.00 O1 C

ATOM 3353 H12A POPCO 26 -3.105 -40.122 22.525 1.00 0.00 O1 H

ATOM 3354 H12B POPCO 26 -1.927 -39.734 21.196 1.00 0.00 O1 H

ATOM 3355 C13 POPCO 26 -2.496 -38.135 24.140 1.00 0.00 O1 C

ATOM 3356 H13A POPCO 26 -2.617 -39.059 24.632 1.00 0.00 O1 H

ATOM 3357 H13B POPCO 26 -3.455 -37.690 23.945 1.00 0.00 O1 H

ATOM 3358 H13C POPCO 26 -2.051 -37.393 24.664 1.00 0.00 O1 H

ATOM 3359 C14 POPCO 26 -0.473 -39.128 23.218 1.00 0.00 O1 C

ATOM 3360 H14A POPCO 26 0.101 -39.504 22.368 1.00 0.00 O1 H

ATOM 3361 H14B POPCO 26 -0.548 -39.945 23.986 1.00 0.00 O1 H

ATOM 3362 H14C POPCO 26 0.192 -38.508 23.773 1.00 0.00 O1 H

ATOM 3363 C15 POPCO 26 -1.429 -37.115 22.241 1.00 0.00 O1 C

ATOM 3364 H15A POPCO 26 -1.033 -37.321 21.205 1.00 0.00 O1 H

ATOM 3365 H15B POPCO 26 -2.346 -36.516 22.196 1.00 0.00 O1 H

ATOM 3366 H15C POPCO 26 -0.765 -36.527 22.741 1.00 0.00 O1 H

ATOM 3367 C11 POPCO 26 -3.671 -38.533 21.096 1.00 0.00 O1 C

ATOM 3368 H11A POPCO 26 -3.283 -37.597 20.569 1.00 0.00 O1 H

ATOM 3369 H11B POPCO 26 -3.922 -39.208 20.216 1.00 0.00 O1 H

ATOM 3370 P POPCO 26 -5.524 -36.916 22.217 1.00 0.00 O1 P

ATOM 3371 O13 POPCO 26 -4.440 -36.030 22.778 1.00 0.00 O1 O

ATOM 3372 O14 POPCO 26 -6.737 -37.072 23.056 1.00 0.00 O1 O

ATOM 3373 O12 POPCO 26 -4.860 -38.330 21.920 1.00 0.00 O1 O

ATOM 3374 O11 POPCO 26 -6.011 -36.271 20.844 1.00 0.00 O1 O

ATOM 3375 C1 POPCO 26 -5.664 -36.587 19.494 1.00 0.00 O1 C

ATOM 3376 HA POPCO 26 -4.630 -36.849 19.363 1.00 0.00 O1 H

ATOM 3377 HB POPCO 26 -6.378 -37.382 19.158 1.00 0.00 O1 H

ATOM 3378 C2 POPCO 26 -5.795 -35.416 18.457 1.00 0.00 O1 C

ATOM 3379 HS POPCO 26 -6.834 -34.979 18.517 1.00 0.00 O1 H

ATOM 3380 O21 POPCO 26 -5.547 -35.893 17.190 1.00 0.00 O1 O

ATOM 3381 C21 POPCO 26 -6.234 -35.532 16.139 1.00 0.00 O1 C

ATOM 3382 O22 POPCO 26 -6.950 -34.552 16.096 1.00 0.00 O1 O

ATOM 3383 C22 POPCO 26 -5.883 -36.387 14.937 1.00 0.00 O1 C

ATOM 3384 H2R POPCO 26 -4.793 -36.486 15.054 1.00 0.00 O1 H

ATOM 3385 H2S POPCO 26 -6.435 -37.398 14.861 1.00 0.00 O1 H

ATOM 3386 C3 POPCO 26 -4.929 -34.168 18.695 1.00 0.00 O1 C

ATOM 3387 HX POPCO 26 -5.271 -33.429 17.914 1.00 0.00 O1 H

ATOM 3388 HY POPCO 26 -5.182 -33.818 19.737 1.00 0.00 O1 H

ATOM 3389 O31 POPCO 26 -3.530 -34.466 18.631 1.00 0.00 O1 O

ATOM 3390 C31 POPCO 26 -2.741 -33.380 18.381 1.00 0.00 O1 C

ATOM 3391 O32 POPCO 26 -3.041 -32.237 18.561 1.00 0.00 O1 O

ATOM 3392 C32 POPCO 26 -1.360 -33.847 17.970 1.00 0.00 O1 C

ATOM 3393 H2X POPCO 26 -0.644 -33.750 18.839 1.00 0.00 O1 H

ATOM 3394 H2Y POPCO 26 -1.210 -34.875 17.600 1.00 0.00 O1 H

ATOM 3395 C23 POPCO 26 -6.101 -35.626 13.604 1.00 0.00 O1 C

ATOM 3396 H3R POPCO 26 -7.182 -35.797 13.232 1.00 0.00 O1 H

ATOM 3397 H3S POPCO 26 -5.988 -34.523 13.716 1.00 0.00 O1 H

ATOM 3398 C24 POPCO 26 -5.198 -36.253 12.595 1.00 0.00 O1 C

ATOM 3399 H4R POPCO 26 -4.185 -36.459 12.984 1.00 0.00 O1 H

ATOM 3400 H4S POPCO 26 -5.603 -37.187 12.368 1.00 0.00 O1 H

ATOM 3401 C25 POPCO 26 -5.103 -35.553 11.264 1.00 0.00 O1 C

ATOM 3402 H5R POPCO 26 -6.124 -35.606 10.711 1.00 0.00 O1 H

ATOM 3403 H5S POPCO 26 -4.689 -34.551 11.383 1.00 0.00 O1 H

ATOM 3404 C26 POPCO 26 -4.121 -36.355 10.336 1.00 0.00 O1 C

ATOM 3405 H6R POPCO 26 -3.058 -36.166 10.693 1.00 0.00 O1 H

ATOM 3406 H6S POPCO 26 -4.300 -37.425 10.399 1.00 0.00 O1 H

ATOM 3407 C27 POPCO 26 -4.276 -35.713 8.943 1.00 0.00 O1 C

ATOM 3408 H7R POPCO 26 -5.218 -35.827 8.491 1.00 0.00 O1 H

ATOM 3409 H7S POPCO 26 -4.246 -34.613 9.117 1.00 0.00 O1 H

ATOM 3410 C28 POPCO 26 -3.258 -36.224 7.882 1.00 0.00 O1 C

ATOM 3411 H8R POPCO 26 -2.356 -36.317 8.570 1.00 0.00 O1 H

ATOM 3412 H8S POPCO 26 -3.578 -37.247 7.558 1.00 0.00 O1 H

ATOM 3413 C29 POPCO 26 -3.029 -35.233 6.728 1.00 0.00 O1 C

ATOM 3414 H91 POPCO 26 -2.658 -34.248 7.113 1.00 0.00 O1 H

ATOM 3415 C210 POPCO 26 -3.169 -35.465 5.377 1.00 0.00 O1 C

ATOM 3416 H101 POPCO 26 -3.111 -34.603 4.642 1.00 0.00 O1 H

ATOM 3417 C211 POPCO 26 -3.531 -36.786 4.778 1.00 0.00 O1 C

ATOM 3418 H11R POPCO 26 -3.615 -37.660 5.503 1.00 0.00 O1 H

ATOM 3419 H11S POPCO 26 -4.478 -36.591 4.264 1.00 0.00 O1 H

ATOM 3420 C212 POPCO 26 -2.421 -37.100 3.789 1.00 0.00 O1 C

ATOM 3421 H12R POPCO 26 -1.394 -36.858 4.123 1.00 0.00 O1 H

ATOM 3422 H12S POPCO 26 -2.486 -38.176 3.631 1.00 0.00 O1 H

ATOM 3423 C213 POPCO 26 -2.674 -36.333 2.521 1.00 0.00 O1 C

ATOM 3424 H13R POPCO 26 -3.616 -36.727 2.255 1.00 0.00 O1 H

ATOM 3425 H13S POPCO 26 -2.725 -35.203 2.599 1.00 0.00 O1 H

ATOM 3426 C214 POPCO 26 -1.720 -36.694 1.425 1.00 0.00 O1 C

ATOM 3427 H14R POPCO 26 -0.730 -36.321 1.737 1.00 0.00 O1 H

ATOM 3428 H14S POPCO 26 -1.646 -37.805 1.212 1.00 0.00 O1 H

ATOM 3429 C215 POPCO 26 -2.045 -36.010 0.107 1.00 0.00 O1 C

ATOM 3430 H15R POPCO 26 -3.086 -36.460 -0.209 1.00 0.00 O1 H

ATOM 3431 H15S POPCO 26 -2.055 -34.899 0.384 1.00 0.00 O1 H

ATOM 3432 C216 POPCO 26 -1.095 -36.215 -1.077 1.00 0.00 O1 C

ATOM 3433 H16R POPCO 26 0.005 -35.945 -0.803 1.00 0.00 O1 H

ATOM 3434 H16S POPCO 26 -1.066 -37.249 -1.284 1.00 0.00 O1 H

ATOM 3435 C217 POPCO 26 -1.380 -35.446 -2.332 1.00 0.00 O1 C

ATOM 3436 H17R POPCO 26 -2.461 -35.548 -2.529 1.00 0.00 O1 H

ATOM 3437 H17S POPCO 26 -1.150 -34.371 -2.094 1.00 0.00 O1 H

ATOM 3438 C218 POPCO 26 -0.453 -35.834 -3.477 1.00 0.00 O1 C

ATOM 3439 H18R POPCO 26 -0.859 -35.518 -4.449 1.00 0.00 O1 H

ATOM 3440 H18S POPCO 26 0.531 -35.327 -3.356 1.00 0.00 O1 H

ATOM 3441 H18T POPCO 26 -0.270 -36.860 -3.496 1.00 0.00 O1 H

ATOM 3442 C33 POPCO 26 -0.825 -32.942 16.787 1.00 0.00 O1 C

ATOM 3443 H3X POPCO 26 -0.943 -31.865 17.136 1.00 0.00 O1 H

ATOM 3444 H3Y POPCO 26 0.183 -33.088 16.577 1.00 0.00 O1 H

ATOM 3445 C34 POPCO 26 -1.547 -33.300 15.486 1.00 0.00 O1 C

ATOM 3446 H4X POPCO 26 -1.571 -34.335 15.498 1.00 0.00 O1 H

ATOM 3447 H4Y POPCO 26 -2.628 -32.999 15.525 1.00 0.00 O1 H

ATOM 3448 C35 POPCO 26 -1.090 -32.757 14.110 1.00 0.00 O1 C

ATOM 3449 H5X POPCO 26 -1.313 -31.671 13.996 1.00 0.00 O1 H

ATOM 3450 H5Y POPCO 26 0.011 -32.860 14.160 1.00 0.00 O1 H

ATOM 3451 C36 POPCO 26 -1.381 -33.620 12.830 1.00 0.00 O1 C

ATOM 3452 H6X POPCO 26 -1.092 -34.698 13.027 1.00 0.00 O1 H

ATOM 3453 H6Y POPCO 26 -2.484 -33.650 12.603 1.00 0.00 O1 H

ATOM 3454 C37 POPCO 26 -0.502 -33.171 11.718 1.00 0.00 O1 C

ATOM 3455 H7X POPCO 26 -0.677 -32.042 11.492 1.00 0.00 O1 H

ATOM 3456 H7Y POPCO 26 0.551 -33.155 12.167 1.00 0.00 O1 H

ATOM 3457 C38 POPCO 26 -0.599 -34.035 10.474 1.00 0.00 O1 C

ATOM 3458 H8X POPCO 26 -0.437 -35.119 10.709 1.00 0.00 O1 H

ATOM 3459 H8Y POPCO 26 -1.641 -33.875 10.059 1.00 0.00 O1 H

ATOM 3460 C39 POPCO 26 0.429 -33.502 9.412 1.00 0.00 O1 C

ATOM 3461 H9X POPCO 26 0.253 -32.438 9.182 1.00 0.00 O1 H

ATOM 3462 H9Y POPCO 26 1.527 -33.509 9.879 1.00 0.00 O1 H

ATOM 3463 C310 POPCO 26 0.405 -34.261 8.095 1.00 0.00 O1 C

ATOM 3464 H10X POPCO 26 0.516 -35.312 8.364 1.00 0.00 O1 H

ATOM 3465 H10Y POPCO 26 -0.613 -33.957 7.688 1.00 0.00 O1 H

ATOM 3466 C311 POPCO 26 1.409 -33.765 7.100 1.00 0.00 O1 C

ATOM 3467 H11X POPCO 26 1.585 -32.618 7.255 1.00 0.00 O1 H

ATOM 3468 H11Y POPCO 26 2.462 -34.252 7.194 1.00 0.00 O1 H

ATOM 3469 C312 POPCO 26 0.937 -34.145 5.653 1.00 0.00 O1 C

ATOM 3470 H12X POPCO 26 0.421 -35.099 5.730 1.00 0.00 O1 H

ATOM 3471 H12Y POPCO 26 0.146 -33.405 5.401 1.00 0.00 O1 H

ATOM 3472 C313 POPCO 26 2.041 -34.363 4.557 1.00 0.00 O1 C

ATOM 3473 H13X POPCO 26 2.752 -33.518 4.589 1.00 0.00 O1 H

ATOM 3474 H13Y POPCO 26 2.442 -35.354 4.748 1.00 0.00 O1 H

ATOM 3475 C314 POPCO 26 1.499 -34.367 3.124 1.00 0.00 O1 C

ATOM 3476 H14X POPCO 26 0.869 -35.280 3.069 1.00 0.00 O1 H

ATOM 3477 H14Y POPCO 26 0.803 -33.522 3.070 1.00 0.00 O1 H

ATOM 3478 C315 POPCO 26 2.539 -34.229 1.995 1.00 0.00 O1 C

ATOM 3479 H15X POPCO 26 2.806 -33.138 2.134 1.00 0.00 O1 H

ATOM 3480 H15Y POPCO 26 3.466 -34.869 2.209 1.00 0.00 O1 H

ATOM 3481 C316 POPCO 26 2.052 -34.670 0.600 1.00 0.00 O1 C

ATOM 3482 H16X POPCO 26 1.264 -34.109 0.143 1.00 0.00 O1 H

ATOM 3483 H16Y POPCO 26 2.806 -34.646 -0.185 1.00 0.00 O1 H

ATOM 3484 H16Z POPCO 26 1.698 -35.706 0.631 1.00 0.00 O1 H

ATOM 3485 N POPCO 27 -8.146 -24.360 22.370 1.00 0.00 O1 N

ATOM 3486 C12 POPCO 27 -7.350 -25.520 22.911 1.00 0.00 O1 C

ATOM 3487 H12A POPCO 27 -7.712 -25.670 23.901 1.00 0.00 O1 H

ATOM 3488 H12B POPCO 27 -6.258 -25.316 22.840 1.00 0.00 O1 H

ATOM 3489 C13 POPCO 27 -9.565 -24.407 22.830 1.00 0.00 O1 C

ATOM 3490 H13A POPCO 27 -9.634 -24.106 23.861 1.00 0.00 O1 H

ATOM 3491 H13B POPCO 27 -9.811 -25.511 22.690 1.00 0.00 O1 H

ATOM 3492 H13C POPCO 27 -10.351 -23.888 22.164 1.00 0.00 O1 H

ATOM 3493 C14 POPCO 27 -7.453 -23.079 22.839 1.00 0.00 O1 C

ATOM 3494 H14A POPCO 27 -6.367 -23.173 22.552 1.00 0.00 O1 H

ATOM 3495 H14B POPCO 27 -7.500 -22.789 23.889 1.00 0.00 O1 H

ATOM 3496 H14C POPCO 27 -7.867 -22.345 22.141 1.00 0.00 O1 H

ATOM 3497 C15 POPCO 27 -8.025 -24.310 20.895 1.00 0.00 O1 C

ATOM 3498 H15A POPCO 27 -7.026 -24.299 20.571 1.00 0.00 O1 H

ATOM 3499 H15B POPCO 27 -8.543 -25.247 20.490 1.00 0.00 O1 H

ATOM 3500 H15C POPCO 27 -8.523 -23.509 20.424 1.00 0.00 O1 H

ATOM 3501 C11 POPCO 27 -7.503 -26.918 22.195 1.00 0.00 O1 C

ATOM 3502 H11A POPCO 27 -7.190 -26.770 21.135 1.00 0.00 O1 H

ATOM 3503 H11B POPCO 27 -6.655 -27.633 22.527 1.00 0.00 O1 H

ATOM 3504 P POPCO 27 -10.093 -27.532 21.477 1.00 0.00 O1 P

ATOM 3505 O13 POPCO 27 -11.188 -26.812 22.181 1.00 0.00 O1 O

ATOM 3506 O14 POPCO 27 -10.448 -28.939 21.076 1.00 0.00 O1 O

ATOM 3507 O12 POPCO 27 -8.762 -27.492 22.355 1.00 0.00 O1 O

ATOM 3508 O11 POPCO 27 -9.771 -26.651 20.151 1.00 0.00 O1 O

ATOM 3509 C1 POPCO 27 -10.084 -26.994 18.808 1.00 0.00 O1 C

ATOM 3510 HA POPCO 27 -10.087 -28.137 18.646 1.00 0.00 O1 H

ATOM 3511 HB POPCO 27 -11.096 -26.713 18.648 1.00 0.00 O1 H

ATOM 3512 C2 POPCO 27 -9.110 -26.259 17.868 1.00 0.00 O1 C

ATOM 3513 HS POPCO 27 -9.217 -25.167 18.242 1.00 0.00 O1 H

ATOM 3514 O21 POPCO 27 -9.564 -26.424 16.480 1.00 0.00 O1 O

ATOM 3515 C21 POPCO 27 -9.157 -25.424 15.701 1.00 0.00 O1 C

ATOM 3516 O22 POPCO 27 -8.504 -24.476 16.159 1.00 0.00 O1 O

ATOM 3517 C22 POPCO 27 -9.530 -25.658 14.220 1.00 0.00 O1 C

ATOM 3518 H2R POPCO 27 -9.409 -26.757 13.950 1.00 0.00 O1 H

ATOM 3519 H2S POPCO 27 -10.559 -25.342 14.024 1.00 0.00 O1 H

ATOM 3520 C3 POPCO 27 -7.633 -26.801 17.976 1.00 0.00 O1 C

ATOM 3521 HX POPCO 27 -7.008 -26.236 17.276 1.00 0.00 O1 H

ATOM 3522 HY POPCO 27 -7.370 -26.606 19.013 1.00 0.00 O1 H

ATOM 3523 O31 POPCO 27 -7.512 -28.232 17.760 1.00 0.00 O1 O

ATOM 3524 C31 POPCO 27 -6.304 -28.623 17.986 1.00 0.00 O1 C

ATOM 3525 O32 POPCO 27 -5.371 -27.968 18.420 1.00 0.00 O1 O

ATOM 3526 C32 POPCO 27 -6.263 -30.198 17.647 1.00 0.00 O1 C

ATOM 3527 H2X POPCO 27 -5.468 -30.679 18.273 1.00 0.00 O1 H

ATOM 3528 H2Y POPCO 27 -7.291 -30.583 17.700 1.00 0.00 O1 H

ATOM 3529 C23 POPCO 27 -8.540 -24.787 13.386 1.00 0.00 O1 C

ATOM 3530 H3R POPCO 27 -8.596 -23.741 13.720 1.00 0.00 O1 H

ATOM 3531 H3S POPCO 27 -7.505 -25.099 13.523 1.00 0.00 O1 H

ATOM 3532 C24 POPCO 27 -8.937 -24.954 11.885 1.00 0.00 O1 C

ATOM 3533 H4R POPCO 27 -9.074 -26.054 11.689 1.00 0.00 O1 H

ATOM 3534 H4S POPCO 27 -9.841 -24.553 11.649 1.00 0.00 O1 H

ATOM 3535 C25 POPCO 27 -7.959 -24.370 10.893 1.00 0.00 O1 C

ATOM 3536 H5R POPCO 27 -7.656 -23.307 11.273 1.00 0.00 O1 H

ATOM 3537 H5S POPCO 27 -7.121 -25.125 10.908 1.00 0.00 O1 H

ATOM 3538 C26 POPCO 27 -8.674 -24.382 9.538 1.00 0.00 O1 C

ATOM 3539 H6R POPCO 27 -8.886 -25.429 9.234 1.00 0.00 O1 H

ATOM 3540 H6S POPCO 27 -9.638 -23.938 9.571 1.00 0.00 O1 H

ATOM 3541 C27 POPCO 27 -7.801 -23.623 8.469 1.00 0.00 O1 C

ATOM 3542 H7R POPCO 27 -7.585 -22.558 8.845 1.00 0.00 O1 H

ATOM 3543 H7S POPCO 27 -6.893 -24.329 8.338 1.00 0.00 O1 H

ATOM 3544 C28 POPCO 27 -8.583 -23.509 7.139 1.00 0.00 O1 C

ATOM 3545 H8R POPCO 27 -8.844 -24.597 7.041 1.00 0.00 O1 H

ATOM 3546 H8S POPCO 27 -9.398 -22.852 7.284 1.00 0.00 O1 H

ATOM 3547 C29 POPCO 27 -7.772 -23.053 5.937 1.00 0.00 O1 C

ATOM 3548 H91 POPCO 27 -7.068 -22.155 6.068 1.00 0.00 O1 H

ATOM 3549 C210 POPCO 27 -7.785 -23.672 4.745 1.00 0.00 O1 C

ATOM 3550 H101 POPCO 27 -7.212 -23.191 3.978 1.00 0.00 O1 H

ATOM 3551 C211 POPCO 27 -8.623 -24.925 4.424 1.00 0.00 O1 C

ATOM 3552 H11R POPCO 27 -8.614 -25.651 5.292 1.00 0.00 O1 H

ATOM 3553 H11S POPCO 27 -9.683 -24.671 4.128 1.00 0.00 O1 H

ATOM 3554 C212 POPCO 27 -8.189 -25.791 3.214 1.00 0.00 O1 C

ATOM 3555 H12R POPCO 27 -7.169 -26.154 3.395 1.00 0.00 O1 H

ATOM 3556 H12S POPCO 27 -8.829 -26.705 3.328 1.00 0.00 O1 H

ATOM 3557 C213 POPCO 27 -8.466 -25.223 1.807 1.00 0.00 O1 C

ATOM 3558 H13R POPCO 27 -9.479 -24.795 1.852 1.00 0.00 O1 H

ATOM 3559 H13S POPCO 27 -7.770 -24.383 1.561 1.00 0.00 O1 H

ATOM 3560 C214 POPCO 27 -8.523 -26.316 0.737 1.00 0.00 O1 C

ATOM 3561 H14R POPCO 27 -7.507 -26.780 0.541 1.00 0.00 O1 H

ATOM 3562 H14S POPCO 27 -9.269 -27.051 1.083 1.00 0.00 O1 H

ATOM 3563 C215 POPCO 27 -8.996 -25.878 -0.620 1.00 0.00 O1 C

ATOM 3564 H15R POPCO 27 -9.957 -25.334 -0.523 1.00 0.00 O1 H

ATOM 3565 H15S POPCO 27 -8.268 -25.150 -1.123 1.00 0.00 O1 H

ATOM 3566 C216 POPCO 27 -9.165 -27.133 -1.558 1.00 0.00 O1 C

ATOM 3567 H16R POPCO 27 -8.209 -27.701 -1.577 1.00 0.00 O1 H

ATOM 3568 H16S POPCO 27 -9.870 -27.867 -1.001 1.00 0.00 O1 H

ATOM 3569 C217 POPCO 27 -9.689 -26.881 -2.985 1.00 0.00 O1 C

ATOM 3570 H17R POPCO 27 -10.726 -26.499 -2.988 1.00 0.00 O1 H

ATOM 3571 H17S POPCO 27 -8.954 -26.081 -3.268 1.00 0.00 O1 H

ATOM 3572 C218 POPCO 27 -9.652 -28.121 -3.927 1.00 0.00 O1 C

ATOM 3573 H18R POPCO 27 -10.330 -27.825 -4.742 1.00 0.00 O1 H

ATOM 3574 H18S POPCO 27 -8.635 -28.364 -4.247 1.00 0.00 O1 H

ATOM 3575 H18T POPCO 27 -10.186 -28.993 -3.341 1.00 0.00 O1 H

ATOM 3576 C33 POPCO 27 -5.828 -30.341 16.170 1.00 0.00 O1 C

ATOM 3577 H3X POPCO 27 -4.682 -30.226 16.139 1.00 0.00 O1 H

ATOM 3578 H3Y POPCO 27 -6.202 -31.365 15.707 1.00 0.00 O1 H

ATOM 3579 C34 POPCO 27 -6.522 -29.405 15.122 1.00 0.00 O1 C

ATOM 3580 H4X POPCO 27 -7.593 -29.441 15.283 1.00 0.00 O1 H

ATOM 3581 H4Y POPCO 27 -6.130 -28.350 15.083 1.00 0.00 O1 H

ATOM 3582 C35 POPCO 27 -6.360 -29.869 13.644 1.00 0.00 O1 C

ATOM 3583 H5X POPCO 27 -5.259 -29.940 13.358 1.00 0.00 O1 H

ATOM 3584 H5Y POPCO 27 -6.908 -30.854 13.629 1.00 0.00 O1 H

ATOM 3585 C36 POPCO 27 -7.153 -28.928 12.662 1.00 0.00 O1 C

ATOM 3586 H6X POPCO 27 -8.244 -28.855 12.874 1.00 0.00 O1 H

ATOM 3587 H6Y POPCO 27 -6.717 -27.860 12.665 1.00 0.00 O1 H

ATOM 3588 C37 POPCO 27 -7.072 -29.269 11.145 1.00 0.00 O1 C

ATOM 3589 H7X POPCO 27 -5.949 -29.335 10.954 1.00 0.00 O1 H

ATOM 3590 H7Y POPCO 27 -7.482 -30.235 11.055 1.00 0.00 O1 H

ATOM 3591 C38 POPCO 27 -7.615 -28.216 10.172 1.00 0.00 O1 C

ATOM 3592 H8X POPCO 27 -8.710 -28.192 10.484 1.00 0.00 O1 H

ATOM 3593 H8Y POPCO 27 -7.016 -27.249 10.243 1.00 0.00 O1 H

ATOM 3594 C39 POPCO 27 -7.387 -28.746 8.759 1.00 0.00 O1 C

ATOM 3595 H9X POPCO 27 -6.276 -28.628 8.637 1.00 0.00 O1 H

ATOM 3596 H9Y POPCO 27 -7.690 -29.799 8.657 1.00 0.00 O1 H

ATOM 3597 C310 POPCO 27 -8.191 -28.067 7.616 1.00 0.00 O1 C

ATOM 3598 H10X POPCO 27 -9.206 -27.973 8.082 1.00 0.00 O1 H

ATOM 3599 H10Y POPCO 27 -7.836 -27.014 7.617 1.00 0.00 O1 H

ATOM 3600 C311 POPCO 27 -8.136 -28.706 6.230 1.00 0.00 O1 C

ATOM 3601 H11X POPCO 27 -8.479 -29.761 6.421 1.00 0.00 O1 H

ATOM 3602 H11Y POPCO 27 -8.846 -28.207 5.598 1.00 0.00 O1 H

ATOM 3603 C312 POPCO 27 -6.803 -28.730 5.484 1.00 0.00 O1 C

ATOM 3604 H12X POPCO 27 -6.333 -27.693 5.498 1.00 0.00 O1 H

ATOM 3605 H12Y POPCO 27 -6.028 -29.308 6.021 1.00 0.00 O1 H

ATOM 3606 C313 POPCO 27 -7.026 -29.415 4.094 1.00 0.00 O1 C

ATOM 3607 H13X POPCO 27 -7.140 -30.488 4.345 1.00 0.00 O1 H

ATOM 3608 H13Y POPCO 27 -8.025 -29.107 3.785 1.00 0.00 O1 H

ATOM 3609 C314 POPCO 27 -5.937 -29.045 3.047 1.00 0.00 O1 C

ATOM 3610 H14X POPCO 27 -5.843 -27.959 3.257 1.00 0.00 O1 H

ATOM 3611 H14Y POPCO 27 -5.017 -29.425 3.416 1.00 0.00 O1 H

ATOM 3612 C315 POPCO 27 -6.260 -29.412 1.618 1.00 0.00 O1 C

ATOM 3613 H15X POPCO 27 -6.250 -30.503 1.441 1.00 0.00 O1 H

ATOM 3614 H15Y POPCO 27 -7.223 -29.003 1.509 1.00 0.00 O1 H

ATOM 3615 C316 POPCO 27 -5.504 -28.680 0.500 1.00 0.00 O1 C

ATOM 3616 H16X POPCO 27 -4.539 -29.138 0.362 1.00 0.00 O1 H

ATOM 3617 H16Y POPCO 27 -6.003 -28.812 -0.494 1.00 0.00 O1 H

ATOM 3618 H16Z POPCO 27 -5.533 -27.617 0.839 1.00 0.00 O1 H

ATOM 3619 N POPCO 28 -0.696 -18.682 22.782 1.00 0.00 O1 N

ATOM 3620 C12 POPCO 28 -0.525 -18.812 21.231 1.00 0.00 O1 C

ATOM 3621 H12A POPCO 28 0.479 -18.815 21.007 1.00 0.00 O1 H

ATOM 3622 H12B POPCO 28 -0.959 -17.929 20.778 1.00 0.00 O1 H

ATOM 3623 C13 POPCO 28 0.098 -19.718 23.522 1.00 0.00 O1 C

ATOM 3624 H13A POPCO 28 1.145 -19.559 23.356 1.00 0.00 O1 H

ATOM 3625 H13B POPCO 28 -0.057 -20.751 23.125 1.00 0.00 O1 H

ATOM 3626 H13C POPCO 28 -0.056 -19.739 24.574 1.00 0.00 O1 H

ATOM 3627 C14 POPCO 28 -0.173 -17.301 23.215 1.00 0.00 O1 C

ATOM 3628 H14A POPCO 28 -0.834 -16.464 23.009 1.00 0.00 O1 H

ATOM 3629 H14B POPCO 28 0.821 -17.160 22.758 1.00 0.00 O1 H

ATOM 3630 H14C POPCO 28 -0.096 -17.344 24.299 1.00 0.00 O1 H

ATOM 3631 C15 POPCO 28 -2.201 -18.781 22.948 1.00 0.00 O1 C

ATOM 3632 H15A POPCO 28 -2.786 -18.077 22.375 1.00 0.00 O1 H

ATOM 3633 H15B POPCO 28 -2.495 -19.816 22.648 1.00 0.00 O1 H

ATOM 3634 H15C POPCO 28 -2.480 -18.543 23.944 1.00 0.00 O1 H

ATOM 3635 C11 POPCO 28 -1.126 -19.967 20.411 1.00 0.00 O1 C

ATOM 3636 H11A POPCO 28 -2.248 -19.843 20.378 1.00 0.00 O1 H

ATOM 3637 H11B POPCO 28 -0.822 -19.773 19.340 1.00 0.00 O1 H

ATOM 3638 P POPCO 28 -1.519 -22.380 21.327 1.00 0.00 O1 P

ATOM 3639 O13 POPCO 28 -2.369 -21.773 22.436 1.00 0.00 O1 O

ATOM 3640 O14 POPCO 28 -0.677 -23.502 21.734 1.00 0.00 O1 O

ATOM 3641 O12 POPCO 28 -0.566 -21.184 20.867 1.00 0.00 O1 O

ATOM 3642 O11 POPCO 28 -2.376 -22.836 20.117 1.00 0.00 O1 O

ATOM 3643 C1 POPCO 28 -1.929 -22.743 18.706 1.00 0.00 O1 C

ATOM 3644 HA POPCO 28 -1.493 -21.819 18.465 1.00 0.00 O1 H

ATOM 3645 HB POPCO 28 -1.280 -23.624 18.516 1.00 0.00 O1 H

ATOM 3646 C2 POPCO 28 -3.201 -22.889 17.778 1.00 0.00 O1 C

ATOM 3647 HS POPCO 28 -3.700 -23.931 17.893 1.00 0.00 O1 H

ATOM 3648 O21 POPCO 28 -2.749 -22.646 16.417 1.00 0.00 O1 O

ATOM 3649 C21 POPCO 28 -3.729 -22.780 15.507 1.00 0.00 O1 C

ATOM 3650 O22 POPCO 28 -4.709 -23.491 15.532 1.00 0.00 O1 O

ATOM 3651 C22 POPCO 28 -3.530 -21.815 14.331 1.00 0.00 O1 C

ATOM 3652 H2R POPCO 28 -4.517 -21.646 13.842 1.00 0.00 O1 H

ATOM 3653 H2S POPCO 28 -3.085 -20.911 14.713 1.00 0.00 O1 H

ATOM 3654 C3 POPCO 28 -4.282 -21.846 18.172 1.00 0.00 O1 C

ATOM 3655 HX POPCO 28 -5.121 -21.966 17.417 1.00 0.00 O1 H

ATOM 3656 HY POPCO 28 -4.648 -22.195 19.120 1.00 0.00 O1 H

ATOM 3657 O31 POPCO 28 -3.683 -20.522 18.211 1.00 0.00 O1 O

ATOM 3658 C31 POPCO 28 -4.537 -19.519 18.252 1.00 0.00 O1 C

ATOM 3659 O32 POPCO 28 -5.769 -19.527 18.224 1.00 0.00 O1 O

ATOM 3660 C32 POPCO 28 -3.756 -18.224 18.214 1.00 0.00 O1 C

ATOM 3661 H2X POPCO 28 -3.935 -17.702 19.224 1.00 0.00 O1 H

ATOM 3662 H2Y POPCO 28 -2.671 -18.476 18.221 1.00 0.00 O1 H

ATOM 3663 C23 POPCO 28 -2.603 -22.367 13.279 1.00 0.00 O1 C

ATOM 3664 H3R POPCO 28 -1.666 -22.690 13.827 1.00 0.00 O1 H

ATOM 3665 H3S POPCO 28 -3.167 -23.248 12.993 1.00 0.00 O1 H

ATOM 3666 C24 POPCO 28 -2.426 -21.310 12.135 1.00 0.00 O1 C

ATOM 3667 H4R POPCO 28 -3.405 -20.766 12.051 1.00 0.00 O1 H

ATOM 3668 H4S POPCO 28 -1.709 -20.590 12.429 1.00 0.00 O1 H

ATOM 3669 C25 POPCO 28 -1.920 -21.981 10.815 1.00 0.00 O1 C

ATOM 3670 H5R POPCO 28 -1.023 -22.700 10.969 1.00 0.00 O1 H

ATOM 3671 H5S POPCO 28 -2.748 -22.442 10.219 1.00 0.00 O1 H

ATOM 3672 C26 POPCO 28 -1.308 -20.945 9.832 1.00 0.00 O1 C

ATOM 3673 H6R POPCO 28 -1.949 -20.064 9.719 1.00 0.00 O1 H

ATOM 3674 H6S POPCO 28 -0.468 -20.458 10.385 1.00 0.00 O1 H

ATOM 3675 C27 POPCO 28 -0.943 -21.310 8.389 1.00 0.00 O1 C

ATOM 3676 H7R POPCO 28 -0.182 -22.102 8.331 1.00 0.00 O1 H

ATOM 3677 H7S POPCO 28 -1.895 -21.842 8.013 1.00 0.00 O1 H

ATOM 3678 C28 POPCO 28 -0.513 -20.134 7.616 1.00 0.00 O1 C

ATOM 3679 H8R POPCO 28 -1.090 -19.186 7.892 1.00 0.00 O1 H

ATOM 3680 H8S POPCO 28 0.587 -19.912 7.737 1.00 0.00 O1 H

ATOM 3681 C29 POPCO 28 -1.010 -20.378 6.228 1.00 0.00 O1 C

ATOM 3682 H91 POPCO 28 -2.116 -20.522 6.154 1.00 0.00 O1 H

ATOM 3683 C210 POPCO 28 -0.289 -20.594 5.149 1.00 0.00 O1 C

ATOM 3684 H101 POPCO 28 -0.729 -20.611 4.168 1.00 0.00 O1 H

ATOM 3685 C211 POPCO 28 1.196 -20.490 4.881 1.00 0.00 O1 C

ATOM 3686 H11R POPCO 28 1.796 -20.477 5.848 1.00 0.00 O1 H

ATOM 3687 H11S POPCO 28 1.501 -21.493 4.484 1.00 0.00 O1 H

ATOM 3688 C212 POPCO 28 1.813 -19.345 4.007 1.00 0.00 O1 C

ATOM 3689 H12R POPCO 28 1.469 -18.346 4.437 1.00 0.00 O1 H

ATOM 3690 H12S POPCO 28 2.895 -19.399 3.957 1.00 0.00 O1 H

ATOM 3691 C213 POPCO 28 1.301 -19.491 2.591 1.00 0.00 O1 C

ATOM 3692 H13R POPCO 28 1.258 -20.571 2.329 1.00 0.00 O1 H

ATOM 3693 H13S POPCO 28 0.383 -18.985 2.572 1.00 0.00 O1 H

ATOM 3694 C214 POPCO 28 2.156 -18.890 1.455 1.00 0.00 O1 C

ATOM 3695 H14R POPCO 28 2.033 -17.814 1.635 1.00 0.00 O1 H

ATOM 3696 H14S POPCO 28 3.236 -19.144 1.526 1.00 0.00 O1 H

ATOM 3697 C215 POPCO 28 1.723 -19.190 -0.061 1.00 0.00 O1 C

ATOM 3698 H15R POPCO 28 2.245 -20.040 -0.366 1.00 0.00 O1 H

ATOM 3699 H15S POPCO 28 0.662 -19.459 -0.124 1.00 0.00 O1 H

ATOM 3700 C216 POPCO 28 1.773 -17.980 -0.956 1.00 0.00 O1 C

ATOM 3701 H16R POPCO 28 1.199 -17.090 -0.679 1.00 0.00 O1 H

ATOM 3702 H16S POPCO 28 2.845 -17.609 -1.121 1.00 0.00 O1 H

ATOM 3703 C217 POPCO 28 1.338 -18.297 -2.417 1.00 0.00 O1 C

ATOM 3704 H17R POPCO 28 2.277 -18.702 -2.918 1.00 0.00 O1 H

ATOM 3705 H17S POPCO 28 0.534 -19.033 -2.407 1.00 0.00 O1 H

ATOM 3706 C218 POPCO 28 0.974 -17.095 -3.232 1.00 0.00 O1 C

ATOM 3707 H18R POPCO 28 0.971 -17.288 -4.390 1.00 0.00 O1 H

ATOM 3708 H18S POPCO 28 -0.059 -16.822 -3.015 1.00 0.00 O1 H

ATOM 3709 H18T POPCO 28 1.686 -16.319 -2.849 1.00 0.00 O1 H

ATOM 3710 C33 POPCO 28 -4.199 -17.381 16.999 1.00 0.00 O1 C

ATOM 3711 H3X POPCO 28 -5.348 -17.419 16.874 1.00 0.00 O1 H

ATOM 3712 H3Y POPCO 28 -3.789 -16.343 17.272 1.00 0.00 O1 H

ATOM 3713 C34 POPCO 28 -3.614 -17.859 15.679 1.00 0.00 O1 C

ATOM 3714 H4X POPCO 28 -2.650 -18.381 15.790 1.00 0.00 O1 H

ATOM 3715 H4Y POPCO 28 -4.293 -18.641 15.382 1.00 0.00 O1 H

ATOM 3716 C35 POPCO 28 -3.707 -16.897 14.510 1.00 0.00 O1 C

ATOM 3717 H5X POPCO 28 -4.816 -16.610 14.419 1.00 0.00 O1 H

ATOM 3718 H5Y POPCO 28 -3.028 -16.035 14.648 1.00 0.00 O1 H

ATOM 3719 C36 POPCO 28 -3.320 -17.532 13.199 1.00 0.00 O1 C

ATOM 3720 H6X POPCO 28 -2.391 -18.126 13.366 1.00 0.00 O1 H

ATOM 3721 H6Y POPCO 28 -3.992 -18.232 12.662 1.00 0.00 O1 H

ATOM 3722 C37 POPCO 28 -2.950 -16.307 12.260 1.00 0.00 O1 C

ATOM 3723 H7X POPCO 28 -3.890 -15.682 12.136 1.00 0.00 O1 H

ATOM 3724 H7Y POPCO 28 -2.137 -15.587 12.733 1.00 0.00 O1 H

ATOM 3725 C38 POPCO 28 -2.394 -16.856 10.914 1.00 0.00 O1 C

ATOM 3726 H8X POPCO 28 -1.492 -17.464 11.082 1.00 0.00 O1 H

ATOM 3727 H8Y POPCO 28 -3.159 -17.376 10.349 1.00 0.00 O1 H

ATOM 3728 C39 POPCO 28 -1.979 -15.676 9.947 1.00 0.00 O1 C

ATOM 3729 H9X POPCO 28 -2.908 -15.224 9.625 1.00 0.00 O1 H

ATOM 3730 H9Y POPCO 28 -1.290 -15.037 10.515 1.00 0.00 O1 H

ATOM 3731 C310 POPCO 28 -1.308 -16.139 8.685 1.00 0.00 O1 C

ATOM 3732 H10X POPCO 28 -0.293 -16.640 8.898 1.00 0.00 O1 H

ATOM 3733 H10Y POPCO 28 -1.924 -16.920 8.189 1.00 0.00 O1 H

ATOM 3734 C311 POPCO 28 -1.161 -15.026 7.597 1.00 0.00 O1 C

ATOM 3735 H11X POPCO 28 -2.205 -14.733 7.450 1.00 0.00 O1 H

ATOM 3736 H11Y POPCO 28 -0.509 -14.167 7.971 1.00 0.00 O1 H

ATOM 3737 C312 POPCO 28 -0.525 -15.641 6.349 1.00 0.00 O1 C

ATOM 3738 H12X POPCO 28 0.383 -16.200 6.677 1.00 0.00 O1 H

ATOM 3739 H12Y POPCO 28 -1.163 -16.452 5.878 1.00 0.00 O1 H

ATOM 3740 C313 POPCO 28 -0.138 -14.539 5.364 1.00 0.00 O1 C

ATOM 3741 H13X POPCO 28 -1.021 -13.949 5.149 1.00 0.00 O1 H

ATOM 3742 H13Y POPCO 28 0.578 -13.801 5.797 1.00 0.00 O1 H

ATOM 3743 C314 POPCO 28 0.507 -15.104 4.046 1.00 0.00 O1 C

ATOM 3744 H14X POPCO 28 1.594 -14.972 4.103 1.00 0.00 O1 H

ATOM 3745 H14Y POPCO 28 0.370 -16.167 3.941 1.00 0.00 O1 H

ATOM 3746 C315 POPCO 28 0.193 -14.370 2.712 1.00 0.00 O1 C

ATOM 3747 H15X POPCO 28 -0.861 -14.327 2.663 1.00 0.00 O1 H

ATOM 3748 H15Y POPCO 28 0.573 -13.353 2.852 1.00 0.00 O1 H

ATOM 3749 C316 POPCO 28 0.947 -14.933 1.561 1.00 0.00 O1 C

ATOM 3750 H16X POPCO 28 0.527 -16.015 1.382 1.00 0.00 O1 H

ATOM 3751 H16Y POPCO 28 0.656 -14.383 0.618 1.00 0.00 O1 H

ATOM 3752 H16Z POPCO 28 2.058 -15.050 1.624 1.00 0.00 O1 H

ATOM 3753 N POPCO 29 -6.688 -10.501 21.789 1.00 0.00 O1 N

ATOM 3754 C12 POPCO 29 -5.502 -10.425 20.826 1.00 0.00 O1 C

ATOM 3755 H12A POPCO 29 -4.641 -10.095 21.394 1.00 0.00 O1 H

ATOM 3756 H12B POPCO 29 -5.628 -9.731 20.026 1.00 0.00 O1 H

ATOM 3757 C13 POPCO 29 -6.232 -11.183 22.969 1.00 0.00 O1 C

ATOM 3758 H13A POPCO 29 -5.490 -10.757 23.491 1.00 0.00 O1 H

ATOM 3759 H13B POPCO 29 -5.878 -12.180 22.618 1.00 0.00 O1 H

ATOM 3760 H13C POPCO 29 -7.059 -11.490 23.677 1.00 0.00 O1 H

ATOM 3761 C14 POPCO 29 -7.109 -9.128 22.156 1.00 0.00 O1 C

ATOM 3762 H14A POPCO 29 -7.463 -8.564 21.270 1.00 0.00 O1 H

ATOM 3763 H14B POPCO 29 -6.382 -8.631 22.830 1.00 0.00 O1 H

ATOM 3764 H14C POPCO 29 -7.924 -9.284 22.855 1.00 0.00 O1 H

ATOM 3765 C15 POPCO 29 -7.786 -11.358 21.135 1.00 0.00 O1 C

ATOM 3766 H15A POPCO 29 -8.252 -10.864 20.274 1.00 0.00 O1 H

ATOM 3767 H15B POPCO 29 -7.429 -12.212 20.657 1.00 0.00 O1 H

ATOM 3768 H15C POPCO 29 -8.481 -11.693 21.879 1.00 0.00 O1 H

ATOM 3769 C11 POPCO 29 -5.092 -11.724 20.027 1.00 0.00 O1 C

ATOM 3770 H11A POPCO 29 -5.845 -12.017 19.376 1.00 0.00 O1 H

ATOM 3771 H11B POPCO 29 -4.246 -11.483 19.336 1.00 0.00 O1 H

ATOM 3772 P POPCO 29 -5.435 -14.164 20.980 1.00 0.00 O1 P

ATOM 3773 O13 POPCO 29 -6.272 -14.187 22.211 1.00 0.00 O1 O

ATOM 3774 O14 POPCO 29 -4.547 -15.333 20.819 1.00 0.00 O1 O

ATOM 3775 O12 POPCO 29 -4.632 -12.794 20.842 1.00 0.00 O1 O

ATOM 3776 O11 POPCO 29 -6.482 -14.196 19.698 1.00 0.00 O1 O

ATOM 3777 C1 POPCO 29 -6.124 -14.457 18.349 1.00 0.00 O1 C

ATOM 3778 HA POPCO 29 -5.292 -13.754 18.019 1.00 0.00 O1 H

ATOM 3779 HB POPCO 29 -5.750 -15.506 18.380 1.00 0.00 O1 H

ATOM 3780 C2 POPCO 29 -7.266 -14.346 17.327 1.00 0.00 O1 C

ATOM 3781 HS POPCO 29 -7.989 -15.079 17.531 1.00 0.00 O1 H

ATOM 3782 O21 POPCO 29 -6.715 -14.585 16.043 1.00 0.00 O1 O

ATOM 3783 C21 POPCO 29 -7.470 -15.109 15.100 1.00 0.00 O1 C

ATOM 3784 O22 POPCO 29 -8.410 -15.878 15.262 1.00 0.00 O1 O

ATOM 3785 C22 POPCO 29 -7.042 -14.499 13.766 1.00 0.00 O1 C

ATOM 3786 H2R POPCO 29 -7.583 -13.514 13.569 1.00 0.00 O1 H

ATOM 3787 H2S POPCO 29 -5.948 -14.374 13.893 1.00 0.00 O1 H

ATOM 3788 C3 POPCO 29 -7.931 -12.938 17.375 1.00 0.00 O1 C

ATOM 3789 HX POPCO 29 -8.777 -13.034 16.587 1.00 0.00 O1 H

ATOM 3790 HY POPCO 29 -8.467 -12.808 18.379 1.00 0.00 O1 H

ATOM 3791 O31 POPCO 29 -7.041 -11.845 17.107 1.00 0.00 O1 O

ATOM 3792 C31 POPCO 29 -7.550 -10.692 17.460 1.00 0.00 O1 C

ATOM 3793 O32 POPCO 29 -8.539 -10.488 18.173 1.00 0.00 O1 O

ATOM 3794 C32 POPCO 29 -6.763 -9.528 16.792 1.00 0.00 O1 C

ATOM 3795 H2X POPCO 29 -6.900 -8.665 17.412 1.00 0.00 O1 H

ATOM 3796 H2Y POPCO 29 -5.717 -9.818 16.814 1.00 0.00 O1 H

ATOM 3797 C23 POPCO 29 -7.343 -15.279 12.456 1.00 0.00 O1 C

ATOM 3798 H3R POPCO 29 -6.740 -16.230 12.415 1.00 0.00 O1 H

ATOM 3799 H3S POPCO 29 -8.412 -15.558 12.503 1.00 0.00 O1 H

ATOM 3800 C24 POPCO 29 -7.097 -14.391 11.173 1.00 0.00 O1 C

ATOM 3801 H4R POPCO 29 -7.774 -13.493 11.223 1.00 0.00 O1 H

ATOM 3802 H4S POPCO 29 -6.034 -14.046 11.318 1.00 0.00 O1 H

ATOM 3803 C25 POPCO 29 -7.342 -15.010 9.777 1.00 0.00 O1 C

ATOM 3804 H5R POPCO 29 -6.498 -15.693 9.661 1.00 0.00 O1 H

ATOM 3805 H5S POPCO 29 -8.308 -15.616 9.798 1.00 0.00 O1 H

ATOM 3806 C26 POPCO 29 -7.329 -14.033 8.594 1.00 0.00 O1 C

ATOM 3807 H6R POPCO 29 -7.995 -13.198 8.965 1.00 0.00 O1 H

ATOM 3808 H6S POPCO 29 -6.349 -13.499 8.464 1.00 0.00 O1 H

ATOM 3809 C27 POPCO 29 -7.773 -14.571 7.284 1.00 0.00 O1 C

ATOM 3810 H7R POPCO 29 -7.097 -15.396 6.885 1.00 0.00 O1 H

ATOM 3811 H7S POPCO 29 -8.771 -15.071 7.361 1.00 0.00 O1 H

ATOM 3812 C28 POPCO 29 -7.868 -13.434 6.235 1.00 0.00 O1 C

ATOM 3813 H8R POPCO 29 -8.407 -12.609 6.697 1.00 0.00 O1 H

ATOM 3814 H8S POPCO 29 -6.790 -13.115 6.032 1.00 0.00 O1 H

ATOM 3815 C29 POPCO 29 -8.590 -13.731 4.977 1.00 0.00 O1 C

ATOM 3816 H91 POPCO 29 -9.618 -13.493 5.059 1.00 0.00 O1 H

ATOM 3817 C210 POPCO 29 -8.053 -14.354 3.885 1.00 0.00 O1 C

ATOM 3818 H101 POPCO 29 -8.728 -14.544 3.013 1.00 0.00 O1 H

ATOM 3819 C211 POPCO 29 -6.615 -14.859 3.849 1.00 0.00 O1 C

ATOM 3820 H11R POPCO 29 -6.090 -14.575 4.839 1.00 0.00 O1 H

ATOM 3821 H11S POPCO 29 -6.656 -15.889 3.514 1.00 0.00 O1 H

ATOM 3822 C212 POPCO 29 -5.819 -14.116 2.740 1.00 0.00 O1 C

ATOM 3823 H12R POPCO 29 -5.781 -13.024 3.065 1.00 0.00 O1 H

ATOM 3824 H12S POPCO 29 -4.847 -14.669 2.628 1.00 0.00 O1 H

ATOM 3825 C213 POPCO 29 -6.522 -14.295 1.373 1.00 0.00 O1 C

ATOM 3826 H13R POPCO 29 -6.434 -15.412 1.225 1.00 0.00 O1 H

ATOM 3827 H13S POPCO 29 -7.571 -13.958 1.415 1.00 0.00 O1 H

ATOM 3828 C214 POPCO 29 -5.757 -13.564 0.223 1.00 0.00 O1 C

ATOM 3829 H14R POPCO 29 -5.585 -12.452 0.409 1.00 0.00 O1 H

ATOM 3830 H14S POPCO 29 -4.760 -14.071 0.345 1.00 0.00 O1 H

ATOM 3831 C215 POPCO 29 -6.368 -13.713 -1.196 1.00 0.00 O1 C

ATOM 3832 H15R POPCO 29 -6.143 -14.762 -1.482 1.00 0.00 O1 H

ATOM 3833 H15S POPCO 29 -7.480 -13.669 -1.266 1.00 0.00 O1 H

ATOM 3834 C216 POPCO 29 -5.710 -12.731 -2.212 1.00 0.00 O1 C

ATOM 3835 H16R POPCO 29 -5.927 -11.730 -1.842 1.00 0.00 O1 H

ATOM 3836 H16S POPCO 29 -4.596 -12.852 -2.135 1.00 0.00 O1 H

ATOM 3837 C217 POPCO 29 -6.196 -12.862 -3.672 1.00 0.00 O1 C

ATOM 3838 H17R POPCO 29 -6.025 -13.918 -3.956 1.00 0.00 O1 H

ATOM 3839 H17S POPCO 29 -7.333 -12.755 -3.709 1.00 0.00 O1 H

ATOM 3840 C218 POPCO 29 -5.511 -11.937 -4.683 1.00 0.00 O1 C

ATOM 3841 H18R POPCO 29 -5.873 -12.066 -5.719 1.00 0.00 O1 H

ATOM 3842 H18S POPCO 29 -5.718 -10.906 -4.334 1.00 0.00 O1 H

ATOM 3843 H18T POPCO 29 -4.419 -12.085 -4.659 1.00 0.00 O1 H

ATOM 3844 C33 POPCO 29 -7.087 -9.235 15.296 1.00 0.00 O1 C

ATOM 3845 H3X POPCO 29 -8.209 -9.077 15.132 1.00 0.00 O1 H

ATOM 3846 H3Y POPCO 29 -6.461 -8.384 15.123 1.00 0.00 O1 H

ATOM 3847 C34 POPCO 29 -6.617 -10.332 14.350 1.00 0.00 O1 C

ATOM 3848 H4X POPCO 29 -5.825 -10.900 14.816 1.00 0.00 O1 H

ATOM 3849 H4Y POPCO 29 -7.319 -11.182 14.378 1.00 0.00 O1 H

ATOM 3850 C35 POPCO 29 -6.185 -9.898 12.934 1.00 0.00 O1 C

ATOM 3851 H5X POPCO 29 -7.095 -9.584 12.324 1.00 0.00 O1 H

ATOM 3852 H5Y POPCO 29 -5.432 -9.049 13.051 1.00 0.00 O1 H

ATOM 3853 C36 POPCO 29 -5.527 -11.136 12.189 1.00 0.00 O1 C

ATOM 3854 H6X POPCO 29 -4.672 -11.515 12.889 1.00 0.00 O1 H

ATOM 3855 H6Y POPCO 29 -6.258 -11.939 12.097 1.00 0.00 O1 H

ATOM 3856 C37 POPCO 29 -4.828 -10.647 10.943 1.00 0.00 O1 C

ATOM 3857 H7X POPCO 29 -5.518 -9.921 10.368 1.00 0.00 O1 H

ATOM 3858 H7Y POPCO 29 -4.022 -9.977 11.301 1.00 0.00 O1 H

ATOM 3859 C38 POPCO 29 -4.082 -11.695 10.042 1.00 0.00 O1 C

ATOM 3860 H8X POPCO 29 -3.314 -12.251 10.596 1.00 0.00 O1 H

ATOM 3861 H8Y POPCO 29 -4.771 -12.485 9.608 1.00 0.00 O1 H

ATOM 3862 C39 POPCO 29 -3.240 -10.980 8.996 1.00 0.00 O1 C

ATOM 3863 H9X POPCO 29 -3.720 -9.943 9.028 1.00 0.00 O1 H

ATOM 3864 H9Y POPCO 29 -2.207 -10.894 9.297 1.00 0.00 O1 H

ATOM 3865 C310 POPCO 29 -3.426 -11.529 7.612 1.00 0.00 O1 C

ATOM 3866 H10X POPCO 29 -3.446 -12.630 7.631 1.00 0.00 O1 H

ATOM 3867 H10Y POPCO 29 -4.522 -11.406 7.243 1.00 0.00 O1 H

ATOM 3868 C311 POPCO 29 -2.458 -11.115 6.589 1.00 0.00 O1 C

ATOM 3869 H11X POPCO 29 -2.390 -10.046 6.528 1.00 0.00 O1 H

ATOM 3870 H11Y POPCO 29 -1.436 -11.458 6.805 1.00 0.00 O1 H

ATOM 3871 C312 POPCO 29 -2.922 -11.658 5.208 1.00 0.00 O1 C

ATOM 3872 H12X POPCO 29 -3.085 -12.738 5.351 1.00 0.00 O1 H

ATOM 3873 H12Y POPCO 29 -3.955 -11.239 5.026 1.00 0.00 O1 H

ATOM 3874 C313 POPCO 29 -1.975 -11.319 4.036 1.00 0.00 O1 C

ATOM 3875 H13X POPCO 29 -1.711 -10.272 4.003 1.00 0.00 O1 H

ATOM 3876 H13Y POPCO 29 -0.990 -11.699 4.097 1.00 0.00 O1 H

ATOM 3877 C314 POPCO 29 -2.574 -11.684 2.630 1.00 0.00 O1 C

ATOM 3878 H14X POPCO 29 -2.832 -12.802 2.638 1.00 0.00 O1 H

ATOM 3879 H14Y POPCO 29 -3.507 -10.994 2.471 1.00 0.00 O1 H

ATOM 3880 C315 POPCO 29 -1.581 -11.233 1.610 1.00 0.00 O1 C

ATOM 3881 H15X POPCO 29 -1.682 -10.113 1.507 1.00 0.00 O1 H

ATOM 3882 H15Y POPCO 29 -0.597 -11.443 1.998 1.00 0.00 O1 H

ATOM 3883 C316 POPCO 29 -1.819 -11.901 0.264 1.00 0.00 O1 C

ATOM 3884 H16X POPCO 29 -2.902 -11.676 0.029 1.00 0.00 O1 H

ATOM 3885 H16Y POPCO 29 -1.182 -11.742 -0.605 1.00 0.00 O1 H

ATOM 3886 H16Z POPCO 29 -1.848 -13.045 0.427 1.00 0.00 O1 H

ATOM 3887 N POPCO 30 -3.281 0.388 20.116 1.00 0.00 O1 N

ATOM 3888 C12 POPCO 30 -2.509 0.596 18.844 1.00 0.00 O1 C

ATOM 3889 H12A POPCO 30 -1.548 1.139 19.018 1.00 0.00 O1 H

ATOM 3890 H12B POPCO 30 -3.034 1.357 18.260 1.00 0.00 O1 H

ATOM 3891 C13 POPCO 30 -2.389 -0.292 21.083 1.00 0.00 O1 C

ATOM 3892 H13A POPCO 30 -1.376 0.074 21.023 1.00 0.00 O1 H

ATOM 3893 H13B POPCO 30 -2.387 -1.317 20.799 1.00 0.00 O1 H

ATOM 3894 H13C POPCO 30 -2.716 -0.285 22.082 1.00 0.00 O1 H

ATOM 3895 C14 POPCO 30 -3.571 1.724 20.610 1.00 0.00 O1 C

ATOM 3896 H14A POPCO 30 -4.099 2.312 19.770 1.00 0.00 O1 H

ATOM 3897 H14B POPCO 30 -2.704 2.377 20.808 1.00 0.00 O1 H

ATOM 3898 H14C POPCO 30 -4.226 1.716 21.449 1.00 0.00 O1 H

ATOM 3899 C15 POPCO 30 -4.502 -0.345 19.772 1.00 0.00 O1 C

ATOM 3900 H15A POPCO 30 -5.112 0.200 19.082 1.00 0.00 O1 H

ATOM 3901 H15B POPCO 30 -4.327 -1.291 19.358 1.00 0.00 O1 H

ATOM 3902 H15C POPCO 30 -5.114 -0.495 20.702 1.00 0.00 O1 H

ATOM 3903 C11 POPCO 30 -2.233 -0.654 17.943 1.00 0.00 O1 C

ATOM 3904 H11A POPCO 30 -3.196 -1.200 17.739 1.00 0.00 O1 H

ATOM 3905 H11B POPCO 30 -1.787 -0.256 17.005 1.00 0.00 O1 H

ATOM 3906 P POPCO 30 -1.217 -2.914 19.016 1.00 0.00 O1 P

ATOM 3907 O13 POPCO 30 -2.560 -3.001 19.601 1.00 0.00 O1 O

ATOM 3908 O14 POPCO 30 -0.072 -3.147 19.923 1.00 0.00 O1 O

ATOM 3909 O12 POPCO 30 -1.145 -1.396 18.381 1.00 0.00 O1 O

ATOM 3910 O11 POPCO 30 -1.233 -3.853 17.785 1.00 0.00 O1 O

ATOM 3911 C1 POPCO 30 -1.479 -3.432 16.475 1.00 0.00 O1 C

ATOM 3912 HA POPCO 30 -2.537 -2.945 16.361 1.00 0.00 O1 H

ATOM 3913 HB POPCO 30 -0.618 -2.810 16.234 1.00 0.00 O1 H

ATOM 3914 C2 POPCO 30 -1.568 -4.632 15.533 1.00 0.00 O1 C

ATOM 3915 HS POPCO 30 -0.642 -5.182 15.682 1.00 0.00 O1 H

ATOM 3916 O21 POPCO 30 -1.831 -4.054 14.220 1.00 0.00 O1 O

ATOM 3917 C21 POPCO 30 -1.043 -4.433 13.210 1.00 0.00 O1 C

ATOM 3918 O22 POPCO 30 -0.355 -5.476 13.159 1.00 0.00 O1 O

ATOM 3919 C22 POPCO 30 -1.360 -3.572 11.983 1.00 0.00 O1 C

ATOM 3920 H2R POPCO 30 -2.469 -3.630 11.842 1.00 0.00 O1 H

ATOM 3921 H2S POPCO 30 -1.237 -2.491 12.226 1.00 0.00 O1 H

ATOM 3922 C3 POPCO 30 -2.702 -5.644 15.815 1.00 0.00 O1 C

ATOM 3923 HX POPCO 30 -2.649 -6.454 15.120 1.00 0.00 O1 H

ATOM 3924 HY POPCO 30 -2.641 -6.012 16.905 1.00 0.00 O1 H

ATOM 3925 O31 POPCO 30 -4.019 -5.138 15.576 1.00 0.00 O1 O

ATOM 3926 C31 POPCO 30 -5.021 -5.855 15.957 1.00 0.00 O1 C

ATOM 3927 O32 POPCO 30 -4.983 -7.004 16.399 1.00 0.00 O1 O

ATOM 3928 C32 POPCO 30 -6.352 -5.096 15.687 1.00 0.00 O1 C

ATOM 3929 H2X POPCO 30 -7.015 -5.350 16.493 1.00 0.00 O1 H

ATOM 3930 H2Y POPCO 30 -6.260 -4.013 15.704 1.00 0.00 O1 H

ATOM 3931 C23 POPCO 30 -0.589 -3.929 10.690 1.00 0.00 O1 C

ATOM 3932 H3R POPCO 30 0.457 -3.824 10.907 1.00 0.00 O1 H

ATOM 3933 H3S POPCO 30 -0.730 -5.020 10.369 1.00 0.00 O1 H

ATOM 3934 C24 POPCO 30 -1.005 -3.102 9.478 1.00 0.00 O1 C

ATOM 3935 H4R POPCO 30 -2.111 -3.330 9.352 1.00 0.00 O1 H

ATOM 3936 H4S POPCO 30 -0.948 -1.977 9.806 1.00 0.00 O1 H

ATOM 3937 C25 POPCO 30 -0.178 -3.516 8.225 1.00 0.00 O1 C

ATOM 3938 H5R POPCO 30 0.840 -2.974 8.281 1.00 0.00 O1 H

ATOM 3939 H5S POPCO 30 -0.049 -4.564 8.181 1.00 0.00 O1 H

ATOM 3940 C26 POPCO 30 -1.018 -3.121 6.982 1.00 0.00 O1 C

ATOM 3941 H6R POPCO 30 -1.911 -3.772 6.831 1.00 0.00 O1 H

ATOM 3942 H6S POPCO 30 -1.231 -2.042 7.105 1.00 0.00 O1 H

ATOM 3943 C27 POPCO 30 -0.206 -3.245 5.725 1.00 0.00 O1 C

ATOM 3944 H7R POPCO 30 0.622 -2.522 5.726 1.00 0.00 O1 H

ATOM 3945 H7S POPCO 30 0.159 -4.311 5.712 1.00 0.00 O1 H

ATOM 3946 C28 POPCO 30 -1.085 -3.040 4.378 1.00 0.00 O1 C

ATOM 3947 H8R POPCO 30 -1.931 -3.754 4.436 1.00 0.00 O1 H

ATOM 3948 H8S POPCO 30 -1.335 -1.974 4.579 1.00 0.00 O1 H

ATOM 3949 C29 POPCO 30 -0.306 -3.219 3.151 1.00 0.00 O1 C

ATOM 3950 H91 POPCO 30 0.124 -4.266 3.043 1.00 0.00 O1 H

ATOM 3951 C210 POPCO 30 -0.131 -2.359 2.167 1.00 0.00 O1 C

ATOM 3952 H101 POPCO 30 0.412 -2.673 1.197 1.00 0.00 O1 H

ATOM 3953 C211 POPCO 30 -0.749 -0.979 2.110 1.00 0.00 O1 C

ATOM 3954 H11R POPCO 30 -1.290 -0.674 3.020 1.00 0.00 O1 H

ATOM 3955 H11S POPCO 30 -0.021 -0.104 1.956 1.00 0.00 O1 H

ATOM 3956 C212 POPCO 30 -1.763 -0.898 0.905 1.00 0.00 O1 C

ATOM 3957 H12R POPCO 30 -2.446 -1.775 1.042 1.00 0.00 O1 H

ATOM 3958 H12S POPCO 30 -2.210 0.173 0.878 1.00 0.00 O1 H

ATOM 3959 C213 POPCO 30 -1.082 -1.028 -0.546 1.00 0.00 O1 C

ATOM 3960 H13R POPCO 30 -0.348 -0.168 -0.559 1.00 0.00 O1 H

ATOM 3961 H13S POPCO 30 -0.552 -1.950 -0.582 1.00 0.00 O1 H

ATOM 3962 C214 POPCO 30 -2.080 -0.789 -1.747 1.00 0.00 O1 C

ATOM 3963 H14R POPCO 30 -2.868 -1.595 -1.741 1.00 0.00 O1 H

ATOM 3964 H14S POPCO 30 -2.556 0.182 -1.545 1.00 0.00 O1 H

ATOM 3965 C215 POPCO 30 -1.310 -0.990 -3.057 1.00 0.00 O1 C

ATOM 3966 H15R POPCO 30 -0.758 -0.055 -3.191 1.00 0.00 O1 H

ATOM 3967 H15S POPCO 30 -0.623 -1.849 -3.190 1.00 0.00 O1 H

ATOM 3968 C216 POPCO 30 -2.193 -1.052 -4.343 1.00 0.00 O1 C

ATOM 3969 H16R POPCO 30 -2.984 -1.858 -4.135 1.00 0.00 O1 H

ATOM 3970 H16S POPCO 30 -2.752 -0.134 -4.560 1.00 0.00 O1 H

ATOM 3971 C217 POPCO 30 -1.334 -1.439 -5.579 1.00 0.00 O1 C

ATOM 3972 H17R POPCO 30 -0.492 -0.766 -5.678 1.00 0.00 O1 H

ATOM 3973 H17S POPCO 30 -0.950 -2.442 -5.576 1.00 0.00 O1 H

ATOM 3974 C218 POPCO 30 -2.116 -1.350 -6.881 1.00 0.00 O1 C

ATOM 3975 H18R POPCO 30 -1.467 -1.417 -7.769 1.00 0.00 O1 H

ATOM 3976 H18S POPCO 30 -2.920 -2.152 -7.018 1.00 0.00 O1 H

ATOM 3977 H18T POPCO 30 -2.705 -0.394 -6.961 1.00 0.00 O1 H

ATOM 3978 C33 POPCO 30 -6.974 -5.615 14.390 1.00 0.00 O1 C

ATOM 3979 H3X POPCO 30 -7.409 -6.663 14.511 1.00 0.00 O1 H

ATOM 3980 H3Y POPCO 30 -7.811 -4.857 14.280 1.00 0.00 O1 H

ATOM 3981 C34 POPCO 30 -6.230 -5.387 13.055 1.00 0.00 O1 C

ATOM 3982 H4X POPCO 30 -5.827 -4.278 13.086 1.00 0.00 O1 H

ATOM 3983 H4Y POPCO 30 -5.264 -5.980 13.090 1.00 0.00 O1 H

ATOM 3984 C35 POPCO 30 -6.989 -5.558 11.797 1.00 0.00 O1 C

ATOM 3985 H5X POPCO 30 -7.241 -6.599 11.526 1.00 0.00 O1 H

ATOM 3986 H5Y POPCO 30 -7.978 -5.083 12.077 1.00 0.00 O1 H

ATOM 3987 C36 POPCO 30 -6.365 -4.871 10.637 1.00 0.00 O1 C

ATOM 3988 H6X POPCO 30 -6.031 -3.842 10.918 1.00 0.00 O1 H

ATOM 3989 H6Y POPCO 30 -5.474 -5.498 10.343 1.00 0.00 O1 H

ATOM 3990 C37 POPCO 30 -7.195 -4.899 9.304 1.00 0.00 O1 C

ATOM 3991 H7X POPCO 30 -7.321 -5.995 9.150 1.00 0.00 O1 H

ATOM 3992 H7Y POPCO 30 -8.160 -4.435 9.521 1.00 0.00 O1 H

ATOM 3993 C38 POPCO 30 -6.363 -4.330 8.137 1.00 0.00 O1 C

ATOM 3994 H8X POPCO 30 -6.229 -3.272 8.213 1.00 0.00 O1 H

ATOM 3995 H8Y POPCO 30 -5.335 -4.779 8.185 1.00 0.00 O1 H

ATOM 3996 C39 POPCO 30 -6.938 -4.516 6.657 1.00 0.00 O1 C

ATOM 3997 H9X POPCO 30 -6.932 -5.623 6.494 1.00 0.00 O1 H

ATOM 3998 H9Y POPCO 30 -8.032 -4.352 6.529 1.00 0.00 O1 H

ATOM 3999 C310 POPCO 30 -6.131 -3.799 5.567 1.00 0.00 O1 C

ATOM 4000 H10X POPCO 30 -6.410 -2.743 5.608 1.00 0.00 O1 H

ATOM 4001 H10Y POPCO 30 -5.038 -3.913 5.725 1.00 0.00 O1 H

ATOM 4002 C311 POPCO 30 -6.486 -4.493 4.267 1.00 0.00 O1 C

ATOM 4003 H11X POPCO 30 -6.480 -5.592 4.195 1.00 0.00 O1 H

ATOM 4004 H11Y POPCO 30 -7.639 -4.386 4.292 1.00 0.00 O1 H

ATOM 4005 C312 POPCO 30 -5.875 -3.814 3.069 1.00 0.00 O1 C

ATOM 4006 H12X POPCO 30 -5.897 -2.755 3.081 1.00 0.00 O1 H

ATOM 4007 H12Y POPCO 30 -4.805 -4.049 3.073 1.00 0.00 O1 H

ATOM 4008 C313 POPCO 30 -6.355 -4.232 1.631 1.00 0.00 O1 C

ATOM 4009 H13X POPCO 30 -6.019 -5.231 1.264 1.00 0.00 O1 H

ATOM 4010 H13Y POPCO 30 -7.433 -4.232 1.653 1.00 0.00 O1 H

ATOM 4011 C314 POPCO 30 -6.078 -2.972 0.716 1.00 0.00 O1 C

ATOM 4012 H14X POPCO 30 -6.585 -2.034 1.046 1.00 0.00 O1 H

ATOM 4013 H14Y POPCO 30 -4.982 -2.824 0.841 1.00 0.00 O1 H

ATOM 4014 C315 POPCO 30 -6.300 -3.183 -0.807 1.00 0.00 O1 C

ATOM 4015 H15X POPCO 30 -5.636 -4.056 -1.097 1.00 0.00 O1 H

ATOM 4016 H15Y POPCO 30 -7.394 -3.460 -0.911 1.00 0.00 O1 H

ATOM 4017 C316 POPCO 30 -5.996 -2.015 -1.721 1.00 0.00 O1 C

ATOM 4018 H16X POPCO 30 -4.841 -1.857 -1.676 1.00 0.00 O1 H

ATOM 4019 H16Y POPCO 30 -6.163 -2.142 -2.815 1.00 0.00 O1 H

ATOM 4020 H16Z POPCO 30 -6.596 -1.071 -1.355 1.00 0.00 O1 H

ATOM 4021 N POPCO 1 -44.852 -39.256 -18.256 1.00 0.00 O2 N

ATOM 4022 C12 POPCO 1 -45.907 -40.392 -18.002 1.00 0.00 O2 C

ATOM 4023 H12A POPCO 1 -46.214 -40.714 -19.000 1.00 0.00 O2 H

ATOM 4024 H12B POPCO 1 -46.793 -40.088 -17.600 1.00 0.00 O2 H

ATOM 4025 C13 POPCO 1 -43.803 -39.704 -19.291 1.00 0.00 O2 C

ATOM 4026 H13A POPCO 1 -44.228 -40.040 -20.110 1.00 0.00 O2 H

ATOM 4027 H13B POPCO 1 -43.205 -40.461 -18.816 1.00 0.00 O2 H

ATOM 4028 H13C POPCO 1 -43.129 -38.890 -19.515 1.00 0.00 O2 H

ATOM 4029 C14 POPCO 1 -45.554 -38.013 -18.697 1.00 0.00 O2 C

ATOM 4030 H14A POPCO 1 -46.200 -37.541 -17.876 1.00 0.00 O2 H

ATOM 4031 H14B POPCO 1 -46.247 -38.383 -19.420 1.00 0.00 O2 H

ATOM 4032 H14C POPCO 1 -44.903 -37.306 -19.185 1.00 0.00 O2 H

ATOM 4033 C15 POPCO 1 -44.203 -38.930 -16.935 1.00 0.00 O2 C

ATOM 4034 H15A POPCO 1 -44.969 -38.802 -16.186 1.00 0.00 O2 H

ATOM 4035 H15B POPCO 1 -43.630 -39.746 -16.524 1.00 0.00 O2 H

ATOM 4036 H15C POPCO 1 -43.590 -38.029 -16.924 1.00 0.00 O2 H

ATOM 4037 C11 POPCO 1 -45.350 -41.681 -17.321 1.00 0.00 O2 C

ATOM 4038 H11A POPCO 1 -45.122 -41.598 -16.218 1.00 0.00 O2 H

ATOM 4039 H11B POPCO 1 -46.135 -42.478 -17.342 1.00 0.00 O2 H

ATOM 4040 P POPCO 1 -42.899 -42.504 -17.324 1.00 0.00 O2 P

ATOM 4041 O13 POPCO 1 -41.824 -42.181 -18.237 1.00 0.00 O2 O

ATOM 4042 O14 POPCO 1 -43.030 -43.875 -16.750 1.00 0.00 O2 O

ATOM 4043 O12 POPCO 1 -44.340 -42.286 -18.042 1.00 0.00 O2 O

ATOM 4044 O11 POPCO 1 -42.757 -41.476 -16.166 1.00 0.00 O2 O

ATOM 4045 C1 POPCO 1 -42.582 -42.060 -14.858 1.00 0.00 O2 C

ATOM 4046 HA POPCO 1 -43.365 -42.765 -14.646 1.00 0.00 O2 H

ATOM 4047 HB POPCO 1 -41.607 -42.621 -14.798 1.00 0.00 O2 H

ATOM 4048 C2 POPCO 1 -42.485 -40.991 -13.751 1.00 0.00 O2 C

ATOM 4049 HS POPCO 1 -41.589 -40.385 -14.066 1.00 0.00 O2 H

ATOM 4050 O21 POPCO 1 -42.251 -41.700 -12.539 1.00 0.00 O2 O

ATOM 4051 C21 POPCO 1 -42.036 -40.943 -11.462 1.00 0.00 O2 C

ATOM 4052 O22 POPCO 1 -41.764 -39.781 -11.533 1.00 0.00 O2 O

ATOM 4053 C22 POPCO 1 -42.240 -41.712 -10.106 1.00 0.00 O2 C

ATOM 4054 H2R POPCO 1 -43.344 -41.748 -9.958 1.00 0.00 O2 H

ATOM 4055 H2S POPCO 1 -41.903 -42.776 -10.335 1.00 0.00 O2 H

ATOM 4056 C3 POPCO 1 -43.731 -40.010 -13.566 1.00 0.00 O2 C

ATOM 4057 HX POPCO 1 -43.549 -39.234 -12.819 1.00 0.00 O2 H

ATOM 4058 HY POPCO 1 -43.815 -39.482 -14.578 1.00 0.00 O2 H

ATOM 4059 O31 POPCO 1 -45.034 -40.650 -13.306 1.00 0.00 O2 O

ATOM 4060 C31 POPCO 1 -46.071 -40.102 -13.835 1.00 0.00 O2 C

ATOM 4061 O32 POPCO 1 -46.129 -39.116 -14.539 1.00 0.00 O2 O

ATOM 4062 C32 POPCO 1 -47.302 -40.961 -13.640 1.00 0.00 O2 C

ATOM 4063 H2X POPCO 1 -47.846 -40.889 -14.575 1.00 0.00 O2 H

ATOM 4064 H2Y POPCO 1 -47.046 -42.047 -13.445 1.00 0.00 O2 H

ATOM 4065 C23 POPCO 1 -41.784 -40.935 -8.847 1.00 0.00 O2 C

ATOM 4066 H3R POPCO 1 -40.741 -40.806 -9.062 1.00 0.00 O2 H

ATOM 4067 H3S POPCO 1 -42.337 -39.972 -8.942 1.00 0.00 O2 H

ATOM 4068 C24 POPCO 1 -41.940 -41.641 -7.470 1.00 0.00 O2 C

ATOM 4069 H4R POPCO 1 -43.006 -41.889 -7.375 1.00 0.00 O2 H

ATOM 4070 H4S POPCO 1 -41.247 -42.472 -7.274 1.00 0.00 O2 H

ATOM 4071 C25 POPCO 1 -41.602 -40.554 -6.347 1.00 0.00 O2 C

ATOM 4072 H5R POPCO 1 -40.471 -40.338 -6.516 1.00 0.00 O2 H

ATOM 4073 H5S POPCO 1 -42.179 -39.646 -6.614 1.00 0.00 O2 H

ATOM 4074 C26 POPCO 1 -41.923 -41.054 -4.956 1.00 0.00 O2 C

ATOM 4075 H6R POPCO 1 -43.030 -41.148 -4.745 1.00 0.00 O2 H

ATOM 4076 H6S POPCO 1 -41.522 -42.110 -4.976 1.00 0.00 O2 H

ATOM 4077 C27 POPCO 1 -41.208 -40.297 -3.779 1.00 0.00 O2 C

ATOM 4078 H7R POPCO 1 -40.113 -40.485 -3.972 1.00 0.00 O2 H

ATOM 4079 H7S POPCO 1 -41.562 -39.249 -3.888 1.00 0.00 O2 H

ATOM 4080 C28 POPCO 1 -41.536 -41.009 -2.434 1.00 0.00 O2 C

ATOM 4081 H8R POPCO 1 -42.633 -41.214 -2.359 1.00 0.00 O2 H

ATOM 4082 H8S POPCO 1 -41.096 -42.095 -2.492 1.00 0.00 O2 H

ATOM 4083 C29 POPCO 1 -40.987 -40.442 -1.147 1.00 0.00 O2 C

ATOM 4084 H91 POPCO 1 -39.920 -40.563 -0.957 1.00 0.00 O2 H

ATOM 4085 C210 POPCO 1 -41.588 -39.743 -0.200 1.00 0.00 O2 C

ATOM 4086 H101 POPCO 1 -40.949 -39.375 0.610 1.00 0.00 O2 H

ATOM 4087 C211 POPCO 1 -43.022 -39.317 -0.024 1.00 0.00 O2 C

ATOM 4088 H11R POPCO 1 -43.092 -38.200 0.009 1.00 0.00 O2 H

ATOM 4089 H11S POPCO 1 -43.732 -39.677 -0.834 1.00 0.00 O2 H

ATOM 4090 C212 POPCO 1 -43.630 -39.772 1.370 1.00 0.00 O2 C

ATOM 4091 H12R POPCO 1 -44.711 -39.656 1.316 1.00 0.00 O2 H

ATOM 4092 H12S POPCO 1 -43.540 -40.935 1.370 1.00 0.00 O2 H

ATOM 4093 C213 POPCO 1 -42.952 -39.165 2.556 1.00 0.00 O2 C

ATOM 4094 H13R POPCO 1 -42.146 -39.758 2.849 1.00 0.00 O2 H

ATOM 4095 H13S POPCO 1 -42.629 -38.119 2.481 1.00 0.00 O2 H

ATOM 4096 C214 POPCO 1 -43.851 -39.251 3.777 1.00 0.00 O2 C

ATOM 4097 H14R POPCO 1 -44.634 -38.526 3.725 1.00 0.00 O2 H

ATOM 4098 H14S POPCO 1 -44.205 -40.298 3.756 1.00 0.00 O2 H

ATOM 4099 C215 POPCO 1 -43.021 -39.053 5.015 1.00 0.00 O2 C

ATOM 4100 H15R POPCO 1 -42.249 -39.825 5.108 1.00 0.00 O2 H

ATOM 4101 H15S POPCO 1 -42.471 -38.066 4.859 1.00 0.00 O2 H

ATOM 4102 C216 POPCO 1 -43.863 -38.876 6.293 1.00 0.00 O2 C

ATOM 4103 H16R POPCO 1 -44.709 -38.018 6.213 1.00 0.00 O2 H

ATOM 4104 H16S POPCO 1 -44.455 -39.806 6.588 1.00 0.00 O2 H

ATOM 4105 C217 POPCO 1 -43.076 -38.497 7.597 1.00 0.00 O2 C

ATOM 4106 H17R POPCO 1 -42.282 -39.267 7.730 1.00 0.00 O2 H

ATOM 4107 H17S POPCO 1 -42.660 -37.485 7.389 1.00 0.00 O2 H

ATOM 4108 C218 POPCO 1 -44.007 -38.414 8.822 1.00 0.00 O2 C

ATOM 4109 H18R POPCO 1 -44.742 -39.250 8.897 1.00 0.00 O2 H

ATOM 4110 H18S POPCO 1 -43.457 -38.290 9.727 1.00 0.00 O2 H

ATOM 4111 H18T POPCO 1 -44.641 -37.542 8.628 1.00 0.00 O2 H

ATOM 4112 C33 POPCO 1 -48.055 -40.375 -12.439 1.00 0.00 O2 C

ATOM 4113 H3X POPCO 1 -48.502 -39.405 -12.604 1.00 0.00 O2 H

ATOM 4114 H3Y POPCO 1 -48.858 -41.061 -12.296 1.00 0.00 O2 H

ATOM 4115 C34 POPCO 1 -47.163 -40.312 -11.142 1.00 0.00 O2 C

ATOM 4116 H4X POPCO 1 -46.588 -41.243 -10.959 1.00 0.00 O2 H

ATOM 4117 H4Y POPCO 1 -46.460 -39.461 -11.167 1.00 0.00 O2 H

ATOM 4118 C35 POPCO 1 -48.077 -40.089 -9.884 1.00 0.00 O2 C

ATOM 4119 H5X POPCO 1 -48.822 -39.339 -10.143 1.00 0.00 O2 H

ATOM 4120 H5Y POPCO 1 -48.543 -41.141 -9.876 1.00 0.00 O2 H

ATOM 4121 C36 POPCO 1 -47.214 -39.866 -8.611 1.00 0.00 O2 C

ATOM 4122 H6X POPCO 1 -46.733 -40.869 -8.428 1.00 0.00 O2 H

ATOM 4123 H6Y POPCO 1 -46.474 -39.053 -8.733 1.00 0.00 O2 H

ATOM 4124 C37 POPCO 1 -48.115 -39.550 -7.389 1.00 0.00 O2 C

ATOM 4125 H7X POPCO 1 -48.609 -38.613 -7.620 1.00 0.00 O2 H

ATOM 4126 H7Y POPCO 1 -48.900 -40.255 -7.211 1.00 0.00 O2 H

ATOM 4127 C38 POPCO 1 -47.225 -39.503 -6.037 1.00 0.00 O2 C

ATOM 4128 H8X POPCO 1 -46.567 -40.440 -5.876 1.00 0.00 O2 H

ATOM 4129 H8Y POPCO 1 -46.508 -38.616 -6.102 1.00 0.00 O2 H

ATOM 4130 C39 POPCO 1 -48.157 -39.314 -4.782 1.00 0.00 O2 C

ATOM 4131 H9X POPCO 1 -48.713 -38.330 -4.863 1.00 0.00 O2 H

ATOM 4132 H9Y POPCO 1 -48.886 -40.216 -4.898 1.00 0.00 O2 H

ATOM 4133 C310 POPCO 1 -47.402 -39.427 -3.475 1.00 0.00 O2 C

ATOM 4134 H10X POPCO 1 -46.778 -40.420 -3.516 1.00 0.00 O2 H

ATOM 4135 H10Y POPCO 1 -46.658 -38.630 -3.555 1.00 0.00 O2 H

ATOM 4136 C311 POPCO 1 -48.318 -39.307 -2.298 1.00 0.00 O2 C

ATOM 4137 H11X POPCO 1 -48.977 -38.426 -2.361 1.00 0.00 O2 H

ATOM 4138 H11Y POPCO 1 -48.984 -40.171 -2.269 1.00 0.00 O2 H

ATOM 4139 C312 POPCO 1 -47.504 -39.409 -1.033 1.00 0.00 O2 C

ATOM 4140 H12X POPCO 1 -46.833 -40.330 -1.033 1.00 0.00 O2 H

ATOM 4141 H12Y POPCO 1 -46.891 -38.447 -1.032 1.00 0.00 O2 H

ATOM 4142 C313 POPCO 1 -48.393 -39.506 0.249 1.00 0.00 O2 C

ATOM 4143 H13X POPCO 1 -48.932 -38.535 0.501 1.00 0.00 O2 H

ATOM 4144 H13Y POPCO 1 -49.169 -40.227 -0.018 1.00 0.00 O2 H

ATOM 4145 C314 POPCO 1 -47.575 -39.819 1.435 1.00 0.00 O2 C

ATOM 4146 H14X POPCO 1 -47.094 -40.758 1.279 1.00 0.00 O2 H

ATOM 4147 H14Y POPCO 1 -46.847 -39.033 1.514 1.00 0.00 O2 H

ATOM 4148 C315 POPCO 1 -48.359 -39.842 2.721 1.00 0.00 O2 C

ATOM 4149 H15X POPCO 1 -49.010 -38.892 2.750 1.00 0.00 O2 H

ATOM 4150 H15Y POPCO 1 -49.011 -40.766 2.793 1.00 0.00 O2 H

ATOM 4151 C316 POPCO 1 -47.464 -39.813 3.948 1.00 0.00 O2 C

ATOM 4152 H16X POPCO 1 -46.880 -38.949 4.114 1.00 0.00 O2 H

ATOM 4153 H16Y POPCO 1 -48.090 -39.732 4.842 1.00 0.00 O2 H

ATOM 4154 H16Z POPCO 1 -46.865 -40.764 4.049 1.00 0.00 O2 H

ATOM 4155 N POPCO 2 -38.759 -33.599 -22.631 1.00 0.00 O2 N

ATOM 4156 C12 POPCO 2 -38.010 -34.790 -22.118 1.00 0.00 O2 C

ATOM 4157 H12A POPCO 2 -37.330 -35.133 -22.904 1.00 0.00 O2 H

ATOM 4158 H12B POPCO 2 -38.618 -35.636 -21.922 1.00 0.00 O2 H

ATOM 4159 C13 POPCO 2 -37.888 -32.674 -23.477 1.00 0.00 O2 C

ATOM 4160 H13A POPCO 2 -37.249 -33.219 -24.182 1.00 0.00 O2 H

ATOM 4161 H13B POPCO 2 -37.067 -32.237 -22.835 1.00 0.00 O2 H

ATOM 4162 H13C POPCO 2 -38.474 -31.830 -23.932 1.00 0.00 O2 H

ATOM 4163 C14 POPCO 2 -39.838 -34.131 -23.510 1.00 0.00 O2 C

ATOM 4164 H14A POPCO 2 -40.662 -34.665 -23.011 1.00 0.00 O2 H

ATOM 4165 H14B POPCO 2 -39.355 -34.651 -24.288 1.00 0.00 O2 H

ATOM 4166 H14C POPCO 2 -40.357 -33.293 -23.949 1.00 0.00 O2 H

ATOM 4167 C15 POPCO 2 -39.331 -32.768 -21.537 1.00 0.00 O2 C

ATOM 4168 H15A POPCO 2 -39.986 -33.264 -20.894 1.00 0.00 O2 H

ATOM 4169 H15B POPCO 2 -38.434 -32.550 -20.874 1.00 0.00 O2 H

ATOM 4170 H15C POPCO 2 -39.701 -31.812 -21.775 1.00 0.00 O2 H

ATOM 4171 C11 POPCO 2 -36.976 -34.595 -20.874 1.00 0.00 O2 C

ATOM 4172 H11A POPCO 2 -37.594 -34.204 -20.082 1.00 0.00 O2 H

ATOM 4173 H11B POPCO 2 -36.450 -35.518 -20.561 1.00 0.00 O2 H

ATOM 4174 P POPCO 2 -35.562 -32.281 -20.628 1.00 0.00 O2 P

ATOM 4175 O13 POPCO 2 -35.788 -31.218 -21.585 1.00 0.00 O2 O

ATOM 4176 O14 POPCO 2 -34.229 -32.283 -19.989 1.00 0.00 O2 O

ATOM 4177 O12 POPCO 2 -35.940 -33.695 -21.321 1.00 0.00 O2 O

ATOM 4178 O11 POPCO 2 -36.654 -32.101 -19.475 1.00 0.00 O2 O

ATOM 4179 C1 POPCO 2 -36.354 -32.554 -18.135 1.00 0.00 O2 C

ATOM 4180 HA POPCO 2 -36.136 -33.662 -18.175 1.00 0.00 O2 H

ATOM 4181 HB POPCO 2 -35.567 -31.929 -17.721 1.00 0.00 O2 H

ATOM 4182 C2 POPCO 2 -37.594 -32.359 -17.248 1.00 0.00 O2 C

ATOM 4183 HS POPCO 2 -37.859 -31.268 -17.342 1.00 0.00 O2 H

ATOM 4184 O21 POPCO 2 -37.373 -32.767 -15.839 1.00 0.00 O2 O

ATOM 4185 C21 POPCO 2 -36.853 -31.840 -15.102 1.00 0.00 O2 C

ATOM 4186 O22 POPCO 2 -36.569 -30.724 -15.413 1.00 0.00 O2 O

ATOM 4187 C22 POPCO 2 -36.853 -32.277 -13.625 1.00 0.00 O2 C

ATOM 4188 H2R POPCO 2 -37.329 -33.280 -13.592 1.00 0.00 O2 H

ATOM 4189 H2S POPCO 2 -35.825 -32.347 -13.190 1.00 0.00 O2 H

ATOM 4190 C3 POPCO 2 -38.868 -33.117 -17.687 1.00 0.00 O2 C

ATOM 4191 HX POPCO 2 -39.658 -32.914 -16.861 1.00 0.00 O2 H

ATOM 4192 HY POPCO 2 -39.192 -32.716 -18.684 1.00 0.00 O2 H

ATOM 4193 O31 POPCO 2 -38.605 -34.497 -17.558 1.00 0.00 O2 O

ATOM 4194 C31 POPCO 2 -39.521 -35.211 -18.254 1.00 0.00 O2 C

ATOM 4195 O32 POPCO 2 -40.123 -34.847 -19.257 1.00 0.00 O2 O

ATOM 4196 C32 POPCO 2 -39.691 -36.547 -17.556 1.00 0.00 O2 C

ATOM 4197 H2X POPCO 2 -39.624 -37.310 -18.363 1.00 0.00 O2 H

ATOM 4198 H2Y POPCO 2 -38.834 -36.654 -16.844 1.00 0.00 O2 H

ATOM 4199 C23 POPCO 2 -37.643 -31.245 -12.725 1.00 0.00 O2 C

ATOM 4200 H3R POPCO 2 -37.053 -30.289 -12.549 1.00 0.00 O2 H

ATOM 4201 H3S POPCO 2 -38.537 -30.898 -13.159 1.00 0.00 O2 H

ATOM 4202 C24 POPCO 2 -37.930 -31.890 -11.346 1.00 0.00 O2 C

ATOM 4203 H4R POPCO 2 -38.594 -32.843 -11.462 1.00 0.00 O2 H

ATOM 4204 H4S POPCO 2 -36.974 -32.057 -10.831 1.00 0.00 O2 H

ATOM 4205 C25 POPCO 2 -38.716 -30.924 -10.449 1.00 0.00 O2 C

ATOM 4206 H5R POPCO 2 -38.331 -29.902 -10.659 1.00 0.00 O2 H

ATOM 4207 H5S POPCO 2 -39.755 -30.846 -10.837 1.00 0.00 O2 H

ATOM 4208 C26 POPCO 2 -38.725 -31.229 -8.998 1.00 0.00 O2 C

ATOM 4209 H6R POPCO 2 -39.324 -32.128 -8.841 1.00 0.00 O2 H

ATOM 4210 H6S POPCO 2 -37.704 -31.458 -8.643 1.00 0.00 O2 H

ATOM 4211 C27 POPCO 2 -39.242 -30.144 -8.093 1.00 0.00 O2 C

ATOM 4212 H7R POPCO 2 -38.754 -29.187 -8.400 1.00 0.00 O2 H

ATOM 4213 H7S POPCO 2 -40.352 -30.077 -8.064 1.00 0.00 O2 H

ATOM 4214 C28 POPCO 2 -38.806 -30.515 -6.674 1.00 0.00 O2 C

ATOM 4215 H8R POPCO 2 -39.194 -31.479 -6.479 1.00 0.00 O2 H

ATOM 4216 H8S POPCO 2 -37.671 -30.543 -6.572 1.00 0.00 O2 H

ATOM 4217 C29 POPCO 2 -39.251 -29.594 -5.650 1.00 0.00 O2 C

ATOM 4218 H91 POPCO 2 -39.466 -28.581 -5.996 1.00 0.00 O2 H

ATOM 4219 C210 POPCO 2 -39.301 -29.747 -4.277 1.00 0.00 O2 C

ATOM 4220 H101 POPCO 2 -39.670 -28.977 -3.562 1.00 0.00 O2 H

ATOM 4221 C211 POPCO 2 -38.858 -31.082 -3.587 1.00 0.00 O2 C

ATOM 4222 H11R POPCO 2 -38.690 -31.931 -4.328 1.00 0.00 O2 H

ATOM 4223 H11S POPCO 2 -37.962 -30.944 -2.968 1.00 0.00 O2 H

ATOM 4224 C212 POPCO 2 -39.974 -31.589 -2.578 1.00 0.00 O2 C

ATOM 4225 H12R POPCO 2 -41.019 -31.391 -2.966 1.00 0.00 O2 H

ATOM 4226 H12S POPCO 2 -39.938 -32.656 -2.351 1.00 0.00 O2 H

ATOM 4227 C213 POPCO 2 -39.908 -30.900 -1.236 1.00 0.00 O2 C

ATOM 4228 H13R POPCO 2 -38.905 -30.875 -0.905 1.00 0.00 O2 H

ATOM 4229 H13S POPCO 2 -40.344 -29.839 -1.348 1.00 0.00 O2 H

ATOM 4230 C214 POPCO 2 -40.814 -31.693 -0.270 1.00 0.00 O2 C

ATOM 4231 H14R POPCO 2 -41.874 -31.622 -0.621 1.00 0.00 O2 H

ATOM 4232 H14S POPCO 2 -40.666 -32.806 -0.297 1.00 0.00 O2 H

ATOM 4233 C215 POPCO 2 -40.557 -31.249 1.151 1.00 0.00 O2 C

ATOM 4234 H15R POPCO 2 -39.517 -31.402 1.472 1.00 0.00 O2 H

ATOM 4235 H15S POPCO 2 -40.652 -30.201 1.256 1.00 0.00 O2 H

ATOM 4236 C216 POPCO 2 -41.484 -32.067 2.070 1.00 0.00 O2 C

ATOM 4237 H16R POPCO 2 -42.571 -31.699 1.779 1.00 0.00 O2 H

ATOM 4238 H16S POPCO 2 -41.307 -33.152 1.896 1.00 0.00 O2 H

ATOM 4239 C217 POPCO 2 -41.234 -31.855 3.509 1.00 0.00 O2 C

ATOM 4240 H17R POPCO 2 -40.146 -32.077 3.799 1.00 0.00 O2 H

ATOM 4241 H17S POPCO 2 -41.462 -30.765 3.730 1.00 0.00 O2 H

ATOM 4242 C218 POPCO 2 -42.178 -32.634 4.474 1.00 0.00 O2 C

ATOM 4243 H18R POPCO 2 -41.838 -32.309 5.511 1.00 0.00 O2 H

ATOM 4244 H18S POPCO 2 -43.254 -32.485 4.376 1.00 0.00 O2 H

ATOM 4245 H18T POPCO 2 -41.983 -33.723 4.434 1.00 0.00 O2 H

ATOM 4246 C33 POPCO 2 -40.955 -36.741 -16.719 1.00 0.00 O2 C

ATOM 4247 H3X POPCO 2 -41.763 -36.656 -17.489 1.00 0.00 O2 H

ATOM 4248 H3Y POPCO 2 -40.998 -37.789 -16.291 1.00 0.00 O2 H

ATOM 4249 C34 POPCO 2 -41.060 -35.658 -15.629 1.00 0.00 O2 C

ATOM 4250 H4X POPCO 2 -40.011 -35.428 -15.105 1.00 0.00 O2 H

ATOM 4251 H4Y POPCO 2 -41.389 -34.701 -15.946 1.00 0.00 O2 H

ATOM 4252 C35 POPCO 2 -41.750 -36.266 -14.318 1.00 0.00 O2 C

ATOM 4253 H5X POPCO 2 -42.843 -36.264 -14.440 1.00 0.00 O2 H

ATOM 4254 H5Y POPCO 2 -41.288 -37.236 -14.236 1.00 0.00 O2 H

ATOM 4255 C36 POPCO 2 -41.402 -35.455 -13.060 1.00 0.00 O2 C

ATOM 4256 H6X POPCO 2 -40.324 -35.125 -13.110 1.00 0.00 O2 H

ATOM 4257 H6Y POPCO 2 -42.051 -34.539 -13.174 1.00 0.00 O2 H

ATOM 4258 C37 POPCO 2 -41.686 -36.207 -11.806 1.00 0.00 O2 C

ATOM 4259 H7X POPCO 2 -42.781 -36.613 -11.753 1.00 0.00 O2 H

ATOM 4260 H7Y POPCO 2 -41.074 -37.125 -11.556 1.00 0.00 O2 H

ATOM 4261 C38 POPCO 2 -41.553 -35.287 -10.566 1.00 0.00 O2 C

ATOM 4262 H8X POPCO 2 -40.489 -34.998 -10.399 1.00 0.00 O2 H

ATOM 4263 H8Y POPCO 2 -42.164 -34.345 -10.749 1.00 0.00 O2 H

ATOM 4264 C39 POPCO 2 -42.176 -35.963 -9.291 1.00 0.00 O2 C

ATOM 4265 H9X POPCO 2 -43.165 -36.315 -9.364 1.00 0.00 O2 H

ATOM 4266 H9Y POPCO 2 -41.632 -36.860 -8.988 1.00 0.00 O2 H

ATOM 4267 C310 POPCO 2 -42.205 -35.048 -8.066 1.00 0.00 O2 C

ATOM 4268 H10X POPCO 2 -41.125 -34.857 -7.839 1.00 0.00 O2 H

ATOM 4269 H10Y POPCO 2 -42.687 -34.056 -8.393 1.00 0.00 O2 H

ATOM 4270 C311 POPCO 2 -42.945 -35.623 -6.869 1.00 0.00 O2 C

ATOM 4271 H11X POPCO 2 -44.014 -35.657 -7.134 1.00 0.00 O2 H

ATOM 4272 H11Y POPCO 2 -42.586 -36.659 -6.712 1.00 0.00 O2 H

ATOM 4273 C312 POPCO 2 -42.797 -34.861 -5.534 1.00 0.00 O2 C

ATOM 4274 H12X POPCO 2 -41.692 -34.650 -5.339 1.00 0.00 O2 H

ATOM 4275 H12Y POPCO 2 -43.280 -33.857 -5.660 1.00 0.00 O2 H

ATOM 4276 C313 POPCO 2 -43.439 -35.637 -4.392 1.00 0.00 O2 C

ATOM 4277 H13X POPCO 2 -44.573 -35.741 -4.596 1.00 0.00 O2 H

ATOM 4278 H13Y POPCO 2 -42.964 -36.596 -4.269 1.00 0.00 O2 H

ATOM 4279 C314 POPCO 2 -43.289 -34.854 -3.072 1.00 0.00 O2 C

ATOM 4280 H14X POPCO 2 -42.267 -34.596 -2.787 1.00 0.00 O2 H

ATOM 4281 H14Y POPCO 2 -43.761 -33.865 -3.270 1.00 0.00 O2 H

ATOM 4282 C315 POPCO 2 -44.000 -35.532 -1.869 1.00 0.00 O2 C

ATOM 4283 H15X POPCO 2 -44.996 -35.949 -2.256 1.00 0.00 O2 H

ATOM 4284 H15Y POPCO 2 -43.422 -36.522 -1.689 1.00 0.00 O2 H

ATOM 4285 C316 POPCO 2 -44.054 -34.638 -0.612 1.00 0.00 O2 C

ATOM 4286 H16X POPCO 2 -44.754 -33.787 -0.908 1.00 0.00 O2 H

ATOM 4287 H16Y POPCO 2 -44.453 -35.092 0.308 1.00 0.00 O2 H

ATOM 4288 H16Z POPCO 2 -42.996 -34.288 -0.540 1.00 0.00 O2 H

ATOM 4289 N POPCO 3 -40.645 -24.062 -19.341 1.00 0.00 O2 N

ATOM 4290 C12 POPCO 3 -41.384 -24.146 -18.055 1.00 0.00 O2 C

ATOM 4291 H12A POPCO 3 -42.111 -23.332 -18.072 1.00 0.00 O2 H

ATOM 4292 H12B POPCO 3 -40.652 -23.885 -17.200 1.00 0.00 O2 H

ATOM 4293 C13 POPCO 3 -41.723 -23.771 -20.367 1.00 0.00 O2 C

ATOM 4294 H13A POPCO 3 -42.251 -22.847 -20.080 1.00 0.00 O2 H

ATOM 4295 H13B POPCO 3 -42.406 -24.566 -20.323 1.00 0.00 O2 H

ATOM 4296 H13C POPCO 3 -41.315 -23.897 -21.377 1.00 0.00 O2 H

ATOM 4297 C14 POPCO 3 -39.612 -23.006 -19.278 1.00 0.00 O2 C

ATOM 4298 H14A POPCO 3 -38.807 -23.250 -18.535 1.00 0.00 O2 H

ATOM 4299 H14B POPCO 3 -40.076 -22.116 -18.905 1.00 0.00 O2 H

ATOM 4300 H14C POPCO 3 -39.317 -22.750 -20.285 1.00 0.00 O2 H

ATOM 4301 C15 POPCO 3 -40.089 -25.403 -19.561 1.00 0.00 O2 C

ATOM 4302 H15A POPCO 3 -39.442 -25.647 -18.687 1.00 0.00 O2 H

ATOM 4303 H15B POPCO 3 -40.927 -26.041 -19.517 1.00 0.00 O2 H

ATOM 4304 H15C POPCO 3 -39.423 -25.505 -20.447 1.00 0.00 O2 H

ATOM 4305 C11 POPCO 3 -42.117 -25.483 -17.582 1.00 0.00 O2 C

ATOM 4306 H11A POPCO 3 -41.491 -26.372 -17.548 1.00 0.00 O2 H

ATOM 4307 H11B POPCO 3 -42.348 -25.345 -16.455 1.00 0.00 O2 H

ATOM 4308 P POPCO 3 -43.698 -26.752 -19.370 1.00 0.00 O2 P

ATOM 4309 O13 POPCO 3 -42.641 -26.659 -20.410 1.00 0.00 O2 O

ATOM 4310 O14 POPCO 3 -45.091 -26.551 -19.819 1.00 0.00 O2 O

ATOM 4311 O12 POPCO 3 -43.405 -25.673 -18.205 1.00 0.00 O2 O

ATOM 4312 O11 POPCO 3 -43.448 -28.085 -18.519 1.00 0.00 O2 O

ATOM 4313 C1 POPCO 3 -44.046 -28.093 -17.205 1.00 0.00 O2 C

ATOM 4314 HA POPCO 3 -43.604 -27.290 -16.599 1.00 0.00 O2 H

ATOM 4315 HB POPCO 3 -45.158 -28.070 -17.257 1.00 0.00 O2 H

ATOM 4316 C2 POPCO 3 -43.652 -29.444 -16.507 1.00 0.00 O2 C

ATOM 4317 HS POPCO 3 -44.218 -30.293 -16.905 1.00 0.00 O2 H

ATOM 4318 O21 POPCO 3 -43.911 -29.127 -15.143 1.00 0.00 O2 O

ATOM 4319 C21 POPCO 3 -43.962 -30.017 -14.143 1.00 0.00 O2 C

ATOM 4320 O22 POPCO 3 -43.710 -31.157 -14.271 1.00 0.00 O2 O

ATOM 4321 C22 POPCO 3 -44.174 -29.274 -12.793 1.00 0.00 O2 C

ATOM 4322 H2R POPCO 3 -43.475 -28.380 -12.794 1.00 0.00 O2 H

ATOM 4323 H2S POPCO 3 -45.181 -28.874 -12.787 1.00 0.00 O2 H

ATOM 4324 C3 POPCO 3 -42.118 -29.771 -16.583 1.00 0.00 O2 C

ATOM 4325 HX POPCO 3 -42.076 -30.748 -16.054 1.00 0.00 O2 H

ATOM 4326 HY POPCO 3 -41.875 -29.962 -17.721 1.00 0.00 O2 H

ATOM 4327 O31 POPCO 3 -41.165 -28.801 -16.009 1.00 0.00 O2 O

ATOM 4328 C31 POPCO 3 -40.094 -29.278 -15.422 1.00 0.00 O2 C

ATOM 4329 O32 POPCO 3 -39.939 -30.471 -15.068 1.00 0.00 O2 O

ATOM 4330 C32 POPCO 3 -39.080 -28.203 -15.094 1.00 0.00 O2 C

ATOM 4331 H2X POPCO 3 -38.562 -28.394 -14.144 1.00 0.00 O2 H

ATOM 4332 H2Y POPCO 3 -38.450 -28.247 -15.955 1.00 0.00 O2 H

ATOM 4333 C23 POPCO 3 -43.894 -30.180 -11.530 1.00 0.00 O2 C

ATOM 4334 H3R POPCO 3 -44.702 -30.924 -11.454 1.00 0.00 O2 H

ATOM 4335 H3S POPCO 3 -42.913 -30.719 -11.542 1.00 0.00 O2 H

ATOM 4336 C24 POPCO 3 -43.978 -29.319 -10.309 1.00 0.00 O2 C

ATOM 4337 H4R POPCO 3 -43.104 -28.605 -10.285 1.00 0.00 O2 H

ATOM 4338 H4S POPCO 3 -44.885 -28.801 -10.356 1.00 0.00 O2 H

ATOM 4339 C25 POPCO 3 -43.963 -30.090 -8.930 1.00 0.00 O2 C

ATOM 4340 H5R POPCO 3 -44.923 -30.585 -8.936 1.00 0.00 O2 H

ATOM 4341 H5S POPCO 3 -43.099 -30.817 -8.899 1.00 0.00 O2 H

ATOM 4342 C26 POPCO 3 -43.733 -29.097 -7.760 1.00 0.00 O2 C

ATOM 4343 H6R POPCO 3 -42.827 -28.469 -7.787 1.00 0.00 O2 H

ATOM 4344 H6S POPCO 3 -44.566 -28.437 -7.694 1.00 0.00 O2 H

ATOM 4345 C27 POPCO 3 -43.596 -29.915 -6.468 1.00 0.00 O2 C

ATOM 4346 H7R POPCO 3 -44.454 -30.604 -6.381 1.00 0.00 O2 H

ATOM 4347 H7S POPCO 3 -42.677 -30.609 -6.429 1.00 0.00 O2 H

ATOM 4348 C28 POPCO 3 -43.652 -28.896 -5.332 1.00 0.00 O2 C

ATOM 4349 H8R POPCO 3 -42.777 -28.180 -5.333 1.00 0.00 O2 H

ATOM 4350 H8S POPCO 3 -44.559 -28.278 -5.529 1.00 0.00 O2 H

ATOM 4351 C29 POPCO 3 -43.694 -29.670 -4.036 1.00 0.00 O2 C

ATOM 4352 H91 POPCO 3 -44.231 -30.660 -4.044 1.00 0.00 O2 H

ATOM 4353 C210 POPCO 3 -43.159 -29.415 -2.796 1.00 0.00 O2 C

ATOM 4354 H101 POPCO 3 -43.193 -30.070 -1.909 1.00 0.00 O2 H

ATOM 4355 C211 POPCO 3 -42.361 -28.089 -2.418 1.00 0.00 O2 C

ATOM 4356 H11R POPCO 3 -41.276 -28.336 -2.547 1.00 0.00 O2 H

ATOM 4357 H11S POPCO 3 -42.490 -27.366 -3.226 1.00 0.00 O2 H

ATOM 4358 C212 POPCO 3 -42.683 -27.496 -1.049 1.00 0.00 O2 C

ATOM 4359 H12R POPCO 3 -42.140 -26.501 -1.075 1.00 0.00 O2 H

ATOM 4360 H12S POPCO 3 -43.749 -27.340 -1.134 1.00 0.00 O2 H

ATOM 4361 C213 POPCO 3 -42.354 -28.303 0.189 1.00 0.00 O2 C

ATOM 4362 H13R POPCO 3 -43.111 -29.118 0.291 1.00 0.00 O2 H

ATOM 4363 H13S POPCO 3 -41.343 -28.765 0.046 1.00 0.00 O2 H

ATOM 4364 C214 POPCO 3 -42.431 -27.439 1.505 1.00 0.00 O2 C

ATOM 4365 H14R POPCO 3 -41.551 -26.789 1.569 1.00 0.00 O2 H

ATOM 4366 H14S POPCO 3 -43.429 -26.857 1.380 1.00 0.00 O2 H

ATOM 4367 C215 POPCO 3 -42.409 -28.340 2.803 1.00 0.00 O2 C

ATOM 4368 H15R POPCO 3 -43.284 -29.020 2.731 1.00 0.00 O2 H

ATOM 4369 H15S POPCO 3 -41.451 -28.895 2.768 1.00 0.00 O2 H

ATOM 4370 C216 POPCO 3 -42.604 -27.661 4.108 1.00 0.00 O2 C

ATOM 4371 H16R POPCO 3 -41.804 -26.916 4.152 1.00 0.00 O2 H

ATOM 4372 H16S POPCO 3 -43.552 -27.126 4.168 1.00 0.00 O2 H

ATOM 4373 C217 POPCO 3 -42.343 -28.585 5.343 1.00 0.00 O2 C

ATOM 4374 H17R POPCO 3 -43.234 -29.263 5.306 1.00 0.00 O2 H

ATOM 4375 H17S POPCO 3 -41.520 -29.275 5.105 1.00 0.00 O2 H

ATOM 4376 C218 POPCO 3 -42.165 -27.895 6.747 1.00 0.00 O2 C

ATOM 4377 H18R POPCO 3 -42.290 -26.799 6.667 1.00 0.00 O2 H

ATOM 4378 H18S POPCO 3 -42.856 -28.384 7.510 1.00 0.00 O2 H

ATOM 4379 H18T POPCO 3 -41.073 -28.138 7.094 1.00 0.00 O2 H

ATOM 4380 C33 POPCO 3 -39.616 -26.747 -15.074 1.00 0.00 O2 C

ATOM 4381 H3X POPCO 3 -38.780 -26.136 -14.667 1.00 0.00 O2 H

ATOM 4382 H3Y POPCO 3 -39.932 -26.493 -16.106 1.00 0.00 O2 H

ATOM 4383 C34 POPCO 3 -40.915 -26.443 -14.185 1.00 0.00 O2 C

ATOM 4384 H4X POPCO 3 -41.512 -25.586 -14.681 1.00 0.00 O2 H

ATOM 4385 H4Y POPCO 3 -41.587 -27.313 -14.124 1.00 0.00 O2 H

ATOM 4386 C35 POPCO 3 -40.498 -25.980 -12.736 1.00 0.00 O2 C

ATOM 4387 H5X POPCO 3 -39.695 -26.650 -12.425 1.00 0.00 O2 H

ATOM 4388 H5Y POPCO 3 -39.961 -24.986 -12.822 1.00 0.00 O2 H

ATOM 4389 C36 POPCO 3 -41.725 -25.814 -11.735 1.00 0.00 O2 C

ATOM 4390 H6X POPCO 3 -42.559 -25.392 -12.429 1.00 0.00 O2 H

ATOM 4391 H6Y POPCO 3 -42.043 -26.881 -11.539 1.00 0.00 O2 H

ATOM 4392 C37 POPCO 3 -41.344 -25.006 -10.523 1.00 0.00 O2 C

ATOM 4393 H7X POPCO 3 -40.346 -25.375 -10.188 1.00 0.00 O2 H

ATOM 4394 H7Y POPCO 3 -41.115 -23.999 -10.852 1.00 0.00 O2 H

ATOM 4395 C38 POPCO 3 -42.239 -25.309 -9.356 1.00 0.00 O2 C

ATOM 4396 H8X POPCO 3 -43.251 -24.972 -9.691 1.00 0.00 O2 H

ATOM 4397 H8Y POPCO 3 -42.365 -26.389 -9.054 1.00 0.00 O2 H

ATOM 4398 C39 POPCO 3 -41.760 -24.451 -8.076 1.00 0.00 O2 C

ATOM 4399 H9X POPCO 3 -40.619 -24.395 -7.886 1.00 0.00 O2 H

ATOM 4400 H9Y POPCO 3 -42.216 -23.432 -8.307 1.00 0.00 O2 H

ATOM 4401 C310 POPCO 3 -42.431 -24.973 -6.741 1.00 0.00 O2 C

ATOM 4402 H10X POPCO 3 -43.435 -25.314 -6.956 1.00 0.00 O2 H

ATOM 4403 H10Y POPCO 3 -41.694 -25.793 -6.403 1.00 0.00 O2 H

ATOM 4404 C311 POPCO 3 -42.434 -23.968 -5.589 1.00 0.00 O2 C

ATOM 4405 H11X POPCO 3 -41.549 -23.272 -5.671 1.00 0.00 O2 H

ATOM 4406 H11Y POPCO 3 -43.216 -23.328 -5.948 1.00 0.00 O2 H

ATOM 4407 C312 POPCO 3 -42.668 -24.569 -4.218 1.00 0.00 O2 C

ATOM 4408 H12X POPCO 3 -43.338 -25.418 -4.320 1.00 0.00 O2 H

ATOM 4409 H12Y POPCO 3 -41.655 -24.957 -3.845 1.00 0.00 O2 H

ATOM 4410 C313 POPCO 3 -43.232 -23.553 -3.285 1.00 0.00 O2 C

ATOM 4411 H13X POPCO 3 -42.646 -22.594 -3.357 1.00 0.00 O2 H

ATOM 4412 H13Y POPCO 3 -44.296 -23.255 -3.554 1.00 0.00 O2 H

ATOM 4413 C314 POPCO 3 -43.053 -24.059 -1.835 1.00 0.00 O2 C

ATOM 4414 H14X POPCO 3 -43.664 -24.942 -1.783 1.00 0.00 O2 H

ATOM 4415 H14Y POPCO 3 -42.020 -24.323 -1.570 1.00 0.00 O2 H

ATOM 4416 C315 POPCO 3 -43.657 -23.153 -0.729 1.00 0.00 O2 C

ATOM 4417 H15X POPCO 3 -43.088 -22.151 -0.692 1.00 0.00 O2 H

ATOM 4418 H15Y POPCO 3 -44.734 -22.876 -1.039 1.00 0.00 O2 H

ATOM 4419 C316 POPCO 3 -43.548 -23.888 0.672 1.00 0.00 O2 C

ATOM 4420 H16X POPCO 3 -42.617 -23.686 1.244 1.00 0.00 O2 H

ATOM 4421 H16Y POPCO 3 -44.402 -23.596 1.332 1.00 0.00 O2 H

ATOM 4422 H16Z POPCO 3 -43.727 -24.955 0.396 1.00 0.00 O2 H

ATOM 4423 N POPCO 4 -35.917 -17.335 -18.439 1.00 0.00 O2 N

ATOM 4424 C12 POPCO 4 -36.769 -17.689 -17.242 1.00 0.00 O2 C

ATOM 4425 H12A POPCO 4 -36.433 -16.968 -16.469 1.00 0.00 O2 H

ATOM 4426 H12B POPCO 4 -36.449 -18.682 -16.959 1.00 0.00 O2 H

ATOM 4427 C13 POPCO 4 -35.988 -15.944 -18.824 1.00 0.00 O2 C

ATOM 4428 H13A POPCO 4 -35.684 -15.180 -18.149 1.00 0.00 O2 H

ATOM 4429 H13B POPCO 4 -37.009 -15.713 -18.932 1.00 0.00 O2 H

ATOM 4430 H13C POPCO 4 -35.532 -15.802 -19.788 1.00 0.00 O2 H

ATOM 4431 C14 POPCO 4 -34.430 -17.686 -18.090 1.00 0.00 O2 C

ATOM 4432 H14A POPCO 4 -34.338 -18.684 -17.698 1.00 0.00 O2 H

ATOM 4433 H14B POPCO 4 -33.944 -17.017 -17.388 1.00 0.00 O2 H

ATOM 4434 H14C POPCO 4 -33.876 -17.605 -19.044 1.00 0.00 O2 H

ATOM 4435 C15 POPCO 4 -36.369 -18.159 -19.568 1.00 0.00 O2 C

ATOM 4436 H15A POPCO 4 -36.272 -19.225 -19.411 1.00 0.00 O2 H

ATOM 4437 H15B POPCO 4 -37.410 -17.836 -19.846 1.00 0.00 O2 H

ATOM 4438 H15C POPCO 4 -35.922 -17.874 -20.578 1.00 0.00 O2 H

ATOM 4439 C11 POPCO 4 -38.300 -17.689 -17.438 1.00 0.00 O2 C

ATOM 4440 H11A POPCO 4 -38.606 -18.402 -18.227 1.00 0.00 O2 H

ATOM 4441 H11B POPCO 4 -38.803 -18.032 -16.476 1.00 0.00 O2 H

ATOM 4442 P POPCO 4 -39.965 -16.226 -18.744 1.00 0.00 O2 P

ATOM 4443 O13 POPCO 4 -39.542 -16.881 -19.980 1.00 0.00 O2 O

ATOM 4444 O14 POPCO 4 -40.483 -14.838 -18.927 1.00 0.00 O2 O

ATOM 4445 O12 POPCO 4 -38.812 -16.344 -17.704 1.00 0.00 O2 O

ATOM 4446 O11 POPCO 4 -41.150 -17.111 -18.120 1.00 0.00 O2 O

ATOM 4447 C1 POPCO 4 -41.383 -16.930 -16.710 1.00 0.00 O2 C

ATOM 4448 HA POPCO 4 -40.516 -16.931 -16.168 1.00 0.00 O2 H

ATOM 4449 HB POPCO 4 -41.851 -15.929 -16.529 1.00 0.00 O2 H

ATOM 4450 C2 POPCO 4 -42.357 -17.885 -16.022 1.00 0.00 O2 C

ATOM 4451 HS POPCO 4 -43.309 -17.556 -16.417 1.00 0.00 O2 H

ATOM 4452 O21 POPCO 4 -42.263 -17.554 -14.579 1.00 0.00 O2 O

ATOM 4453 C21 POPCO 4 -42.539 -18.439 -13.623 1.00 0.00 O2 C

ATOM 4454 O22 POPCO 4 -43.129 -19.517 -13.674 1.00 0.00 O2 O

ATOM 4455 C22 POPCO 4 -41.941 -17.737 -12.353 1.00 0.00 O2 C

ATOM 4456 H2R POPCO 4 -40.898 -17.392 -12.389 1.00 0.00 O2 H

ATOM 4457 H2S POPCO 4 -42.599 -16.891 -12.140 1.00 0.00 O2 H

ATOM 4458 C3 POPCO 4 -42.153 -19.328 -16.417 1.00 0.00 O2 C

ATOM 4459 HX POPCO 4 -42.959 -19.928 -15.842 1.00 0.00 O2 H

ATOM 4460 HY POPCO 4 -42.399 -19.465 -17.437 1.00 0.00 O2 H

ATOM 4461 O31 POPCO 4 -40.747 -19.740 -16.098 1.00 0.00 O2 O

ATOM 4462 C31 POPCO 4 -40.500 -20.914 -16.477 1.00 0.00 O2 C

ATOM 4463 O32 POPCO 4 -41.207 -21.726 -17.069 1.00 0.00 O2 O

ATOM 4464 C32 POPCO 4 -38.987 -21.322 -16.133 1.00 0.00 O2 C

ATOM 4465 H2X POPCO 4 -38.647 -21.965 -16.950 1.00 0.00 O2 H

ATOM 4466 H2Y POPCO 4 -38.443 -20.351 -16.093 1.00 0.00 O2 H

ATOM 4467 C23 POPCO 4 -42.045 -18.725 -11.128 1.00 0.00 O2 C

ATOM 4468 H3R POPCO 4 -43.067 -19.134 -11.159 1.00 0.00 O2 H

ATOM 4469 H3S POPCO 4 -41.398 -19.545 -11.361 1.00 0.00 O2 H

ATOM 4470 C24 POPCO 4 -41.722 -18.208 -9.693 1.00 0.00 O2 C

ATOM 4471 H4R POPCO 4 -40.630 -18.069 -9.669 1.00 0.00 O2 H

ATOM 4472 H4S POPCO 4 -42.287 -17.288 -9.393 1.00 0.00 O2 H

ATOM 4473 C25 POPCO 4 -42.227 -19.228 -8.663 1.00 0.00 O2 C

ATOM 4474 H5R POPCO 4 -43.315 -19.376 -8.918 1.00 0.00 O2 H

ATOM 4475 H5S POPCO 4 -41.640 -20.231 -8.849 1.00 0.00 O2 H

ATOM 4476 C26 POPCO 4 -41.811 -18.883 -7.282 1.00 0.00 O2 C

ATOM 4477 H6R POPCO 4 -40.794 -19.063 -7.036 1.00 0.00 O2 H

ATOM 4478 H6S POPCO 4 -42.112 -17.854 -7.058 1.00 0.00 O2 H

ATOM 4479 C27 POPCO 4 -42.704 -19.687 -6.279 1.00 0.00 O2 C

ATOM 4480 H7R POPCO 4 -43.750 -19.485 -6.600 1.00 0.00 O2 H

ATOM 4481 H7S POPCO 4 -42.616 -20.789 -6.479 1.00 0.00 O2 H

ATOM 4482 C28 POPCO 4 -42.276 -19.305 -4.825 1.00 0.00 O2 C

ATOM 4483 H8R POPCO 4 -41.277 -19.823 -4.612 1.00 0.00 O2 H

ATOM 4484 H8S POPCO 4 -41.994 -18.208 -4.862 1.00 0.00 O2 H

ATOM 4485 C29 POPCO 4 -43.339 -19.750 -3.913 1.00 0.00 O2 C

ATOM 4486 H91 POPCO 4 -44.060 -20.597 -4.182 1.00 0.00 O2 H

ATOM 4487 C210 POPCO 4 -43.417 -19.273 -2.676 1.00 0.00 O2 C

ATOM 4488 H101 POPCO 4 -44.123 -19.740 -1.962 1.00 0.00 O2 H

ATOM 4489 C211 POPCO 4 -42.512 -18.200 -2.027 1.00 0.00 O2 C

ATOM 4490 H11R POPCO 4 -41.638 -18.009 -2.771 1.00 0.00 O2 H

ATOM 4491 H11S POPCO 4 -43.131 -17.380 -1.795 1.00 0.00 O2 H

ATOM 4492 C212 POPCO 4 -41.930 -18.533 -0.655 1.00 0.00 O2 C

ATOM 4493 H12R POPCO 4 -41.618 -19.583 -0.873 1.00 0.00 O2 H

ATOM 4494 H12S POPCO 4 -41.041 -17.980 -0.421 1.00 0.00 O2 H

ATOM 4495 C213 POPCO 4 -42.935 -18.454 0.525 1.00 0.00 O2 C

ATOM 4496 H13R POPCO 4 -43.323 -17.360 0.661 1.00 0.00 O2 H

ATOM 4497 H13S POPCO 4 -43.747 -19.215 0.381 1.00 0.00 O2 H

ATOM 4498 C214 POPCO 4 -42.125 -18.767 1.860 1.00 0.00 O2 C

ATOM 4499 H14R POPCO 4 -41.929 -19.877 1.829 1.00 0.00 O2 H

ATOM 4500 H14S POPCO 4 -41.200 -18.152 1.792 1.00 0.00 O2 H

ATOM 4501 C215 POPCO 4 -42.953 -18.638 3.150 1.00 0.00 O2 C

ATOM 4502 H15R POPCO 4 -42.991 -17.551 3.471 1.00 0.00 O2 H

ATOM 4503 H15S POPCO 4 -43.934 -19.036 2.932 1.00 0.00 O2 H

ATOM 4504 C216 POPCO 4 -42.302 -19.417 4.358 1.00 0.00 O2 C

ATOM 4505 H16R POPCO 4 -42.117 -20.479 3.938 1.00 0.00 O2 H

ATOM 4506 H16S POPCO 4 -41.360 -18.953 4.480 1.00 0.00 O2 H

ATOM 4507 C217 POPCO 4 -43.190 -19.564 5.624 1.00 0.00 O2 C

ATOM 4508 H17R POPCO 4 -43.345 -18.581 6.177 1.00 0.00 O2 H

ATOM 4509 H17S POPCO 4 -44.232 -19.894 5.407 1.00 0.00 O2 H

ATOM 4510 C218 POPCO 4 -42.493 -20.541 6.621 1.00 0.00 O2 C

ATOM 4511 H18R POPCO 4 -43.113 -20.656 7.541 1.00 0.00 O2 H

ATOM 4512 H18S POPCO 4 -42.436 -21.501 6.094 1.00 0.00 O2 H

ATOM 4513 H18T POPCO 4 -41.524 -20.211 6.903 1.00 0.00 O2 H

ATOM 4514 C33 POPCO 4 -38.793 -22.090 -14.784 1.00 0.00 O2 C

ATOM 4515 H3X POPCO 4 -39.306 -23.094 -14.661 1.00 0.00 O2 H

ATOM 4516 H3Y POPCO 4 -37.654 -22.277 -14.837 1.00 0.00 O2 H

ATOM 4517 C34 POPCO 4 -39.145 -21.259 -13.513 1.00 0.00 O2 C

ATOM 4518 H4X POPCO 4 -38.802 -20.213 -13.552 1.00 0.00 O2 H

ATOM 4519 H4Y POPCO 4 -40.230 -21.293 -13.379 1.00 0.00 O2 H

ATOM 4520 C35 POPCO 4 -38.402 -21.841 -12.220 1.00 0.00 O2 C

ATOM 4521 H5X POPCO 4 -38.417 -22.953 -12.272 1.00 0.00 O2 H

ATOM 4522 H5Y POPCO 4 -37.341 -21.415 -12.308 1.00 0.00 O2 H

ATOM 4523 C36 POPCO 4 -39.128 -21.403 -10.894 1.00 0.00 O2 C

ATOM 4524 H6X POPCO 4 -39.169 -20.268 -10.854 1.00 0.00 O2 H

ATOM 4525 H6Y POPCO 4 -40.109 -21.778 -10.889 1.00 0.00 O2 H

ATOM 4526 C37 POPCO 4 -38.426 -21.921 -9.687 1.00 0.00 O2 C

ATOM 4527 H7X POPCO 4 -38.170 -22.951 -9.870 1.00 0.00 O2 H

ATOM 4528 H7Y POPCO 4 -37.436 -21.480 -9.419 1.00 0.00 O2 H

ATOM 4529 C38 POPCO 4 -39.209 -21.688 -8.390 1.00 0.00 O2 C

ATOM 4530 H8X POPCO 4 -39.534 -20.683 -8.197 1.00 0.00 O2 H

ATOM 4531 H8Y POPCO 4 -40.058 -22.354 -8.394 1.00 0.00 O2 H

ATOM 4532 C39 POPCO 4 -38.292 -21.958 -7.154 1.00 0.00 O2 C

ATOM 4533 H9X POPCO 4 -37.962 -22.999 -7.133 1.00 0.00 O2 H

ATOM 4534 H9Y POPCO 4 -37.421 -21.349 -7.308 1.00 0.00 O2 H

ATOM 4535 C310 POPCO 4 -39.057 -21.533 -5.848 1.00 0.00 O2 C

ATOM 4536 H10X POPCO 4 -38.728 -20.535 -5.539 1.00 0.00 O2 H

ATOM 4537 H10Y POPCO 4 -40.168 -21.405 -5.874 1.00 0.00 O2 H

ATOM 4538 C311 POPCO 4 -38.795 -22.503 -4.751 1.00 0.00 O2 C

ATOM 4539 H11X POPCO 4 -39.145 -23.470 -5.116 1.00 0.00 O2 H

ATOM 4540 H11Y POPCO 4 -37.750 -22.503 -4.595 1.00 0.00 O2 H

ATOM 4541 C312 POPCO 4 -39.465 -21.979 -3.513 1.00 0.00 O2 C

ATOM 4542 H12X POPCO 4 -39.172 -20.957 -3.357 1.00 0.00 O2 H

ATOM 4543 H12Y POPCO 4 -40.539 -21.977 -3.604 1.00 0.00 O2 H

ATOM 4544 C313 POPCO 4 -39.007 -22.779 -2.270 1.00 0.00 O2 C

ATOM 4545 H13X POPCO 4 -39.401 -23.851 -2.350 1.00 0.00 O2 H

ATOM 4546 H13Y POPCO 4 -37.869 -22.926 -2.265 1.00 0.00 O2 H

ATOM 4547 C314 POPCO 4 -39.619 -22.258 -0.964 1.00 0.00 O2 C

ATOM 4548 H14X POPCO 4 -39.464 -21.117 -0.797 1.00 0.00 O2 H

ATOM 4549 H14Y POPCO 4 -40.763 -22.436 -0.879 1.00 0.00 O2 H

ATOM 4550 C315 POPCO 4 -38.998 -22.894 0.274 1.00 0.00 O2 C

ATOM 4551 H15X POPCO 4 -39.165 -23.935 0.170 1.00 0.00 O2 H

ATOM 4552 H15Y POPCO 4 -37.899 -22.760 0.330 1.00 0.00 O2 H

ATOM 4553 C316 POPCO 4 -39.680 -22.382 1.544 1.00 0.00 O2 C

ATOM 4554 H16X POPCO 4 -40.776 -22.625 1.521 1.00 0.00 O2 H

ATOM 4555 H16Y POPCO 4 -39.242 -22.852 2.451 1.00 0.00 O2 H

ATOM 4556 H16Z POPCO 4 -39.776 -21.307 1.592 1.00 0.00 O2 H

ATOM 4557 N POPCO 5 -43.616 -14.527 -20.864 1.00 0.00 O2 N

ATOM 4558 C12 POPCO 5 -42.434 -13.554 -20.562 1.00 0.00 O2 C

ATOM 4559 H12A POPCO 5 -42.004 -13.179 -21.454 1.00 0.00 O2 H

ATOM 4560 H12B POPCO 5 -41.623 -14.120 -20.099 1.00 0.00 O2 H

ATOM 4561 C13 POPCO 5 -44.448 -13.937 -21.979 1.00 0.00 O2 C

ATOM 4562 H13A POPCO 5 -43.881 -13.744 -22.846 1.00 0.00 O2 H

ATOM 4563 H13B POPCO 5 -44.783 -12.894 -21.642 1.00 0.00 O2 H

ATOM 4564 H13C POPCO 5 -45.322 -14.500 -22.235 1.00 0.00 O2 H

ATOM 4565 C14 POPCO 5 -43.140 -15.890 -21.276 1.00 0.00 O2 C

ATOM 4566 H14A POPCO 5 -42.369 -16.245 -20.555 1.00 0.00 O2 H

ATOM 4567 H14B POPCO 5 -42.573 -15.775 -22.294 1.00 0.00 O2 H

ATOM 4568 H14C POPCO 5 -43.879 -16.598 -21.425 1.00 0.00 O2 H

ATOM 4569 C15 POPCO 5 -44.443 -14.755 -19.607 1.00 0.00 O2 C

ATOM 4570 H15A POPCO 5 -43.949 -15.277 -18.840 1.00 0.00 O2 H

ATOM 4571 H15B POPCO 5 -44.743 -13.802 -19.178 1.00 0.00 O2 H

ATOM 4572 H15C POPCO 5 -45.333 -15.258 -19.830 1.00 0.00 O2 H

ATOM 4573 C11 POPCO 5 -42.754 -12.269 -19.758 1.00 0.00 O2 C

ATOM 4574 H11A POPCO 5 -43.085 -12.565 -18.821 1.00 0.00 O2 H

ATOM 4575 H11B POPCO 5 -41.798 -11.716 -19.532 1.00 0.00 O2 H

ATOM 4576 P POPCO 5 -44.586 -10.281 -19.659 1.00 0.00 O2 P

ATOM 4577 O13 POPCO 5 -45.550 -9.691 -20.545 1.00 0.00 O2 O

ATOM 4578 O14 POPCO 5 -43.659 -9.390 -18.985 1.00 0.00 O2 O

ATOM 4579 O12 POPCO 5 -43.688 -11.349 -20.431 1.00 0.00 O2 O

ATOM 4580 O11 POPCO 5 -45.377 -11.147 -18.563 1.00 0.00 O2 O

ATOM 4581 C1 POPCO 5 -45.071 -11.076 -17.143 1.00 0.00 O2 C

ATOM 4582 HA POPCO 5 -43.950 -11.152 -16.947 1.00 0.00 O2 H

ATOM 4583 HB POPCO 5 -45.204 -10.076 -16.818 1.00 0.00 O2 H

ATOM 4584 C2 POPCO 5 -45.926 -12.004 -16.217 1.00 0.00 O2 C

ATOM 4585 HS POPCO 5 -47.053 -11.953 -16.327 1.00 0.00 O2 H

ATOM 4586 O21 POPCO 5 -45.647 -11.672 -14.852 1.00 0.00 O2 O

ATOM 4587 C21 POPCO 5 -46.178 -12.408 -13.919 1.00 0.00 O2 C

ATOM 4588 O22 POPCO 5 -47.161 -13.118 -14.086 1.00 0.00 O2 O

ATOM 4589 C22 POPCO 5 -45.241 -12.359 -12.673 1.00 0.00 O2 C

ATOM 4590 H2R POPCO 5 -44.216 -12.607 -12.959 1.00 0.00 O2 H

ATOM 4591 H2S POPCO 5 -45.080 -11.407 -12.251 1.00 0.00 O2 H

ATOM 4592 C3 POPCO 5 -45.504 -13.478 -16.459 1.00 0.00 O2 C

ATOM 4593 HX POPCO 5 -45.899 -14.186 -15.749 1.00 0.00 O2 H

ATOM 4594 HY POPCO 5 -46.013 -13.900 -17.367 1.00 0.00 O2 H

ATOM 4595 O31 POPCO 5 -44.073 -13.533 -16.668 1.00 0.00 O2 O

ATOM 4596 C31 POPCO 5 -43.318 -13.867 -15.627 1.00 0.00 O2 C

ATOM 4597 O32 POPCO 5 -43.684 -14.391 -14.548 1.00 0.00 O2 O

ATOM 4598 C32 POPCO 5 -41.886 -13.459 -15.916 1.00 0.00 O2 C

ATOM 4599 H2X POPCO 5 -41.464 -13.767 -16.901 1.00 0.00 O2 H

ATOM 4600 H2Y POPCO 5 -41.944 -12.328 -15.834 1.00 0.00 O2 H

ATOM 4601 C23 POPCO 5 -45.642 -13.369 -11.590 1.00 0.00 O2 C

ATOM 4602 H3R POPCO 5 -46.752 -13.352 -11.360 1.00 0.00 O2 H

ATOM 4603 H3S POPCO 5 -45.394 -14.318 -12.017 1.00 0.00 O2 H

ATOM 4604 C24 POPCO 5 -44.946 -13.192 -10.174 1.00 0.00 O2 C

ATOM 4605 H4R POPCO 5 -43.845 -13.140 -10.293 1.00 0.00 O2 H

ATOM 4606 H4S POPCO 5 -45.323 -12.222 -9.747 1.00 0.00 O2 H

ATOM 4607 C25 POPCO 5 -45.037 -14.389 -9.268 1.00 0.00 O2 C

ATOM 4608 H5R POPCO 5 -46.108 -14.673 -9.101 1.00 0.00 O2 H

ATOM 4609 H5S POPCO 5 -44.477 -15.279 -9.573 1.00 0.00 O2 H

ATOM 4610 C26 POPCO 5 -44.483 -14.097 -7.923 1.00 0.00 O2 C

ATOM 4611 H6R POPCO 5 -43.307 -14.119 -7.970 1.00 0.00 O2 H

ATOM 4612 H6S POPCO 5 -44.833 -13.098 -7.550 1.00 0.00 O2 H

ATOM 4613 C27 POPCO 5 -44.849 -15.243 -6.975 1.00 0.00 O2 C

ATOM 4614 H7R POPCO 5 -45.997 -15.275 -6.863 1.00 0.00 O2 H

ATOM 4615 H7S POPCO 5 -44.558 -16.184 -7.468 1.00 0.00 O2 H

ATOM 4616 C28 POPCO 5 -44.170 -15.146 -5.585 1.00 0.00 O2 C

ATOM 4617 H8R POPCO 5 -43.073 -15.557 -5.770 1.00 0.00 O2 H

ATOM 4618 H8S POPCO 5 -44.082 -14.041 -5.341 1.00 0.00 O2 H

ATOM 4619 C29 POPCO 5 -44.755 -15.939 -4.428 1.00 0.00 O2 C

ATOM 4620 H91 POPCO 5 -44.844 -17.021 -4.531 1.00 0.00 O2 H

ATOM 4621 C210 POPCO 5 -45.258 -15.428 -3.249 1.00 0.00 O2 C

ATOM 4622 H101 POPCO 5 -45.717 -16.097 -2.480 1.00 0.00 O2 H

ATOM 4623 C211 POPCO 5 -45.320 -13.996 -2.899 1.00 0.00 O2 C

ATOM 4624 H11R POPCO 5 -44.719 -13.286 -3.545 1.00 0.00 O2 H

ATOM 4625 H11S POPCO 5 -46.420 -13.690 -2.822 1.00 0.00 O2 H

ATOM 4626 C212 POPCO 5 -44.671 -13.785 -1.442 1.00 0.00 O2 C

ATOM 4627 H12R POPCO 5 -43.584 -14.060 -1.468 1.00 0.00 O2 H

ATOM 4628 H12S POPCO 5 -44.693 -12.719 -1.308 1.00 0.00 O2 H

ATOM 4629 C213 POPCO 5 -45.389 -14.493 -0.278 1.00 0.00 O2 C

ATOM 4630 H13R POPCO 5 -46.496 -14.204 -0.239 1.00 0.00 O2 H

ATOM 4631 H13S POPCO 5 -45.398 -15.619 -0.455 1.00 0.00 O2 H

ATOM 4632 C214 POPCO 5 -44.715 -14.382 1.103 1.00 0.00 O2 C

ATOM 4633 H14R POPCO 5 -43.639 -14.630 1.072 1.00 0.00 O2 H

ATOM 4634 H14S POPCO 5 -44.705 -13.319 1.367 1.00 0.00 O2 H

ATOM 4635 C215 POPCO 5 -45.394 -15.029 2.341 1.00 0.00 O2 C

ATOM 4636 H15R POPCO 5 -46.404 -14.596 2.509 1.00 0.00 O2 H

ATOM 4637 H15S POPCO 5 -45.448 -16.142 2.341 1.00 0.00 O2 H

ATOM 4638 C216 POPCO 5 -44.616 -14.541 3.600 1.00 0.00 O2 C

ATOM 4639 H16R POPCO 5 -43.534 -14.883 3.700 1.00 0.00 O2 H

ATOM 4640 H16S POPCO 5 -44.716 -13.427 3.637 1.00 0.00 O2 H

ATOM 4641 C217 POPCO 5 -45.363 -15.140 4.833 1.00 0.00 O2 C

ATOM 4642 H17R POPCO 5 -46.372 -14.724 4.708 1.00 0.00 O2 H

ATOM 4643 H17S POPCO 5 -45.472 -16.148 4.664 1.00 0.00 O2 H

ATOM 4644 C218 POPCO 5 -44.870 -14.730 6.204 1.00 0.00 O2 C

ATOM 4645 H18R POPCO 5 -45.465 -15.178 7.009 1.00 0.00 O2 H

ATOM 4646 H18S POPCO 5 -43.803 -15.114 6.357 1.00 0.00 O2 H

ATOM 4647 H18T POPCO 5 -44.813 -13.638 6.291 1.00 0.00 O2 H

ATOM 4648 C33 POPCO 5 -40.821 -13.916 -14.879 1.00 0.00 O2 C

ATOM 4649 H3X POPCO 5 -40.819 -15.007 -14.754 1.00 0.00 O2 H

ATOM 4650 H3Y POPCO 5 -39.843 -13.522 -15.275 1.00 0.00 O2 H

ATOM 4651 C34 POPCO 5 -41.059 -13.298 -13.432 1.00 0.00 O2 C

ATOM 4652 H4X POPCO 5 -41.405 -12.251 -13.506 1.00 0.00 O2 H

ATOM 4653 H4Y POPCO 5 -41.875 -13.909 -12.991 1.00 0.00 O2 H

ATOM 4654 C35 POPCO 5 -39.745 -13.240 -12.566 1.00 0.00 O2 C

ATOM 4655 H5X POPCO 5 -39.081 -14.106 -12.775 1.00 0.00 O2 H

ATOM 4656 H5Y POPCO 5 -39.166 -12.315 -12.746 1.00 0.00 O2 H

ATOM 4657 C36 POPCO 5 -39.941 -13.423 -11.051 1.00 0.00 O2 C

ATOM 4658 H6X POPCO 5 -40.353 -12.505 -10.695 1.00 0.00 O2 H

ATOM 4659 H6Y POPCO 5 -40.626 -14.322 -10.848 1.00 0.00 O2 H

ATOM 4660 C37 POPCO 5 -38.681 -13.717 -10.241 1.00 0.00 O2 C

ATOM 4661 H7X POPCO 5 -38.090 -14.666 -10.473 1.00 0.00 O2 H

ATOM 4662 H7Y POPCO 5 -38.049 -12.876 -10.462 1.00 0.00 O2 H

ATOM 4663 C38 POPCO 5 -38.981 -13.863 -8.755 1.00 0.00 O2 C

ATOM 4664 H8X POPCO 5 -38.108 -13.627 -8.133 1.00 0.00 O2 H

ATOM 4665 H8Y POPCO 5 -39.695 -13.075 -8.566 1.00 0.00 O2 H

ATOM 4666 C39 POPCO 5 -39.559 -15.220 -8.292 1.00 0.00 O2 C

ATOM 4667 H9X POPCO 5 -40.124 -15.600 -9.183 1.00 0.00 O2 H

ATOM 4668 H9Y POPCO 5 -38.742 -15.926 -8.011 1.00 0.00 O2 H

ATOM 4669 C310 POPCO 5 -40.581 -14.947 -7.187 1.00 0.00 O2 C

ATOM 4670 H10X POPCO 5 -41.231 -14.150 -7.628 1.00 0.00 O2 H

ATOM 4671 H10Y POPCO 5 -41.203 -15.924 -6.910 1.00 0.00 O2 H

ATOM 4672 C311 POPCO 5 -39.892 -14.343 -5.938 1.00 0.00 O2 C

ATOM 4673 H11X POPCO 5 -39.081 -15.172 -5.819 1.00 0.00 O2 H

ATOM 4674 H11Y POPCO 5 -39.282 -13.437 -6.021 1.00 0.00 O2 H

ATOM 4675 C312 POPCO 5 -40.700 -14.365 -4.642 1.00 0.00 O2 C

ATOM 4676 H12X POPCO 5 -41.644 -13.794 -4.812 1.00 0.00 O2 H

ATOM 4677 H12Y POPCO 5 -40.969 -15.397 -4.393 1.00 0.00 O2 H

ATOM 4678 C313 POPCO 5 -39.984 -13.686 -3.452 1.00 0.00 O2 C

ATOM 4679 H13X POPCO 5 -38.964 -14.158 -3.260 1.00 0.00 O2 H

ATOM 4680 H13Y POPCO 5 -39.972 -12.599 -3.609 1.00 0.00 O2 H

ATOM 4681 C314 POPCO 5 -40.909 -13.729 -2.296 1.00 0.00 O2 C

ATOM 4682 H14X POPCO 5 -41.847 -13.248 -2.680 1.00 0.00 O2 H

ATOM 4683 H14Y POPCO 5 -41.348 -14.687 -1.970 1.00 0.00 O2 H

ATOM 4684 C315 POPCO 5 -40.375 -13.053 -1.015 1.00 0.00 O2 C

ATOM 4685 H15X POPCO 5 -39.514 -13.525 -0.473 1.00 0.00 O2 H

ATOM 4686 H15Y POPCO 5 -39.956 -12.072 -1.365 1.00 0.00 O2 H

ATOM 4687 C316 POPCO 5 -41.504 -12.820 -0.019 1.00 0.00 O2 C

ATOM 4688 H16X POPCO 5 -42.094 -13.803 0.008 1.00 0.00 O2 H

ATOM 4689 H16Y POPCO 5 -41.087 -12.507 1.017 1.00 0.00 O2 H

ATOM 4690 H16Z POPCO 5 -42.221 -12.042 -0.372 1.00 0.00 O2 H

ATOM 4691 N POPCO 6 -42.212 -5.938 -18.954 1.00 0.00 O2 N

ATOM 4692 C12 POPCO 6 -42.108 -6.562 -17.541 1.00 0.00 O2 C

ATOM 4693 H12A POPCO 6 -41.827 -7.618 -17.669 1.00 0.00 O2 H

ATOM 4694 H12B POPCO 6 -43.009 -6.483 -16.968 1.00 0.00 O2 H

ATOM 4695 C13 POPCO 6 -41.196 -6.626 -19.730 1.00 0.00 O2 C

ATOM 4696 H13A POPCO 6 -41.232 -7.720 -19.680 1.00 0.00 O2 H

ATOM 4697 H13B POPCO 6 -40.150 -6.433 -19.386 1.00 0.00 O2 H

ATOM 4698 H13C POPCO 6 -41.226 -6.252 -20.778 1.00 0.00 O2 H

ATOM 4699 C14 POPCO 6 -43.579 -6.242 -19.392 1.00 0.00 O2 C

ATOM 4700 H14A POPCO 6 -44.335 -5.745 -18.847 1.00 0.00 O2 H

ATOM 4701 H14B POPCO 6 -43.784 -7.284 -19.281 1.00 0.00 O2 H

ATOM 4702 H14C POPCO 6 -43.750 -6.116 -20.473 1.00 0.00 O2 H

ATOM 4703 C15 POPCO 6 -42.052 -4.439 -18.947 1.00 0.00 O2 C

ATOM 4704 H15A POPCO 6 -42.777 -3.916 -18.349 1.00 0.00 O2 H

ATOM 4705 H15B POPCO 6 -41.080 -4.236 -18.373 1.00 0.00 O2 H

ATOM 4706 H15C POPCO 6 -41.844 -3.996 -19.922 1.00 0.00 O2 H

ATOM 4707 C11 POPCO 6 -41.011 -5.968 -16.626 1.00 0.00 O2 C

ATOM 4708 H11A POPCO 6 -41.436 -4.926 -16.310 1.00 0.00 O2 H

ATOM 4709 H11B POPCO 6 -40.981 -6.452 -15.588 1.00 0.00 O2 H

ATOM 4710 P POPCO 6 -38.796 -4.832 -17.547 1.00 0.00 O2 P

ATOM 4711 O13 POPCO 6 -39.638 -3.598 -17.419 1.00 0.00 O2 O

ATOM 4712 O14 POPCO 6 -38.375 -5.132 -18.913 1.00 0.00 O2 O

ATOM 4713 O12 POPCO 6 -39.629 -6.071 -17.071 1.00 0.00 O2 O

ATOM 4714 O11 POPCO 6 -37.570 -4.861 -16.530 1.00 0.00 O2 O

ATOM 4715 C1 POPCO 6 -37.693 -5.115 -15.196 1.00 0.00 O2 C

ATOM 4716 HA POPCO 6 -38.741 -4.921 -14.828 1.00 0.00 O2 H

ATOM 4717 HB POPCO 6 -37.474 -6.099 -14.927 1.00 0.00 O2 H

ATOM 4718 C2 POPCO 6 -36.766 -4.199 -14.305 1.00 0.00 O2 C

ATOM 4719 HS POPCO 6 -35.683 -4.271 -14.593 1.00 0.00 O2 H

ATOM 4720 O21 POPCO 6 -37.006 -4.680 -12.931 1.00 0.00 O2 O

ATOM 4721 C21 POPCO 6 -36.124 -4.259 -12.064 1.00 0.00 O2 C

ATOM 4722 O22 POPCO 6 -35.178 -3.557 -12.289 1.00 0.00 O2 O

ATOM 4723 C22 POPCO 6 -36.433 -4.709 -10.672 1.00 0.00 O2 C

ATOM 4724 H2R POPCO 6 -37.485 -4.824 -10.497 1.00 0.00 O2 H

ATOM 4725 H2S POPCO 6 -35.973 -5.768 -10.579 1.00 0.00 O2 H

ATOM 4726 C3 POPCO 6 -37.252 -2.748 -14.353 1.00 0.00 O2 C

ATOM 4727 HX POPCO 6 -36.634 -2.029 -13.854 1.00 0.00 O2 H

ATOM 4728 HY POPCO 6 -37.226 -2.393 -15.443 1.00 0.00 O2 H

ATOM 4729 O31 POPCO 6 -38.635 -2.707 -13.927 1.00 0.00 O2 O

ATOM 4730 C31 POPCO 6 -39.190 -1.607 -13.569 1.00 0.00 O2 C

ATOM 4731 O32 POPCO 6 -38.643 -0.565 -13.332 1.00 0.00 O2 O

ATOM 4732 C32 POPCO 6 -40.688 -1.793 -13.393 1.00 0.00 O2 C

ATOM 4733 H2X POPCO 6 -41.265 -1.354 -14.221 1.00 0.00 O2 H

ATOM 4734 H2Y POPCO 6 -41.063 -2.849 -13.450 1.00 0.00 O2 H

ATOM 4735 C23 POPCO 6 -35.771 -3.812 -9.578 1.00 0.00 O2 C

ATOM 4736 H3R POPCO 6 -34.620 -3.829 -9.534 1.00 0.00 O2 H

ATOM 4737 H3S POPCO 6 -36.058 -2.716 -9.718 1.00 0.00 O2 H

ATOM 4738 C24 POPCO 6 -36.214 -4.228 -8.197 1.00 0.00 O2 C

ATOM 4739 H4R POPCO 6 -37.255 -4.139 -8.130 1.00 0.00 O2 H

ATOM 4740 H4S POPCO 6 -35.927 -5.251 -8.030 1.00 0.00 O2 H

ATOM 4741 C25 POPCO 6 -35.606 -3.382 -7.081 1.00 0.00 O2 C

ATOM 4742 H5R POPCO 6 -34.607 -3.730 -6.739 1.00 0.00 O2 H

ATOM 4743 H5S POPCO 6 -35.592 -2.359 -7.471 1.00 0.00 O2 H

ATOM 4744 C26 POPCO 6 -36.590 -3.418 -5.896 1.00 0.00 O2 C

ATOM 4745 H6R POPCO 6 -37.633 -3.111 -6.112 1.00 0.00 O2 H

ATOM 4746 H6S POPCO 6 -36.632 -4.518 -5.612 1.00 0.00 O2 H

ATOM 4747 C27 POPCO 6 -35.975 -2.670 -4.600 1.00 0.00 O2 C

ATOM 4748 H7R POPCO 6 -35.034 -3.241 -4.316 1.00 0.00 O2 H

ATOM 4749 H7S POPCO 6 -35.636 -1.646 -4.913 1.00 0.00 O2 H

ATOM 4750 C28 POPCO 6 -36.837 -2.578 -3.302 1.00 0.00 O2 C

ATOM 4751 H8R POPCO 6 -37.629 -1.852 -3.678 1.00 0.00 O2 H

ATOM 4752 H8S POPCO 6 -37.266 -3.532 -3.067 1.00 0.00 O2 H

ATOM 4753 C29 POPCO 6 -36.032 -1.799 -2.206 1.00 0.00 O2 C

ATOM 4754 H91 POPCO 6 -35.204 -1.158 -2.543 1.00 0.00 O2 H

ATOM 4755 C210 POPCO 6 -36.321 -1.762 -0.912 1.00 0.00 O2 C

ATOM 4756 H101 POPCO 6 -35.702 -1.147 -0.245 1.00 0.00 O2 H

ATOM 4757 C211 POPCO 6 -37.411 -2.487 -0.264 1.00 0.00 O2 C

ATOM 4758 H11R POPCO 6 -38.282 -2.728 -0.938 1.00 0.00 O2 H

ATOM 4759 H11S POPCO 6 -37.008 -3.441 0.008 1.00 0.00 O2 H

ATOM 4760 C212 POPCO 6 -37.951 -1.859 1.099 1.00 0.00 O2 C

ATOM 4761 H12R POPCO 6 -38.687 -1.071 0.966 1.00 0.00 O2 H

ATOM 4762 H12S POPCO 6 -38.375 -2.736 1.618 1.00 0.00 O2 H

ATOM 4763 C213 POPCO 6 -36.913 -1.353 2.099 1.00 0.00 O2 C

ATOM 4764 H13R POPCO 6 -36.058 -2.076 2.156 1.00 0.00 O2 H

ATOM 4765 H13S POPCO 6 -36.387 -0.461 1.649 1.00 0.00 O2 H

ATOM 4766 C214 POPCO 6 -37.386 -1.158 3.499 1.00 0.00 O2 C

ATOM 4767 H14R POPCO 6 -38.117 -0.306 3.514 1.00 0.00 O2 H

ATOM 4768 H14S POPCO 6 -37.886 -2.118 3.841 1.00 0.00 O2 H

ATOM 4769 C215 POPCO 6 -36.279 -0.677 4.533 1.00 0.00 O2 C

ATOM 4770 H15R POPCO 6 -35.402 -1.252 4.296 1.00 0.00 O2 H

ATOM 4771 H15S POPCO 6 -36.127 0.387 4.223 1.00 0.00 O2 H

ATOM 4772 C216 POPCO 6 -36.759 -0.780 5.947 1.00 0.00 O2 C

ATOM 4773 H16R POPCO 6 -37.796 -0.307 5.937 1.00 0.00 O2 H

ATOM 4774 H16S POPCO 6 -36.783 -1.853 6.238 1.00 0.00 O2 H

ATOM 4775 C217 POPCO 6 -36.067 0.027 7.012 1.00 0.00 O2 C

ATOM 4776 H17R POPCO 6 -35.101 -0.450 7.225 1.00 0.00 O2 H

ATOM 4777 H17S POPCO 6 -35.796 1.068 6.703 1.00 0.00 O2 H

ATOM 4778 C218 POPCO 6 -36.939 0.117 8.285 1.00 0.00 O2 C

ATOM 4779 H18R POPCO 6 -36.423 0.446 9.152 1.00 0.00 O2 H

ATOM 4780 H18S POPCO 6 -37.834 0.779 8.069 1.00 0.00 O2 H

ATOM 4781 H18T POPCO 6 -37.330 -0.891 8.593 1.00 0.00 O2 H

ATOM 4782 C33 POPCO 6 -41.145 -1.262 -11.999 1.00 0.00 O2 C

ATOM 4783 H3X POPCO 6 -40.848 -0.198 -11.932 1.00 0.00 O2 H

ATOM 4784 H3Y POPCO 6 -42.180 -1.550 -11.872 1.00 0.00 O2 H

ATOM 4785 C34 POPCO 6 -40.438 -2.058 -10.819 1.00 0.00 O2 C

ATOM 4786 H4X POPCO 6 -40.177 -3.076 -11.130 1.00 0.00 O2 H

ATOM 4787 H4Y POPCO 6 -39.435 -1.558 -10.595 1.00 0.00 O2 H

ATOM 4788 C35 POPCO 6 -41.335 -2.131 -9.656 1.00 0.00 O2 C

ATOM 4789 H5X POPCO 6 -41.688 -1.189 -9.341 1.00 0.00 O2 H

ATOM 4790 H5Y POPCO 6 -42.235 -2.729 -9.979 1.00 0.00 O2 H

ATOM 4791 C36 POPCO 6 -40.606 -2.636 -8.414 1.00 0.00 O2 C

ATOM 4792 H6X POPCO 6 -40.296 -3.688 -8.615 1.00 0.00 O2 H

ATOM 4793 H6Y POPCO 6 -39.621 -2.075 -8.256 1.00 0.00 O2 H

ATOM 4794 C37 POPCO 6 -41.448 -2.544 -7.102 1.00 0.00 O2 C

ATOM 4795 H7X POPCO 6 -41.693 -1.507 -7.100 1.00 0.00 O2 H

ATOM 4796 H7Y POPCO 6 -42.400 -3.159 -7.151 1.00 0.00 O2 H

ATOM 4797 C38 POPCO 6 -40.703 -2.841 -5.759 1.00 0.00 O2 C

ATOM 4798 H8X POPCO 6 -40.691 -3.890 -5.435 1.00 0.00 O2 H

ATOM 4799 H8Y POPCO 6 -39.588 -2.530 -5.882 1.00 0.00 O2 H

ATOM 4800 C39 POPCO 6 -41.438 -2.062 -4.664 1.00 0.00 O2 C

ATOM 4801 H9X POPCO 6 -41.484 -1.010 -5.041 1.00 0.00 O2 H

ATOM 4802 H9Y POPCO 6 -42.485 -2.502 -4.657 1.00 0.00 O2 H

ATOM 4803 C310 POPCO 6 -40.633 -2.117 -3.369 1.00 0.00 O2 C

ATOM 4804 H10X POPCO 6 -40.401 -3.185 -3.057 1.00 0.00 O2 H

ATOM 4805 H10Y POPCO 6 -39.709 -1.529 -3.606 1.00 0.00 O2 H

ATOM 4806 C311 POPCO 6 -41.462 -1.461 -2.279 1.00 0.00 O2 C

ATOM 4807 H11X POPCO 6 -41.040 -0.432 -2.123 1.00 0.00 O2 H

ATOM 4808 H11Y POPCO 6 -42.579 -1.405 -2.598 1.00 0.00 O2 H

ATOM 4809 C312 POPCO 6 -41.420 -2.220 -0.946 1.00 0.00 O2 C

ATOM 4810 H12X POPCO 6 -41.701 -3.261 -1.137 1.00 0.00 O2 H

ATOM 4811 H12Y POPCO 6 -40.390 -2.198 -0.618 1.00 0.00 O2 H

ATOM 4812 C313 POPCO 6 -42.261 -1.493 0.125 1.00 0.00 O2 C

ATOM 4813 H13X POPCO 6 -41.801 -0.448 0.293 1.00 0.00 O2 H

ATOM 4814 H13Y POPCO 6 -43.313 -1.314 -0.149 1.00 0.00 O2 H

ATOM 4815 C314 POPCO 6 -42.293 -2.332 1.391 1.00 0.00 O2 C

ATOM 4816 H14X POPCO 6 -42.609 -3.399 1.273 1.00 0.00 O2 H

ATOM 4817 H14Y POPCO 6 -41.205 -2.351 1.836 1.00 0.00 O2 H

ATOM 4818 C315 POPCO 6 -43.100 -1.701 2.476 1.00 0.00 O2 C

ATOM 4819 H15X POPCO 6 -43.022 -0.616 2.540 1.00 0.00 O2 H

ATOM 4820 H15Y POPCO 6 -44.224 -1.998 2.349 1.00 0.00 O2 H

ATOM 4821 C316 POPCO 6 -42.738 -2.289 3.860 1.00 0.00 O2 C

ATOM 4822 H16X POPCO 6 -41.785 -1.948 4.158 1.00 0.00 O2 H

ATOM 4823 H16Y POPCO 6 -43.539 -1.913 4.601 1.00 0.00 O2 H

ATOM 4824 H16Z POPCO 6 -42.773 -3.442 3.870 1.00 0.00 O2 H

ATOM 4825 N POPCO 7 -32.552 -42.618 -20.621 1.00 0.00 O2 N

ATOM 4826 C12 POPCO 7 -34.051 -42.255 -20.816 1.00 0.00 O2 C

ATOM 4827 H12A POPCO 7 -34.535 -42.221 -19.851 1.00 0.00 O2 H

ATOM 4828 H12B POPCO 7 -34.502 -42.955 -21.502 1.00 0.00 O2 H

ATOM 4829 C13 POPCO 7 -31.944 -41.672 -19.675 1.00 0.00 O2 C

ATOM 4830 H13A POPCO 7 -32.330 -41.834 -18.646 1.00 0.00 O2 H

ATOM 4831 H13B POPCO 7 -32.141 -40.650 -19.955 1.00 0.00 O2 H

ATOM 4832 H13C POPCO 7 -30.867 -41.633 -19.794 1.00 0.00 O2 H

ATOM 4833 C14 POPCO 7 -32.454 -43.962 -20.086 1.00 0.00 O2 C

ATOM 4834 H14A POPCO 7 -32.990 -44.637 -20.755 1.00 0.00 O2 H

ATOM 4835 H14B POPCO 7 -32.975 -44.104 -19.112 1.00 0.00 O2 H

ATOM 4836 H14C POPCO 7 -31.446 -44.288 -20.083 1.00 0.00 O2 H

ATOM 4837 C15 POPCO 7 -31.848 -42.486 -21.881 1.00 0.00 O2 C

ATOM 4838 H15A POPCO 7 -32.185 -43.308 -22.496 1.00 0.00 O2 H

ATOM 4839 H15B POPCO 7 -31.970 -41.523 -22.402 1.00 0.00 O2 H

ATOM 4840 H15C POPCO 7 -30.749 -42.667 -21.735 1.00 0.00 O2 H

ATOM 4841 C11 POPCO 7 -34.476 -40.914 -21.427 1.00 0.00 O2 C

ATOM 4842 H11A POPCO 7 -33.899 -40.548 -22.274 1.00 0.00 O2 H

ATOM 4843 H11B POPCO 7 -35.449 -41.049 -21.855 1.00 0.00 O2 H

ATOM 4844 P POPCO 7 -33.965 -38.621 -20.339 1.00 0.00 O2 P

ATOM 4845 O13 POPCO 7 -34.705 -37.669 -21.186 1.00 0.00 O2 O

ATOM 4846 O14 POPCO 7 -32.506 -38.866 -20.659 1.00 0.00 O2 O

ATOM 4847 O12 POPCO 7 -34.707 -39.941 -20.377 1.00 0.00 O2 O

ATOM 4848 O11 POPCO 7 -34.069 -38.203 -18.862 1.00 0.00 O2 O

ATOM 4849 C1 POPCO 7 -33.673 -38.969 -17.727 1.00 0.00 O2 C

ATOM 4850 HA POPCO 7 -33.958 -40.048 -17.773 1.00 0.00 O2 H

ATOM 4851 HB POPCO 7 -32.587 -38.904 -17.800 1.00 0.00 O2 H

ATOM 4852 C2 POPCO 7 -34.346 -38.304 -16.466 1.00 0.00 O2 C

ATOM 4853 HS POPCO 7 -34.151 -37.194 -16.474 1.00 0.00 O2 H

ATOM 4854 O21 POPCO 7 -33.900 -38.897 -15.208 1.00 0.00 O2 O

ATOM 4855 C21 POPCO 7 -34.140 -38.220 -14.103 1.00 0.00 O2 C

ATOM 4856 O22 POPCO 7 -34.580 -37.101 -14.036 1.00 0.00 O2 O

ATOM 4857 C22 POPCO 7 -33.839 -39.065 -12.928 1.00 0.00 O2 C

ATOM 4858 H2R POPCO 7 -34.131 -40.132 -12.957 1.00 0.00 O2 H

ATOM 4859 H2S POPCO 7 -32.750 -39.062 -12.821 1.00 0.00 O2 H

ATOM 4860 C3 POPCO 7 -35.946 -38.383 -16.469 1.00 0.00 O2 C

ATOM 4861 HX POPCO 7 -36.362 -38.214 -15.426 1.00 0.00 O2 H

ATOM 4862 HY POPCO 7 -36.297 -37.555 -17.135 1.00 0.00 O2 H

ATOM 4863 O31 POPCO 7 -36.382 -39.672 -16.891 1.00 0.00 O2 O

ATOM 4864 C31 POPCO 7 -37.744 -39.907 -16.981 1.00 0.00 O2 C

ATOM 4865 O32 POPCO 7 -38.579 -39.048 -17.028 1.00 0.00 O2 O

ATOM 4866 C32 POPCO 7 -38.049 -41.392 -17.038 1.00 0.00 O2 C

ATOM 4867 H2X POPCO 7 -38.874 -41.576 -17.825 1.00 0.00 O2 H

ATOM 4868 H2Y POPCO 7 -37.073 -41.909 -17.224 1.00 0.00 O2 H

ATOM 4869 C23 POPCO 7 -34.417 -38.486 -11.569 1.00 0.00 O2 C

ATOM 4870 H3R POPCO 7 -33.828 -37.541 -11.240 1.00 0.00 O2 H

ATOM 4871 H3S POPCO 7 -35.436 -38.250 -11.611 1.00 0.00 O2 H

ATOM 4872 C24 POPCO 7 -34.211 -39.521 -10.434 1.00 0.00 O2 C

ATOM 4873 H4R POPCO 7 -34.537 -40.568 -10.589 1.00 0.00 O2 H

ATOM 4874 H4S POPCO 7 -33.155 -39.825 -10.447 1.00 0.00 O2 H

ATOM 4875 C25 POPCO 7 -34.755 -38.982 -9.051 1.00 0.00 O2 C

ATOM 4876 H5R POPCO 7 -34.526 -37.948 -9.025 1.00 0.00 O2 H

ATOM 4877 H5S POPCO 7 -35.939 -39.047 -9.157 1.00 0.00 O2 H

ATOM 4878 C26 POPCO 7 -34.402 -39.708 -7.713 1.00 0.00 O2 C

ATOM 4879 H6R POPCO 7 -34.589 -40.793 -7.750 1.00 0.00 O2 H

ATOM 4880 H6S POPCO 7 -33.269 -39.686 -7.602 1.00 0.00 O2 H

ATOM 4881 C27 POPCO 7 -35.216 -39.044 -6.563 1.00 0.00 O2 C

ATOM 4882 H7R POPCO 7 -35.248 -37.937 -6.779 1.00 0.00 O2 H

ATOM 4883 H7S POPCO 7 -36.294 -39.313 -6.770 1.00 0.00 O2 H

ATOM 4884 C28 POPCO 7 -34.685 -39.279 -5.160 1.00 0.00 O2 C

ATOM 4885 H8R POPCO 7 -34.466 -40.386 -5.064 1.00 0.00 O2 H

ATOM 4886 H8S POPCO 7 -33.659 -38.830 -5.199 1.00 0.00 O2 H

ATOM 4887 C29 POPCO 7 -35.492 -38.688 -4.010 1.00 0.00 O2 C

ATOM 4888 H91 POPCO 7 -35.753 -37.664 -4.101 1.00 0.00 O2 H

ATOM 4889 C210 POPCO 7 -35.736 -39.189 -2.805 1.00 0.00 O2 C

ATOM 4890 H101 POPCO 7 -36.356 -38.643 -2.103 1.00 0.00 O2 H

ATOM 4891 C211 POPCO 7 -35.415 -40.593 -2.303 1.00 0.00 O2 C

ATOM 4892 H11R POPCO 7 -36.379 -41.084 -2.477 1.00 0.00 O2 H

ATOM 4893 H11S POPCO 7 -34.529 -41.003 -2.823 1.00 0.00 O2 H

ATOM 4894 C212 POPCO 7 -35.195 -40.629 -0.756 1.00 0.00 O2 C

ATOM 4895 H12R POPCO 7 -35.002 -41.701 -0.389 1.00 0.00 O2 H

ATOM 4896 H12S POPCO 7 -34.274 -40.107 -0.556 1.00 0.00 O2 H

ATOM 4897 C213 POPCO 7 -36.311 -40.052 0.187 1.00 0.00 O2 C

ATOM 4898 H13R POPCO 7 -36.509 -39.027 -0.110 1.00 0.00 O2 H

ATOM 4899 H13S POPCO 7 -37.256 -40.639 0.063 1.00 0.00 O2 H

ATOM 4900 C214 POPCO 7 -35.934 -40.251 1.658 1.00 0.00 O2 C

ATOM 4901 H14R POPCO 7 -35.921 -41.332 1.945 1.00 0.00 O2 H

ATOM 4902 H14S POPCO 7 -34.941 -39.855 1.823 1.00 0.00 O2 H

ATOM 4903 C215 POPCO 7 -36.967 -39.545 2.541 1.00 0.00 O2 C

ATOM 4904 H15R POPCO 7 -36.921 -38.427 2.388 1.00 0.00 O2 H

ATOM 4905 H15S POPCO 7 -37.972 -39.990 2.251 1.00 0.00 O2 H

ATOM 4906 C216 POPCO 7 -36.675 -39.860 4.044 1.00 0.00 O2 C

ATOM 4907 H16R POPCO 7 -36.663 -40.939 4.341 1.00 0.00 O2 H

ATOM 4908 H16S POPCO 7 -35.613 -39.559 4.285 1.00 0.00 O2 H

ATOM 4909 C217 POPCO 7 -37.766 -39.232 4.931 1.00 0.00 O2 C

ATOM 4910 H17R POPCO 7 -37.841 -38.135 4.784 1.00 0.00 O2 H

ATOM 4911 H17S POPCO 7 -38.805 -39.569 4.621 1.00 0.00 O2 H

ATOM 4912 C218 POPCO 7 -37.533 -39.606 6.405 1.00 0.00 O2 C

ATOM 4913 H18R POPCO 7 -38.479 -39.631 6.981 1.00 0.00 O2 H

ATOM 4914 H18S POPCO 7 -37.055 -40.582 6.544 1.00 0.00 O2 H

ATOM 4915 H18T POPCO 7 -36.834 -38.865 6.847 1.00 0.00 O2 H

ATOM 4916 C33 POPCO 7 -38.610 -41.830 -15.699 1.00 0.00 O2 C

ATOM 4917 H3X POPCO 7 -39.680 -41.616 -15.607 1.00 0.00 O2 H

ATOM 4918 H3Y POPCO 7 -38.820 -42.937 -15.765 1.00 0.00 O2 H

ATOM 4919 C34 POPCO 7 -37.821 -41.435 -14.433 1.00 0.00 O2 C

ATOM 4920 H4X POPCO 7 -36.825 -41.887 -14.385 1.00 0.00 O2 H

ATOM 4921 H4Y POPCO 7 -37.641 -40.326 -14.385 1.00 0.00 O2 H

ATOM 4922 C35 POPCO 7 -38.553 -41.743 -13.144 1.00 0.00 O2 C

ATOM 4923 H5X POPCO 7 -39.334 -41.008 -12.973 1.00 0.00 O2 H

ATOM 4924 H5Y POPCO 7 -38.899 -42.820 -13.267 1.00 0.00 O2 H

ATOM 4925 C36 POPCO 7 -37.601 -41.614 -11.946 1.00 0.00 O2 C

ATOM 4926 H6X POPCO 7 -36.692 -42.190 -12.156 1.00 0.00 O2 H

ATOM 4927 H6Y POPCO 7 -37.303 -40.513 -11.874 1.00 0.00 O2 H

ATOM 4928 C37 POPCO 7 -38.242 -42.057 -10.629 1.00 0.00 O2 C

ATOM 4929 H7X POPCO 7 -39.072 -41.372 -10.456 1.00 0.00 O2 H

ATOM 4930 H7Y POPCO 7 -38.655 -43.133 -10.738 1.00 0.00 O2 H

ATOM 4931 C38 POPCO 7 -37.336 -41.995 -9.444 1.00 0.00 O2 C

ATOM 4932 H8X POPCO 7 -36.492 -42.692 -9.514 1.00 0.00 O2 H

ATOM 4933 H8Y POPCO 7 -36.904 -40.972 -9.232 1.00 0.00 O2 H

ATOM 4934 C39 POPCO 7 -38.089 -42.406 -8.175 1.00 0.00 O2 C

ATOM 4935 H9X POPCO 7 -38.964 -41.662 -8.106 1.00 0.00 O2 H

ATOM 4936 H9Y POPCO 7 -38.517 -43.441 -8.397 1.00 0.00 O2 H

ATOM 4937 C310 POPCO 7 -37.221 -42.379 -6.897 1.00 0.00 O2 C

ATOM 4938 H10X POPCO 7 -36.266 -42.832 -7.073 1.00 0.00 O2 H

ATOM 4939 H10Y POPCO 7 -36.961 -41.325 -6.599 1.00 0.00 O2 H

ATOM 4940 C311 POPCO 7 -38.006 -43.003 -5.694 1.00 0.00 O2 C

ATOM 4941 H11X POPCO 7 -38.669 -42.150 -5.333 1.00 0.00 O2 H

ATOM 4942 H11Y POPCO 7 -38.757 -43.788 -6.070 1.00 0.00 O2 H

ATOM 4943 C312 POPCO 7 -37.209 -43.454 -4.434 1.00 0.00 O2 C

ATOM 4944 H12X POPCO 7 -36.646 -44.391 -4.575 1.00 0.00 O2 H

ATOM 4945 H12Y POPCO 7 -36.399 -42.798 -4.138 1.00 0.00 O2 H

ATOM 4946 C313 POPCO 7 -38.085 -43.757 -3.267 1.00 0.00 O2 C

ATOM 4947 H13X POPCO 7 -38.918 -42.997 -3.203 1.00 0.00 O2 H

ATOM 4948 H13Y POPCO 7 -38.614 -44.729 -3.411 1.00 0.00 O2 H

ATOM 4949 C314 POPCO 7 -37.326 -43.942 -1.940 1.00 0.00 O2 C

ATOM 4950 H14X POPCO 7 -36.722 -44.842 -2.258 1.00 0.00 O2 H

ATOM 4951 H14Y POPCO 7 -36.646 -43.110 -1.799 1.00 0.00 O2 H

ATOM 4952 C315 POPCO 7 -38.155 -44.133 -0.625 1.00 0.00 O2 C

ATOM 4953 H15X POPCO 7 -38.919 -43.306 -0.512 1.00 0.00 O2 H

ATOM 4954 H15Y POPCO 7 -38.825 -45.010 -0.736 1.00 0.00 O2 H

ATOM 4955 C316 POPCO 7 -37.240 -44.095 0.556 1.00 0.00 O2 C

ATOM 4956 H16X POPCO 7 -37.122 -43.118 1.048 1.00 0.00 O2 H

ATOM 4957 H16Y POPCO 7 -37.523 -44.808 1.353 1.00 0.00 O2 H

ATOM 4958 H16Z POPCO 7 -36.261 -44.449 0.210 1.00 0.00 O2 H

ATOM 4959 N POPCO 8 -31.780 -35.056 -20.414 1.00 0.00 O2 N

ATOM 4960 C12 POPCO 8 -31.841 -35.878 -19.099 1.00 0.00 O2 C

ATOM 4961 H12A POPCO 8 -32.313 -36.827 -19.289 1.00 0.00 O2 H

ATOM 4962 H12B POPCO 8 -32.588 -35.356 -18.524 1.00 0.00 O2 H

ATOM 4963 C13 POPCO 8 -30.921 -35.655 -21.375 1.00 0.00 O2 C

ATOM 4964 H13A POPCO 8 -31.046 -36.733 -21.434 1.00 0.00 O2 H

ATOM 4965 H13B POPCO 8 -29.907 -35.570 -21.004 1.00 0.00 O2 H

ATOM 4966 H13C POPCO 8 -31.063 -35.259 -22.377 1.00 0.00 O2 H

ATOM 4967 C14 POPCO 8 -33.153 -35.013 -21.076 1.00 0.00 O2 C

ATOM 4968 H14A POPCO 8 -33.786 -34.382 -20.470 1.00 0.00 O2 H

ATOM 4969 H14B POPCO 8 -33.617 -35.888 -21.137 1.00 0.00 O2 H

ATOM 4970 H14C POPCO 8 -33.158 -34.483 -22.064 1.00 0.00 O2 H

ATOM 4971 C15 POPCO 8 -31.298 -33.676 -20.081 1.00 0.00 O2 C

ATOM 4972 H15A POPCO 8 -32.083 -33.147 -19.616 1.00 0.00 O2 H

ATOM 4973 H15B POPCO 8 -30.392 -33.618 -19.660 1.00 0.00 O2 H

ATOM 4974 H15C POPCO 8 -30.994 -33.128 -20.974 1.00 0.00 O2 H

ATOM 4975 C11 POPCO 8 -30.521 -36.076 -18.273 1.00 0.00 O2 C

ATOM 4976 H11A POPCO 8 -30.081 -35.059 -17.932 1.00 0.00 O2 H

ATOM 4977 H11B POPCO 8 -30.745 -36.736 -17.367 1.00 0.00 O2 H

ATOM 4978 P POPCO 8 -27.999 -36.798 -19.038 1.00 0.00 O2 P

ATOM 4979 O13 POPCO 8 -27.444 -36.253 -20.268 1.00 0.00 O2 O

ATOM 4980 O14 POPCO 8 -27.585 -38.206 -18.644 1.00 0.00 O2 O

ATOM 4981 O12 POPCO 8 -29.556 -36.811 -19.171 1.00 0.00 O2 O

ATOM 4982 O11 POPCO 8 -27.789 -35.703 -17.915 1.00 0.00 O2 O

ATOM 4983 C1 POPCO 8 -27.686 -36.052 -16.556 1.00 0.00 O2 C

ATOM 4984 HA POPCO 8 -28.591 -36.348 -16.084 1.00 0.00 O2 H

ATOM 4985 HB POPCO 8 -26.866 -36.810 -16.371 1.00 0.00 O2 H

ATOM 4986 C2 POPCO 8 -27.241 -34.795 -15.721 1.00 0.00 O2 C

ATOM 4987 HS POPCO 8 -26.106 -34.772 -15.826 1.00 0.00 O2 H

ATOM 4988 O21 POPCO 8 -27.605 -35.003 -14.327 1.00 0.00 O2 O

ATOM 4989 C21 POPCO 8 -26.772 -34.587 -13.351 1.00 0.00 O2 C

ATOM 4990 O22 POPCO 8 -25.584 -34.713 -13.341 1.00 0.00 O2 O

ATOM 4991 C22 POPCO 8 -27.582 -33.951 -12.241 1.00 0.00 O2 C

ATOM 4992 H2R POPCO 8 -27.694 -32.845 -12.586 1.00 0.00 O2 H

ATOM 4993 H2S POPCO 8 -28.579 -34.489 -12.265 1.00 0.00 O2 H

ATOM 4994 C3 POPCO 8 -27.759 -33.380 -16.267 1.00 0.00 O2 C

ATOM 4995 HX POPCO 8 -27.323 -32.597 -15.713 1.00 0.00 O2 H

ATOM 4996 HY POPCO 8 -27.643 -33.328 -17.313 1.00 0.00 O2 H

ATOM 4997 O31 POPCO 8 -29.187 -33.352 -16.016 1.00 0.00 O2 O

ATOM 4998 C31 POPCO 8 -29.951 -32.340 -16.511 1.00 0.00 O2 C

ATOM 4999 O32 POPCO 8 -29.591 -31.502 -17.292 1.00 0.00 O2 O

ATOM 5000 C32 POPCO 8 -31.354 -32.396 -15.941 1.00 0.00 O2 C

ATOM 5001 H2X POPCO 8 -32.076 -32.335 -16.814 1.00 0.00 O2 H

ATOM 5002 H2Y POPCO 8 -31.529 -33.458 -15.556 1.00 0.00 O2 H

ATOM 5003 C23 POPCO 8 -26.879 -34.130 -10.896 1.00 0.00 O2 C

ATOM 5004 H3R POPCO 8 -26.758 -35.241 -10.903 1.00 0.00 O2 H

ATOM 5005 H3S POPCO 8 -25.898 -33.769 -10.966 1.00 0.00 O2 H

ATOM 5006 C24 POPCO 8 -27.657 -33.596 -9.747 1.00 0.00 O2 C

ATOM 5007 H4R POPCO 8 -27.939 -32.510 -9.862 1.00 0.00 O2 H

ATOM 5008 H4S POPCO 8 -28.613 -34.151 -9.638 1.00 0.00 O2 H

ATOM 5009 C25 POPCO 8 -26.806 -33.631 -8.510 1.00 0.00 O2 C

ATOM 5010 H5R POPCO 8 -26.384 -34.654 -8.309 1.00 0.00 O2 H

ATOM 5011 H5S POPCO 8 -25.977 -32.964 -8.665 1.00 0.00 O2 H

ATOM 5012 C26 POPCO 8 -27.687 -33.258 -7.354 1.00 0.00 O2 C

ATOM 5013 H6R POPCO 8 -27.846 -32.141 -7.207 1.00 0.00 O2 H

ATOM 5014 H6S POPCO 8 -28.715 -33.737 -7.432 1.00 0.00 O2 H

ATOM 5015 C27 POPCO 8 -27.194 -33.791 -6.000 1.00 0.00 O2 C

ATOM 5016 H7R POPCO 8 -26.950 -34.846 -6.070 1.00 0.00 O2 H

ATOM 5017 H7S POPCO 8 -26.313 -33.212 -5.723 1.00 0.00 O2 H

ATOM 5018 C28 POPCO 8 -28.224 -33.416 -4.859 1.00 0.00 O2 C

ATOM 5019 H8R POPCO 8 -28.025 -32.380 -4.629 1.00 0.00 O2 H

ATOM 5020 H8S POPCO 8 -29.254 -33.479 -5.307 1.00 0.00 O2 H

ATOM 5021 C29 POPCO 8 -28.169 -34.247 -3.635 1.00 0.00 O2 C

ATOM 5022 H91 POPCO 8 -28.583 -35.250 -3.645 1.00 0.00 O2 H

ATOM 5023 C210 POPCO 8 -27.676 -33.888 -2.433 1.00 0.00 O2 C

ATOM 5024 H101 POPCO 8 -27.731 -34.633 -1.620 1.00 0.00 O2 H

ATOM 5025 C211 POPCO 8 -27.011 -32.538 -2.030 1.00 0.00 O2 C

ATOM 5026 H11R POPCO 8 -25.927 -32.665 -1.609 1.00 0.00 O2 H

ATOM 5027 H11S POPCO 8 -26.960 -31.840 -2.884 1.00 0.00 O2 H

ATOM 5028 C212 POPCO 8 -27.769 -31.717 -0.944 1.00 0.00 O2 C

ATOM 5029 H12R POPCO 8 -27.322 -30.732 -0.971 1.00 0.00 O2 H

ATOM 5030 H12S POPCO 8 -28.876 -31.746 -1.277 1.00 0.00 O2 H

ATOM 5031 C213 POPCO 8 -27.643 -32.507 0.361 1.00 0.00 O2 C

ATOM 5032 H13R POPCO 8 -28.330 -33.416 0.282 1.00 0.00 O2 H

ATOM 5033 H13S POPCO 8 -26.581 -32.823 0.531 1.00 0.00 O2 H

ATOM 5034 C214 POPCO 8 -28.069 -31.695 1.595 1.00 0.00 O2 C

ATOM 5035 H14R POPCO 8 -27.422 -30.862 1.556 1.00 0.00 O2 H

ATOM 5036 H14S POPCO 8 -29.190 -31.401 1.519 1.00 0.00 O2 H

ATOM 5037 C215 POPCO 8 -27.845 -32.582 2.838 1.00 0.00 O2 C

ATOM 5038 H15R POPCO 8 -28.276 -33.561 2.637 1.00 0.00 O2 H

ATOM 5039 H15S POPCO 8 -26.769 -32.750 2.865 1.00 0.00 O2 H

ATOM 5040 C216 POPCO 8 -28.558 -32.010 4.106 1.00 0.00 O2 C

ATOM 5041 H16R POPCO 8 -28.215 -31.018 4.319 1.00 0.00 O2 H

ATOM 5042 H16S POPCO 8 -29.600 -31.963 3.965 1.00 0.00 O2 H

ATOM 5043 C217 POPCO 8 -28.303 -32.807 5.342 1.00 0.00 O2 C

ATOM 5044 H17R POPCO 8 -28.783 -33.757 5.301 1.00 0.00 O2 H

ATOM 5045 H17S POPCO 8 -27.242 -33.178 5.472 1.00 0.00 O2 H

ATOM 5046 C218 POPCO 8 -28.787 -32.137 6.663 1.00 0.00 O2 C

ATOM 5047 H18R POPCO 8 -28.806 -32.953 7.456 1.00 0.00 O2 H

ATOM 5048 H18S POPCO 8 -28.006 -31.512 7.064 1.00 0.00 O2 H

ATOM 5049 H18T POPCO 8 -29.790 -31.640 6.506 1.00 0.00 O2 H

ATOM 5050 C33 POPCO 8 -31.504 -31.369 -14.765 1.00 0.00 O2 C

ATOM 5051 H3X POPCO 8 -31.503 -30.331 -15.128 1.00 0.00 O2 H

ATOM 5052 H3Y POPCO 8 -32.495 -31.510 -14.336 1.00 0.00 O2 H

ATOM 5053 C34 POPCO 8 -30.541 -31.456 -13.561 1.00 0.00 O2 C

ATOM 5054 H4X POPCO 8 -30.429 -32.412 -13.113 1.00 0.00 O2 H

ATOM 5055 H4Y POPCO 8 -29.533 -31.219 -13.952 1.00 0.00 O2 H

ATOM 5056 C35 POPCO 8 -30.972 -30.406 -12.529 1.00 0.00 O2 C

ATOM 5057 H5X POPCO 8 -30.798 -29.378 -12.788 1.00 0.00 O2 H

ATOM 5058 H5Y POPCO 8 -32.041 -30.416 -12.309 1.00 0.00 O2 H

ATOM 5059 C36 POPCO 8 -30.399 -30.656 -11.071 1.00 0.00 O2 C

ATOM 5060 H6X POPCO 8 -30.288 -31.793 -10.873 1.00 0.00 O2 H

ATOM 5061 H6Y POPCO 8 -29.428 -30.210 -11.021 1.00 0.00 O2 H

ATOM 5062 C37 POPCO 8 -31.320 -30.001 -10.075 1.00 0.00 O2 C

ATOM 5063 H7X POPCO 8 -31.505 -28.954 -10.414 1.00 0.00 O2 H

ATOM 5064 H7Y POPCO 8 -32.358 -30.513 -10.017 1.00 0.00 O2 H

ATOM 5065 C38 POPCO 8 -30.755 -29.957 -8.685 1.00 0.00 O2 C

ATOM 5066 H8X POPCO 8 -30.274 -30.945 -8.595 1.00 0.00 O2 H

ATOM 5067 H8Y POPCO 8 -30.085 -29.065 -8.493 1.00 0.00 O2 H

ATOM 5068 C39 POPCO 8 -31.757 -29.961 -7.529 1.00 0.00 O2 C

ATOM 5069 H9X POPCO 8 -32.480 -29.162 -7.599 1.00 0.00 O2 H

ATOM 5070 H9Y POPCO 8 -32.474 -30.833 -7.616 1.00 0.00 O2 H

ATOM 5071 C310 POPCO 8 -31.094 -30.088 -6.157 1.00 0.00 O2 C

ATOM 5072 H10X POPCO 8 -30.451 -30.989 -6.194 1.00 0.00 O2 H

ATOM 5073 H10Y POPCO 8 -30.378 -29.213 -6.136 1.00 0.00 O2 H

ATOM 5074 C311 POPCO 8 -32.049 -30.228 -4.992 1.00 0.00 O2 C

ATOM 5075 H11X POPCO 8 -32.663 -29.329 -4.948 1.00 0.00 O2 H

ATOM 5076 H11Y POPCO 8 -32.752 -31.070 -5.240 1.00 0.00 O2 H

ATOM 5077 C312 POPCO 8 -31.370 -30.617 -3.689 1.00 0.00 O2 C

ATOM 5078 H12X POPCO 8 -30.892 -31.521 -3.928 1.00 0.00 O2 H

ATOM 5079 H12Y POPCO 8 -30.636 -29.950 -3.344 1.00 0.00 O2 H

ATOM 5080 C313 POPCO 8 -32.402 -30.849 -2.540 1.00 0.00 O2 C

ATOM 5081 H13X POPCO 8 -32.866 -29.897 -2.303 1.00 0.00 O2 H

ATOM 5082 H13Y POPCO 8 -33.154 -31.415 -2.955 1.00 0.00 O2 H

ATOM 5083 C314 POPCO 8 -31.594 -31.390 -1.221 1.00 0.00 O2 C

ATOM 5084 H14X POPCO 8 -30.843 -32.110 -1.646 1.00 0.00 O2 H

ATOM 5085 H14Y POPCO 8 -31.081 -30.526 -0.760 1.00 0.00 O2 H

ATOM 5086 C315 POPCO 8 -32.431 -32.193 -0.202 1.00 0.00 O2 C

ATOM 5087 H15X POPCO 8 -33.234 -31.528 0.071 1.00 0.00 O2 H

ATOM 5088 H15Y POPCO 8 -32.835 -33.086 -0.772 1.00 0.00 O2 H

ATOM 5089 C316 POPCO 8 -31.647 -32.733 1.033 1.00 0.00 O2 C

ATOM 5090 H16X POPCO 8 -31.112 -31.984 1.665 1.00 0.00 O2 H

ATOM 5091 H16Y POPCO 8 -32.227 -33.396 1.699 1.00 0.00 O2 H

ATOM 5092 H16Z POPCO 8 -30.827 -33.344 0.617 1.00 0.00 O2 H

ATOM 5093 N POPCO 9 -34.343 -28.275 -18.671 1.00 0.00 O2 N

ATOM 5094 C12 POPCO 9 -33.619 -27.001 -19.031 1.00 0.00 O2 C

ATOM 5095 H12A POPCO 9 -33.705 -26.871 -20.064 1.00 0.00 O2 H

ATOM 5096 H12B POPCO 9 -32.556 -27.143 -18.682 1.00 0.00 O2 H

ATOM 5097 C13 POPCO 9 -35.636 -28.601 -19.415 1.00 0.00 O2 C

ATOM 5098 H13A POPCO 9 -35.511 -28.558 -20.497 1.00 0.00 O2 H

ATOM 5099 H13B POPCO 9 -36.397 -27.905 -19.151 1.00 0.00 O2 H

ATOM 5100 H13C POPCO 9 -36.039 -29.564 -19.164 1.00 0.00 O2 H

ATOM 5101 C14 POPCO 9 -33.388 -29.406 -19.013 1.00 0.00 O2 C

ATOM 5102 H14A POPCO 9 -32.347 -29.176 -18.671 1.00 0.00 O2 H

ATOM 5103 H14B POPCO 9 -33.326 -29.661 -20.068 1.00 0.00 O2 H

ATOM 5104 H14C POPCO 9 -33.764 -30.267 -18.581 1.00 0.00 O2 H

ATOM 5105 C15 POPCO 9 -34.615 -28.415 -17.183 1.00 0.00 O2 C

ATOM 5106 H15A POPCO 9 -33.777 -28.080 -16.591 1.00 0.00 O2 H

ATOM 5107 H15B POPCO 9 -35.448 -27.790 -16.984 1.00 0.00 O2 H

ATOM 5108 H15C POPCO 9 -34.848 -29.419 -16.896 1.00 0.00 O2 H

ATOM 5109 C11 POPCO 9 -34.108 -25.652 -18.306 1.00 0.00 O2 C

ATOM 5110 H11A POPCO 9 -33.924 -25.799 -17.219 1.00 0.00 O2 H

ATOM 5111 H11B POPCO 9 -33.449 -24.834 -18.598 1.00 0.00 O2 H

ATOM 5112 P POPCO 9 -36.751 -25.453 -17.646 1.00 0.00 O2 P

ATOM 5113 O13 POPCO 9 -37.618 -26.524 -18.201 1.00 0.00 O2 O

ATOM 5114 O14 POPCO 9 -37.395 -24.148 -17.358 1.00 0.00 O2 O

ATOM 5115 O12 POPCO 9 -35.538 -25.331 -18.608 1.00 0.00 O2 O

ATOM 5116 O11 POPCO 9 -36.188 -25.986 -16.314 1.00 0.00 O2 O

ATOM 5117 C1 POPCO 9 -36.296 -25.272 -15.039 1.00 0.00 O2 C

ATOM 5118 HA POPCO 9 -35.487 -24.510 -15.054 1.00 0.00 O2 H

ATOM 5119 HB POPCO 9 -37.266 -24.729 -14.974 1.00 0.00 O2 H

ATOM 5120 C2 POPCO 9 -36.246 -26.231 -13.755 1.00 0.00 O2 C

ATOM 5121 HS POPCO 9 -37.096 -26.929 -13.886 1.00 0.00 O2 H

ATOM 5122 O21 POPCO 9 -36.457 -25.451 -12.587 1.00 0.00 O2 O

ATOM 5123 C21 POPCO 9 -36.685 -26.175 -11.477 1.00 0.00 O2 C

ATOM 5124 O22 POPCO 9 -37.311 -27.198 -11.550 1.00 0.00 O2 O

ATOM 5125 C22 POPCO 9 -36.133 -25.541 -10.180 1.00 0.00 O2 C

ATOM 5126 H2R POPCO 9 -35.053 -25.276 -10.249 1.00 0.00 O2 H

ATOM 5127 H2S POPCO 9 -36.789 -24.681 -9.895 1.00 0.00 O2 H

ATOM 5128 C3 POPCO 9 -34.964 -27.131 -13.733 1.00 0.00 O2 C

ATOM 5129 HX POPCO 9 -34.941 -27.948 -12.859 1.00 0.00 O2 H

ATOM 5130 HY POPCO 9 -34.976 -27.737 -14.634 1.00 0.00 O2 H

ATOM 5131 O31 POPCO 9 -33.798 -26.327 -13.634 1.00 0.00 O2 O

ATOM 5132 C31 POPCO 9 -32.715 -26.709 -14.317 1.00 0.00 O2 C

ATOM 5133 O32 POPCO 9 -32.601 -27.788 -14.868 1.00 0.00 O2 O

ATOM 5134 C32 POPCO 9 -31.625 -25.639 -14.387 1.00 0.00 O2 C

ATOM 5135 H2X POPCO 9 -31.148 -25.731 -15.385 1.00 0.00 O2 H

ATOM 5136 H2Y POPCO 9 -32.036 -24.610 -14.220 1.00 0.00 O2 H

ATOM 5137 C23 POPCO 9 -36.167 -26.403 -8.963 1.00 0.00 O2 C

ATOM 5138 H3R POPCO 9 -37.271 -26.676 -8.892 1.00 0.00 O2 H

ATOM 5139 H3S POPCO 9 -35.703 -27.357 -9.241 1.00 0.00 O2 H

ATOM 5140 C24 POPCO 9 -35.653 -25.850 -7.654 1.00 0.00 O2 C

ATOM 5141 H4R POPCO 9 -34.595 -25.474 -7.826 1.00 0.00 O2 H

ATOM 5142 H4S POPCO 9 -36.251 -24.965 -7.362 1.00 0.00 O2 H

ATOM 5143 C25 POPCO 9 -35.757 -26.921 -6.512 1.00 0.00 O2 C

ATOM 5144 H5R POPCO 9 -36.826 -27.116 -6.290 1.00 0.00 O2 H

ATOM 5145 H5S POPCO 9 -35.367 -27.884 -6.851 1.00 0.00 O2 H

ATOM 5146 C26 POPCO 9 -35.108 -26.557 -5.168 1.00 0.00 O2 C

ATOM 5147 H6R POPCO 9 -34.019 -26.550 -5.394 1.00 0.00 O2 H

ATOM 5148 H6S POPCO 9 -35.355 -25.517 -4.896 1.00 0.00 O2 H

ATOM 5149 C27 POPCO 9 -35.591 -27.581 -4.127 1.00 0.00 O2 C

ATOM 5150 H7R POPCO 9 -36.596 -27.196 -3.752 1.00 0.00 O2 H

ATOM 5151 H7S POPCO 9 -35.796 -28.497 -4.605 1.00 0.00 O2 H

ATOM 5152 C28 POPCO 9 -34.717 -27.849 -2.908 1.00 0.00 O2 C

ATOM 5153 H8R POPCO 9 -33.698 -28.114 -3.288 1.00 0.00 O2 H

ATOM 5154 H8S POPCO 9 -34.596 -26.838 -2.410 1.00 0.00 O2 H

ATOM 5155 C29 POPCO 9 -35.319 -28.867 -1.978 1.00 0.00 O2 C

ATOM 5156 H91 POPCO 9 -35.914 -29.616 -2.447 1.00 0.00 O2 H

ATOM 5157 C210 POPCO 9 -35.206 -28.953 -0.618 1.00 0.00 O2 C

ATOM 5158 H101 POPCO 9 -35.813 -29.756 -0.120 1.00 0.00 O2 H

ATOM 5159 C211 POPCO 9 -34.480 -27.972 0.257 1.00 0.00 O2 C

ATOM 5160 H11R POPCO 9 -33.911 -27.186 -0.382 1.00 0.00 O2 H

ATOM 5161 H11S POPCO 9 -35.337 -27.454 0.807 1.00 0.00 O2 H

ATOM 5162 C212 POPCO 9 -33.548 -28.775 1.223 1.00 0.00 O2 C

ATOM 5163 H12R POPCO 9 -32.954 -29.427 0.527 1.00 0.00 O2 H

ATOM 5164 H12S POPCO 9 -32.822 -28.107 1.742 1.00 0.00 O2 H

ATOM 5165 C213 POPCO 9 -34.290 -29.394 2.393 1.00 0.00 O2 C

ATOM 5166 H13R POPCO 9 -34.768 -28.532 2.820 1.00 0.00 O2 H

ATOM 5167 H13S POPCO 9 -35.047 -30.114 1.985 1.00 0.00 O2 H

ATOM 5168 C214 POPCO 9 -33.453 -30.125 3.520 1.00 0.00 O2 C

ATOM 5169 H14R POPCO 9 -33.486 -31.248 3.449 1.00 0.00 O2 H

ATOM 5170 H14S POPCO 9 -32.444 -29.766 3.323 1.00 0.00 O2 H

ATOM 5171 C215 POPCO 9 -33.796 -29.685 4.958 1.00 0.00 O2 C

ATOM 5172 H15R POPCO 9 -33.339 -28.601 5.062 1.00 0.00 O2 H

ATOM 5173 H15S POPCO 9 -34.892 -29.664 5.153 1.00 0.00 O2 H

ATOM 5174 C216 POPCO 9 -33.183 -30.565 6.024 1.00 0.00 O2 C

ATOM 5175 H16R POPCO 9 -33.615 -31.608 6.058 1.00 0.00 O2 H

ATOM 5176 H16S POPCO 9 -32.097 -30.683 5.803 1.00 0.00 O2 H

ATOM 5177 C217 POPCO 9 -33.534 -29.906 7.383 1.00 0.00 O2 C

ATOM 5178 H17R POPCO 9 -33.323 -28.805 7.435 1.00 0.00 O2 H

ATOM 5179 H17S POPCO 9 -34.648 -30.128 7.503 1.00 0.00 O2 H

ATOM 5180 C218 POPCO 9 -32.790 -30.469 8.602 1.00 0.00 O2 C

ATOM 5181 H18R POPCO 9 -33.245 -29.932 9.485 1.00 0.00 O2 H

ATOM 5182 H18S POPCO 9 -32.994 -31.531 8.807 1.00 0.00 O2 H

ATOM 5183 H18T POPCO 9 -31.672 -30.453 8.532 1.00 0.00 O2 H

ATOM 5184 C33 POPCO 9 -30.522 -25.887 -13.275 1.00 0.00 O2 C

ATOM 5185 H3X POPCO 9 -29.980 -26.828 -13.371 1.00 0.00 O2 H

ATOM 5186 H3Y POPCO 9 -29.755 -25.108 -13.243 1.00 0.00 O2 H

ATOM 5187 C34 POPCO 9 -31.313 -25.841 -11.904 1.00 0.00 O2 C

ATOM 5188 H4X POPCO 9 -32.041 -24.913 -11.943 1.00 0.00 O2 H

ATOM 5189 H4Y POPCO 9 -31.867 -26.685 -11.642 1.00 0.00 O2 H

ATOM 5190 C35 POPCO 9 -30.407 -25.525 -10.695 1.00 0.00 O2 C

ATOM 5191 H5X POPCO 9 -29.518 -26.223 -10.765 1.00 0.00 O2 H

ATOM 5192 H5Y POPCO 9 -29.902 -24.522 -10.933 1.00 0.00 O2 H

ATOM 5193 C36 POPCO 9 -31.105 -25.439 -9.366 1.00 0.00 O2 C

ATOM 5194 H6X POPCO 9 -32.076 -24.856 -9.503 1.00 0.00 O2 H

ATOM 5195 H6Y POPCO 9 -31.518 -26.394 -9.015 1.00 0.00 O2 H

ATOM 5196 C37 POPCO 9 -30.469 -24.804 -8.198 1.00 0.00 O2 C

ATOM 5197 H7X POPCO 9 -29.519 -25.457 -8.005 1.00 0.00 O2 H

ATOM 5198 H7Y POPCO 9 -30.119 -23.743 -8.333 1.00 0.00 O2 H

ATOM 5199 C38 POPCO 9 -31.430 -25.005 -6.960 1.00 0.00 O2 C

ATOM 5200 H8X POPCO 9 -32.325 -24.331 -7.004 1.00 0.00 O2 H

ATOM 5201 H8Y POPCO 9 -31.836 -26.012 -6.897 1.00 0.00 O2 H

ATOM 5202 C39 POPCO 9 -30.618 -24.975 -5.710 1.00 0.00 O2 C

ATOM 5203 H9X POPCO 9 -29.837 -25.660 -5.796 1.00 0.00 O2 H

ATOM 5204 H9Y POPCO 9 -30.194 -23.945 -5.542 1.00 0.00 O2 H

ATOM 5205 C310 POPCO 9 -31.459 -25.350 -4.363 1.00 0.00 O2 C

ATOM 5206 H10X POPCO 9 -32.499 -25.061 -4.271 1.00 0.00 O2 H

ATOM 5207 H10Y POPCO 9 -31.436 -26.431 -4.370 1.00 0.00 O2 H

ATOM 5208 C311 POPCO 9 -30.707 -24.949 -3.002 1.00 0.00 O2 C

ATOM 5209 H11X POPCO 9 -29.652 -25.271 -3.014 1.00 0.00 O2 H

ATOM 5210 H11Y POPCO 9 -30.622 -23.851 -2.882 1.00 0.00 O2 H

ATOM 5211 C312 POPCO 9 -31.358 -25.549 -1.738 1.00 0.00 O2 C

ATOM 5212 H12X POPCO 9 -32.428 -25.308 -1.807 1.00 0.00 O2 H

ATOM 5213 H12Y POPCO 9 -31.369 -26.688 -1.773 1.00 0.00 O2 H

ATOM 5214 C313 POPCO 9 -30.677 -25.113 -0.407 1.00 0.00 O2 C

ATOM 5215 H13X POPCO 9 -29.591 -25.283 -0.471 1.00 0.00 O2 H

ATOM 5216 H13Y POPCO 9 -30.971 -24.050 -0.268 1.00 0.00 O2 H

ATOM 5217 C314 POPCO 9 -31.307 -25.683 0.889 1.00 0.00 O2 C

ATOM 5218 H14X POPCO 9 -32.444 -25.602 0.806 1.00 0.00 O2 H

ATOM 5219 H14Y POPCO 9 -31.066 -26.780 0.933 1.00 0.00 O2 H

ATOM 5220 C315 POPCO 9 -30.850 -25.049 2.224 1.00 0.00 O2 C

ATOM 5221 H15X POPCO 9 -29.743 -24.993 2.224 1.00 0.00 O2 H

ATOM 5222 H15Y POPCO 9 -31.251 -23.995 2.136 1.00 0.00 O2 H

ATOM 5223 C316 POPCO 9 -31.313 -25.791 3.472 1.00 0.00 O2 C

ATOM 5224 H16X POPCO 9 -31.099 -26.810 3.303 1.00 0.00 O2 H

ATOM 5225 H16Y POPCO 9 -30.899 -25.506 4.457 1.00 0.00 O2 H

ATOM 5226 H16Z POPCO 9 -32.383 -25.923 3.444 1.00 0.00 O2 H

ATOM 5227 N POPCO 10 -28.092 -17.685 -18.139 1.00 0.00 O2 N

ATOM 5228 C12 POPCO 10 -28.647 -19.114 -17.722 1.00 0.00 O2 C

ATOM 5229 H12A POPCO 10 -28.019 -19.904 -18.041 1.00 0.00 O2 H

ATOM 5230 H12B POPCO 10 -29.574 -19.227 -18.232 1.00 0.00 O2 H

ATOM 5231 C13 POPCO 10 -26.720 -17.605 -17.821 1.00 0.00 O2 C

ATOM 5232 H13A POPCO 10 -26.080 -18.494 -18.069 1.00 0.00 O2 H

ATOM 5233 H13B POPCO 10 -26.553 -17.530 -16.700 1.00 0.00 O2 H

ATOM 5234 H13C POPCO 10 -26.259 -16.786 -18.351 1.00 0.00 O2 H

ATOM 5235 C14 POPCO 10 -28.218 -17.471 -19.667 1.00 0.00 O2 C

ATOM 5236 H14A POPCO 10 -29.261 -17.476 -19.921 1.00 0.00 O2 H

ATOM 5237 H14B POPCO 10 -27.799 -18.377 -20.114 1.00 0.00 O2 H

ATOM 5238 H14C POPCO 10 -27.616 -16.622 -19.976 1.00 0.00 O2 H

ATOM 5239 C15 POPCO 10 -28.651 -16.577 -17.322 1.00 0.00 O2 C

ATOM 5240 H15A POPCO 10 -29.641 -16.366 -17.663 1.00 0.00 O2 H

ATOM 5241 H15B POPCO 10 -28.554 -16.741 -16.223 1.00 0.00 O2 H

ATOM 5242 H15C POPCO 10 -28.014 -15.769 -17.499 1.00 0.00 O2 H

ATOM 5243 C11 POPCO 10 -28.899 -19.448 -16.184 1.00 0.00 O2 C

ATOM 5244 H11A POPCO 10 -29.635 -18.666 -15.926 1.00 0.00 O2 H

ATOM 5245 H11B POPCO 10 -29.396 -20.370 -15.933 1.00 0.00 O2 H

ATOM 5246 P POPCO 10 -27.337 -18.418 -14.198 1.00 0.00 O2 P

ATOM 5247 O13 POPCO 10 -26.169 -17.547 -14.574 1.00 0.00 O2 O

ATOM 5248 O14 POPCO 10 -27.087 -19.331 -13.060 1.00 0.00 O2 O

ATOM 5249 O12 POPCO 10 -27.724 -19.287 -15.456 1.00 0.00 O2 O

ATOM 5250 O11 POPCO 10 -28.625 -17.520 -13.960 1.00 0.00 O2 O

ATOM 5251 C1 POPCO 10 -29.266 -17.759 -12.676 1.00 0.00 O2 C

ATOM 5252 HA POPCO 10 -29.597 -18.803 -12.648 1.00 0.00 O2 H

ATOM 5253 HB POPCO 10 -28.547 -17.573 -11.752 1.00 0.00 O2 H

ATOM 5254 C2 POPCO 10 -30.518 -16.834 -12.491 1.00 0.00 O2 C

ATOM 5255 HS POPCO 10 -30.226 -15.765 -12.700 1.00 0.00 O2 H

ATOM 5256 O21 POPCO 10 -31.123 -17.055 -11.140 1.00 0.00 O2 O

ATOM 5257 C21 POPCO 10 -30.755 -16.264 -10.180 1.00 0.00 O2 C

ATOM 5258 O22 POPCO 10 -30.294 -15.147 -10.307 1.00 0.00 O2 O

ATOM 5259 C22 POPCO 10 -30.893 -16.973 -8.815 1.00 0.00 O2 C

ATOM 5260 H2R POPCO 10 -31.827 -17.570 -8.719 1.00 0.00 O2 H

ATOM 5261 H2S POPCO 10 -30.105 -17.773 -8.649 1.00 0.00 O2 H

ATOM 5262 C3 POPCO 10 -31.543 -17.134 -13.607 1.00 0.00 O2 C

ATOM 5263 HX POPCO 10 -32.480 -16.547 -13.395 1.00 0.00 O2 H

ATOM 5264 HY POPCO 10 -31.023 -16.866 -14.543 1.00 0.00 O2 H

ATOM 5265 O31 POPCO 10 -31.925 -18.564 -13.724 1.00 0.00 O2 O

ATOM 5266 C31 POPCO 10 -32.880 -18.907 -14.518 1.00 0.00 O2 C

ATOM 5267 O32 POPCO 10 -33.369 -18.268 -15.432 1.00 0.00 O2 O

ATOM 5268 C32 POPCO 10 -33.290 -20.397 -14.311 1.00 0.00 O2 C

ATOM 5269 H2X POPCO 10 -33.966 -20.662 -15.169 1.00 0.00 O2 H

ATOM 5270 H2Y POPCO 10 -32.350 -21.007 -14.425 1.00 0.00 O2 H

ATOM 5271 C23 POPCO 10 -31.046 -16.001 -7.636 1.00 0.00 O2 C

ATOM 5272 H3R POPCO 10 -30.323 -15.203 -7.668 1.00 0.00 O2 H

ATOM 5273 H3S POPCO 10 -31.970 -15.383 -7.742 1.00 0.00 O2 H

ATOM 5274 C24 POPCO 10 -30.922 -16.741 -6.241 1.00 0.00 O2 C

ATOM 5275 H4R POPCO 10 -31.543 -17.660 -6.092 1.00 0.00 O2 H

ATOM 5276 H4S POPCO 10 -29.888 -17.100 -6.060 1.00 0.00 O2 H

ATOM 5277 C25 POPCO 10 -30.959 -15.761 -5.005 1.00 0.00 O2 C

ATOM 5278 H5R POPCO 10 -30.295 -14.852 -5.250 1.00 0.00 O2 H

ATOM 5279 H5S POPCO 10 -31.966 -15.299 -4.970 1.00 0.00 O2 H

ATOM 5280 C26 POPCO 10 -30.535 -16.452 -3.731 1.00 0.00 O2 C

ATOM 5281 H6R POPCO 10 -31.359 -17.196 -3.387 1.00 0.00 O2 H

ATOM 5282 H6S POPCO 10 -29.585 -17.032 -3.816 1.00 0.00 O2 H

ATOM 5283 C27 POPCO 10 -30.342 -15.455 -2.543 1.00 0.00 O2 C

ATOM 5284 H7R POPCO 10 -29.544 -14.771 -2.897 1.00 0.00 O2 H

ATOM 5285 H7S POPCO 10 -31.295 -14.860 -2.444 1.00 0.00 O2 H

ATOM 5286 C28 POPCO 10 -29.973 -16.243 -1.280 1.00 0.00 O2 C

ATOM 5287 H8R POPCO 10 -30.773 -17.029 -1.084 1.00 0.00 O2 H

ATOM 5288 H8S POPCO 10 -29.036 -16.811 -1.470 1.00 0.00 O2 H

ATOM 5289 C29 POPCO 10 -29.673 -15.345 -0.015 1.00 0.00 O2 C

ATOM 5290 H91 POPCO 10 -28.727 -14.722 0.005 1.00 0.00 O2 H

ATOM 5291 C210 POPCO 10 -30.353 -15.192 1.106 1.00 0.00 O2 C

ATOM 5292 H101 POPCO 10 -29.868 -14.510 1.830 1.00 0.00 O2 H

ATOM 5293 C211 POPCO 10 -31.647 -15.813 1.396 1.00 0.00 O2 C

ATOM 5294 H11R POPCO 10 -32.392 -15.047 1.470 1.00 0.00 O2 H

ATOM 5295 H11S POPCO 10 -32.072 -16.503 0.651 1.00 0.00 O2 H

ATOM 5296 C212 POPCO 10 -31.503 -16.663 2.756 1.00 0.00 O2 C

ATOM 5297 H12R POPCO 10 -32.382 -17.367 2.710 1.00 0.00 O2 H

ATOM 5298 H12S POPCO 10 -30.554 -17.127 2.636 1.00 0.00 O2 H

ATOM 5299 C213 POPCO 10 -31.538 -15.886 4.059 1.00 0.00 O2 C

ATOM 5300 H13R POPCO 10 -30.637 -15.237 4.066 1.00 0.00 O2 H

ATOM 5301 H13S POPCO 10 -32.456 -15.287 4.101 1.00 0.00 O2 H

ATOM 5302 C214 POPCO 10 -31.347 -16.772 5.306 1.00 0.00 O2 C

ATOM 5303 H14R POPCO 10 -31.933 -17.696 5.223 1.00 0.00 O2 H

ATOM 5304 H14S POPCO 10 -30.231 -16.946 5.365 1.00 0.00 O2 H

ATOM 5305 C215 POPCO 10 -31.725 -16.139 6.713 1.00 0.00 O2 C

ATOM 5306 H15R POPCO 10 -31.144 -15.142 6.880 1.00 0.00 O2 H

ATOM 5307 H15S POPCO 10 -32.857 -15.867 6.655 1.00 0.00 O2 H

ATOM 5308 C216 POPCO 10 -31.457 -16.972 8.040 1.00 0.00 O2 C

ATOM 5309 H16R POPCO 10 -32.199 -17.796 8.176 1.00 0.00 O2 H

ATOM 5310 H16S POPCO 10 -30.471 -17.504 7.845 1.00 0.00 O2 H

ATOM 5311 C217 POPCO 10 -31.290 -16.122 9.323 1.00 0.00 O2 C

ATOM 5312 H17R POPCO 10 -30.482 -15.341 9.126 1.00 0.00 O2 H

ATOM 5313 H17S POPCO 10 -32.256 -15.711 9.497 1.00 0.00 O2 H

ATOM 5314 C218 POPCO 10 -30.877 -16.927 10.595 1.00 0.00 O2 C

ATOM 5315 H18R POPCO 10 -31.217 -16.401 11.438 1.00 0.00 O2 H

ATOM 5316 H18S POPCO 10 -31.538 -17.817 10.654 1.00 0.00 O2 H

ATOM 5317 H18T POPCO 10 -29.751 -17.016 10.586 1.00 0.00 O2 H

ATOM 5318 C33 POPCO 10 -34.140 -20.713 -13.092 1.00 0.00 O2 C

ATOM 5319 H3X POPCO 10 -35.133 -20.262 -13.273 1.00 0.00 O2 H

ATOM 5320 H3Y POPCO 10 -34.347 -21.837 -12.930 1.00 0.00 O2 H

ATOM 5321 C34 POPCO 10 -33.650 -20.116 -11.770 1.00 0.00 O2 C

ATOM 5322 H4X POPCO 10 -32.519 -20.289 -11.643 1.00 0.00 O2 H

ATOM 5323 H4Y POPCO 10 -33.819 -19.006 -11.794 1.00 0.00 O2 H

ATOM 5324 C35 POPCO 10 -34.274 -20.664 -10.478 1.00 0.00 O2 C

ATOM 5325 H5X POPCO 10 -35.381 -20.490 -10.559 1.00 0.00 O2 H

ATOM 5326 H5Y POPCO 10 -34.040 -21.823 -10.444 1.00 0.00 O2 H

ATOM 5327 C36 POPCO 10 -33.709 -19.994 -9.211 1.00 0.00 O2 C

ATOM 5328 H6X POPCO 10 -32.557 -19.889 -9.208 1.00 0.00 O2 H

ATOM 5329 H6Y POPCO 10 -34.029 -18.930 -9.197 1.00 0.00 O2 H

ATOM 5330 C37 POPCO 10 -34.096 -20.615 -7.753 1.00 0.00 O2 C

ATOM 5331 H7X POPCO 10 -35.142 -20.837 -7.665 1.00 0.00 O2 H

ATOM 5332 H7Y POPCO 10 -33.622 -21.601 -7.642 1.00 0.00 O2 H

ATOM 5333 C38 POPCO 10 -33.486 -19.661 -6.676 1.00 0.00 O2 C

ATOM 5334 H8X POPCO 10 -32.408 -19.586 -6.846 1.00 0.00 O2 H

ATOM 5335 H8Y POPCO 10 -34.102 -18.742 -6.671 1.00 0.00 O2 H

ATOM 5336 C39 POPCO 10 -33.659 -20.427 -5.326 1.00 0.00 O2 C

ATOM 5337 H9X POPCO 10 -34.641 -20.834 -5.384 1.00 0.00 O2 H

ATOM 5338 H9Y POPCO 10 -32.978 -21.314 -5.293 1.00 0.00 O2 H

ATOM 5339 C310 POPCO 10 -33.616 -19.415 -4.106 1.00 0.00 O2 C

ATOM 5340 H10X POPCO 10 -32.616 -18.919 -4.129 1.00 0.00 O2 H

ATOM 5341 H10Y POPCO 10 -34.385 -18.667 -4.278 1.00 0.00 O2 H

ATOM 5342 C311 POPCO 10 -33.805 -20.219 -2.800 1.00 0.00 O2 C

ATOM 5343 H11X POPCO 10 -34.884 -20.475 -2.700 1.00 0.00 O2 H

ATOM 5344 H11Y POPCO 10 -33.284 -21.154 -3.038 1.00 0.00 O2 H

ATOM 5345 C312 POPCO 10 -33.287 -19.505 -1.518 1.00 0.00 O2 C

ATOM 5346 H12X POPCO 10 -32.180 -19.522 -1.761 1.00 0.00 O2 H

ATOM 5347 H12Y POPCO 10 -33.682 -18.509 -1.575 1.00 0.00 O2 H

ATOM 5348 C313 POPCO 10 -33.744 -20.227 -0.283 1.00 0.00 O2 C

ATOM 5349 H13X POPCO 10 -34.898 -20.271 -0.208 1.00 0.00 O2 H

ATOM 5350 H13Y POPCO 10 -33.370 -21.239 -0.239 1.00 0.00 O2 H

ATOM 5351 C314 POPCO 10 -33.219 -19.694 1.066 1.00 0.00 O2 C

ATOM 5352 H14X POPCO 10 -32.115 -19.576 1.035 1.00 0.00 O2 H

ATOM 5353 H14Y POPCO 10 -33.675 -18.626 1.116 1.00 0.00 O2 H

ATOM 5354 C315 POPCO 10 -33.723 -20.395 2.367 1.00 0.00 O2 C

ATOM 5355 H15X POPCO 10 -34.820 -20.658 2.346 1.00 0.00 O2 H

ATOM 5356 H15Y POPCO 10 -33.211 -21.375 2.408 1.00 0.00 O2 H

ATOM 5357 C316 POPCO 10 -33.531 -19.551 3.645 1.00 0.00 O2 C

ATOM 5358 H16X POPCO 10 -34.139 -18.594 3.548 1.00 0.00 O2 H

ATOM 5359 H16Y POPCO 10 -33.937 -20.109 4.574 1.00 0.00 O2 H

ATOM 5360 H16Z POPCO 10 -32.498 -19.197 3.924 1.00 0.00 O2 H

ATOM 5361 N POPCO 11 -34.075 -13.344 -24.850 1.00 0.00 O2 N

ATOM 5362 C12 POPCO 11 -35.018 -12.905 -23.670 1.00 0.00 O2 C

ATOM 5363 H12A POPCO 11 -36.002 -13.324 -23.784 1.00 0.00 O2 H

ATOM 5364 H12B POPCO 11 -35.186 -11.821 -23.701 1.00 0.00 O2 H

ATOM 5365 C13 POPCO 11 -34.256 -14.701 -25.203 1.00 0.00 O2 C

ATOM 5366 H13A POPCO 11 -35.256 -14.980 -25.489 1.00 0.00 O2 H

ATOM 5367 H13B POPCO 11 -34.090 -15.222 -24.284 1.00 0.00 O2 H

ATOM 5368 H13C POPCO 11 -33.619 -15.056 -25.966 1.00 0.00 O2 H

ATOM 5369 C14 POPCO 11 -34.227 -12.451 -26.043 1.00 0.00 O2 C

ATOM 5370 H14A POPCO 11 -34.021 -11.409 -25.824 1.00 0.00 O2 H

ATOM 5371 H14B POPCO 11 -35.200 -12.517 -26.484 1.00 0.00 O2 H

ATOM 5372 H14C POPCO 11 -33.560 -12.784 -26.877 1.00 0.00 O2 H

ATOM 5373 C15 POPCO 11 -32.624 -13.307 -24.377 1.00 0.00 O2 C

ATOM 5374 H15A POPCO 11 -32.343 -12.356 -23.972 1.00 0.00 O2 H

ATOM 5375 H15B POPCO 11 -32.484 -14.103 -23.650 1.00 0.00 O2 H

ATOM 5376 H15C POPCO 11 -31.890 -13.498 -25.101 1.00 0.00 O2 H

ATOM 5377 C11 POPCO 11 -34.525 -13.277 -22.289 1.00 0.00 O2 C

ATOM 5378 H11A POPCO 11 -33.635 -12.811 -21.832 1.00 0.00 O2 H

ATOM 5379 H11B POPCO 11 -35.285 -12.874 -21.552 1.00 0.00 O2 H

ATOM 5380 P POPCO 11 -33.509 -15.700 -21.741 1.00 0.00 O2 P

ATOM 5381 O13 POPCO 11 -32.935 -16.053 -23.061 1.00 0.00 O2 O

ATOM 5382 O14 POPCO 11 -33.933 -16.829 -20.883 1.00 0.00 O2 O

ATOM 5383 O12 POPCO 11 -34.685 -14.699 -22.114 1.00 0.00 O2 O

ATOM 5384 O11 POPCO 11 -32.519 -14.694 -20.957 1.00 0.00 O2 O

ATOM 5385 C1 POPCO 11 -32.874 -14.420 -19.547 1.00 0.00 O2 C

ATOM 5386 HA POPCO 11 -33.822 -13.810 -19.402 1.00 0.00 O2 H

ATOM 5387 HB POPCO 11 -32.979 -15.400 -18.991 1.00 0.00 O2 H

ATOM 5388 C2 POPCO 11 -31.804 -13.564 -18.776 1.00 0.00 O2 C

ATOM 5389 HS POPCO 11 -30.868 -14.172 -18.740 1.00 0.00 O2 H

ATOM 5390 O21 POPCO 11 -32.305 -13.252 -17.514 1.00 0.00 O2 O

ATOM 5391 C21 POPCO 11 -31.388 -13.106 -16.595 1.00 0.00 O2 C

ATOM 5392 O22 POPCO 11 -30.258 -13.608 -16.610 1.00 0.00 O2 O

ATOM 5393 C22 POPCO 11 -32.080 -12.311 -15.446 1.00 0.00 O2 C

ATOM 5394 H2R POPCO 11 -31.603 -11.354 -15.575 1.00 0.00 O2 H

ATOM 5395 H2S POPCO 11 -33.096 -12.104 -15.716 1.00 0.00 O2 H

ATOM 5396 C3 POPCO 11 -31.569 -12.257 -19.540 1.00 0.00 O2 C

ATOM 5397 HX POPCO 11 -30.649 -11.826 -19.202 1.00 0.00 O2 H

ATOM 5398 HY POPCO 11 -31.273 -12.554 -20.580 1.00 0.00 O2 H

ATOM 5399 O31 POPCO 11 -32.757 -11.378 -19.540 1.00 0.00 O2 O

ATOM 5400 C31 POPCO 11 -32.797 -10.431 -20.506 1.00 0.00 O2 C

ATOM 5401 O32 POPCO 11 -32.094 -10.474 -21.478 1.00 0.00 O2 O

ATOM 5402 C32 POPCO 11 -33.758 -9.309 -20.054 1.00 0.00 O2 C

ATOM 5403 H2X POPCO 11 -33.846 -8.712 -20.958 1.00 0.00 O2 H

ATOM 5404 H2Y POPCO 11 -34.774 -9.695 -19.900 1.00 0.00 O2 H

ATOM 5405 C23 POPCO 11 -31.758 -12.851 -14.043 1.00 0.00 O2 C

ATOM 5406 H3R POPCO 11 -32.089 -13.929 -14.022 1.00 0.00 O2 H

ATOM 5407 H3S POPCO 11 -30.643 -12.685 -13.852 1.00 0.00 O2 H

ATOM 5408 C24 POPCO 11 -32.514 -12.106 -12.915 1.00 0.00 O2 C

ATOM 5409 H4R POPCO 11 -32.302 -11.072 -12.869 1.00 0.00 O2 H

ATOM 5410 H4S POPCO 11 -33.647 -12.278 -12.857 1.00 0.00 O2 H

ATOM 5411 C25 POPCO 11 -32.049 -12.704 -11.645 1.00 0.00 O2 C

ATOM 5412 H5R POPCO 11 -32.428 -13.793 -11.599 1.00 0.00 O2 H

ATOM 5413 H5S POPCO 11 -30.978 -12.767 -11.644 1.00 0.00 O2 H

ATOM 5414 C26 POPCO 11 -32.578 -11.950 -10.369 1.00 0.00 O2 C

ATOM 5415 H6R POPCO 11 -32.051 -10.992 -10.229 1.00 0.00 O2 H

ATOM 5416 H6S POPCO 11 -33.663 -11.883 -10.391 1.00 0.00 O2 H

ATOM 5417 C27 POPCO 11 -32.183 -12.732 -9.106 1.00 0.00 O2 C

ATOM 5418 H7R POPCO 11 -32.702 -13.687 -9.120 1.00 0.00 O2 H

ATOM 5419 H7S POPCO 11 -31.084 -12.870 -9.153 1.00 0.00 O2 H

ATOM 5420 C28 POPCO 11 -32.675 -12.067 -7.815 1.00 0.00 O2 C

ATOM 5421 H8R POPCO 11 -32.410 -11.005 -7.931 1.00 0.00 O2 H

ATOM 5422 H8S POPCO 11 -33.764 -12.138 -7.680 1.00 0.00 O2 H

ATOM 5423 C29 POPCO 11 -31.955 -12.619 -6.578 1.00 0.00 O2 C

ATOM 5424 H91 POPCO 11 -31.006 -13.152 -6.858 1.00 0.00 O2 H

ATOM 5425 C210 POPCO 11 -32.132 -12.425 -5.282 1.00 0.00 O2 C

ATOM 5426 H101 POPCO 11 -31.544 -12.984 -4.573 1.00 0.00 O2 H

ATOM 5427 C211 POPCO 11 -33.256 -11.584 -4.785 1.00 0.00 O2 C

ATOM 5428 H11R POPCO 11 -33.499 -10.852 -5.528 1.00 0.00 O2 H

ATOM 5429 H11S POPCO 11 -34.155 -12.207 -4.599 1.00 0.00 O2 H

ATOM 5430 C212 POPCO 11 -33.040 -10.837 -3.387 1.00 0.00 O2 C

ATOM 5431 H12R POPCO 11 -32.112 -10.154 -3.420 1.00 0.00 O2 H

ATOM 5432 H12S POPCO 11 -33.952 -10.193 -3.276 1.00 0.00 O2 H

ATOM 5433 C213 POPCO 11 -32.906 -11.783 -2.240 1.00 0.00 O2 C

ATOM 5434 H13R POPCO 11 -33.836 -12.405 -2.348 1.00 0.00 O2 H

ATOM 5435 H13S POPCO 11 -32.039 -12.543 -2.535 1.00 0.00 O2 H

ATOM 5436 C214 POPCO 11 -32.811 -11.035 -0.908 1.00 0.00 O2 C

ATOM 5437 H14R POPCO 11 -31.967 -10.324 -0.933 1.00 0.00 O2 H

ATOM 5438 H14S POPCO 11 -33.797 -10.438 -0.725 1.00 0.00 O2 H

ATOM 5439 C215 POPCO 11 -32.578 -11.880 0.304 1.00 0.00 O2 C

ATOM 5440 H15R POPCO 11 -33.155 -12.842 0.357 1.00 0.00 O2 H

ATOM 5441 H15S POPCO 11 -31.534 -12.353 0.238 1.00 0.00 O2 H

ATOM 5442 C216 POPCO 11 -32.656 -11.083 1.633 1.00 0.00 O2 C

ATOM 5443 H16R POPCO 11 -32.174 -10.133 1.472 1.00 0.00 O2 H

ATOM 5444 H16S POPCO 11 -33.688 -10.807 1.849 1.00 0.00 O2 H

ATOM 5445 C217 POPCO 11 -32.029 -11.841 2.791 1.00 0.00 O2 C

ATOM 5446 H17R POPCO 11 -32.442 -12.835 2.865 1.00 0.00 O2 H

ATOM 5447 H17S POPCO 11 -30.969 -12.108 2.610 1.00 0.00 O2 H

ATOM 5448 C218 POPCO 11 -32.089 -10.999 4.147 1.00 0.00 O2 C

ATOM 5449 H18R POPCO 11 -32.180 -11.623 5.014 1.00 0.00 O2 H

ATOM 5450 H18S POPCO 11 -31.169 -10.250 4.119 1.00 0.00 O2 H

ATOM 5451 H18T POPCO 11 -32.969 -10.397 4.088 1.00 0.00 O2 H

ATOM 5452 C33 POPCO 11 -33.324 -8.443 -18.831 1.00 0.00 O2 C

ATOM 5453 H3X POPCO 11 -32.392 -8.017 -19.159 1.00 0.00 O2 H

ATOM 5454 H3Y POPCO 11 -34.103 -7.575 -18.760 1.00 0.00 O2 H

ATOM 5455 C34 POPCO 11 -33.139 -9.049 -17.455 1.00 0.00 O2 C

ATOM 5456 H4X POPCO 11 -33.871 -9.794 -17.126 1.00 0.00 O2 H

ATOM 5457 H4Y POPCO 11 -32.035 -9.504 -17.367 1.00 0.00 O2 H

ATOM 5458 C35 POPCO 11 -33.201 -7.929 -16.389 1.00 0.00 O2 C

ATOM 5459 H5X POPCO 11 -32.425 -7.104 -16.572 1.00 0.00 O2 H

ATOM 5460 H5Y POPCO 11 -34.216 -7.383 -16.475 1.00 0.00 O2 H

ATOM 5461 C36 POPCO 11 -33.159 -8.625 -14.998 1.00 0.00 O2 C

ATOM 5462 H6X POPCO 11 -33.924 -9.490 -15.053 1.00 0.00 O2 H

ATOM 5463 H6Y POPCO 11 -32.152 -9.076 -14.912 1.00 0.00 O2 H

ATOM 5464 C37 POPCO 11 -33.501 -7.731 -13.732 1.00 0.00 O2 C

ATOM 5465 H7X POPCO 11 -32.823 -6.828 -13.850 1.00 0.00 O2 H

ATOM 5466 H7Y POPCO 11 -34.619 -7.458 -13.893 1.00 0.00 O2 H

ATOM 5467 C38 POPCO 11 -33.159 -8.321 -12.338 1.00 0.00 O2 C

ATOM 5468 H8X POPCO 11 -33.698 -9.293 -12.337 1.00 0.00 O2 H

ATOM 5469 H8Y POPCO 11 -32.075 -8.533 -12.061 1.00 0.00 O2 H

ATOM 5470 C39 POPCO 11 -33.721 -7.502 -11.156 1.00 0.00 O2 C

ATOM 5471 H9X POPCO 11 -33.433 -6.436 -11.130 1.00 0.00 O2 H

ATOM 5472 H9Y POPCO 11 -34.856 -7.684 -11.233 1.00 0.00 O2 H

ATOM 5473 C310 POPCO 11 -33.328 -8.013 -9.786 1.00 0.00 O2 C

ATOM 5474 H10X POPCO 11 -33.517 -9.128 -9.711 1.00 0.00 O2 H

ATOM 5475 H10Y POPCO 11 -32.250 -7.805 -9.775 1.00 0.00 O2 H

ATOM 5476 C311 POPCO 11 -34.048 -7.233 -8.678 1.00 0.00 O2 C

ATOM 5477 H11X POPCO 11 -33.637 -6.214 -8.778 1.00 0.00 O2 H

ATOM 5478 H11Y POPCO 11 -35.202 -7.194 -8.832 1.00 0.00 O2 H

ATOM 5479 C312 POPCO 11 -33.637 -7.688 -7.233 1.00 0.00 O2 C

ATOM 5480 H12X POPCO 11 -33.565 -8.796 -7.105 1.00 0.00 O2 H

ATOM 5481 H12Y POPCO 11 -32.571 -7.395 -7.195 1.00 0.00 O2 H

ATOM 5482 C313 POPCO 11 -34.402 -7.068 -6.055 1.00 0.00 O2 C

ATOM 5483 H13X POPCO 11 -34.530 -5.975 -6.058 1.00 0.00 O2 H

ATOM 5484 H13Y POPCO 11 -35.425 -7.431 -6.040 1.00 0.00 O2 H

ATOM 5485 C314 POPCO 11 -33.775 -7.514 -4.704 1.00 0.00 O2 C

ATOM 5486 H14X POPCO 11 -33.663 -8.599 -4.760 1.00 0.00 O2 H

ATOM 5487 H14Y POPCO 11 -32.831 -7.042 -4.621 1.00 0.00 O2 H

ATOM 5488 C315 POPCO 11 -34.615 -7.234 -3.437 1.00 0.00 O2 C

ATOM 5489 H15X POPCO 11 -35.281 -8.077 -3.348 1.00 0.00 O2 H

ATOM 5490 H15Y POPCO 11 -33.972 -7.317 -2.510 1.00 0.00 O2 H

ATOM 5491 C316 POPCO 11 -35.299 -5.864 -3.376 1.00 0.00 O2 C

ATOM 5492 H16X POPCO 11 -35.895 -5.673 -4.218 1.00 0.00 O2 H

ATOM 5493 H16Y POPCO 11 -35.927 -5.719 -2.414 1.00 0.00 O2 H

ATOM 5494 H16Z POPCO 11 -34.438 -5.151 -3.269 1.00 0.00 O2 H

ATOM 5495 N POPCO 12 -26.975 -3.373 -20.829 1.00 0.00 O2 N

ATOM 5496 C12 POPCO 12 -28.372 -2.821 -20.376 1.00 0.00 O2 C

ATOM 5497 H12A POPCO 12 -28.680 -2.079 -21.030 1.00 0.00 O2 H

ATOM 5498 H12B POPCO 12 -28.087 -2.197 -19.525 1.00 0.00 O2 H

ATOM 5499 C13 POPCO 12 -27.022 -3.763 -22.329 1.00 0.00 O2 C

ATOM 5500 H13A POPCO 12 -27.613 -3.000 -22.854 1.00 0.00 O2 H

ATOM 5501 H13B POPCO 12 -27.509 -4.798 -22.350 1.00 0.00 O2 H

ATOM 5502 H13C POPCO 12 -25.993 -3.837 -22.666 1.00 0.00 O2 H

ATOM 5503 C14 POPCO 12 -25.962 -2.322 -20.620 1.00 0.00 O2 C

ATOM 5504 H14A POPCO 12 -25.779 -2.304 -19.555 1.00 0.00 O2 H

ATOM 5505 H14B POPCO 12 -26.093 -1.332 -21.065 1.00 0.00 O2 H

ATOM 5506 H14C POPCO 12 -25.034 -2.728 -20.949 1.00 0.00 O2 H

ATOM 5507 C15 POPCO 12 -26.576 -4.622 -20.154 1.00 0.00 O2 C

ATOM 5508 H15A POPCO 12 -26.708 -4.529 -19.072 1.00 0.00 O2 H

ATOM 5509 H15B POPCO 12 -27.243 -5.472 -20.511 1.00 0.00 O2 H

ATOM 5510 H15C POPCO 12 -25.611 -4.945 -20.282 1.00 0.00 O2 H

ATOM 5511 C11 POPCO 12 -29.522 -3.744 -19.912 1.00 0.00 O2 C

ATOM 5512 H11A POPCO 12 -29.266 -4.166 -18.916 1.00 0.00 O2 H

ATOM 5513 H11B POPCO 12 -30.416 -3.064 -19.753 1.00 0.00 O2 H

ATOM 5514 P POPCO 12 -29.415 -6.289 -20.692 1.00 0.00 O2 P

ATOM 5515 O13 POPCO 12 -28.266 -6.541 -21.629 1.00 0.00 O2 O

ATOM 5516 O14 POPCO 12 -30.532 -7.181 -20.818 1.00 0.00 O2 O

ATOM 5517 O12 POPCO 12 -29.806 -4.767 -20.800 1.00 0.00 O2 O

ATOM 5518 O11 POPCO 12 -28.788 -6.522 -19.159 1.00 0.00 O2 O

ATOM 5519 C1 POPCO 12 -29.637 -6.741 -18.099 1.00 0.00 O2 C

ATOM 5520 HA POPCO 12 -30.352 -5.922 -17.898 1.00 0.00 O2 H

ATOM 5521 HB POPCO 12 -30.197 -7.694 -18.266 1.00 0.00 O2 H

ATOM 5522 C2 POPCO 12 -28.781 -7.011 -16.811 1.00 0.00 O2 C

ATOM 5523 HS POPCO 12 -28.606 -8.153 -16.921 1.00 0.00 O2 H

ATOM 5524 O21 POPCO 12 -29.613 -6.665 -15.704 1.00 0.00 O2 O

ATOM 5525 C21 POPCO 12 -29.543 -7.513 -14.632 1.00 0.00 O2 C

ATOM 5526 O22 POPCO 12 -29.303 -8.684 -14.735 1.00 0.00 O2 O

ATOM 5527 C22 POPCO 12 -29.721 -6.797 -13.274 1.00 0.00 O2 C

ATOM 5528 H2R POPCO 12 -29.088 -5.934 -13.354 1.00 0.00 O2 H

ATOM 5529 H2S POPCO 12 -30.744 -6.371 -13.237 1.00 0.00 O2 H

ATOM 5530 C3 POPCO 12 -27.356 -6.349 -16.662 1.00 0.00 O2 C

ATOM 5531 HX POPCO 12 -26.988 -6.526 -15.648 1.00 0.00 O2 H

ATOM 5532 HY POPCO 12 -26.654 -6.771 -17.477 1.00 0.00 O2 H

ATOM 5533 O31 POPCO 12 -27.318 -4.880 -16.995 1.00 0.00 O2 O

ATOM 5534 C31 POPCO 12 -27.536 -4.050 -15.887 1.00 0.00 O2 C

ATOM 5535 O32 POPCO 12 -27.860 -4.457 -14.810 1.00 0.00 O2 O

ATOM 5536 C32 POPCO 12 -27.589 -2.578 -16.357 1.00 0.00 O2 C

ATOM 5537 H2X POPCO 12 -26.817 -2.404 -17.046 1.00 0.00 O2 H

ATOM 5538 H2Y POPCO 12 -28.530 -2.408 -16.816 1.00 0.00 O2 H

ATOM 5539 C23 POPCO 12 -29.251 -7.604 -12.050 1.00 0.00 O2 C

ATOM 5540 H3R POPCO 12 -29.905 -8.419 -11.807 1.00 0.00 O2 H

ATOM 5541 H3S POPCO 12 -28.369 -8.090 -12.261 1.00 0.00 O2 H

ATOM 5542 C24 POPCO 12 -29.123 -6.644 -10.879 1.00 0.00 O2 C

ATOM 5543 H4R POPCO 12 -28.113 -6.203 -11.060 1.00 0.00 O2 H

ATOM 5544 H4S POPCO 12 -29.961 -5.873 -10.857 1.00 0.00 O2 H

ATOM 5545 C25 POPCO 12 -29.210 -7.201 -9.399 1.00 0.00 O2 C

ATOM 5546 H5R POPCO 12 -30.266 -7.633 -9.220 1.00 0.00 O2 H

ATOM 5547 H5S POPCO 12 -28.473 -7.997 -9.206 1.00 0.00 O2 H

ATOM 5548 C26 POPCO 12 -28.876 -6.040 -8.415 1.00 0.00 O2 C

ATOM 5549 H6R POPCO 12 -27.877 -5.599 -8.602 1.00 0.00 O2 H

ATOM 5550 H6S POPCO 12 -29.742 -5.307 -8.575 1.00 0.00 O2 H

ATOM 5551 C27 POPCO 12 -29.037 -6.285 -6.890 1.00 0.00 O2 C

ATOM 5552 H7R POPCO 12 -29.830 -7.043 -6.863 1.00 0.00 O2 H

ATOM 5553 H7S POPCO 12 -28.030 -6.718 -6.621 1.00 0.00 O2 H

ATOM 5554 C28 POPCO 12 -29.454 -5.040 -6.023 1.00 0.00 O2 C

ATOM 5555 H8R POPCO 12 -28.743 -4.165 -6.312 1.00 0.00 O2 H

ATOM 5556 H8S POPCO 12 -30.437 -4.642 -6.302 1.00 0.00 O2 H

ATOM 5557 C29 POPCO 12 -29.439 -5.550 -4.567 1.00 0.00 O2 C

ATOM 5558 H91 POPCO 12 -30.033 -6.420 -4.480 1.00 0.00 O2 H

ATOM 5559 C210 POPCO 12 -28.736 -5.035 -3.508 1.00 0.00 O2 C

ATOM 5560 H101 POPCO 12 -28.930 -5.452 -2.538 1.00 0.00 O2 H

ATOM 5561 C211 POPCO 12 -27.978 -3.770 -3.429 1.00 0.00 O2 C

ATOM 5562 H11R POPCO 12 -27.500 -3.574 -4.383 1.00 0.00 O2 H

ATOM 5563 H11S POPCO 12 -28.608 -2.937 -3.166 1.00 0.00 O2 H

ATOM 5564 C212 POPCO 12 -27.039 -3.711 -2.206 1.00 0.00 O2 C

ATOM 5565 H12R POPCO 12 -26.134 -4.300 -2.366 1.00 0.00 O2 H

ATOM 5566 H12S POPCO 12 -26.596 -2.678 -2.028 1.00 0.00 O2 H

ATOM 5567 C213 POPCO 12 -27.683 -3.985 -0.907 1.00 0.00 O2 C

ATOM 5568 H13R POPCO 12 -28.474 -3.132 -0.845 1.00 0.00 O2 H

ATOM 5569 H13S POPCO 12 -28.158 -4.991 -0.842 1.00 0.00 O2 H

ATOM 5570 C214 POPCO 12 -26.834 -3.747 0.321 1.00 0.00 O2 C

ATOM 5571 H14R POPCO 12 -25.940 -4.396 0.400 1.00 0.00 O2 H

ATOM 5572 H14S POPCO 12 -26.516 -2.665 0.349 1.00 0.00 O2 H

ATOM 5573 C215 POPCO 12 -27.604 -3.979 1.609 1.00 0.00 O2 C

ATOM 5574 H15R POPCO 12 -28.632 -3.546 1.550 1.00 0.00 O2 H

ATOM 5575 H15S POPCO 12 -27.859 -5.043 1.921 1.00 0.00 O2 H

ATOM 5576 C216 POPCO 12 -26.965 -3.253 2.758 1.00 0.00 O2 C

ATOM 5577 H16R POPCO 12 -25.866 -3.484 2.644 1.00 0.00 O2 H

ATOM 5578 H16S POPCO 12 -27.147 -2.215 2.544 1.00 0.00 O2 H

ATOM 5579 C217 POPCO 12 -27.610 -3.707 4.052 1.00 0.00 O2 C

ATOM 5580 H17R POPCO 12 -28.522 -3.074 4.044 1.00 0.00 O2 H

ATOM 5581 H17S POPCO 12 -27.896 -4.768 4.151 1.00 0.00 O2 H

ATOM 5582 C218 POPCO 12 -26.678 -3.498 5.255 1.00 0.00 O2 C

ATOM 5583 H18R POPCO 12 -27.160 -3.814 6.184 1.00 0.00 O2 H

ATOM 5584 H18S POPCO 12 -25.813 -4.158 5.266 1.00 0.00 O2 H

ATOM 5585 H18T POPCO 12 -26.454 -2.390 5.338 1.00 0.00 O2 H

ATOM 5586 C33 POPCO 12 -27.420 -1.583 -15.121 1.00 0.00 O2 C

ATOM 5587 H3X POPCO 12 -26.767 -2.004 -14.361 1.00 0.00 O2 H

ATOM 5588 H3Y POPCO 12 -26.890 -0.597 -15.475 1.00 0.00 O2 H

ATOM 5589 C34 POPCO 12 -28.747 -1.127 -14.465 1.00 0.00 O2 C

ATOM 5590 H4X POPCO 12 -29.517 -0.715 -15.117 1.00 0.00 O2 H

ATOM 5591 H4Y POPCO 12 -29.215 -2.045 -14.054 1.00 0.00 O2 H

ATOM 5592 C35 POPCO 12 -28.549 -0.022 -13.347 1.00 0.00 O2 C

ATOM 5593 H5X POPCO 12 -27.664 -0.367 -12.808 1.00 0.00 O2 H

ATOM 5594 H5Y POPCO 12 -28.277 0.934 -13.897 1.00 0.00 O2 H

ATOM 5595 C36 POPCO 12 -29.679 0.023 -12.337 1.00 0.00 O2 C

ATOM 5596 H6X POPCO 12 -30.705 0.251 -12.592 1.00 0.00 O2 H

ATOM 5597 H6Y POPCO 12 -29.865 -1.029 -12.040 1.00 0.00 O2 H

ATOM 5598 C37 POPCO 12 -29.324 0.885 -11.095 1.00 0.00 O2 C

ATOM 5599 H7X POPCO 12 -28.208 0.905 -10.887 1.00 0.00 O2 H

ATOM 5600 H7Y POPCO 12 -29.528 1.974 -11.332 1.00 0.00 O2 H

ATOM 5601 C38 POPCO 12 -30.023 0.421 -9.810 1.00 0.00 O2 C

ATOM 5602 H8X POPCO 12 -31.138 0.294 -10.130 1.00 0.00 O2 H

ATOM 5603 H8Y POPCO 12 -29.628 -0.575 -9.569 1.00 0.00 O2 H

ATOM 5604 C39 POPCO 12 -29.858 1.355 -8.622 1.00 0.00 O2 C

ATOM 5605 H9X POPCO 12 -29.695 2.462 -8.722 1.00 0.00 O2 H

ATOM 5606 H9Y POPCO 12 -30.852 1.460 -8.139 1.00 0.00 O2 H

ATOM 5607 C310 POPCO 12 -28.977 0.687 -7.540 1.00 0.00 O2 C

ATOM 5608 H10X POPCO 12 -29.182 -0.362 -7.659 1.00 0.00 O2 H

ATOM 5609 H10Y POPCO 12 -27.912 0.765 -7.763 1.00 0.00 O2 H

ATOM 5610 C311 POPCO 12 -29.278 1.171 -6.085 1.00 0.00 O2 C

ATOM 5611 H11X POPCO 12 -29.113 2.307 -6.001 1.00 0.00 O2 H

ATOM 5612 H11Y POPCO 12 -30.344 1.149 -5.965 1.00 0.00 O2 H

ATOM 5613 C312 POPCO 12 -28.531 0.349 -4.967 1.00 0.00 O2 C

ATOM 5614 H12X POPCO 12 -28.806 -0.686 -5.101 1.00 0.00 O2 H

ATOM 5615 H12Y POPCO 12 -27.503 0.352 -5.208 1.00 0.00 O2 H

ATOM 5616 C313 POPCO 12 -28.692 0.784 -3.548 1.00 0.00 O2 C

ATOM 5617 H13X POPCO 12 -28.497 1.868 -3.338 1.00 0.00 O2 H

ATOM 5618 H13Y POPCO 12 -29.756 0.454 -3.238 1.00 0.00 O2 H

ATOM 5619 C314 POPCO 12 -27.682 0.038 -2.608 1.00 0.00 O2 C

ATOM 5620 H14X POPCO 12 -28.242 -0.891 -2.368 1.00 0.00 O2 H

ATOM 5621 H14Y POPCO 12 -26.705 -0.183 -3.075 1.00 0.00 O2 H

ATOM 5622 C315 POPCO 12 -27.535 0.587 -1.190 1.00 0.00 O2 C

ATOM 5623 H15X POPCO 12 -26.661 0.155 -0.696 1.00 0.00 O2 H

ATOM 5624 H15Y POPCO 12 -27.472 1.679 -1.378 1.00 0.00 O2 H

ATOM 5625 C316 POPCO 12 -28.681 0.262 -0.209 1.00 0.00 O2 C

ATOM 5626 H16X POPCO 12 -28.860 -0.842 -0.308 1.00 0.00 O2 H

ATOM 5627 H16Y POPCO 12 -28.484 0.619 0.785 1.00 0.00 O2 H

ATOM 5628 H16Z POPCO 12 -29.603 0.792 -0.468 1.00 0.00 O2 H

ATOM 5629 N POPCO 13 -27.526 -39.249 -22.444 1.00 0.00 O2 N

ATOM 5630 C12 POPCO 13 -27.822 -39.619 -20.992 1.00 0.00 O2 C

ATOM 5631 H12A POPCO 13 -28.732 -39.114 -20.586 1.00 0.00 O2 H

ATOM 5632 H12B POPCO 13 -27.178 -39.012 -20.376 1.00 0.00 O2 H

ATOM 5633 C13 POPCO 13 -28.752 -39.514 -23.209 1.00 0.00 O2 C

ATOM 5634 H13A POPCO 13 -29.624 -39.079 -22.768 1.00 0.00 O2 H

ATOM 5635 H13B POPCO 13 -28.977 -40.510 -23.217 1.00 0.00 O2 H

ATOM 5636 H13C POPCO 13 -28.637 -39.115 -24.242 1.00 0.00 O2 H

ATOM 5637 C14 POPCO 13 -27.215 -37.799 -22.613 1.00 0.00 O2 C

ATOM 5638 H14A POPCO 13 -26.395 -37.505 -21.980 1.00 0.00 O2 H

ATOM 5639 H14B POPCO 13 -28.077 -37.243 -22.285 1.00 0.00 O2 H

ATOM 5640 H14C POPCO 13 -26.946 -37.481 -23.619 1.00 0.00 O2 H

ATOM 5641 C15 POPCO 13 -26.340 -40.012 -22.980 1.00 0.00 O2 C

ATOM 5642 H15A POPCO 13 -25.430 -39.828 -22.357 1.00 0.00 O2 H

ATOM 5643 H15B POPCO 13 -26.652 -41.079 -22.778 1.00 0.00 O2 H

ATOM 5644 H15C POPCO 13 -26.161 -39.885 -24.027 1.00 0.00 O2 H

ATOM 5645 C11 POPCO 13 -27.683 -41.101 -20.693 1.00 0.00 O2 C

ATOM 5646 H11A POPCO 13 -26.621 -41.417 -20.908 1.00 0.00 O2 H

ATOM 5647 H11B POPCO 13 -27.902 -41.232 -19.569 1.00 0.00 O2 H

ATOM 5648 P POPCO 13 -28.394 -43.340 -21.998 1.00 0.00 O2 P

ATOM 5649 O13 POPCO 13 -28.119 -43.218 -23.459 1.00 0.00 O2 O

ATOM 5650 O14 POPCO 13 -29.466 -44.244 -21.538 1.00 0.00 O2 O

ATOM 5651 O12 POPCO 13 -28.690 -41.831 -21.451 1.00 0.00 O2 O

ATOM 5652 O11 POPCO 13 -27.078 -43.842 -21.214 1.00 0.00 O2 O

ATOM 5653 C1 POPCO 13 -27.101 -43.971 -19.780 1.00 0.00 O2 C

ATOM 5654 HA POPCO 13 -27.532 -43.163 -19.069 1.00 0.00 O2 H

ATOM 5655 HB POPCO 13 -27.652 -44.878 -19.620 1.00 0.00 O2 H

ATOM 5656 C2 POPCO 13 -25.625 -44.319 -19.199 1.00 0.00 O2 C

ATOM 5657 HS POPCO 13 -25.397 -45.279 -19.517 1.00 0.00 O2 H

ATOM 5658 O21 POPCO 13 -25.702 -44.438 -17.747 1.00 0.00 O2 O

ATOM 5659 C21 POPCO 13 -24.660 -44.716 -17.021 1.00 0.00 O2 C

ATOM 5660 O22 POPCO 13 -23.600 -45.093 -17.399 1.00 0.00 O2 O

ATOM 5661 C22 POPCO 13 -25.094 -44.400 -15.547 1.00 0.00 O2 C

ATOM 5662 H2R POPCO 13 -25.340 -43.351 -15.593 1.00 0.00 O2 H

ATOM 5663 H2S POPCO 13 -25.984 -44.982 -15.396 1.00 0.00 O2 H

ATOM 5664 C3 POPCO 13 -24.548 -43.302 -19.731 1.00 0.00 O2 C

ATOM 5665 HX POPCO 13 -23.563 -43.720 -19.546 1.00 0.00 O2 H

ATOM 5666 HY POPCO 13 -24.808 -43.294 -20.776 1.00 0.00 O2 H

ATOM 5667 O31 POPCO 13 -24.672 -41.993 -19.125 1.00 0.00 O2 O

ATOM 5668 C31 POPCO 13 -23.891 -41.057 -19.665 1.00 0.00 O2 C

ATOM 5669 O32 POPCO 13 -22.937 -41.194 -20.431 1.00 0.00 O2 O

ATOM 5670 C32 POPCO 13 -24.488 -39.639 -19.283 1.00 0.00 O2 C

ATOM 5671 H2X POPCO 13 -24.409 -39.026 -20.200 1.00 0.00 O2 H

ATOM 5672 H2Y POPCO 13 -25.567 -39.782 -18.965 1.00 0.00 O2 H

ATOM 5673 C23 POPCO 13 -24.027 -44.836 -14.445 1.00 0.00 O2 C

ATOM 5674 H3R POPCO 13 -24.201 -45.923 -14.300 1.00 0.00 O2 H

ATOM 5675 H3S POPCO 13 -22.966 -44.731 -14.567 1.00 0.00 O2 H

ATOM 5676 C24 POPCO 13 -24.299 -44.063 -13.128 1.00 0.00 O2 C

ATOM 5677 H4R POPCO 13 -24.011 -42.992 -13.206 1.00 0.00 O2 H

ATOM 5678 H4S POPCO 13 -25.385 -44.183 -12.956 1.00 0.00 O2 H

ATOM 5679 C25 POPCO 13 -23.562 -44.777 -11.912 1.00 0.00 O2 C

ATOM 5680 H5R POPCO 13 -23.438 -45.823 -12.121 1.00 0.00 O2 H

ATOM 5681 H5S POPCO 13 -22.499 -44.332 -11.884 1.00 0.00 O2 H

ATOM 5682 C26 POPCO 13 -24.319 -44.505 -10.639 1.00 0.00 O2 C

ATOM 5683 H6R POPCO 13 -24.312 -43.351 -10.394 1.00 0.00 O2 H

ATOM 5684 H6S POPCO 13 -25.348 -44.856 -10.766 1.00 0.00 O2 H

ATOM 5685 C27 POPCO 13 -23.876 -45.272 -9.422 1.00 0.00 O2 C

ATOM 5686 H7R POPCO 13 -23.447 -46.233 -9.898 1.00 0.00 O2 H

ATOM 5687 H7S POPCO 13 -23.182 -44.569 -8.926 1.00 0.00 O2 H

ATOM 5688 C28 POPCO 13 -25.039 -45.606 -8.480 1.00 0.00 O2 C

ATOM 5689 H8R POPCO 13 -25.647 -44.714 -8.265 1.00 0.00 O2 H

ATOM 5690 H8S POPCO 13 -25.738 -46.313 -9.076 1.00 0.00 O2 H

ATOM 5691 C29 POPCO 13 -24.493 -46.376 -7.228 1.00 0.00 O2 C

ATOM 5692 H91 POPCO 13 -23.894 -47.310 -7.375 1.00 0.00 O2 H

ATOM 5693 C210 POPCO 13 -24.707 -46.042 -5.894 1.00 0.00 O2 C

ATOM 5694 H101 POPCO 13 -24.260 -46.695 -5.109 1.00 0.00 O2 H

ATOM 5695 C211 POPCO 13 -25.594 -44.904 -5.412 1.00 0.00 O2 C

ATOM 5696 H11R POPCO 13 -25.796 -44.230 -6.359 1.00 0.00 O2 H

ATOM 5697 H11S POPCO 13 -26.610 -45.258 -5.157 1.00 0.00 O2 H

ATOM 5698 C212 POPCO 13 -24.955 -43.986 -4.353 1.00 0.00 O2 C

ATOM 5699 H12R POPCO 13 -24.036 -43.537 -4.856 1.00 0.00 O2 H

ATOM 5700 H12S POPCO 13 -25.730 -43.169 -4.157 1.00 0.00 O2 H

ATOM 5701 C213 POPCO 13 -24.642 -44.702 -3.020 1.00 0.00 O2 C

ATOM 5702 H13R POPCO 13 -25.514 -45.392 -2.732 1.00 0.00 O2 H

ATOM 5703 H13S POPCO 13 -23.708 -45.332 -3.193 1.00 0.00 O2 H

ATOM 5704 C214 POPCO 13 -24.117 -43.715 -1.985 1.00 0.00 O2 C

ATOM 5705 H14R POPCO 13 -23.205 -43.319 -2.390 1.00 0.00 O2 H

ATOM 5706 H14S POPCO 13 -24.797 -42.867 -1.780 1.00 0.00 O2 H

ATOM 5707 C215 POPCO 13 -23.745 -44.344 -0.564 1.00 0.00 O2 C

ATOM 5708 H15R POPCO 13 -24.603 -44.997 -0.281 1.00 0.00 O2 H

ATOM 5709 H15S POPCO 13 -22.759 -44.914 -0.645 1.00 0.00 O2 H

ATOM 5710 C216 POPCO 13 -23.538 -43.202 0.524 1.00 0.00 O2 C

ATOM 5711 H16R POPCO 13 -22.781 -42.482 0.158 1.00 0.00 O2 H

ATOM 5712 H16S POPCO 13 -24.398 -42.533 0.538 1.00 0.00 O2 H

ATOM 5713 C217 POPCO 13 -23.258 -43.715 1.905 1.00 0.00 O2 C

ATOM 5714 H17R POPCO 13 -24.061 -44.437 2.198 1.00 0.00 O2 H

ATOM 5715 H17S POPCO 13 -22.326 -44.379 1.949 1.00 0.00 O2 H

ATOM 5716 C218 POPCO 13 -23.167 -42.702 3.030 1.00 0.00 O2 C

ATOM 5717 H18R POPCO 13 -22.798 -43.126 3.980 1.00 0.00 O2 H

ATOM 5718 H18S POPCO 13 -22.718 -41.799 2.651 1.00 0.00 O2 H

ATOM 5719 H18T POPCO 13 -24.223 -42.492 3.302 1.00 0.00 O2 H

ATOM 5720 C33 POPCO 13 -23.924 -39.005 -18.010 1.00 0.00 O2 C

ATOM 5721 H3X POPCO 13 -22.817 -38.894 -18.034 1.00 0.00 O2 H

ATOM 5722 H3Y POPCO 13 -24.470 -37.987 -18.045 1.00 0.00 O2 H

ATOM 5723 C34 POPCO 13 -24.225 -39.669 -16.667 1.00 0.00 O2 C

ATOM 5724 H4X POPCO 13 -25.335 -39.929 -16.737 1.00 0.00 O2 H

ATOM 5725 H4Y POPCO 13 -23.557 -40.571 -16.561 1.00 0.00 O2 H

ATOM 5726 C35 POPCO 13 -23.865 -38.942 -15.338 1.00 0.00 O2 C

ATOM 5727 H5X POPCO 13 -22.742 -38.881 -15.360 1.00 0.00 O2 H

ATOM 5728 H5Y POPCO 13 -24.403 -37.957 -15.245 1.00 0.00 O2 H

ATOM 5729 C36 POPCO 13 -24.230 -39.828 -14.175 1.00 0.00 O2 C

ATOM 5730 H6X POPCO 13 -25.335 -39.922 -14.102 1.00 0.00 O2 H

ATOM 5731 H6Y POPCO 13 -23.854 -40.845 -14.137 1.00 0.00 O2 H

ATOM 5732 C37 POPCO 13 -23.715 -39.284 -12.853 1.00 0.00 O2 C

ATOM 5733 H7X POPCO 13 -22.612 -39.196 -12.966 1.00 0.00 O2 H

ATOM 5734 H7Y POPCO 13 -24.136 -38.317 -12.605 1.00 0.00 O2 H

ATOM 5735 C38 POPCO 13 -23.940 -40.195 -11.649 1.00 0.00 O2 C

ATOM 5736 H8X POPCO 13 -25.063 -40.351 -11.570 1.00 0.00 O2 H

ATOM 5737 H8Y POPCO 13 -23.391 -41.132 -11.824 1.00 0.00 O2 H

ATOM 5738 C39 POPCO 13 -23.472 -39.625 -10.330 1.00 0.00 O2 C

ATOM 5739 H9X POPCO 13 -22.365 -39.561 -10.375 1.00 0.00 O2 H

ATOM 5740 H9Y POPCO 13 -23.808 -38.580 -10.217 1.00 0.00 O2 H

ATOM 5741 C310 POPCO 13 -23.841 -40.450 -9.135 1.00 0.00 O2 C

ATOM 5742 H10X POPCO 13 -24.929 -40.483 -9.137 1.00 0.00 O2 H

ATOM 5743 H10Y POPCO 13 -23.469 -41.416 -9.165 1.00 0.00 O2 H

ATOM 5744 C311 POPCO 13 -23.326 -39.822 -7.842 1.00 0.00 O2 C

ATOM 5745 H11X POPCO 13 -22.183 -39.894 -7.817 1.00 0.00 O2 H

ATOM 5746 H11Y POPCO 13 -23.742 -38.811 -7.710 1.00 0.00 O2 H

ATOM 5747 C312 POPCO 13 -23.696 -40.550 -6.561 1.00 0.00 O2 C

ATOM 5748 H12X POPCO 13 -24.807 -40.701 -6.436 1.00 0.00 O2 H

ATOM 5749 H12Y POPCO 13 -23.071 -41.516 -6.537 1.00 0.00 O2 H

ATOM 5750 C313 POPCO 13 -23.214 -39.784 -5.344 1.00 0.00 O2 C

ATOM 5751 H13X POPCO 13 -22.124 -39.551 -5.562 1.00 0.00 O2 H

ATOM 5752 H13Y POPCO 13 -23.816 -38.857 -5.131 1.00 0.00 O2 H

ATOM 5753 C314 POPCO 13 -23.173 -40.565 -4.051 1.00 0.00 O2 C

ATOM 5754 H14X POPCO 13 -24.116 -40.811 -3.695 1.00 0.00 O2 H

ATOM 5755 H14Y POPCO 13 -22.796 -41.575 -4.269 1.00 0.00 O2 H

ATOM 5756 C315 POPCO 13 -22.402 -39.761 -2.976 1.00 0.00 O2 C

ATOM 5757 H15X POPCO 13 -21.485 -39.380 -3.452 1.00 0.00 O2 H

ATOM 5758 H15Y POPCO 13 -23.021 -38.903 -2.735 1.00 0.00 O2 H

ATOM 5759 C316 POPCO 13 -22.152 -40.413 -1.611 1.00 0.00 O2 C

ATOM 5760 H16X POPCO 13 -21.673 -41.388 -1.557 1.00 0.00 O2 H

ATOM 5761 H16Y POPCO 13 -21.675 -39.716 -0.862 1.00 0.00 O2 H

ATOM 5762 H16Z POPCO 13 -23.170 -40.590 -1.269 1.00 0.00 O2 H

ATOM 5763 N POPCO 14 -19.755 -34.358 -24.369 1.00 0.00 O2 N

ATOM 5764 C12 POPCO 14 -18.877 -35.236 -23.435 1.00 0.00 O2 C

ATOM 5765 H12A POPCO 14 -18.385 -34.518 -22.746 1.00 0.00 O2 H

ATOM 5766 H12B POPCO 14 -18.182 -35.675 -24.083 1.00 0.00 O2 H

ATOM 5767 C13 POPCO 14 -20.602 -33.451 -23.595 1.00 0.00 O2 C

ATOM 5768 H13A POPCO 14 -20.095 -33.388 -22.633 1.00 0.00 O2 H

ATOM 5769 H13B POPCO 14 -21.516 -33.929 -23.177 1.00 0.00 O2 H

ATOM 5770 H13C POPCO 14 -20.986 -32.485 -24.070 1.00 0.00 O2 H

ATOM 5771 C14 POPCO 14 -18.923 -33.636 -25.340 1.00 0.00 O2 C

ATOM 5772 H14A POPCO 14 -18.396 -34.341 -25.927 1.00 0.00 O2 H

ATOM 5773 H14B POPCO 14 -18.313 -32.988 -24.798 1.00 0.00 O2 H

ATOM 5774 H14C POPCO 14 -19.518 -32.996 -26.021 1.00 0.00 O2 H

ATOM 5775 C15 POPCO 14 -20.682 -35.274 -25.041 1.00 0.00 O2 C

ATOM 5776 H15A POPCO 14 -20.243 -35.882 -25.789 1.00 0.00 O2 H

ATOM 5777 H15B POPCO 14 -21.296 -35.892 -24.378 1.00 0.00 O2 H

ATOM 5778 H15C POPCO 14 -21.467 -34.659 -25.577 1.00 0.00 O2 H

ATOM 5779 C11 POPCO 14 -19.569 -36.362 -22.561 1.00 0.00 O2 C

ATOM 5780 H11A POPCO 14 -19.927 -37.088 -23.269 1.00 0.00 O2 H

ATOM 5781 H11B POPCO 14 -18.860 -36.888 -21.883 1.00 0.00 O2 H

ATOM 5782 P POPCO 14 -22.139 -36.035 -21.801 1.00 0.00 O2 P

ATOM 5783 O13 POPCO 14 -22.532 -36.836 -22.961 1.00 0.00 O2 O

ATOM 5784 O14 POPCO 14 -22.722 -34.696 -21.828 1.00 0.00 O2 O

ATOM 5785 O12 POPCO 14 -20.572 -35.776 -21.672 1.00 0.00 O2 O

ATOM 5786 O11 POPCO 14 -22.318 -36.783 -20.470 1.00 0.00 O2 O

ATOM 5787 C1 POPCO 14 -22.371 -36.084 -19.168 1.00 0.00 O2 C

ATOM 5788 HA POPCO 14 -22.168 -35.004 -19.276 1.00 0.00 O2 H

ATOM 5789 HB POPCO 14 -23.378 -36.316 -18.720 1.00 0.00 O2 H

ATOM 5790 C2 POPCO 14 -21.293 -36.493 -18.089 1.00 0.00 O2 C

ATOM 5791 HS POPCO 14 -21.246 -37.568 -18.142 1.00 0.00 O2 H

ATOM 5792 O21 POPCO 14 -21.741 -36.052 -16.784 1.00 0.00 O2 O

ATOM 5793 C21 POPCO 14 -21.137 -36.582 -15.726 1.00 0.00 O2 C

ATOM 5794 O22 POPCO 14 -20.219 -37.356 -15.743 1.00 0.00 O2 O

ATOM 5795 C22 POPCO 14 -21.797 -36.037 -14.477 1.00 0.00 O2 C

ATOM 5796 H2R POPCO 14 -21.561 -34.927 -14.445 1.00 0.00 O2 H

ATOM 5797 H2S POPCO 14 -22.880 -36.278 -14.370 1.00 0.00 O2 H

ATOM 5798 C3 POPCO 14 -19.889 -35.863 -18.499 1.00 0.00 O2 C

ATOM 5799 HX POPCO 14 -19.130 -36.312 -17.789 1.00 0.00 O2 H

ATOM 5800 HY POPCO 14 -19.775 -36.267 -19.555 1.00 0.00 O2 H

ATOM 5801 O31 POPCO 14 -19.793 -34.421 -18.489 1.00 0.00 O2 O

ATOM 5802 C31 POPCO 14 -18.641 -33.947 -18.783 1.00 0.00 O2 C

ATOM 5803 O32 POPCO 14 -17.757 -34.463 -19.444 1.00 0.00 O2 O

ATOM 5804 C32 POPCO 14 -18.413 -32.506 -18.244 1.00 0.00 O2 C

ATOM 5805 H2X POPCO 14 -17.420 -32.268 -18.045 1.00 0.00 O2 H

ATOM 5806 H2Y POPCO 14 -18.650 -31.730 -19.013 1.00 0.00 O2 H

ATOM 5807 C23 POPCO 14 -21.044 -36.543 -13.169 1.00 0.00 O2 C

ATOM 5808 H3R POPCO 14 -21.217 -37.654 -13.161 1.00 0.00 O2 H

ATOM 5809 H3S POPCO 14 -19.904 -36.338 -13.198 1.00 0.00 O2 H

ATOM 5810 C24 POPCO 14 -21.554 -36.034 -11.837 1.00 0.00 O2 C

ATOM 5811 H4R POPCO 14 -21.332 -34.935 -11.861 1.00 0.00 O2 H

ATOM 5812 H4S POPCO 14 -22.612 -36.308 -11.823 1.00 0.00 O2 H

ATOM 5813 C25 POPCO 14 -20.730 -36.612 -10.711 1.00 0.00 O2 C

ATOM 5814 H5R POPCO 14 -20.691 -37.772 -10.700 1.00 0.00 O2 H

ATOM 5815 H5S POPCO 14 -19.675 -36.276 -10.870 1.00 0.00 O2 H

ATOM 5816 C26 POPCO 14 -21.151 -36.114 -9.310 1.00 0.00 O2 C

ATOM 5817 H6R POPCO 14 -21.189 -35.019 -9.187 1.00 0.00 O2 H

ATOM 5818 H6S POPCO 14 -22.179 -36.590 -9.172 1.00 0.00 O2 H

ATOM 5819 C27 POPCO 14 -20.380 -36.746 -8.120 1.00 0.00 O2 C

ATOM 5820 H7R POPCO 14 -20.264 -37.887 -8.440 1.00 0.00 O2 H

ATOM 5821 H7S POPCO 14 -19.331 -36.255 -7.974 1.00 0.00 O2 H

ATOM 5822 C28 POPCO 14 -21.145 -36.704 -6.793 1.00 0.00 O2 C

ATOM 5823 H8R POPCO 14 -21.230 -35.665 -6.481 1.00 0.00 O2 H

ATOM 5824 H8S POPCO 14 -22.195 -37.028 -6.987 1.00 0.00 O2 H

ATOM 5825 C29 POPCO 14 -20.399 -37.575 -5.808 1.00 0.00 O2 C

ATOM 5826 H91 POPCO 14 -20.228 -38.574 -6.117 1.00 0.00 O2 H

ATOM 5827 C210 POPCO 14 -19.945 -37.243 -4.606 1.00 0.00 O2 C

ATOM 5828 H101 POPCO 14 -19.319 -37.833 -3.982 1.00 0.00 O2 H

ATOM 5829 C211 POPCO 14 -20.235 -35.867 -3.941 1.00 0.00 O2 C

ATOM 5830 H11R POPCO 14 -19.722 -34.957 -4.433 1.00 0.00 O2 H

ATOM 5831 H11S POPCO 14 -21.258 -35.608 -3.935 1.00 0.00 O2 H

ATOM 5832 C212 POPCO 14 -19.842 -35.744 -2.449 1.00 0.00 O2 C

ATOM 5833 H12R POPCO 14 -18.780 -35.644 -2.279 1.00 0.00 O2 H

ATOM 5834 H12S POPCO 14 -20.246 -34.787 -2.040 1.00 0.00 O2 H

ATOM 5835 C213 POPCO 14 -20.482 -36.804 -1.487 1.00 0.00 O2 C

ATOM 5836 H13R POPCO 14 -21.490 -36.532 -1.718 1.00 0.00 O2 H

ATOM 5837 H13S POPCO 14 -20.199 -37.806 -1.796 1.00 0.00 O2 H

ATOM 5838 C214 POPCO 14 -20.250 -36.523 -0.022 1.00 0.00 O2 C

ATOM 5839 H14R POPCO 14 -19.148 -36.644 0.168 1.00 0.00 O2 H

ATOM 5840 H14S POPCO 14 -20.617 -35.515 0.283 1.00 0.00 O2 H

ATOM 5841 C215 POPCO 14 -20.933 -37.469 0.927 1.00 0.00 O2 C

ATOM 5842 H15R POPCO 14 -22.024 -37.382 0.705 1.00 0.00 O2 H

ATOM 5843 H15S POPCO 14 -20.615 -38.508 0.803 1.00 0.00 O2 H

ATOM 5844 C216 POPCO 14 -20.645 -36.917 2.323 1.00 0.00 O2 C

ATOM 5845 H16R POPCO 14 -19.625 -36.982 2.616 1.00 0.00 O2 H

ATOM 5846 H16S POPCO 14 -20.879 -35.837 2.159 1.00 0.00 O2 H

ATOM 5847 C217 POPCO 14 -21.563 -37.590 3.353 1.00 0.00 O2 C

ATOM 5848 H17R POPCO 14 -22.616 -37.433 3.029 1.00 0.00 O2 H

ATOM 5849 H17S POPCO 14 -21.294 -38.673 3.333 1.00 0.00 O2 H

ATOM 5850 C218 POPCO 14 -21.366 -37.028 4.739 1.00 0.00 O2 C

ATOM 5851 H18R POPCO 14 -21.994 -37.539 5.442 1.00 0.00 O2 H

ATOM 5852 H18S POPCO 14 -20.275 -37.180 5.025 1.00 0.00 O2 H

ATOM 5853 H18T POPCO 14 -21.694 -35.996 4.803 1.00 0.00 O2 H

ATOM 5854 C33 POPCO 14 -19.203 -32.121 -16.996 1.00 0.00 O2 C

ATOM 5855 H3X POPCO 14 -18.929 -31.118 -16.759 1.00 0.00 O2 H

ATOM 5856 H3Y POPCO 14 -20.315 -31.997 -17.141 1.00 0.00 O2 H

ATOM 5857 C34 POPCO 14 -18.813 -33.001 -15.828 1.00 0.00 O2 C

ATOM 5858 H4X POPCO 14 -19.072 -34.086 -16.078 1.00 0.00 O2 H

ATOM 5859 H4Y POPCO 14 -17.674 -32.928 -15.785 1.00 0.00 O2 H

ATOM 5860 C35 POPCO 14 -19.424 -32.513 -14.573 1.00 0.00 O2 C

ATOM 5861 H5X POPCO 14 -19.073 -31.435 -14.533 1.00 0.00 O2 H

ATOM 5862 H5Y POPCO 14 -20.495 -32.588 -14.525 1.00 0.00 O2 H

ATOM 5863 C36 POPCO 14 -18.841 -33.226 -13.451 1.00 0.00 O2 C

ATOM 5864 H6X POPCO 14 -19.262 -34.285 -13.410 1.00 0.00 O2 H

ATOM 5865 H6Y POPCO 14 -17.759 -33.337 -13.585 1.00 0.00 O2 H

ATOM 5866 C37 POPCO 14 -19.027 -32.431 -12.060 1.00 0.00 O2 C

ATOM 5867 H7X POPCO 14 -18.253 -31.659 -12.165 1.00 0.00 O2 H

ATOM 5868 H7Y POPCO 14 -19.991 -31.955 -12.232 1.00 0.00 O2 H

ATOM 5869 C38 POPCO 14 -18.836 -33.246 -10.852 1.00 0.00 O2 C

ATOM 5870 H8X POPCO 14 -19.763 -33.837 -10.678 1.00 0.00 O2 H

ATOM 5871 H8Y POPCO 14 -18.025 -33.965 -10.885 1.00 0.00 O2 H

ATOM 5872 C39 POPCO 14 -18.544 -32.312 -9.709 1.00 0.00 O2 C

ATOM 5873 H9X POPCO 14 -17.649 -31.591 -9.866 1.00 0.00 O2 H

ATOM 5874 H9Y POPCO 14 -19.424 -31.607 -9.547 1.00 0.00 O2 H

ATOM 5875 C310 POPCO 14 -18.121 -33.226 -8.437 1.00 0.00 O2 C

ATOM 5876 H10X POPCO 14 -19.161 -33.755 -8.266 1.00 0.00 O2 H

ATOM 5877 H10Y POPCO 14 -17.483 -34.073 -8.530 1.00 0.00 O2 H

ATOM 5878 C311 POPCO 14 -17.637 -32.338 -7.226 1.00 0.00 O2 C

ATOM 5879 H11X POPCO 14 -16.717 -31.853 -7.664 1.00 0.00 O2 H

ATOM 5880 H11Y POPCO 14 -18.468 -31.588 -7.108 1.00 0.00 O2 H

ATOM 5881 C312 POPCO 14 -17.375 -33.201 -5.941 1.00 0.00 O2 C

ATOM 5882 H12X POPCO 14 -18.317 -33.851 -5.856 1.00 0.00 O2 H

ATOM 5883 H12Y POPCO 14 -16.484 -33.920 -6.113 1.00 0.00 O2 H

ATOM 5884 C313 POPCO 14 -17.299 -32.326 -4.651 1.00 0.00 O2 C

ATOM 5885 H13X POPCO 14 -16.341 -31.786 -4.567 1.00 0.00 O2 H

ATOM 5886 H13Y POPCO 14 -18.055 -31.517 -4.862 1.00 0.00 O2 H

ATOM 5887 C314 POPCO 14 -17.558 -33.077 -3.374 1.00 0.00 O2 C

ATOM 5888 H14X POPCO 14 -18.587 -33.475 -3.301 1.00 0.00 O2 H

ATOM 5889 H14Y POPCO 14 -17.013 -34.048 -3.359 1.00 0.00 O2 H

ATOM 5890 C315 POPCO 14 -17.223 -32.266 -2.077 1.00 0.00 O2 C

ATOM 5891 H15X POPCO 14 -16.121 -31.974 -2.071 1.00 0.00 O2 H

ATOM 5892 H15Y POPCO 14 -17.740 -31.347 -2.037 1.00 0.00 O2 H

ATOM 5893 C316 POPCO 14 -17.545 -33.072 -0.853 1.00 0.00 O2 C

ATOM 5894 H16X POPCO 14 -16.873 -33.907 -0.847 1.00 0.00 O2 H

ATOM 5895 H16Y POPCO 14 -17.308 -32.488 0.048 1.00 0.00 O2 H

ATOM 5896 H16Z POPCO 14 -18.537 -33.462 -0.858 1.00 0.00 O2 H

ATOM 5897 N POPCO 15 -28.010 -24.182 -20.914 1.00 0.00 O2 N

ATOM 5898 C12 POPCO 15 -28.153 -23.787 -19.432 1.00 0.00 O2 C

ATOM 5899 H12A POPCO 15 -28.936 -23.080 -19.356 1.00 0.00 O2 H

ATOM 5900 H12B POPCO 15 -27.330 -23.136 -19.102 1.00 0.00 O2 H

ATOM 5901 C13 POPCO 15 -29.218 -24.735 -21.450 1.00 0.00 O2 C

ATOM 5902 H13A POPCO 15 -30.051 -24.059 -21.364 1.00 0.00 O2 H

ATOM 5903 H13B POPCO 15 -29.569 -25.648 -20.964 1.00 0.00 O2 H

ATOM 5904 H13C POPCO 15 -29.083 -24.998 -22.516 1.00 0.00 O2 H

ATOM 5905 C14 POPCO 15 -27.444 -23.074 -21.710 1.00 0.00 O2 C

ATOM 5906 H14A POPCO 15 -26.517 -22.784 -21.162 1.00 0.00 O2 H

ATOM 5907 H14B POPCO 15 -28.140 -22.198 -21.588 1.00 0.00 O2 H

ATOM 5908 H14C POPCO 15 -27.301 -23.395 -22.679 1.00 0.00 O2 H

ATOM 5909 C15 POPCO 15 -27.073 -25.289 -20.969 1.00 0.00 O2 C

ATOM 5910 H15A POPCO 15 -26.276 -25.139 -20.305 1.00 0.00 O2 H

ATOM 5911 H15B POPCO 15 -27.717 -26.177 -20.603 1.00 0.00 O2 H

ATOM 5912 H15C POPCO 15 -26.824 -25.549 -21.982 1.00 0.00 O2 H

ATOM 5913 C11 POPCO 15 -28.322 -24.850 -18.385 1.00 0.00 O2 C

ATOM 5914 H11A POPCO 15 -27.406 -25.426 -18.395 1.00 0.00 O2 H

ATOM 5915 H11B POPCO 15 -28.437 -24.362 -17.427 1.00 0.00 O2 H

ATOM 5916 P POPCO 15 -29.611 -27.125 -18.872 1.00 0.00 O2 P

ATOM 5917 O13 POPCO 15 -29.375 -27.423 -20.329 1.00 0.00 O2 O

ATOM 5918 O14 POPCO 15 -30.879 -27.642 -18.385 1.00 0.00 O2 O

ATOM 5919 O12 POPCO 15 -29.499 -25.563 -18.587 1.00 0.00 O2 O

ATOM 5920 O11 POPCO 15 -28.421 -27.771 -18.139 1.00 0.00 O2 O

ATOM 5921 C1 POPCO 15 -28.623 -28.124 -16.752 1.00 0.00 O2 C

ATOM 5922 HA POPCO 15 -28.773 -27.239 -16.165 1.00 0.00 O2 H

ATOM 5923 HB POPCO 15 -29.572 -28.685 -16.601 1.00 0.00 O2 H

ATOM 5924 C2 POPCO 15 -27.429 -28.849 -16.081 1.00 0.00 O2 C

ATOM 5925 HS POPCO 15 -27.653 -29.894 -16.294 1.00 0.00 O2 H

ATOM 5926 O21 POPCO 15 -27.436 -28.541 -14.704 1.00 0.00 O2 O

ATOM 5927 C21 POPCO 15 -26.774 -29.258 -13.868 1.00 0.00 O2 C

ATOM 5928 O22 POPCO 15 -26.053 -30.238 -14.007 1.00 0.00 O2 O

ATOM 5929 C22 POPCO 15 -27.060 -28.655 -12.421 1.00 0.00 O2 C

ATOM 5930 H2R POPCO 15 -26.564 -27.642 -12.552 1.00 0.00 O2 H

ATOM 5931 H2S POPCO 15 -28.086 -28.559 -12.100 1.00 0.00 O2 H

ATOM 5932 C3 POPCO 15 -26.103 -28.461 -16.756 1.00 0.00 O2 C

ATOM 5933 HX POPCO 15 -25.374 -29.037 -16.157 1.00 0.00 O2 H

ATOM 5934 HY POPCO 15 -25.951 -28.809 -17.856 1.00 0.00 O2 H

ATOM 5935 O31 POPCO 15 -25.898 -27.045 -16.731 1.00 0.00 O2 O

ATOM 5936 C31 POPCO 15 -24.747 -26.499 -17.168 1.00 0.00 O2 C

ATOM 5937 O32 POPCO 15 -23.904 -27.114 -17.781 1.00 0.00 O2 O

ATOM 5938 C32 POPCO 15 -24.709 -25.030 -16.738 1.00 0.00 O2 C

ATOM 5939 H2X POPCO 15 -23.653 -24.706 -16.717 1.00 0.00 O2 H

ATOM 5940 H2Y POPCO 15 -25.185 -24.284 -17.393 1.00 0.00 O2 H

ATOM 5941 C23 POPCO 15 -26.426 -29.403 -11.263 1.00 0.00 O2 C

ATOM 5942 H3R POPCO 15 -27.040 -30.350 -11.093 1.00 0.00 O2 H

ATOM 5943 H3S POPCO 15 -25.472 -29.847 -11.492 1.00 0.00 O2 H

ATOM 5944 C24 POPCO 15 -26.483 -28.628 -9.902 1.00 0.00 O2 C

ATOM 5945 H4R POPCO 15 -25.922 -27.693 -10.003 1.00 0.00 O2 H

ATOM 5946 H4S POPCO 15 -27.528 -28.382 -9.724 1.00 0.00 O2 H

ATOM 5947 C25 POPCO 15 -25.948 -29.482 -8.715 1.00 0.00 O2 C

ATOM 5948 H5R POPCO 15 -26.136 -30.577 -8.753 1.00 0.00 O2 H

ATOM 5949 H5S POPCO 15 -24.896 -29.454 -8.869 1.00 0.00 O2 H

ATOM 5950 C26 POPCO 15 -26.269 -28.896 -7.365 1.00 0.00 O2 C

ATOM 5951 H6R POPCO 15 -25.800 -27.852 -7.285 1.00 0.00 O2 H

ATOM 5952 H6S POPCO 15 -27.361 -28.804 -7.001 1.00 0.00 O2 H

ATOM 5953 C27 POPCO 15 -25.715 -29.860 -6.268 1.00 0.00 O2 C

ATOM 5954 H7R POPCO 15 -26.419 -30.708 -6.178 1.00 0.00 O2 H

ATOM 5955 H7S POPCO 15 -24.721 -30.283 -6.589 1.00 0.00 O2 H

ATOM 5956 C28 POPCO 15 -25.585 -29.125 -4.949 1.00 0.00 O2 C

ATOM 5957 H8R POPCO 15 -25.842 -28.065 -5.073 1.00 0.00 O2 H

ATOM 5958 H8S POPCO 15 -26.346 -29.564 -4.202 1.00 0.00 O2 H

ATOM 5959 C29 POPCO 15 -24.181 -29.211 -4.387 1.00 0.00 O2 C

ATOM 5960 H91 POPCO 15 -23.435 -28.871 -5.163 1.00 0.00 O2 H

ATOM 5961 C210 POPCO 15 -23.747 -29.603 -3.217 1.00 0.00 O2 C

ATOM 5962 H101 POPCO 15 -22.689 -29.690 -3.015 1.00 0.00 O2 H

ATOM 5963 C211 POPCO 15 -24.575 -30.118 -2.036 1.00 0.00 O2 C

ATOM 5964 H11R POPCO 15 -25.693 -30.029 -2.098 1.00 0.00 O2 H

ATOM 5965 H11S POPCO 15 -24.410 -31.225 -1.867 1.00 0.00 O2 H

ATOM 5966 C212 POPCO 15 -24.096 -29.492 -0.694 1.00 0.00 O2 C

ATOM 5967 H12R POPCO 15 -22.984 -29.618 -0.600 1.00 0.00 O2 H

ATOM 5968 H12S POPCO 15 -24.290 -28.484 -0.725 1.00 0.00 O2 H

ATOM 5969 C213 POPCO 15 -24.846 -29.914 0.615 1.00 0.00 O2 C

ATOM 5970 H13R POPCO 15 -25.873 -29.805 0.427 1.00 0.00 O2 H

ATOM 5971 H13S POPCO 15 -24.660 -31.007 0.650 1.00 0.00 O2 H

ATOM 5972 C214 POPCO 15 -24.457 -29.256 1.920 1.00 0.00 O2 C

ATOM 5973 H14R POPCO 15 -23.309 -29.174 2.024 1.00 0.00 O2 H

ATOM 5974 H14S POPCO 15 -24.808 -28.247 1.889 1.00 0.00 O2 H

ATOM 5975 C215 POPCO 15 -24.985 -29.971 3.234 1.00 0.00 O2 C

ATOM 5976 H15R POPCO 15 -26.103 -30.088 3.174 1.00 0.00 O2 H

ATOM 5977 H15S POPCO 15 -24.487 -30.939 3.310 1.00 0.00 O2 H

ATOM 5978 C216 POPCO 15 -24.518 -29.166 4.496 1.00 0.00 O2 C

ATOM 5979 H16R POPCO 15 -23.498 -28.939 4.428 1.00 0.00 O2 H

ATOM 5980 H16S POPCO 15 -25.052 -28.167 4.602 1.00 0.00 O2 H

ATOM 5981 C217 POPCO 15 -24.751 -29.746 5.859 1.00 0.00 O2 C

ATOM 5982 H17R POPCO 15 -25.854 -29.827 5.925 1.00 0.00 O2 H

ATOM 5983 H17S POPCO 15 -24.297 -30.763 5.911 1.00 0.00 O2 H

ATOM 5984 C218 POPCO 15 -24.210 -28.951 7.037 1.00 0.00 O2 C

ATOM 5985 H18R POPCO 15 -24.311 -29.387 8.045 1.00 0.00 O2 H

ATOM 5986 H18S POPCO 15 -23.140 -28.793 6.848 1.00 0.00 O2 H

ATOM 5987 H18T POPCO 15 -24.751 -27.942 7.072 1.00 0.00 O2 H

ATOM 5988 C33 POPCO 15 -25.269 -24.916 -15.313 1.00 0.00 O2 C

ATOM 5989 H3X POPCO 15 -25.344 -23.825 -15.187 1.00 0.00 O2 H

ATOM 5990 H3Y POPCO 15 -26.232 -25.340 -14.907 1.00 0.00 O2 H

ATOM 5991 C34 POPCO 15 -24.312 -25.588 -14.289 1.00 0.00 O2 C

ATOM 5992 H4X POPCO 15 -24.297 -26.653 -14.460 1.00 0.00 O2 H

ATOM 5993 H4Y POPCO 15 -23.329 -25.374 -14.634 1.00 0.00 O2 H

ATOM 5994 C35 POPCO 15 -24.553 -25.162 -12.835 1.00 0.00 O2 C

ATOM 5995 H5X POPCO 15 -24.282 -24.117 -12.704 1.00 0.00 O2 H

ATOM 5996 H5Y POPCO 15 -25.664 -25.329 -12.721 1.00 0.00 O2 H

ATOM 5997 C36 POPCO 15 -23.731 -26.038 -11.952 1.00 0.00 O2 C

ATOM 5998 H6X POPCO 15 -24.293 -26.985 -11.743 1.00 0.00 O2 H

ATOM 5999 H6Y POPCO 15 -22.739 -26.255 -12.419 1.00 0.00 O2 H

ATOM 6000 C37 POPCO 15 -23.580 -25.355 -10.575 1.00 0.00 O2 C

ATOM 6001 H7X POPCO 15 -24.504 -25.051 -10.106 1.00 0.00 O2 H

ATOM 6002 H7Y POPCO 15 -23.055 -24.407 -10.717 1.00 0.00 O2 H

ATOM 6003 C38 POPCO 15 -22.788 -26.166 -9.512 1.00 0.00 O2 C

ATOM 6004 H8X POPCO 15 -23.375 -27.110 -9.414 1.00 0.00 O2 H

ATOM 6005 H8Y POPCO 15 -21.752 -26.335 -9.886 1.00 0.00 O2 H

ATOM 6006 C39 POPCO 15 -22.636 -25.314 -8.288 1.00 0.00 O2 C

ATOM 6007 H9X POPCO 15 -21.695 -24.748 -8.229 1.00 0.00 O2 H

ATOM 6008 H9Y POPCO 15 -23.489 -24.623 -8.247 1.00 0.00 O2 H

ATOM 6009 C310 POPCO 15 -22.722 -26.242 -7.174 1.00 0.00 O2 C

ATOM 6010 H10X POPCO 15 -23.794 -26.600 -7.110 1.00 0.00 O2 H

ATOM 6011 H10Y POPCO 15 -22.036 -27.137 -7.161 1.00 0.00 O2 H

ATOM 6012 C311 POPCO 15 -22.438 -25.469 -5.862 1.00 0.00 O2 C

ATOM 6013 H11X POPCO 15 -21.330 -25.181 -5.737 1.00 0.00 O2 H

ATOM 6014 H11Y POPCO 15 -23.118 -24.601 -5.888 1.00 0.00 O2 H

ATOM 6015 C312 POPCO 15 -22.748 -26.274 -4.665 1.00 0.00 O2 C

ATOM 6016 H12X POPCO 15 -23.823 -26.601 -4.802 1.00 0.00 O2 H

ATOM 6017 H12Y POPCO 15 -22.117 -27.120 -4.581 1.00 0.00 O2 H

ATOM 6018 C313 POPCO 15 -22.636 -25.500 -3.328 1.00 0.00 O2 C

ATOM 6019 H13X POPCO 15 -21.599 -25.291 -3.191 1.00 0.00 O2 H

ATOM 6020 H13Y POPCO 15 -23.333 -24.662 -3.382 1.00 0.00 O2 H

ATOM 6021 C314 POPCO 15 -23.031 -26.362 -2.074 1.00 0.00 O2 C

ATOM 6022 H14X POPCO 15 -24.133 -26.563 -2.182 1.00 0.00 O2 H

ATOM 6023 H14Y POPCO 15 -22.483 -27.308 -2.078 1.00 0.00 O2 H

ATOM 6024 C315 POPCO 15 -22.842 -25.444 -0.830 1.00 0.00 O2 C

ATOM 6025 H15X POPCO 15 -21.743 -25.168 -0.785 1.00 0.00 O2 H

ATOM 6026 H15Y POPCO 15 -23.337 -24.482 -0.958 1.00 0.00 O2 H

ATOM 6027 C316 POPCO 15 -23.287 -26.084 0.440 1.00 0.00 O2 C

ATOM 6028 H16X POPCO 15 -22.728 -27.072 0.566 1.00 0.00 O2 H

ATOM 6029 H16Y POPCO 15 -22.845 -25.490 1.273 1.00 0.00 O2 H

ATOM 6030 H16Z POPCO 15 -24.407 -26.261 0.513 1.00 0.00 O2 H

ATOM 6031 N POPCO 16 -22.788 -17.377 -21.362 1.00 0.00 O2 N

ATOM 6032 C12 POPCO 16 -23.220 -17.414 -19.884 1.00 0.00 O2 C

ATOM 6033 H12A POPCO 16 -23.874 -16.635 -19.594 1.00 0.00 O2 H

ATOM 6034 H12B POPCO 16 -22.350 -17.046 -19.265 1.00 0.00 O2 H

ATOM 6035 C13 POPCO 16 -24.022 -17.320 -22.197 1.00 0.00 O2 C

ATOM 6036 H13A POPCO 16 -24.613 -16.467 -22.052 1.00 0.00 O2 H

ATOM 6037 H13B POPCO 16 -24.714 -18.098 -21.817 1.00 0.00 O2 H

ATOM 6038 H13C POPCO 16 -23.921 -17.422 -23.302 1.00 0.00 O2 H

ATOM 6039 C14 POPCO 16 -22.070 -16.123 -21.793 1.00 0.00 O2 C

ATOM 6040 H14A POPCO 16 -21.206 -16.057 -21.264 1.00 0.00 O2 H

ATOM 6041 H14B POPCO 16 -22.698 -15.247 -21.629 1.00 0.00 O2 H

ATOM 6042 H14C POPCO 16 -21.651 -16.131 -22.780 1.00 0.00 O2 H

ATOM 6043 C15 POPCO 16 -21.903 -18.594 -21.645 1.00 0.00 O2 C

ATOM 6044 H15A POPCO 16 -21.200 -18.674 -20.878 1.00 0.00 O2 H

ATOM 6045 H15B POPCO 16 -22.545 -19.424 -21.394 1.00 0.00 O2 H

ATOM 6046 H15C POPCO 16 -21.661 -18.675 -22.707 1.00 0.00 O2 H

ATOM 6047 C11 POPCO 16 -23.641 -18.744 -19.258 1.00 0.00 O2 C

ATOM 6048 H11A POPCO 16 -22.840 -19.512 -19.386 1.00 0.00 O2 H

ATOM 6049 H11B POPCO 16 -23.740 -18.512 -18.193 1.00 0.00 O2 H

ATOM 6050 P POPCO 16 -25.330 -20.803 -19.814 1.00 0.00 O2 P

ATOM 6051 O13 POPCO 16 -25.083 -21.320 -21.152 1.00 0.00 O2 O

ATOM 6052 O14 POPCO 16 -26.740 -20.960 -19.392 1.00 0.00 O2 O

ATOM 6053 O12 POPCO 16 -24.797 -19.235 -19.869 1.00 0.00 O2 O

ATOM 6054 O11 POPCO 16 -24.335 -21.592 -18.912 1.00 0.00 O2 O

ATOM 6055 C1 POPCO 16 -24.527 -21.197 -17.532 1.00 0.00 O2 C

ATOM 6056 HA POPCO 16 -24.794 -20.085 -17.338 1.00 0.00 O2 H

ATOM 6057 HB POPCO 16 -25.303 -21.817 -17.143 1.00 0.00 O2 H

ATOM 6058 C2 POPCO 16 -23.397 -21.536 -16.564 1.00 0.00 O2 C

ATOM 6059 HS POPCO 16 -23.329 -22.627 -16.539 1.00 0.00 O2 H

ATOM 6060 O21 POPCO 16 -23.682 -20.953 -15.306 1.00 0.00 O2 O

ATOM 6061 C21 POPCO 16 -23.080 -21.498 -14.233 1.00 0.00 O2 C

ATOM 6062 O22 POPCO 16 -22.405 -22.503 -14.177 1.00 0.00 O2 O

ATOM 6063 C22 POPCO 16 -23.455 -20.688 -13.023 1.00 0.00 O2 C

ATOM 6064 H2R POPCO 16 -22.609 -19.960 -12.897 1.00 0.00 O2 H

ATOM 6065 H2S POPCO 16 -24.424 -20.083 -13.151 1.00 0.00 O2 H

ATOM 6066 C3 POPCO 16 -22.018 -20.983 -17.023 1.00 0.00 O2 C

ATOM 6067 HX POPCO 16 -21.342 -21.364 -16.250 1.00 0.00 O2 H

ATOM 6068 HY POPCO 16 -21.803 -21.390 -18.082 1.00 0.00 O2 H

ATOM 6069 O31 POPCO 16 -21.820 -19.510 -17.076 1.00 0.00 O2 O

ATOM 6070 C31 POPCO 16 -20.578 -19.164 -17.440 1.00 0.00 O2 C

ATOM 6071 O32 POPCO 16 -19.737 -19.900 -17.898 1.00 0.00 O2 O

ATOM 6072 C32 POPCO 16 -20.208 -17.742 -16.917 1.00 0.00 O2 C

ATOM 6073 H2X POPCO 16 -19.160 -17.461 -17.274 1.00 0.00 O2 H

ATOM 6074 H2Y POPCO 16 -20.939 -17.054 -17.464 1.00 0.00 O2 H

ATOM 6075 C23 POPCO 16 -23.518 -21.467 -11.761 1.00 0.00 O2 C

ATOM 6076 H3R POPCO 16 -24.605 -21.904 -11.819 1.00 0.00 O2 H

ATOM 6077 H3S POPCO 16 -22.877 -22.346 -11.747 1.00 0.00 O2 H

ATOM 6078 C24 POPCO 16 -23.289 -20.705 -10.472 1.00 0.00 O2 C

ATOM 6079 H4R POPCO 16 -22.284 -20.143 -10.501 1.00 0.00 O2 H

ATOM 6080 H4S POPCO 16 -24.075 -19.901 -10.231 1.00 0.00 O2 H

ATOM 6081 C25 POPCO 16 -23.269 -21.676 -9.263 1.00 0.00 O2 C

ATOM 6082 H5R POPCO 16 -24.303 -22.118 -9.056 1.00 0.00 O2 H

ATOM 6083 H5S POPCO 16 -22.597 -22.567 -9.365 1.00 0.00 O2 H

ATOM 6084 C26 POPCO 16 -22.668 -20.959 -7.952 1.00 0.00 O2 C

ATOM 6085 H6R POPCO 16 -21.576 -21.032 -7.780 1.00 0.00 O2 H

ATOM 6086 H6S POPCO 16 -22.874 -19.909 -8.165 1.00 0.00 O2 H

ATOM 6087 C27 POPCO 16 -23.375 -21.433 -6.679 1.00 0.00 O2 C

ATOM 6088 H7R POPCO 16 -24.439 -20.939 -6.588 1.00 0.00 O2 H

ATOM 6089 H7S POPCO 16 -23.474 -22.563 -6.643 1.00 0.00 O2 H

ATOM 6090 C28 POPCO 16 -22.459 -20.914 -5.554 1.00 0.00 O2 C

ATOM 6091 H8R POPCO 16 -21.474 -21.254 -5.668 1.00 0.00 O2 H

ATOM 6092 H8S POPCO 16 -22.480 -19.784 -5.633 1.00 0.00 O2 H

ATOM 6093 C29 POPCO 16 -23.128 -21.284 -4.198 1.00 0.00 O2 C

ATOM 6094 H91 POPCO 16 -24.228 -21.154 -4.178 1.00 0.00 O2 H

ATOM 6095 C210 POPCO 16 -22.557 -21.678 -3.085 1.00 0.00 O2 C

ATOM 6096 H101 POPCO 16 -23.154 -21.769 -2.156 1.00 0.00 O2 H

ATOM 6097 C211 POPCO 16 -21.074 -21.689 -3.001 1.00 0.00 O2 C

ATOM 6098 H11R POPCO 16 -20.501 -21.959 -4.013 1.00 0.00 O2 H

ATOM 6099 H11S POPCO 16 -20.785 -20.717 -2.761 1.00 0.00 O2 H

ATOM 6100 C212 POPCO 16 -20.511 -22.516 -1.886 1.00 0.00 O2 C

ATOM 6101 H12R POPCO 16 -20.786 -23.586 -2.003 1.00 0.00 O2 H

ATOM 6102 H12S POPCO 16 -19.401 -22.585 -1.905 1.00 0.00 O2 H

ATOM 6103 C213 POPCO 16 -20.939 -22.083 -0.507 1.00 0.00 O2 C

ATOM 6104 H13R POPCO 16 -20.972 -20.942 -0.481 1.00 0.00 O2 H

ATOM 6105 H13S POPCO 16 -21.946 -22.456 -0.191 1.00 0.00 O2 H

ATOM 6106 C214 POPCO 16 -20.012 -22.601 0.562 1.00 0.00 O2 C

ATOM 6107 H14R POPCO 16 -19.854 -23.685 0.483 1.00 0.00 O2 H

ATOM 6108 H14S POPCO 16 -19.050 -22.072 0.530 1.00 0.00 O2 H

ATOM 6109 C215 POPCO 16 -20.707 -22.342 1.937 1.00 0.00 O2 C

ATOM 6110 H15R POPCO 16 -20.754 -21.267 1.995 1.00 0.00 O2 H

ATOM 6111 H15S POPCO 16 -21.722 -22.686 1.962 1.00 0.00 O2 H

ATOM 6112 C216 POPCO 16 -19.978 -22.788 3.228 1.00 0.00 O2 C

ATOM 6113 H16R POPCO 16 -20.130 -23.910 3.280 1.00 0.00 O2 H

ATOM 6114 H16S POPCO 16 -18.919 -22.619 3.235 1.00 0.00 O2 H

ATOM 6115 C217 POPCO 16 -20.759 -22.141 4.426 1.00 0.00 O2 C

ATOM 6116 H17R POPCO 16 -20.845 -21.073 4.234 1.00 0.00 O2 H

ATOM 6117 H17S POPCO 16 -21.792 -22.599 4.433 1.00 0.00 O2 H

ATOM 6118 C218 POPCO 16 -20.090 -22.413 5.798 1.00 0.00 O2 C

ATOM 6119 H18R POPCO 16 -20.547 -21.637 6.447 1.00 0.00 O2 H

ATOM 6120 H18S POPCO 16 -20.450 -23.351 6.041 1.00 0.00 O2 H

ATOM 6121 H18T POPCO 16 -18.937 -22.338 5.667 1.00 0.00 O2 H

ATOM 6122 C33 POPCO 16 -20.301 -17.604 -15.338 1.00 0.00 O2 C

ATOM 6123 H3X POPCO 16 -19.988 -16.598 -14.906 1.00 0.00 O2 H

ATOM 6124 H3Y POPCO 16 -21.404 -17.652 -15.037 1.00 0.00 O2 H

ATOM 6125 C34 POPCO 16 -19.651 -18.698 -14.589 1.00 0.00 O2 C

ATOM 6126 H4X POPCO 16 -20.341 -19.609 -14.588 1.00 0.00 O2 H

ATOM 6127 H4Y POPCO 16 -18.583 -18.857 -15.028 1.00 0.00 O2 H

ATOM 6128 C35 POPCO 16 -19.677 -18.416 -13.110 1.00 0.00 O2 C

ATOM 6129 H5X POPCO 16 -19.220 -17.409 -12.929 1.00 0.00 O2 H

ATOM 6130 H5Y POPCO 16 -20.742 -18.197 -12.839 1.00 0.00 O2 H

ATOM 6131 C36 POPCO 16 -19.191 -19.425 -12.091 1.00 0.00 O2 C

ATOM 6132 H6X POPCO 16 -19.747 -20.400 -12.036 1.00 0.00 O2 H

ATOM 6133 H6Y POPCO 16 -18.240 -19.755 -12.455 1.00 0.00 O2 H

ATOM 6134 C37 POPCO 16 -19.064 -18.852 -10.698 1.00 0.00 O2 C

ATOM 6135 H7X POPCO 16 -18.302 -18.022 -10.792 1.00 0.00 O2 H

ATOM 6136 H7Y POPCO 16 -20.055 -18.383 -10.383 1.00 0.00 O2 H

ATOM 6137 C38 POPCO 16 -18.602 -19.871 -9.671 1.00 0.00 O2 C

ATOM 6138 H8X POPCO 16 -19.456 -20.600 -9.479 1.00 0.00 O2 H

ATOM 6139 H8Y POPCO 16 -17.697 -20.416 -9.957 1.00 0.00 O2 H

ATOM 6140 C39 POPCO 16 -18.226 -19.172 -8.318 1.00 0.00 O2 C

ATOM 6141 H9X POPCO 16 -17.113 -18.981 -8.336 1.00 0.00 O2 H

ATOM 6142 H9Y POPCO 16 -18.731 -18.165 -8.213 1.00 0.00 O2 H

ATOM 6143 C310 POPCO 16 -18.683 -19.852 -7.040 1.00 0.00 O2 C

ATOM 6144 H10X POPCO 16 -19.791 -19.846 -6.945 1.00 0.00 O2 H

ATOM 6145 H10Y POPCO 16 -18.391 -20.895 -7.144 1.00 0.00 O2 H

ATOM 6146 C311 POPCO 16 -18.048 -19.346 -5.789 1.00 0.00 O2 C

ATOM 6147 H11X POPCO 16 -16.982 -19.687 -5.782 1.00 0.00 O2 H

ATOM 6148 H11Y POPCO 16 -18.131 -18.247 -5.811 1.00 0.00 O2 H

ATOM 6149 C312 POPCO 16 -18.693 -19.832 -4.517 1.00 0.00 O2 C

ATOM 6150 H12X POPCO 16 -19.802 -19.761 -4.456 1.00 0.00 O2 H

ATOM 6151 H12Y POPCO 16 -18.459 -20.949 -4.330 1.00 0.00 O2 H

ATOM 6152 C313 POPCO 16 -18.122 -19.086 -3.294 1.00 0.00 O2 C

ATOM 6153 H13X POPCO 16 -16.969 -19.287 -3.343 1.00 0.00 O2 H

ATOM 6154 H13Y POPCO 16 -18.285 -17.993 -3.570 1.00 0.00 O2 H

ATOM 6155 C314 POPCO 16 -18.586 -19.402 -1.842 1.00 0.00 O2 C

ATOM 6156 H14X POPCO 16 -19.752 -19.394 -1.795 1.00 0.00 O2 H

ATOM 6157 H14Y POPCO 16 -18.405 -20.409 -1.571 1.00 0.00 O2 H

ATOM 6158 C315 POPCO 16 -17.932 -18.527 -0.724 1.00 0.00 O2 C

ATOM 6159 H15X POPCO 16 -16.836 -18.693 -0.857 1.00 0.00 O2 H

ATOM 6160 H15Y POPCO 16 -18.038 -17.459 -1.076 1.00 0.00 O2 H

ATOM 6161 C316 POPCO 16 -18.347 -18.733 0.783 1.00 0.00 O2 C

ATOM 6162 H16X POPCO 16 -18.243 -19.807 1.167 1.00 0.00 O2 H

ATOM 6163 H16Y POPCO 16 -17.880 -18.132 1.511 1.00 0.00 O2 H

ATOM 6164 H16Z POPCO 16 -19.429 -18.476 0.908 1.00 0.00 O2 H

ATOM 6165 N POPCO 17 -27.885 -10.291 -21.702 1.00 0.00 O2 N

ATOM 6166 C12 POPCO 17 -28.077 -11.419 -20.706 1.00 0.00 O2 C

ATOM 6167 H12A POPCO 17 -28.507 -12.330 -21.295 1.00 0.00 O2 H

ATOM 6168 H12B POPCO 17 -28.773 -11.092 -19.901 1.00 0.00 O2 H

ATOM 6169 C13 POPCO 17 -27.092 -10.876 -22.859 1.00 0.00 O2 C

ATOM 6170 H13A POPCO 17 -27.588 -11.838 -23.190 1.00 0.00 O2 H

ATOM 6171 H13B POPCO 17 -26.041 -11.186 -22.582 1.00 0.00 O2 H

ATOM 6172 H13C POPCO 17 -27.045 -10.134 -23.708 1.00 0.00 O2 H

ATOM 6173 C14 POPCO 17 -29.155 -9.784 -22.093 1.00 0.00 O2 C

ATOM 6174 H14A POPCO 17 -29.815 -9.304 -21.343 1.00 0.00 O2 H

ATOM 6175 H14B POPCO 17 -29.680 -10.552 -22.757 1.00 0.00 O2 H

ATOM 6176 H14C POPCO 17 -28.999 -8.934 -22.743 1.00 0.00 O2 H

ATOM 6177 C15 POPCO 17 -27.123 -9.141 -21.047 1.00 0.00 O2 C

ATOM 6178 H15A POPCO 17 -27.762 -8.769 -20.200 1.00 0.00 O2 H

ATOM 6179 H15B POPCO 17 -26.169 -9.468 -20.774 1.00 0.00 O2 H

ATOM 6180 H15C POPCO 17 -27.085 -8.286 -21.716 1.00 0.00 O2 H

ATOM 6181 C11 POPCO 17 -26.907 -12.291 -20.136 1.00 0.00 O2 C

ATOM 6182 H11A POPCO 17 -25.972 -11.703 -20.111 1.00 0.00 O2 H

ATOM 6183 H11B POPCO 17 -27.164 -12.460 -19.113 1.00 0.00 O2 H

ATOM 6184 P POPCO 17 -25.704 -14.543 -20.854 1.00 0.00 O2 P

ATOM 6185 O13 POPCO 17 -25.475 -14.784 -22.300 1.00 0.00 O2 O

ATOM 6186 O14 POPCO 17 -25.982 -15.695 -20.008 1.00 0.00 O2 O

ATOM 6187 O12 POPCO 17 -26.834 -13.531 -20.855 1.00 0.00 O2 O

ATOM 6188 O11 POPCO 17 -24.410 -13.886 -20.173 1.00 0.00 O2 O

ATOM 6189 C1 POPCO 17 -24.220 -13.703 -18.757 1.00 0.00 O2 C

ATOM 6190 HA POPCO 17 -24.986 -13.018 -18.413 1.00 0.00 O2 H

ATOM 6191 HB POPCO 17 -24.396 -14.652 -18.280 1.00 0.00 O2 H

ATOM 6192 C2 POPCO 17 -22.861 -12.843 -18.482 1.00 0.00 O2 C

ATOM 6193 HS POPCO 17 -22.003 -13.530 -18.825 1.00 0.00 O2 H

ATOM 6194 O21 POPCO 17 -22.776 -12.536 -17.103 1.00 0.00 O2 O

ATOM 6195 C21 POPCO 17 -22.294 -13.435 -16.272 1.00 0.00 O2 C

ATOM 6196 O22 POPCO 17 -21.805 -14.495 -16.567 1.00 0.00 O2 O

ATOM 6197 C22 POPCO 17 -22.574 -12.957 -14.834 1.00 0.00 O2 C

ATOM 6198 H2R POPCO 17 -22.010 -11.955 -14.563 1.00 0.00 O2 H

ATOM 6199 H2S POPCO 17 -23.679 -12.674 -14.757 1.00 0.00 O2 H

ATOM 6200 C3 POPCO 17 -22.777 -11.539 -19.320 1.00 0.00 O2 C

ATOM 6201 HX POPCO 17 -21.796 -11.194 -19.162 1.00 0.00 O2 H

ATOM 6202 HY POPCO 17 -22.914 -11.680 -20.388 1.00 0.00 O2 H

ATOM 6203 O31 POPCO 17 -23.771 -10.598 -18.886 1.00 0.00 O2 O

ATOM 6204 C31 POPCO 17 -23.316 -9.380 -18.809 1.00 0.00 O2 C

ATOM 6205 O32 POPCO 17 -22.203 -8.965 -19.182 1.00 0.00 O2 O

ATOM 6206 C32 POPCO 17 -24.373 -8.481 -18.158 1.00 0.00 O2 C

ATOM 6207 H2X POPCO 17 -23.912 -7.456 -18.036 1.00 0.00 O2 H

ATOM 6208 H2Y POPCO 17 -25.342 -8.429 -18.728 1.00 0.00 O2 H

ATOM 6209 C23 POPCO 17 -22.243 -13.864 -13.659 1.00 0.00 O2 C

ATOM 6210 H3R POPCO 17 -22.748 -14.806 -13.885 1.00 0.00 O2 H

ATOM 6211 H3S POPCO 17 -21.192 -14.159 -13.645 1.00 0.00 O2 H

ATOM 6212 C24 POPCO 17 -22.739 -13.325 -12.321 1.00 0.00 O2 C

ATOM 6213 H4R POPCO 17 -22.295 -12.333 -12.153 1.00 0.00 O2 H

ATOM 6214 H4S POPCO 17 -23.836 -13.130 -12.591 1.00 0.00 O2 H

ATOM 6215 C25 POPCO 17 -22.555 -14.212 -11.051 1.00 0.00 O2 C

ATOM 6216 H5R POPCO 17 -22.903 -15.249 -11.149 1.00 0.00 O2 H

ATOM 6217 H5S POPCO 17 -21.534 -14.185 -10.650 1.00 0.00 O2 H

ATOM 6218 C26 POPCO 17 -23.531 -13.811 -9.914 1.00 0.00 O2 C

ATOM 6219 H6R POPCO 17 -23.272 -12.666 -9.686 1.00 0.00 O2 H

ATOM 6220 H6S POPCO 17 -24.540 -14.037 -10.200 1.00 0.00 O2 H

ATOM 6221 C27 POPCO 17 -23.234 -14.550 -8.606 1.00 0.00 O2 C

ATOM 6222 H7R POPCO 17 -23.099 -15.651 -8.853 1.00 0.00 O2 H

ATOM 6223 H7S POPCO 17 -22.300 -14.095 -8.248 1.00 0.00 O2 H

ATOM 6224 C28 POPCO 17 -24.363 -14.201 -7.610 1.00 0.00 O2 C

ATOM 6225 H8R POPCO 17 -24.397 -13.082 -7.623 1.00 0.00 O2 H

ATOM 6226 H8S POPCO 17 -25.403 -14.517 -7.998 1.00 0.00 O2 H

ATOM 6227 C29 POPCO 17 -24.106 -14.730 -6.198 1.00 0.00 O2 C

ATOM 6228 H91 POPCO 17 -24.391 -15.772 -6.030 1.00 0.00 O2 H

ATOM 6229 C210 POPCO 17 -23.461 -14.032 -5.188 1.00 0.00 O2 C

ATOM 6230 H101 POPCO 17 -23.349 -14.577 -4.232 1.00 0.00 O2 H

ATOM 6231 C211 POPCO 17 -22.858 -12.624 -5.235 1.00 0.00 O2 C

ATOM 6232 H11R POPCO 17 -21.749 -12.733 -4.912 1.00 0.00 O2 H

ATOM 6233 H11S POPCO 17 -22.854 -12.209 -6.262 1.00 0.00 O2 H

ATOM 6234 C212 POPCO 17 -23.613 -11.641 -4.287 1.00 0.00 O2 C

ATOM 6235 H12R POPCO 17 -23.278 -10.615 -4.587 1.00 0.00 O2 H

ATOM 6236 H12S POPCO 17 -24.744 -11.634 -4.342 1.00 0.00 O2 H

ATOM 6237 C213 POPCO 17 -23.099 -11.798 -2.871 1.00 0.00 O2 C

ATOM 6238 H13R POPCO 17 -23.364 -12.932 -2.737 1.00 0.00 O2 H

ATOM 6239 H13S POPCO 17 -21.986 -11.712 -2.813 1.00 0.00 O2 H

ATOM 6240 C214 POPCO 17 -23.912 -10.999 -1.872 1.00 0.00 O2 C

ATOM 6241 H14R POPCO 17 -23.623 -9.920 -1.991 1.00 0.00 O2 H

ATOM 6242 H14S POPCO 17 -24.999 -11.123 -1.957 1.00 0.00 O2 H

ATOM 6243 C215 POPCO 17 -23.299 -11.310 -0.497 1.00 0.00 O2 C

ATOM 6244 H15R POPCO 17 -23.610 -12.344 -0.233 1.00 0.00 O2 H

ATOM 6245 H15S POPCO 17 -22.157 -11.384 -0.536 1.00 0.00 O2 H

ATOM 6246 C216 POPCO 17 -23.848 -10.296 0.462 1.00 0.00 O2 C

ATOM 6247 H16R POPCO 17 -23.253 -9.369 0.436 1.00 0.00 O2 H

ATOM 6248 H16S POPCO 17 -24.907 -10.078 0.214 1.00 0.00 O2 H

ATOM 6249 C217 POPCO 17 -23.927 -10.711 1.929 1.00 0.00 O2 C

ATOM 6250 H17R POPCO 17 -24.784 -11.403 2.090 1.00 0.00 O2 H

ATOM 6251 H17S POPCO 17 -23.012 -11.170 2.307 1.00 0.00 O2 H

ATOM 6252 C218 POPCO 17 -24.053 -9.448 2.864 1.00 0.00 O2 C

ATOM 6253 H18R POPCO 17 -23.873 -9.760 3.892 1.00 0.00 O2 H

ATOM 6254 H18S POPCO 17 -23.354 -8.685 2.644 1.00 0.00 O2 H

ATOM 6255 H18T POPCO 17 -25.030 -8.949 2.801 1.00 0.00 O2 H

ATOM 6256 C33 POPCO 17 -24.802 -8.837 -16.686 1.00 0.00 O2 C

ATOM 6257 H3X POPCO 17 -23.821 -8.792 -16.030 1.00 0.00 O2 H

ATOM 6258 H3Y POPCO 17 -25.428 -7.979 -16.430 1.00 0.00 O2 H

ATOM 6259 C34 POPCO 17 -25.655 -10.113 -16.422 1.00 0.00 O2 C

ATOM 6260 H4X POPCO 17 -26.374 -10.350 -17.194 1.00 0.00 O2 H

ATOM 6261 H4Y POPCO 17 -24.960 -10.985 -16.314 1.00 0.00 O2 H

ATOM 6262 C35 POPCO 17 -26.373 -9.971 -15.028 1.00 0.00 O2 C

ATOM 6263 H5X POPCO 17 -26.772 -8.990 -14.939 1.00 0.00 O2 H

ATOM 6264 H5Y POPCO 17 -27.179 -10.706 -14.930 1.00 0.00 O2 H

ATOM 6265 C36 POPCO 17 -25.397 -10.038 -13.836 1.00 0.00 O2 C

ATOM 6266 H6X POPCO 17 -25.104 -11.120 -13.551 1.00 0.00 O2 H

ATOM 6267 H6Y POPCO 17 -24.471 -9.542 -14.072 1.00 0.00 O2 H

ATOM 6268 C37 POPCO 17 -26.005 -9.386 -12.549 1.00 0.00 O2 C

ATOM 6269 H7X POPCO 17 -26.060 -8.265 -12.727 1.00 0.00 O2 H

ATOM 6270 H7Y POPCO 17 -26.997 -9.859 -12.239 1.00 0.00 O2 H

ATOM 6271 C38 POPCO 17 -25.086 -9.271 -11.274 1.00 0.00 O2 C

ATOM 6272 H8X POPCO 17 -24.662 -10.222 -10.865 1.00 0.00 O2 H

ATOM 6273 H8Y POPCO 17 -24.292 -8.653 -11.651 1.00 0.00 O2 H

ATOM 6274 C39 POPCO 17 -25.851 -8.639 -10.071 1.00 0.00 O2 C

ATOM 6275 H9X POPCO 17 -25.982 -7.521 -10.323 1.00 0.00 O2 H

ATOM 6276 H9Y POPCO 17 -26.905 -9.069 -10.043 1.00 0.00 O2 H

ATOM 6277 C310 POPCO 17 -25.123 -8.923 -8.801 1.00 0.00 O2 C

ATOM 6278 H10X POPCO 17 -25.381 -9.933 -8.438 1.00 0.00 O2 H

ATOM 6279 H10Y POPCO 17 -24.001 -8.659 -9.056 1.00 0.00 O2 H

ATOM 6280 C311 POPCO 17 -25.522 -7.908 -7.808 1.00 0.00 O2 C

ATOM 6281 H11X POPCO 17 -25.207 -6.889 -8.222 1.00 0.00 O2 H

ATOM 6282 H11Y POPCO 17 -26.643 -7.841 -7.863 1.00 0.00 O2 H

ATOM 6283 C312 POPCO 17 -25.139 -8.233 -6.443 1.00 0.00 O2 C

ATOM 6284 H12X POPCO 17 -25.286 -9.312 -6.194 1.00 0.00 O2 H

ATOM 6285 H12Y POPCO 17 -24.011 -7.928 -6.444 1.00 0.00 O2 H

ATOM 6286 C313 POPCO 17 -25.899 -7.483 -5.349 1.00 0.00 O2 C

ATOM 6287 H13X POPCO 17 -25.896 -6.366 -5.404 1.00 0.00 O2 H

ATOM 6288 H13Y POPCO 17 -26.968 -7.845 -5.325 1.00 0.00 O2 H

ATOM 6289 C314 POPCO 17 -25.357 -7.971 -4.035 1.00 0.00 O2 C

ATOM 6290 H14X POPCO 17 -25.268 -9.066 -4.015 1.00 0.00 O2 H

ATOM 6291 H14Y POPCO 17 -24.327 -7.521 -3.948 1.00 0.00 O2 H

ATOM 6292 C315 POPCO 17 -26.263 -7.467 -2.895 1.00 0.00 O2 C

ATOM 6293 H15X POPCO 17 -26.374 -6.391 -2.911 1.00 0.00 O2 H

ATOM 6294 H15Y POPCO 17 -27.235 -7.958 -3.088 1.00 0.00 O2 H

ATOM 6295 C316 POPCO 17 -25.814 -7.910 -1.563 1.00 0.00 O2 C

ATOM 6296 H16X POPCO 17 -24.797 -7.496 -1.307 1.00 0.00 O2 H

ATOM 6297 H16Y POPCO 17 -26.542 -7.506 -0.775 1.00 0.00 O2 H

ATOM 6298 H16Z POPCO 17 -25.712 -9.036 -1.504 1.00 0.00 O2 H

ATOM 6299 N POPCO 18 -19.286 -5.185 -18.753 1.00 0.00 O2 N

ATOM 6300 C12 POPCO 18 -20.078 -4.313 -17.808 1.00 0.00 O2 C

ATOM 6301 H12A POPCO 18 -20.217 -3.302 -18.182 1.00 0.00 O2 H

ATOM 6302 H12B POPCO 18 -19.519 -4.351 -16.859 1.00 0.00 O2 H

ATOM 6303 C13 POPCO 18 -19.796 -5.123 -20.143 1.00 0.00 O2 C

ATOM 6304 H13A POPCO 18 -19.780 -4.153 -20.474 1.00 0.00 O2 H

ATOM 6305 H13B POPCO 18 -20.800 -5.627 -20.188 1.00 0.00 O2 H

ATOM 6306 H13C POPCO 18 -19.153 -5.828 -20.696 1.00 0.00 O2 H

ATOM 6307 C14 POPCO 18 -17.871 -4.802 -18.789 1.00 0.00 O2 C

ATOM 6308 H14A POPCO 18 -17.312 -5.116 -17.846 1.00 0.00 O2 H

ATOM 6309 H14B POPCO 18 -17.858 -3.729 -18.840 1.00 0.00 O2 H

ATOM 6310 H14C POPCO 18 -17.280 -5.159 -19.742 1.00 0.00 O2 H

ATOM 6311 C15 POPCO 18 -19.375 -6.626 -18.373 1.00 0.00 O2 C

ATOM 6312 H15A POPCO 18 -18.811 -6.894 -17.538 1.00 0.00 O2 H

ATOM 6313 H15B POPCO 18 -20.432 -6.915 -18.242 1.00 0.00 O2 H

ATOM 6314 H15C POPCO 18 -19.043 -7.291 -19.093 1.00 0.00 O2 H

ATOM 6315 C11 POPCO 18 -21.541 -4.859 -17.515 1.00 0.00 O2 C

ATOM 6316 H11A POPCO 18 -21.850 -5.464 -18.464 1.00 0.00 O2 H

ATOM 6317 H11B POPCO 18 -21.640 -5.505 -16.667 1.00 0.00 O2 H

ATOM 6318 P POPCO 18 -23.973 -4.050 -17.675 1.00 0.00 O2 P

ATOM 6319 O13 POPCO 18 -24.068 -5.052 -18.775 1.00 0.00 O2 O

ATOM 6320 O14 POPCO 18 -24.753 -2.886 -18.058 1.00 0.00 O2 O

ATOM 6321 O12 POPCO 18 -22.420 -3.708 -17.499 1.00 0.00 O2 O

ATOM 6322 O11 POPCO 18 -24.457 -4.758 -16.335 1.00 0.00 O2 O

ATOM 6323 C1 POPCO 18 -24.015 -4.021 -15.183 1.00 0.00 O2 C

ATOM 6324 HA POPCO 18 -22.913 -3.919 -15.191 1.00 0.00 O2 H

ATOM 6325 HB POPCO 18 -24.538 -3.008 -14.955 1.00 0.00 O2 H

ATOM 6326 C2 POPCO 18 -24.427 -4.899 -13.884 1.00 0.00 O2 C

ATOM 6327 HS POPCO 18 -25.513 -5.125 -14.005 1.00 0.00 O2 H

ATOM 6328 O21 POPCO 18 -24.251 -4.054 -12.721 1.00 0.00 O2 O

ATOM 6329 C21 POPCO 18 -25.042 -4.326 -11.711 1.00 0.00 O2 C

ATOM 6330 O22 POPCO 18 -25.809 -5.239 -11.484 1.00 0.00 O2 O

ATOM 6331 C22 POPCO 18 -24.883 -3.230 -10.746 1.00 0.00 O2 C

ATOM 6332 H2R POPCO 18 -23.901 -2.828 -10.703 1.00 0.00 O2 H

ATOM 6333 H2S POPCO 18 -25.523 -2.372 -11.003 1.00 0.00 O2 H

ATOM 6334 C3 POPCO 18 -23.763 -6.291 -13.752 1.00 0.00 O2 C

ATOM 6335 HX POPCO 18 -24.141 -6.927 -12.859 1.00 0.00 O2 H

ATOM 6336 HY POPCO 18 -23.953 -6.816 -14.676 1.00 0.00 O2 H

ATOM 6337 O31 POPCO 18 -22.354 -6.025 -13.558 1.00 0.00 O2 O

ATOM 6338 C31 POPCO 18 -21.634 -7.122 -13.527 1.00 0.00 O2 C

ATOM 6339 O32 POPCO 18 -22.022 -8.251 -13.862 1.00 0.00 O2 O

ATOM 6340 C32 POPCO 18 -20.184 -6.729 -13.312 1.00 0.00 O2 C

ATOM 6341 H2X POPCO 18 -19.603 -7.683 -13.435 1.00 0.00 O2 H

ATOM 6342 H2Y POPCO 18 -19.743 -5.973 -14.015 1.00 0.00 O2 H

ATOM 6343 C23 POPCO 18 -25.163 -3.694 -9.286 1.00 0.00 O2 C

ATOM 6344 H3R POPCO 18 -26.213 -3.520 -9.125 1.00 0.00 O2 H

ATOM 6345 H3S POPCO 18 -24.942 -4.761 -9.171 1.00 0.00 O2 H

ATOM 6346 C24 POPCO 18 -24.300 -2.902 -8.171 1.00 0.00 O2 C

ATOM 6347 H4R POPCO 18 -23.231 -2.874 -8.427 1.00 0.00 O2 H

ATOM 6348 H4S POPCO 18 -24.659 -1.869 -8.248 1.00 0.00 O2 H

ATOM 6349 C25 POPCO 18 -24.552 -3.446 -6.759 1.00 0.00 O2 C

ATOM 6350 H5R POPCO 18 -25.599 -3.079 -6.502 1.00 0.00 O2 H

ATOM 6351 H5S POPCO 18 -24.529 -4.548 -6.783 1.00 0.00 O2 H

ATOM 6352 C26 POPCO 18 -23.577 -3.081 -5.632 1.00 0.00 O2 C

ATOM 6353 H6R POPCO 18 -22.526 -3.313 -5.938 1.00 0.00 O2 H

ATOM 6354 H6S POPCO 18 -23.550 -1.977 -5.377 1.00 0.00 O2 H

ATOM 6355 C27 POPCO 18 -23.930 -3.876 -4.365 1.00 0.00 O2 C

ATOM 6356 H7R POPCO 18 -24.876 -3.423 -3.923 1.00 0.00 O2 H

ATOM 6357 H7S POPCO 18 -24.059 -4.946 -4.673 1.00 0.00 O2 H

ATOM 6358 C28 POPCO 18 -22.875 -3.980 -3.204 1.00 0.00 O2 C

ATOM 6359 H8R POPCO 18 -21.934 -4.261 -3.644 1.00 0.00 O2 H

ATOM 6360 H8S POPCO 18 -22.695 -2.899 -2.805 1.00 0.00 O2 H

ATOM 6361 C29 POPCO 18 -23.402 -4.953 -2.177 1.00 0.00 O2 C

ATOM 6362 H91 POPCO 18 -23.623 -5.943 -2.548 1.00 0.00 O2 H

ATOM 6363 C210 POPCO 18 -23.587 -4.716 -0.820 1.00 0.00 O2 C

ATOM 6364 H101 POPCO 18 -23.963 -5.599 -0.266 1.00 0.00 O2 H

ATOM 6365 C211 POPCO 18 -23.270 -3.462 -0.010 1.00 0.00 O2 C

ATOM 6366 H11R POPCO 18 -22.641 -2.782 -0.683 1.00 0.00 O2 H

ATOM 6367 H11S POPCO 18 -24.118 -2.941 0.455 1.00 0.00 O2 H

ATOM 6368 C212 POPCO 18 -22.402 -3.901 1.212 1.00 0.00 O2 C

ATOM 6369 H12R POPCO 18 -21.479 -4.332 0.748 1.00 0.00 O2 H

ATOM 6370 H12S POPCO 18 -22.133 -2.994 1.761 1.00 0.00 O2 H

ATOM 6371 C213 POPCO 18 -23.196 -4.950 2.018 1.00 0.00 O2 C

ATOM 6372 H13R POPCO 18 -24.322 -4.658 2.089 1.00 0.00 O2 H

ATOM 6373 H13S POPCO 18 -23.030 -5.966 1.519 1.00 0.00 O2 H

ATOM 6374 C214 POPCO 18 -22.733 -5.017 3.445 1.00 0.00 O2 C

ATOM 6375 H14R POPCO 18 -21.655 -4.787 3.481 1.00 0.00 O2 H

ATOM 6376 H14S POPCO 18 -23.014 -4.090 3.976 1.00 0.00 O2 H

ATOM 6377 C215 POPCO 18 -23.227 -6.237 4.187 1.00 0.00 O2 C

ATOM 6378 H15R POPCO 18 -24.228 -6.513 3.874 1.00 0.00 O2 H

ATOM 6379 H15S POPCO 18 -22.499 -7.034 3.958 1.00 0.00 O2 H

ATOM 6380 C216 POPCO 18 -23.313 -6.084 5.697 1.00 0.00 O2 C

ATOM 6381 H16R POPCO 18 -22.389 -5.987 6.224 1.00 0.00 O2 H

ATOM 6382 H16S POPCO 18 -23.977 -5.172 5.920 1.00 0.00 O2 H

ATOM 6383 C217 POPCO 18 -23.811 -7.360 6.220 1.00 0.00 O2 C

ATOM 6384 H17R POPCO 18 -24.716 -7.664 5.691 1.00 0.00 O2 H

ATOM 6385 H17S POPCO 18 -23.120 -8.241 5.976 1.00 0.00 O2 H

ATOM 6386 C218 POPCO 18 -23.996 -7.239 7.773 1.00 0.00 O2 C

ATOM 6387 H18R POPCO 18 -24.393 -8.218 8.126 1.00 0.00 O2 H

ATOM 6388 H18S POPCO 18 -23.017 -6.854 8.210 1.00 0.00 O2 H

ATOM 6389 H18T POPCO 18 -24.773 -6.551 8.034 1.00 0.00 O2 H

ATOM 6390 C33 POPCO 18 -19.942 -6.127 -11.860 1.00 0.00 O2 C

ATOM 6391 H3X POPCO 18 -18.898 -5.938 -11.636 1.00 0.00 O2 H

ATOM 6392 H3Y POPCO 18 -20.449 -5.200 -11.829 1.00 0.00 O2 H

ATOM 6393 C34 POPCO 18 -20.589 -7.071 -10.822 1.00 0.00 O2 C

ATOM 6394 H4X POPCO 18 -21.699 -7.110 -10.816 1.00 0.00 O2 H

ATOM 6395 H4Y POPCO 18 -20.180 -8.107 -10.925 1.00 0.00 O2 H

ATOM 6396 C35 POPCO 18 -20.154 -6.682 -9.390 1.00 0.00 O2 C

ATOM 6397 H5X POPCO 18 -19.052 -7.047 -9.336 1.00 0.00 O2 H

ATOM 6398 H5Y POPCO 18 -20.184 -5.556 -9.213 1.00 0.00 O2 H

ATOM 6399 C36 POPCO 18 -20.883 -7.389 -8.264 1.00 0.00 O2 C

ATOM 6400 H6X POPCO 18 -21.873 -7.002 -8.202 1.00 0.00 O2 H

ATOM 6401 H6Y POPCO 18 -20.956 -8.504 -8.599 1.00 0.00 O2 H

ATOM 6402 C37 POPCO 18 -20.144 -7.227 -6.920 1.00 0.00 O2 C

ATOM 6403 H7X POPCO 18 -19.183 -7.820 -6.955 1.00 0.00 O2 H

ATOM 6404 H7Y POPCO 18 -19.893 -6.196 -6.908 1.00 0.00 O2 H

ATOM 6405 C38 POPCO 18 -20.977 -7.555 -5.635 1.00 0.00 O2 C

ATOM 6406 H8X POPCO 18 -21.780 -6.709 -5.558 1.00 0.00 O2 H

ATOM 6407 H8Y POPCO 18 -21.485 -8.499 -5.627 1.00 0.00 O2 H

ATOM 6408 C39 POPCO 18 -20.063 -7.462 -4.410 1.00 0.00 O2 C

ATOM 6409 H9X POPCO 18 -19.313 -8.283 -4.569 1.00 0.00 O2 H

ATOM 6410 H9Y POPCO 18 -19.571 -6.510 -4.389 1.00 0.00 O2 H

ATOM 6411 C310 POPCO 18 -20.847 -7.748 -3.112 1.00 0.00 O2 C

ATOM 6412 H10X POPCO 18 -21.614 -6.907 -2.864 1.00 0.00 O2 H

ATOM 6413 H10Y POPCO 18 -21.302 -8.790 -3.206 1.00 0.00 O2 H

ATOM 6414 C311 POPCO 18 -19.854 -7.751 -1.870 1.00 0.00 O2 C

ATOM 6415 H11X POPCO 18 -19.030 -8.497 -1.853 1.00 0.00 O2 H

ATOM 6416 H11Y POPCO 18 -19.254 -6.854 -1.927 1.00 0.00 O2 H

ATOM 6417 C312 POPCO 18 -20.484 -7.701 -0.474 1.00 0.00 O2 C

ATOM 6418 H12X POPCO 18 -21.106 -6.863 -0.365 1.00 0.00 O2 H

ATOM 6419 H12Y POPCO 18 -21.118 -8.633 -0.226 1.00 0.00 O2 H

ATOM 6420 C313 POPCO 18 -19.588 -7.682 0.716 1.00 0.00 O2 C

ATOM 6421 H13X POPCO 18 -19.005 -8.630 0.705 1.00 0.00 O2 H

ATOM 6422 H13Y POPCO 18 -18.827 -6.892 0.609 1.00 0.00 O2 H

ATOM 6423 C314 POPCO 18 -20.317 -7.599 2.109 1.00 0.00 O2 C

ATOM 6424 H14X POPCO 18 -20.823 -6.659 2.120 1.00 0.00 O2 H

ATOM 6425 H14Y POPCO 18 -21.222 -8.191 2.067 1.00 0.00 O2 H

ATOM 6426 C315 POPCO 18 -19.362 -7.963 3.230 1.00 0.00 O2 C

ATOM 6427 H15X POPCO 18 -18.801 -8.875 2.923 1.00 0.00 O2 H

ATOM 6428 H15Y POPCO 18 -18.603 -7.218 3.248 1.00 0.00 O2 H

ATOM 6429 C316 POPCO 18 -20.010 -8.161 4.610 1.00 0.00 O2 C

ATOM 6430 H16X POPCO 18 -20.882 -8.904 4.589 1.00 0.00 O2 H

ATOM 6431 H16Y POPCO 18 -19.190 -8.574 5.297 1.00 0.00 O2 H

ATOM 6432 H16Z POPCO 18 -20.402 -7.186 4.887 1.00 0.00 O2 H

ATOM 6433 N POPCO 19 -17.377 -41.448 -24.051 1.00 0.00 O2 N

ATOM 6434 C12 POPCO 19 -17.240 -42.307 -22.825 1.00 0.00 O2 C

ATOM 6435 H12A POPCO 19 -16.703 -43.216 -23.041 1.00 0.00 O2 H

ATOM 6436 H12B POPCO 19 -18.273 -42.576 -22.462 1.00 0.00 O2 H

ATOM 6437 C13 POPCO 19 -16.113 -41.453 -24.930 1.00 0.00 O2 C

ATOM 6438 H13A POPCO 19 -15.866 -42.522 -25.221 1.00 0.00 O2 H

ATOM 6439 H13B POPCO 19 -15.349 -40.965 -24.367 1.00 0.00 O2 H

ATOM 6440 H13C POPCO 19 -16.095 -40.901 -25.894 1.00 0.00 O2 H

ATOM 6441 C14 POPCO 19 -18.604 -41.899 -24.731 1.00 0.00 O2 C

ATOM 6442 H14A POPCO 19 -19.405 -41.751 -23.980 1.00 0.00 O2 H

ATOM 6443 H14B POPCO 19 -18.716 -42.877 -24.996 1.00 0.00 O2 H

ATOM 6444 H14C POPCO 19 -18.814 -41.212 -25.585 1.00 0.00 O2 H

ATOM 6445 C15 POPCO 19 -17.457 -39.975 -23.683 1.00 0.00 O2 C

ATOM 6446 H15A POPCO 19 -18.268 -39.908 -22.926 1.00 0.00 O2 H

ATOM 6447 H15B POPCO 19 -16.520 -39.753 -23.174 1.00 0.00 O2 H

ATOM 6448 H15C POPCO 19 -17.786 -39.489 -24.581 1.00 0.00 O2 H

ATOM 6449 C11 POPCO 19 -16.426 -41.783 -21.665 1.00 0.00 O2 C

ATOM 6450 H11A POPCO 19 -16.798 -40.790 -21.445 1.00 0.00 O2 H

ATOM 6451 H11B POPCO 19 -16.666 -42.389 -20.828 1.00 0.00 O2 H

ATOM 6452 P POPCO 19 -13.942 -40.750 -22.064 1.00 0.00 O2 P

ATOM 6453 O13 POPCO 19 -14.090 -40.160 -23.395 1.00 0.00 O2 O

ATOM 6454 O14 POPCO 19 -12.553 -41.170 -21.795 1.00 0.00 O2 O

ATOM 6455 O12 POPCO 19 -15.026 -41.897 -22.010 1.00 0.00 O2 O

ATOM 6456 O11 POPCO 19 -14.460 -39.694 -21.000 1.00 0.00 O2 O

ATOM 6457 C1 POPCO 19 -14.540 -40.014 -19.642 1.00 0.00 O2 C

ATOM 6458 HA POPCO 19 -15.093 -40.992 -19.429 1.00 0.00 O2 H

ATOM 6459 HB POPCO 19 -13.497 -40.069 -19.225 1.00 0.00 O2 H

ATOM 6460 C2 POPCO 19 -15.251 -38.954 -18.824 1.00 0.00 O2 C

ATOM 6461 HS POPCO 19 -14.681 -38.020 -19.094 1.00 0.00 O2 H

ATOM 6462 O21 POPCO 19 -15.156 -39.314 -17.432 1.00 0.00 O2 O

ATOM 6463 C21 POPCO 19 -14.311 -38.666 -16.676 1.00 0.00 O2 C

ATOM 6464 O22 POPCO 19 -13.523 -37.808 -17.030 1.00 0.00 O2 O

ATOM 6465 C22 POPCO 19 -14.383 -39.168 -15.233 1.00 0.00 O2 C

ATOM 6466 H2R POPCO 19 -14.417 -40.316 -15.184 1.00 0.00 O2 H

ATOM 6467 H2S POPCO 19 -13.474 -38.988 -14.664 1.00 0.00 O2 H

ATOM 6468 C3 POPCO 19 -16.758 -38.689 -19.191 1.00 0.00 O2 C

ATOM 6469 HX POPCO 19 -17.065 -37.791 -18.530 1.00 0.00 O2 H

ATOM 6470 HY POPCO 19 -16.908 -38.280 -20.200 1.00 0.00 O2 H

ATOM 6471 O31 POPCO 19 -17.737 -39.709 -18.840 1.00 0.00 O2 O

ATOM 6472 C31 POPCO 19 -18.755 -39.939 -19.684 1.00 0.00 O2 C

ATOM 6473 O32 POPCO 19 -18.860 -39.408 -20.785 1.00 0.00 O2 O

ATOM 6474 C32 POPCO 19 -20.005 -40.704 -19.127 1.00 0.00 O2 C

ATOM 6475 H2X POPCO 19 -20.694 -39.882 -18.688 1.00 0.00 O2 H

ATOM 6476 H2Y POPCO 19 -20.496 -41.132 -19.922 1.00 0.00 O2 H

ATOM 6477 C23 POPCO 19 -15.592 -38.523 -14.555 1.00 0.00 O2 C

ATOM 6478 H3R POPCO 19 -15.491 -37.452 -14.275 1.00 0.00 O2 H

ATOM 6479 H3S POPCO 19 -16.507 -38.642 -15.208 1.00 0.00 O2 H

ATOM 6480 C24 POPCO 19 -15.892 -39.207 -13.233 1.00 0.00 O2 C

ATOM 6481 H4R POPCO 19 -16.761 -39.933 -13.369 1.00 0.00 O2 H

ATOM 6482 H4S POPCO 19 -15.077 -39.851 -12.797 1.00 0.00 O2 H

ATOM 6483 C25 POPCO 19 -16.354 -38.249 -12.054 1.00 0.00 O2 C

ATOM 6484 H5R POPCO 19 -15.510 -37.728 -11.593 1.00 0.00 O2 H

ATOM 6485 H5S POPCO 19 -16.997 -37.489 -12.602 1.00 0.00 O2 H

ATOM 6486 C26 POPCO 19 -16.991 -39.164 -10.884 1.00 0.00 O2 C

ATOM 6487 H6R POPCO 19 -18.083 -39.237 -10.985 1.00 0.00 O2 H

ATOM 6488 H6S POPCO 19 -16.596 -40.218 -10.963 1.00 0.00 O2 H

ATOM 6489 C27 POPCO 19 -16.765 -38.501 -9.476 1.00 0.00 O2 C

ATOM 6490 H7R POPCO 19 -15.688 -38.278 -9.362 1.00 0.00 O2 H

ATOM 6491 H7S POPCO 19 -17.384 -37.604 -9.558 1.00 0.00 O2 H

ATOM 6492 C28 POPCO 19 -17.392 -39.295 -8.256 1.00 0.00 O2 C

ATOM 6493 H8R POPCO 19 -18.456 -39.249 -8.390 1.00 0.00 O2 H

ATOM 6494 H8S POPCO 19 -17.196 -40.380 -8.203 1.00 0.00 O2 H

ATOM 6495 C29 POPCO 19 -17.181 -38.455 -6.967 1.00 0.00 O2 C

ATOM 6496 H91 POPCO 19 -17.146 -37.421 -7.026 1.00 0.00 O2 H

ATOM 6497 C210 POPCO 19 -17.153 -39.016 -5.764 1.00 0.00 O2 C

ATOM 6498 H101 POPCO 19 -17.033 -38.292 -4.953 1.00 0.00 O2 H

ATOM 6499 C211 POPCO 19 -17.204 -40.445 -5.392 1.00 0.00 O2 C

ATOM 6500 H11R POPCO 19 -17.607 -41.071 -6.253 1.00 0.00 O2 H

ATOM 6501 H11S POPCO 19 -16.136 -40.803 -5.172 1.00 0.00 O2 H

ATOM 6502 C212 POPCO 19 -18.053 -40.837 -4.215 1.00 0.00 O2 C

ATOM 6503 H12R POPCO 19 -19.142 -40.538 -4.246 1.00 0.00 O2 H

ATOM 6504 H12S POPCO 19 -17.863 -41.950 -4.063 1.00 0.00 O2 H

ATOM 6505 C213 POPCO 19 -17.707 -40.081 -2.919 1.00 0.00 O2 C

ATOM 6506 H13R POPCO 19 -16.590 -40.242 -2.717 1.00 0.00 O2 H

ATOM 6507 H13S POPCO 19 -18.034 -39.025 -2.959 1.00 0.00 O2 H

ATOM 6508 C214 POPCO 19 -18.495 -40.691 -1.779 1.00 0.00 O2 C

ATOM 6509 H14R POPCO 19 -19.554 -40.637 -1.896 1.00 0.00 O2 H

ATOM 6510 H14S POPCO 19 -18.235 -41.799 -1.831 1.00 0.00 O2 H

ATOM 6511 C215 POPCO 19 -18.111 -40.151 -0.381 1.00 0.00 O2 C

ATOM 6512 H15R POPCO 19 -16.959 -40.188 -0.291 1.00 0.00 O2 H

ATOM 6513 H15S POPCO 19 -18.444 -39.101 -0.421 1.00 0.00 O2 H

ATOM 6514 C216 POPCO 19 -18.745 -40.892 0.790 1.00 0.00 O2 C

ATOM 6515 H16R POPCO 19 -19.820 -40.617 0.751 1.00 0.00 O2 H

ATOM 6516 H16S POPCO 19 -18.666 -42.025 0.662 1.00 0.00 O2 H

ATOM 6517 C217 POPCO 19 -18.310 -40.273 2.086 1.00 0.00 O2 C
[truncated: 2,118,100 more chars]
